# A Mono‐Substituted Silicon(II) Cation: A Crystalline “Supersilylene”

**DOI:** 10.1002/anie.202009874

**Published:** 2020-08-26

**Authors:** Alexander Hinz

**Affiliations:** ^1^ Karlsruher Institut für Technologie Institut für Anorganische Chemie Engesserstraße 15 76131 Karlsruhe Germany

**Keywords:** cation, crystal structure, silicon, silylene, steric bulk

## Abstract

Mono‐coordinated silicon(II) cations are predicted to be reactive ambiphiles, combining the typically high Lewis acidity of silicon cations with nucleophilicity due to the presence of an electron pair at the same atomic centre. Here, a carbazole‐derived scaffold was used to isolate salts with a mono‐coordinated silicon(II) cation, [RSi]^+^ (R=bulky carbazolyl substituent), by halide abstraction from a base‐free halosilylene, RSiI, with Ag[Al(O^*t*^Bu^F^)_4_]. Despite the bulk of the carbazolyl moiety, the silylenylium cation [RSi]^+^ retains high reactivity. It was shown to react with an amine to form three bonds at the silicon atom in one reaction which conforms with the notion of a “supersilylene”. The resulting silylium cation [RSi(H)NR′_2_]^+^ (in the formal oxidation state Si^IV^) obtained by oxidative addition of an NH bond at [RSi]^+^ is even more acidic than the silylenylium cation (Si^II^) due to the absence of a lone pair of electrons the silicon atom.

## Graphical abstract

A mono‐coordinated silicon cation was prepared by halide abstraction from a iodosilylene. It is shown to react with an amine to form three bonds at the silicon atom in one reaction which conforms with the notion of a “supersilylene”.

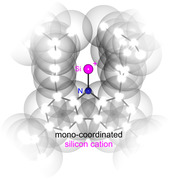

Silylium cations ([R′_3_Si]^+^, R′=monodentate monoanionic substituent) are of great interest as they are considerably more Lewis acidic than their lighter congeners, carbenium cations (R′_3_C^+^).^1^ In 1993, crystallographic evidence for the existence of adducts of such silylium cations was provided for the first time by the groups of Reed and Lambert with the examples of [^*i*^Pr_3_Si(MeCN)]^+^ and [^*i*^Pr_3_Si(CB_9_H_5_Br_5_)] as well as [Et_3_Si(toluene)]^+^.^2^, ^3^ In these three examples, the silicon atom adopts tetracoordination and the positive charge is predominantly localized on the coordinated donor moieties. It took the groups nine more years to achieve the isolation of salts with the donor‐free genuine silylium cation [Mes_3_Si]^+^ (Mes=2,4,6‐trimethylphenyl).^4^ Since then, the reactivity of silylium cations has been intensively studied and was found to be dominated by extreme Lewis acidity which makes them suitable catalysts for reactions such as hydrodefluorinations, C−H arylations and C−H alkylations.^5^ In contrast, Si^II^ compounds in their singlet ground state are generally ambiphilic, as their Si atom has a lone pair of electrons but only possesses an electron sextet. West's landmark report detailing the isolation of the first silylene in 1994 initiated the investigation of their reactivity.^6^ Three general reactivity patterns can be distinguished as follows: a) Silylenes can act as Lewis acids. For instance, Roesky and Filippou found independently that simple silylenes such as SiCl_2_ and SiBr_2_ can be stabilized by suitable carbene donors to make molecular synthetic equivalents of Si^II^ available.^7^, ^8^ b) Silylenes can act as a base themselves. Silylenes are strong σ‐donor ligands which can be incorporated in transition metal complexes to be utilized in catalysis.^9^, ^10^ c) The presence of both Lewis acidic and basic properties at the silicon atom can make them reactive ambiphiles. Many silylenes react with small molecules^11^, ^12^ such as H_2_, C_2_H_2_, NH_3_, or CO_2_ as shown by Aldridge,^13^ Driess,^14^ Iwamoto,^15^ Roesky,^16^, ^17^ and Inoue.^18^

A combination of enhanced Lewis acidity and ambiphilicity was predicted for cationic Si^II^ species. These molecules of the general formula [R′Si]^+^ have been referred to with a variety of names, such as silanetriyl, silyne, or silyliumylidene cations, among which the latter is the currently dominating term. More consistently, they could be called silaylidenylium or silylenylium ions, the latter of which will be used in this article. These Si^II^ cations have been pursued intensively. A mass‐spectrometric study by Gaspar targeting the reactivity of [HSi]^+^ with diethylamine (HNEt_2_) in the gas phase indicated that three bonds are formed in a single reactive encounter which prompted the authors to coin the phrase “supersilylenes” for these cations.^19^, ^20^ Numerous attempts to isolate such mono‐coordinated cations were unsuccessful, but Si^II^ cations with a higher coordination number could be obtained, such as Jutzi's [Cp*Si]^+^ and masked Si^II^ cations of the general formula [R′Si(L)_*x*_]^+^ (L=neutral donor ligand).^21^, ^22^, ^23^, ^24^, ^25^, ^26^, ^27^, ^28^ In all these examples, the donor‐stabilisation causes a coordination number greater than one which prevents the envisaged three‐bond‐forming‐reactivity.^29^ Müller spectroscopically observed that by thermal decomposition of a dibenzosilanorbornadienyl cation in benzene solution an aryl diphenyl silylium ion was formed which indicates that a Si^II^ cation benzene had been intermediarily present.^23^ To date, the lack of substituents which provide sufficient shielding for the stabilisation of free silyliumylidenes has led to the notion that “Clearly, such steric protection cannot be achieved by the lonely substituent of silyliumylidenes” (R′Si^+^).^30^

After the recent synthesis of heavier chlorotetrylenes and the corresponding tetrylenium cations bearing a sterically demanding carbazolyl substituent (R, *R*=1,8‐bis(3,5‐ di‐*tert*‐butyl‐phenyl)‐3,6‐di‐*tert*‐butyl‐carbazolyl) in our group, the question whether analogous silicon compounds were accessible as well, inevitably had to be asked. Initially the issue was tackled computationally (Figure 1). The computations (Gaussian16 B.01, PBE0 hybrid functional, def2SVP basis set) revealed that the chlorosilylene RSiCl is a stable molecule on the energy hypersurface (see Figure 1, SI 4.1). Then the energetics for the typical decomposition reactions, namely insertion of the Si atom in one of the C−C or C−H bonds of the flanking arene groups of the substituent, were investigated. For the C−C insertion reaction of RSiCl, the activation barrier was computationally determined to be 144.4 kJ mol^−1^ which might be thermally accessible, but the product (isomer **I**) is less stable than the chlorosilylene isomer by 25.7 kJ mol^−1^. On the other hand, the CH insertion product (isomer **II**) is thermodynamically favoured over the chlorosilylene RSiCl, but the activation barrier of 202.5 kJ mol^−1^ for this reaction is too high to be feasible. Similar energy profiles were found for the other base‐free halosilylenes RSiX (X=Cl, Br, I) and the silylenylium cation [RSi]^+^ (details see SI 4.1). With these data available, experiments were initiated.

**Figure 1:**
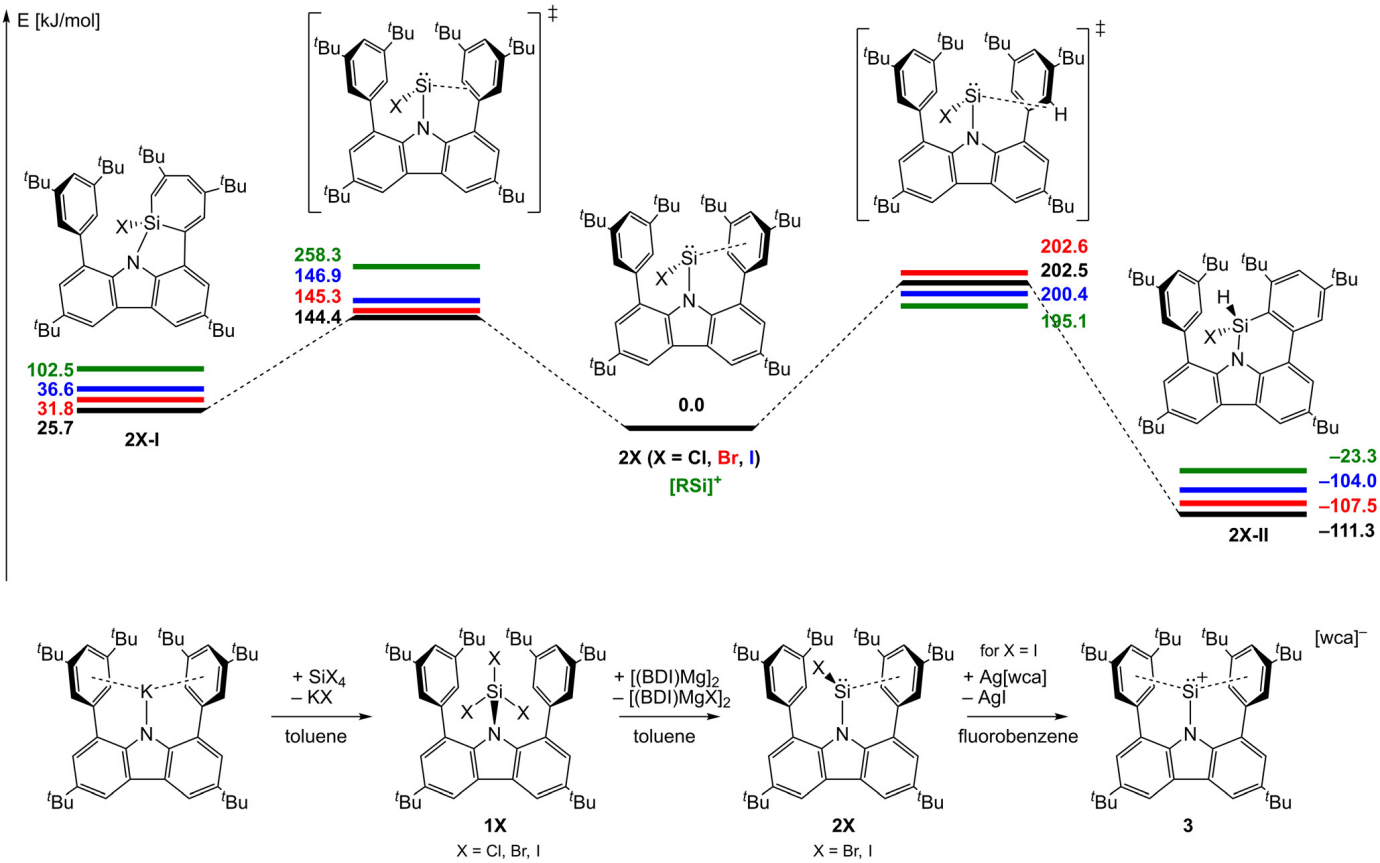
Top: Computed energy profile for carbazolyl‐silylene derivatives **2X** and [RSi]^+^ (*R*=1,8‐bis(3,5‐di‐*tert*‐butyl‐phenyl)‐3,6‐di‐*tert*‐butyl‐carbazole, energy not to scale). Bottom: Synthesis of base‐free halosilylenes RSiBr (**2Br**) and RSiI (**2I**, BDI=β‐diketiminate) and subsequent iodide abstraction from **2I**.

The metathesis reaction of potassium carbazolide (R‐K) with silicon halides SiX_4_ afforded the trihalosilane derivatives RSiX_3_ (**1X**, X=Cl, Br, I; Figure 1) in good to moderate yields (**1Cl** 77 %, **1Br** 71 %, **1I** 41 %). The molecular structure of these trihalosilane derivatives is surprising in the regard that the N−Si bond is bent out of the plane spanned by the carbazole C atoms by more than 40° (see SI 2.1–2.3), thereby reducing the effective steric bulk of the carbazolyl substituent drastically. The ^29^Si NMR resonances were found in the expected range for the respective trihalosilane (**1Cl** −23.5, **1Br** −59.5, **1I** −199.3 ppm). While all attempts of reduction of RSiCl_3_ to RSiCl failed, the heavier homologues RSiBr_3_ and RSiI_3_ could be reduced to afford the first base‐free halosilylenes, RSiBr (**2Br**) and RSiI (**2I**), where the best results were achieved with Mg^I^ compounds as reducing agents.^31^, ^32^ The reduction reactions were carried out by combining stoichiometric amounts of RSiX_3_ and [(^Mes^BDI)Mg]_2_ (BDI=β‐diketiminate, Mes=2,4,6‐trimethylphenyl) in toluene. After sonication at ambient temperature, the solvent was evaporated and the solid was extracted with *n*‐hexane to afford RSiX in moderate yields (**2Br** 50 %, **2I** 64 %). By removal of two substituents from the silicon atom the occupied space is reduced, hence the molecular structures show the silicon atom in the carbazole plane (Figure 2). The ^29^Si NMR resonances for **2Br** and **2I** were observed at +129.2 and +152.8 ppm, respectively, in the characteristic region for aminosilylenes. During all reduction reactions, an interesting silylene‐type by‐product was observed (*δ*(^29^Si)=+147.5 ppm). The compound could be isolated and identified as the bis‐carbazolyl silylene R_2_Si which is the bulkiest silylene known to date (see SI 2.5.1).

**Figure 2:**
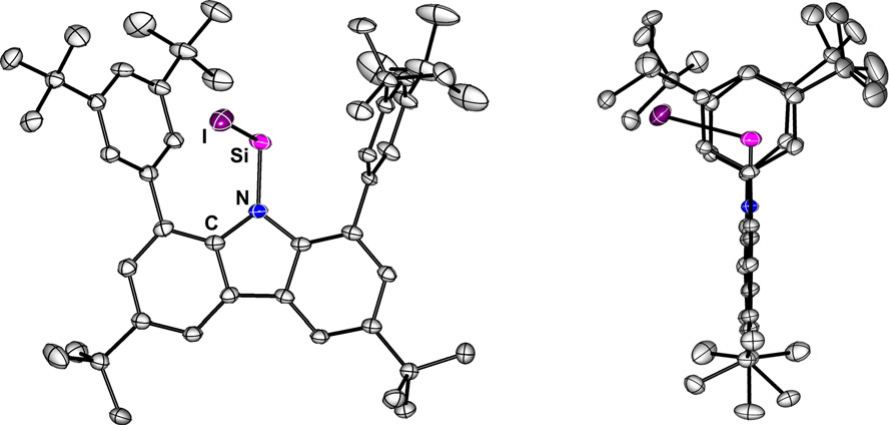
Molecular structure of RSiI (**2I**), thermal ellipsoids at 50 % probability. Selected bond lengths [Å]: Si‐I 2.5552(10); Si‐N 1.792(3); N‐Si‐I 103.26(9); angle sum at N 360°.

The halosilylenes **2Br** and **2I** could prove versatile starting materials for a wide range of reactions, but the focus of this study was set on halide abstraction reactions. Due to the high inherent reactivity of the silylenes and the reaction product, viable solvents for these reactions were only hydrocarbons and aromatic solvents. As a halide abstraction reagent, Ag^+^ salts were chosen, as neither Li^+^, Na^+^, K^+^ or Cs^+^ salts showed any reactivity in these solvents. Initial experiments of the reaction of **2Br** with Ag[wca] [wca=Al(OC_4_F_9_)_4_] yielded an intractable mixture of compounds in which no [RSi]^+^ was detected. However, the reaction of **2I** with Ag[wca] afforded [RSi][wca] (**3**) in 83 % yield as a yellow compound. In solution, the cation was characterized by multinuclear NMR spectroscopy in excellent agreement with the computationally predicted values for donor‐free [RSi]^+^ in the gas phase. The ^1^H NMR spectrum clearly indicates a *C*_2*v*_ symmetric compound as all CH_3_ groups of the arene‐*tert*‐butyl moieties are magnetically equivalent. In the ^13^C NMR spectrum, there is a characteristic shift of the resonance of the *meta*‐C atoms of the arenes to higher frequencies (164.26 ppm) which was also observed for the heavier homologuous [RE]^+^ species (E=Ge, Sn, Pb).^33^ The ^15^N NMR resonance (obs. +206.2 ppm, calc. +226 ppm) is significantly shifted compared to the starting material, RSiI (**2I**, +139.6 ppm, calc. +157 ppm). In contrast, ^29^Si NMR spectroscopy revealed that the Si atom is more shielded in **3** (+56.8 ppm, calc. +53 ppm) than in **2I** (+152.8 ppm, calc. +168 ppm). This shielding effect can be rationalized by considering the interaction with the flanking arenes: removal of one of the flanking arenes in the computed model compounds does not cause a significant shift in the ^29^Si NMR resonance (*δ*(^29^Si)=+75 ppm), but upon removal of both arenes, the resonance is predicted at *δ*(^29^Si)=+604 ppm (see SI 4.2). These values are in stark contrast to the value found for the penta‐coordinated [Cp*Si]^+^ (*δ*(^29^Si)=−400 ppm).^21^ The interaction of the Si atom with the arenes in **3** provides a stabilization energy of 181.2 kJ mol^−1^ (see SI 4.6). After many attempts, crystallisation by slow concentration of a solution of [RSi][wca] in fluorobenzene was successful, allowing the unambiguous identification of [RSi][wca] as a salt in the solid state (Figure 3). The structure of the cation shows a short N−Si bond (1.712(2) Å) which is contracted compared to both the Si^II^ species RSiI (1.792(3) Å) and even the Si^IV^ compound RSiI_3_ (1.770(2) Å). The three shortest Si‐C contacts to the flanking arenes in [RSi]^+^ are 2.654(2), 2.732(2) and 2.741(2) Å which is considerably longer than in Jutzi's [Cp*Si]^+^ (2.14 to 2.16 Å) and typical silylium‐arene contacts in arene adducts of [Et_3_Si]^+^ (2.18 Å) and [Me_3_Si]^+^ (2.12–2.18 Å).^3^, ^34^

**Figure 3:**
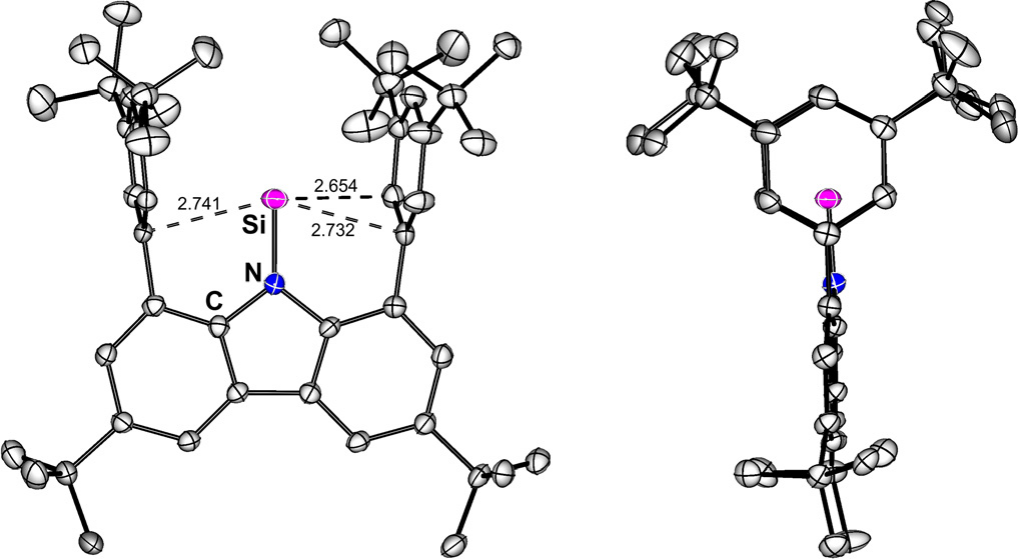
Molecular structure of [RSi][wca] (**3**, 130 K, shortest Si‐C contacts are indicated). Thermal ellipsoids at 50 % probability, H atoms and counterion omitted for clarity. Bond length Si‐N 1.714(2) Å, sum of angles at N: 359.5°.

The optical absorption of the silylenylium compound in fluorobenzene with a maximum at 406 nm (*ϵ*=2.33×10^3^ L mol^−1^ cm^−1^) is accurately reproduced by computations (obs. 406 nm, calc. 416 nm). As it is the lowest energy electronic transition, HOMO and LUMO are the major contributors for this electronic excitation (Figure 4). The HOMO of [RSi]^+^ is a mainly carbazole‐centred π‐type orbital with a small lobe extending to the Si‐N scaffold which indicates a small degree of double bond character with a strongly polarized π bond. Consequently, the LUMO is dominated by the π* contribution of the Si−N bond with a large contribution of the unoccupied p_x_ orbital of Si. The LUMO+1 involves the related in‐plane p_y_ orbital and the HOMO−3 shows significant contributions of the lone pair at the Si atom. This orbital depiction illustrates that [RSi]^+^ is an ambiphile where Lewis acidity is centred on the Si atom, while the basic functionality can be conveyed via the carbazole π scaffold or via the Si lone pair in a σ fashion.

**Figure 4:**
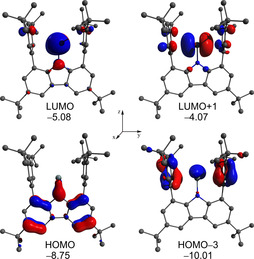
Selected Kohn–Sham orbital representations of [RSi]^+^ (isovalue 0.05, energies in eV).

With the silylenylium salt **3** available, the attention was then caught by options to achieve the envisaged three‐bond‐formation of the “supersilylene” with an amine.^19^ As the amine to study, ^*t*^BuNH_2_ was chosen and despite the steric shielding by the flanking arenes high reactivity of [RSi]^+^ is retained. The expected reaction pathway leads via an initially formed acid‐base adduct [RSi(NH_2_^*t*^Bu)]^+^ (**4**) to the isomeric silylium ion [RSi(H)NH^*t*^Bu]^+^ (**5**). However, when the experiment was conducted and studied by NMR spectroscopy, a spin system consistent with another product was observed which was assigned to the amine adduct of the silylium cation [RSi(H)(NH^*t*^Bu)NH_2_^*t*^Bu]^+^ (**6**). In good agreement with the computed value, the ^29^Si NMR resonance was observed at −29.4 ppm (calc. −36 ppm). The ^1^H NMR spectrum indicates the presence of two distinct N^*t*^Bu groups in proximity to the SiH moiety as well as the presence of an NH and an NH_2_ moiety (see SI 2.7). No intermediate was observed and no reaction to **5** occurred after addition of another equivalent of [RSi][wca] (**3**, Figure S44). The reaction was then studied in silico, confirming that for [RSi]^+^ the reaction with an amine in solution is exergonic and likely to proceed stepwise (Figure 5). The acid‐base adduct [RSi(NH_2_^*t*^Bu)]^+^ (**4**) should be formed initially (Δ*G*=−74.6 kJ mol^−1^) which then undergoes a proton shift reaction (Δ*G*=−42.8 kJ mol^−1^) to the isomeric silylium cation [RSi(H)NH^*t*^Bu)]^+^ (**5**). It should be noted that the monomolecular rearrangement reaction form [RSi(NH_2_^*t*^Bu)]^+^ (**4**) to [RSi(H)NH^*t*^Bu]^+^ (**5**) has a large activation barrier of 206.4 kJ mol^−1^ (computed with a reduced model for both the carbazole and the amine) which is thermally unattainable, so it is likely to proceed with additional molecules of the amine as proton shuttle. Then, the adduct formation with another molecule ^*t*^BuNH_2_ to give **6** (Δ*G*=−113.5 kJ mol^−1^) is more exergonic than the first step forming **4** (see SI 4.4). This indicates a fascinating feature of the silylenylium cation [RSi]^+^, as for this strong Lewis acid, oxidative addition of the NH bond of a substrate molecule yields a silylium cation which is an even stronger Lewis acid. This is corroborated by the computed affinities for fluoride ([RSi]^+^ 864.8, **5** 901.4 kJ mol^−1^) and hydride ions ([RSi]^+^ 759.0, **5** 822.0 kJ mol^−1^) which are lower for the silylenylium ion [RSi]^+^ than derived silylium ion **5** (see SI 4.3). Also, the computed charges of the silicon atom indicate this tendency, as in [RSi(H)NH^*t*^Bu]^+^ the silicon atom bears a considerably more positive charge than in [RSi]^+^ (**5** 1.83*e*; [RSi]^+^ +1.18*e*; cf. [Cp*Si]^+^ +0.99*e*). The reaction of silylenylium cations with suitable substrates such as amines enables a new route to H‐substituted functionalized silylium cations, the first isolated examples of which have only been reported very recently by Oestreich.^35^

**Figure 5:**
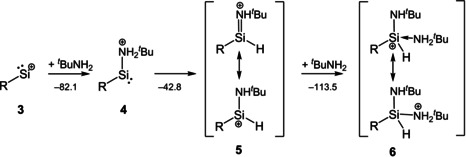
Computed free reaction enthalpies in kJ mol^−1^ for the reactions of [RSi]^+^ with ^*t*^BuNH_2_ (R=bulky carbazolyl substituent, counterion [wca]^−^ omitted).

In conclusion, the synthesis of the mono‐coordinated silylenylium cation [RSi]^+^ by halide abstraction from the first donor‐free halosilylenes is presented. Stabilized by a carbazole scaffold and arene interactions, the cation bears no σ‐donors except for the lone substituent. The predicted reactivity for this type of cation leading to the formation of three bonds at the silicon atom could be demonstrated by a reaction with an amine, enabling a new route to silylium cations bearing an H substituent and a functional group.

Deposition numbers 2010372 (**1Cl**), 2010373 (**1Br**), 2010374 (**1I**), 2010375 (**2Br**), 2010376 (**2I**), 2010377 (**3**), 2010378 (**R_2_Si**) and 2010379 (**2I‐II**) contain the supplementary crystallographic data for this paper. These data are provided free of charge by the joint Cambridge Crystallographic Data Centre and Fachinformationszentrum Karlsruhe Access Structures service.

## Conflict of interest

The authors declare no conflict of interest.

## Supporting information

As a service to our authors and readers, this journal provides supporting information supplied by the authors. Such materials are peer reviewed and may be re‐organized for online delivery, but are not copy‐edited or typeset. Technical support issues arising from supporting information (other than missing files) should be addressed to the authors.

Supplementary

Supplementary

Supplementary

Supplementary

Supplementary

Supplementary
