# Supplementary material for: A Mono‐Substituted Silicon(II) Cation: A Crystalline “Supersilylene”
Source: Angew Chem Int Ed Engl. 2020 Aug 26;59(43):19065–9. doi: 10.1002/anie.202009874 (PMC7590127; doi:10.1002/anie.202009874)
Supplement: Supplementary file 1 — Supplementary [file ANIE-59-19065-s001.pdf]

Angewandte Chemie
Eine Zeitschrift der Gesellschaft Deutscher Chemiker

Supporting Information

A Mono-Substituted Silicon(II) Cation: A Crystalline “Supersilylene”

*Alexander Hinz**

anie_202009874_sm_miscellaneous_information.pdf
anie_202009874_sm_Si_1.tif
anie_202009874_sm_Si_2.tif
anie_202009874_sm_Si_3.tif
anie_202009874_sm_Si_4.tif
anie_202009874_sm_Si_5.tif

Table of Contents

1 General Considerations 3
2 Syntheses 4
2.1 $RSiCl_3$ (**1Cl**) 4
2.2 $RSiBr_3$ (**1Br**) 7
2.3 $RSiI_3$ (**1I**) 10
2.4 RSiBr (**2Br**) 13
2.5 RSiI (**2I**) 16
2.5.1 R_2Si 19
2.5.2 RSiI-II (**2I-II**) 21
2.6 $[RSi][Al(OC_4F_9)_4]$ (**3**) 25
2.7 $[RSi(H)(NH^tBu)NH_2{}^tBu][Al(OC_4F_9)_4]$ (**6**) 28
3 Crystallographic Details 31
4 Computational Details 34
4.1 Isomerisation of **2X** and $[RSi]^+$ 35
4.2 Influence of arene on ^{29}Si NMR of $[RSi]^+$ 36
4.3 F^-/H^- affinities 37
4.4 Reaction pathway of **3** with $^tBuNH_2$ 38
4.5 Orbital depictions of $[RSi]^+$ 39
4.6 Isodesmic Reactions 40
4.7 Optimized structures 41
4.7.1 $RSiCl_3$ 41
4.7.2 $RSiBr_3$ 43
4.7.3 $RSiI_3$ 45
4.7.4 RSiCl 47
4.7.5 RSiCl-I 49
4.7.6 RSiCl-I-TS 51
4.7.7 RSiCl-II 54
4.7.8 RSiCl-II-TS 56
4.7.9 RSiBr 58
4.7.10 RSiBr-I 60
4.7.11 RSiBr-I-TS 62
4.7.12 RSiBr-II 64

4.7.13 RSiBr-II-TS 66
4.7.14 RSiI 69
4.7.15 RSiI-I 71
4.7.16 RSiI-I-TS 73
4.7.17 RSiI-II 75
4.7.18 RSiI-II-TS 77
4.7.19 RSi^+ 79
4.7.20 RSi^+-I 81
4.7.21 RSi^+-I-TS 84
4.7.22 RSi^+-II 86
4.7.23 RSi^+-II-TS 88
4.7.24 RSi^+, one arene 90
4.7.25 RSi^+, no arene 91
4.7.26 $RSi(NH_2{}^tBu)^+$ 92
4.7.27 $RSi(H)NH^tBu^+$ 95
4.7.28 $RSi(H)(NH^tBu)NH_2{}^tBu^+$ 97
4.7.29 model $R^MSi(NH_3)^+$ 100
4.7.30 model $R^MSi(NH_3)^+$ TS 102
4.7.31 model $R^MSi(H)NH_2{}^+$ 103
4.7.32 $[RSi(H)NH_2]^+ + F^-$ 105
4.7.33 $[RSi(H)NH_2]^+ + H^-$ 107
4.7.34 $RSiI + F^-$ 110
4.7.35 $RSiI + H^-$ 112
4.7.36 $RSi^+ + F^-$ 114
4.7.37 $RSi^+ + H^-$ 116
4.7.38 Cp^*Si^+ 118
4.7.39 $Cp^*Si^+ + F^-$ 119
4.7.40 $Cp^*Si^+ + H^-$ 119
4.7.41 $[RSi(H)NH^tBu]^+ + F^-$ 120
4.7.42 $[RSi(H)NH^tBu]^+ + H^-$ 122
4.7.43 R_2Si 125
5 References 130

1 General Considerations

Et_2O (Sigma-Aldrich), THF (Roth, 99%), n-hexane (Roth, 98%) and toluene (Roth, 99%) were dried over sodium and distilled prior to use. Fluorobenzene and dichlorobenzene were degassed and dried over 3 Å molecular sieves. C_6D_6 (Eurisotop) was dried over sodium and distilled prior to use. $SiCl_4$ (abcr, 99%), $SiBr_4$ (abcr, 99%) and SiI_4 (abcr, 99%) were used as received.

Starting materials were prepared according to literature protocols: Potassium carbazolide (RK)[33] and $Ag[Al(OC_4F_9)_4]$[36] were prepared according to established procedures.

NMR spectra were acquired on a Bruker Avance 400 MHz spectrometer. Reported chemical shifts are referenced to the ^{1}H and ^{13}C NMR resonances of the deuterated solvent.[37] Coupling constants *J* are given in Hertz as positive values regardless of their real individual sign. ^{1}H, ^{11}B, ^{13}C, ^{15}N, ^{19}F, ^{29}Si NMR spectra were obtained at 400.1, 128.4, 100.6, 40.6, 376.5, 79.5 MHz, respectively.

IR spectra were recorded on a Bruker Alpha spectrometer using the attenuated total reflection (ATR) technique on powdered samples.

Elemental analyses were obtained with a Vario Micro Cube (Elementar Analysensysteme GmbH) in the institutional technical laboratories of the Karlsruhe Institute of Technology (KIT).

Single crystals were mounted in perfluoropolyalkyl ether oil on a cryo loop and then brought into the cold nitrogen stream of a low-temperature device (Oxford Cryosystems Cryostream unit) so that the oil solidified. Diffraction data were collected using a Stoe IPDS II diffractometer and graphite-monochromated Mo-Kα (0.71073 Å) radiation or a Stoe STADIVARI diffractometer and Ga-Kα (1.34134 Å) radiation. The structures were solved by direct methods with SHELXS[38] or intrinsic phasing with SHELXT[39] followed by full-matrix least-squares refinement using SHELXL-2018/3[40] and the ShelXle GUI.[41] All non-hydrogen atoms were refined anisotropically. The contribution of the hydrogen atoms, in their calculated positions, was included in the refinement using a riding model.

2 Syntheses

2.1 $RSiCl_3$ (**1Cl**)

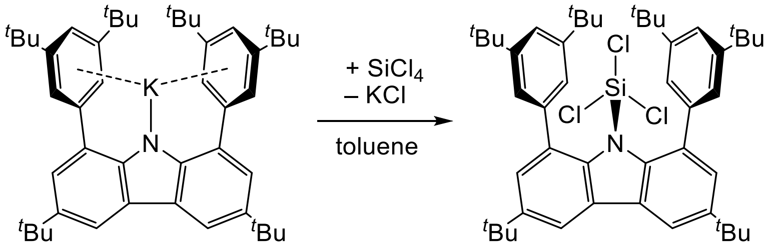

To a solution of 0.2 ml $SiCl_4$ (approx. 300 mg, 1.76 mmol) in 3 ml toluene a solution of 480 mg (0.692 mmol) RK in 10 ml toluene was added dropwise at ambient temperature. The yellow mixture was then heated to 70 °C and stirred overnight. Then, all volatiles were removed in vacuo, affording a pale yellow residue. The residue was extracted with 10 ml of toluene and filtered. The filtrate was concentrated to incipient crystallisation in an oil bath at 50 °C (approx. 2 ml) and then left to cool down overnight, affording colourless crystals. The supernatant was discarded and the crystals were dried in vacuo (418 mg, 0.529 mmol, 77%).

^{1}H NMR (C_6D_6): 1.37 (s, 18 H, Carb-^{t}Bu), 1.39 (s, 36 H, Ar-^{t}Bu), 7.60 (t, J_{HH} = 1.8 Hz, 2 H, $C^{2,7}H$), 7.64 (d, J_{HH} = 2.1 Hz, 2 H, *p*-C*H*), 7.73 (d, J_{HH} = 1.5 Hz, $\nu_{1/2}$ = 2.0 Hz, 4 H, *o*-C*H*), 8.18 (d, J_{HH} = 2.1 Hz, 2 H, $C^{4,5}H$). **^{13}C NMR** (C_6D_6): 31.85 (s, carb-C(CH$_3$)$_3$), 31.82 (s, Ar-C(CH$_3$)$_3$), 34.79 (s, carb-C(CH$_3$)$_3$), 35.11 (s, Ar-C(CH$_3$)$_3$), 115.68 (s, *C*H), 122.37 (s, *C*H), 125.33 (br s, $\nu_{1/2}$ = 25.2 Hz, *C*H), 128.81 (s, *C*H), 131.17 (s), 133.77 (s), 141.57 (s), 142.43 (s), 147.32 (s), 150.91 (s, *m*-*C*). **^{15}N NMR** (C_6D_6): 111.8 (s). **^{29}Si NMR** (C_6D_6): –23.5 (s). **EA** found (calc. for $C_{48}H_{64}NSiCl_3$): C 73.95 (73.03), H 8.42 (8.17), N 1.93 (1.77).

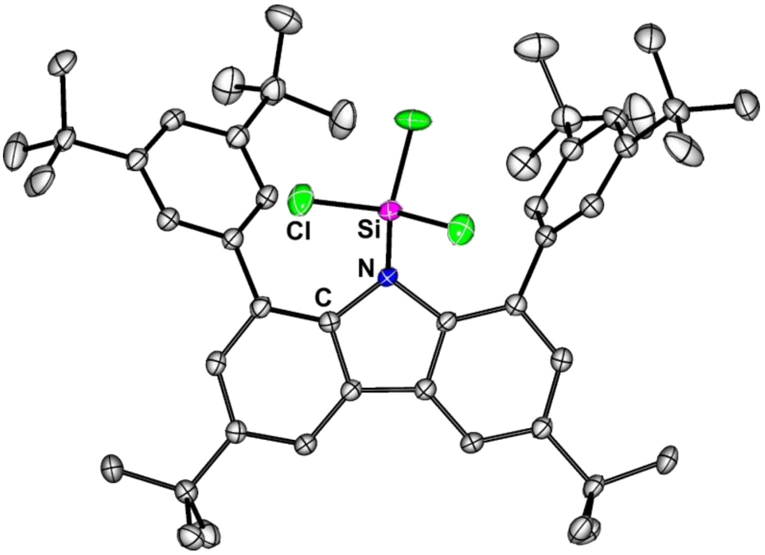

Figure S1: Molecular structure of **1Cl**.

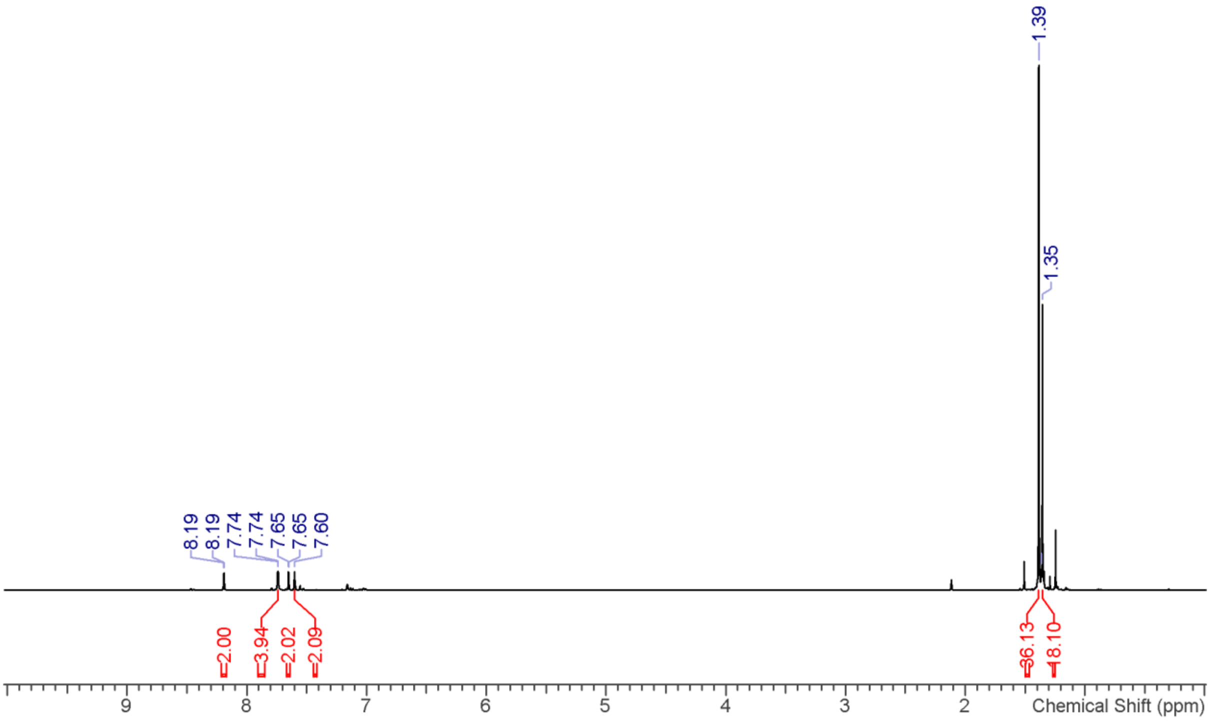

Figure S2: ^{1}H NMR spectrum of **1Cl**.

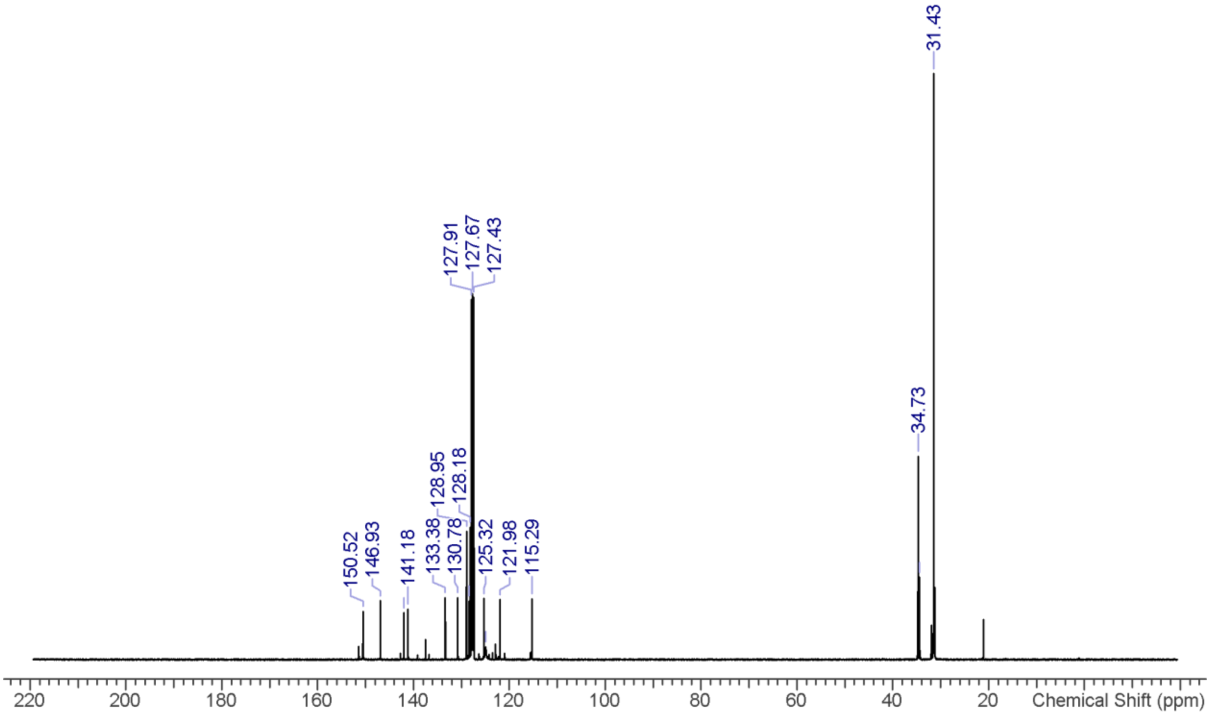

Figure S3: $^{13}C\{^{1}H\}$ NMR spectrum of **1Cl**.

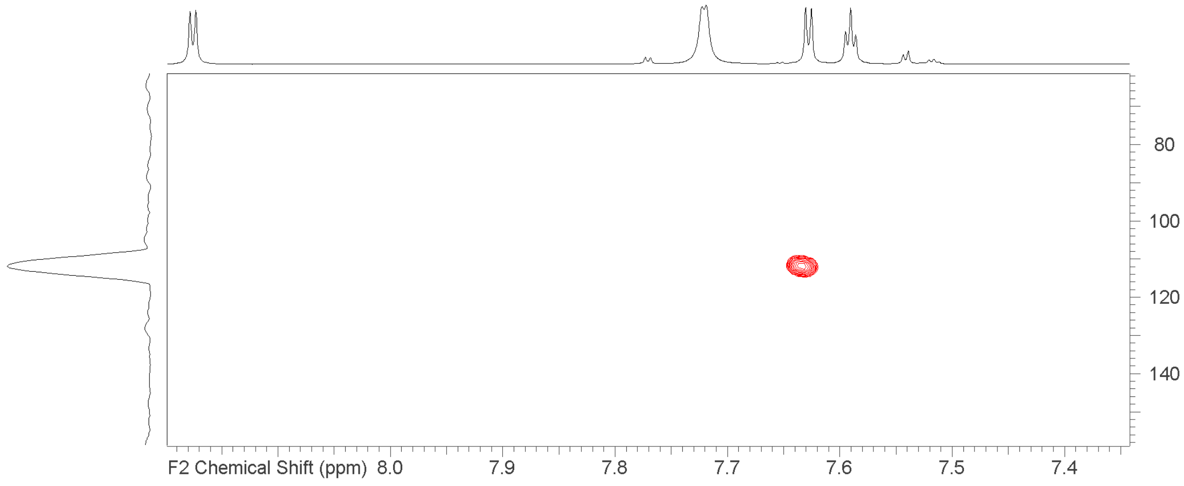

Figure S4: ^{1}H-^{15}N HMBC NMR spectrum of **1Cl**.

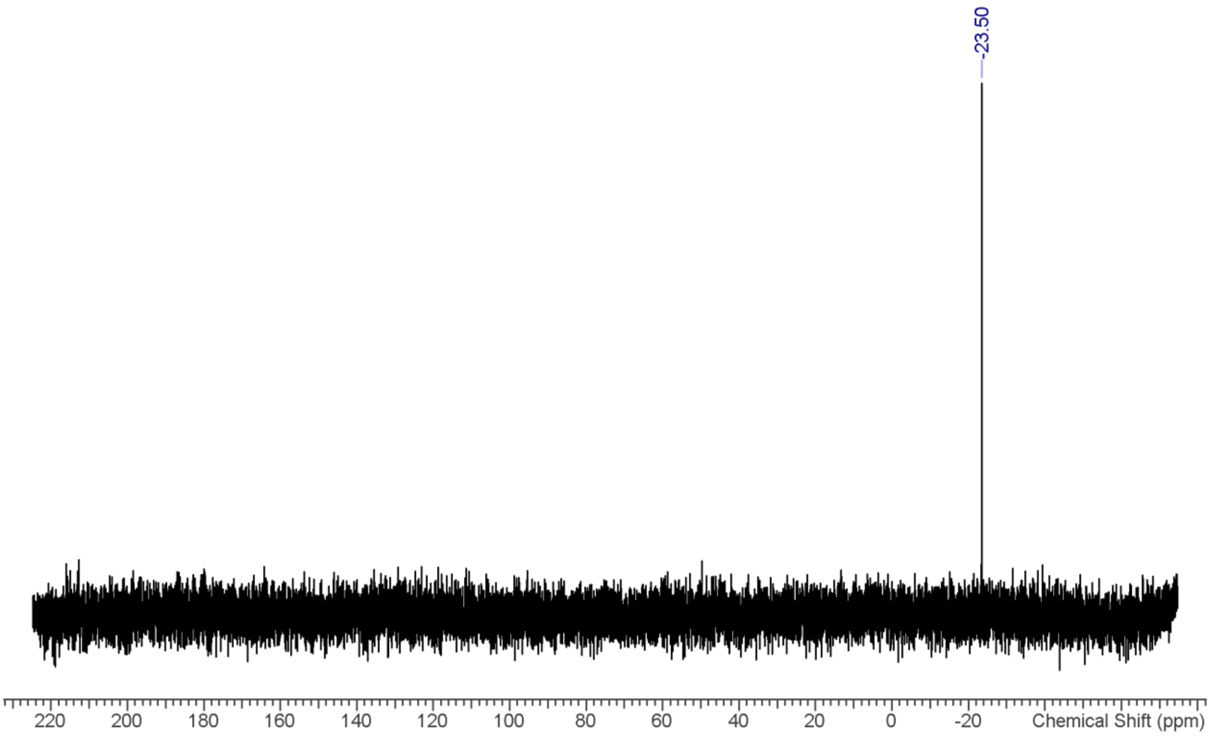

Figure S5: $^{29}Si\{^{1}H\}$ NMR spectrum of **1Cl**.

2.2 $RSiBr_3$ (**1Br**)

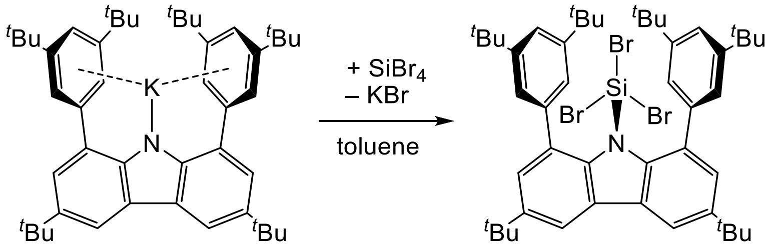

To a solution of 375 mg $SiBr_4$ (1.08 mmol) in 3 ml toluene a solution of 680 mg (0.980 mmol) RK in 15 ml toluene was added dropwise at ambient temperature. The yellow mixture was then heated to 60 °C and stirred overnight. Then, all volatiles were removed in vacuo, affording a pale yellow residue. The residue was extracted with 15 ml of toluene and filtered. The filtrate was concentrated to incipient crystallisation in an oil bath at 50 °C (approx. 3 ml) and then left to cool down overnight, affording pale yellow crystals. The supernatant was discarded and the crystals were dried in vacuo (642 mg, 0.696 mmol, 71%).

^{1}H NMR (C_6D_6): 1.35 (s, 18 H, Carb-^{t}Bu), 1.40 (s, 36 H, Ar-^{t}Bu), 7.60 (t, J_{HH} = 1.8 Hz, 2 H, $C^{2,7}H$), 7.64 (d, J_{HH} = 2.0 Hz, 2 H, *p*-C*H*), 7.78 (d, J_{HH} = 1.6 Hz, 4 H, *o*-C*H*), 8.15 (d, J_{HH} = 2.1 Hz, 2 H, $C^{4,5}H$). **^{13}C NMR** (C_6D_6): 31.78 (s, carb-C(CH_3)$_3$), 31.84 (s, Ar-C(CH_3)$_3$), 34.77 (s, carb-*C*(CH_3)$_3$), 35.11 (s, Ar-*C*(CH_3)$_3$), 115.75 (s, *C*H), 122.48 (s, *C*H), 123.26 (s, *C*H), 125.79 (br s, *C*H), 128.82 (s, *C*H), 131.55 (s), 134.42 (s), 141.82 (s), 143.34 (s), 147.69 (s), 150.84 (s). **^{15}N NMR** (C_6D_6): 113.2 (s). **^{29}Si NMR** (C_6D_6): –59.5 (s). **EA** found (calc. for $C_{48}H_{64}NSiBr_3$): C 62.88 (62.47), H 6.81 (6.99), N 1.68 (1.52).

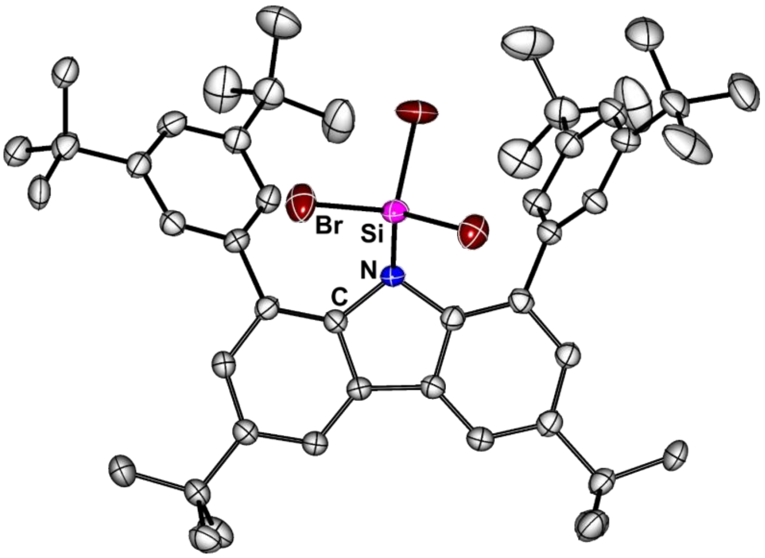

Figure S6: Molecular structure of **1Br**.

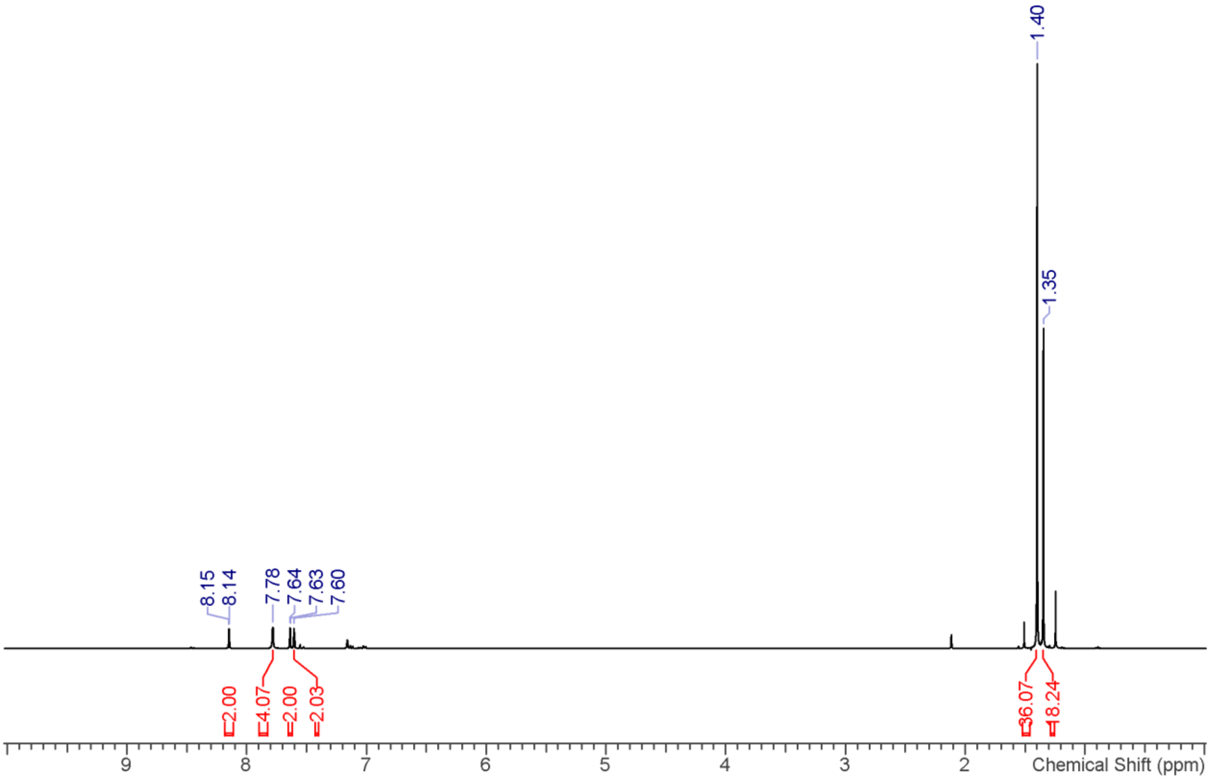

Figure S7: ^{1}H NMR spectrum of **1Br**.

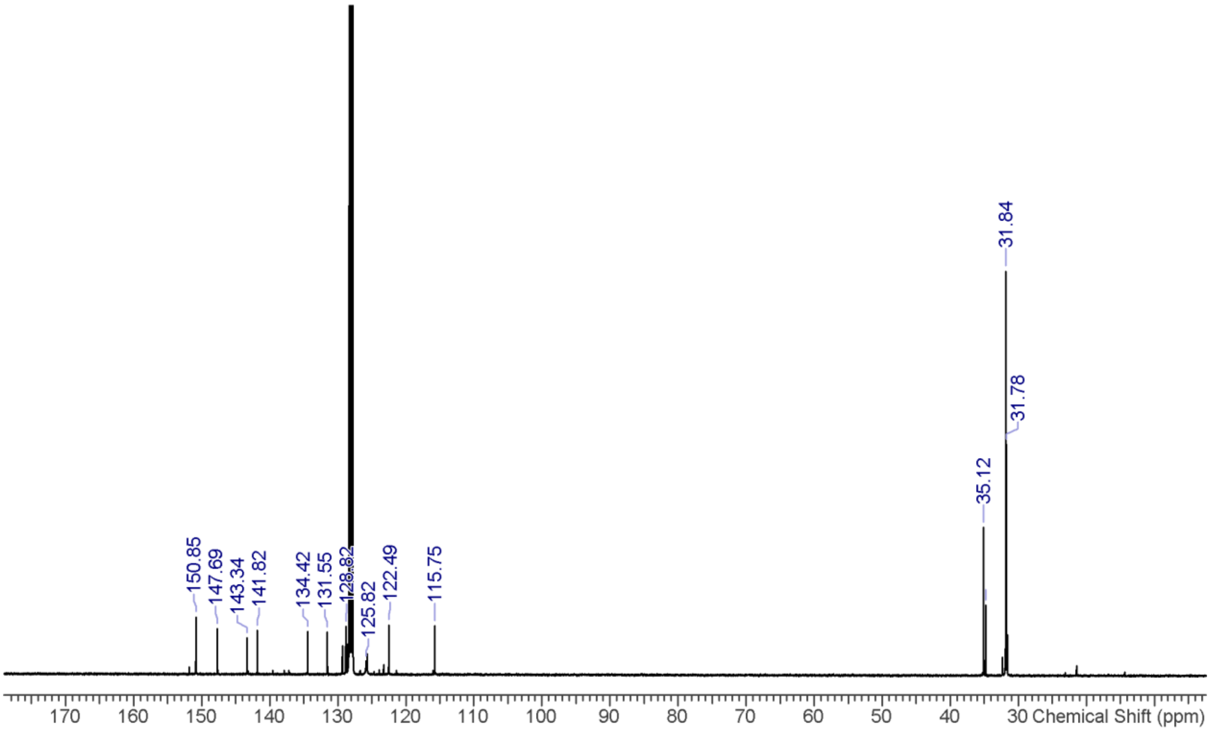

Figure S8: $^{13}C\{^{1}H\}$ NMR spectrum of **1Br**.

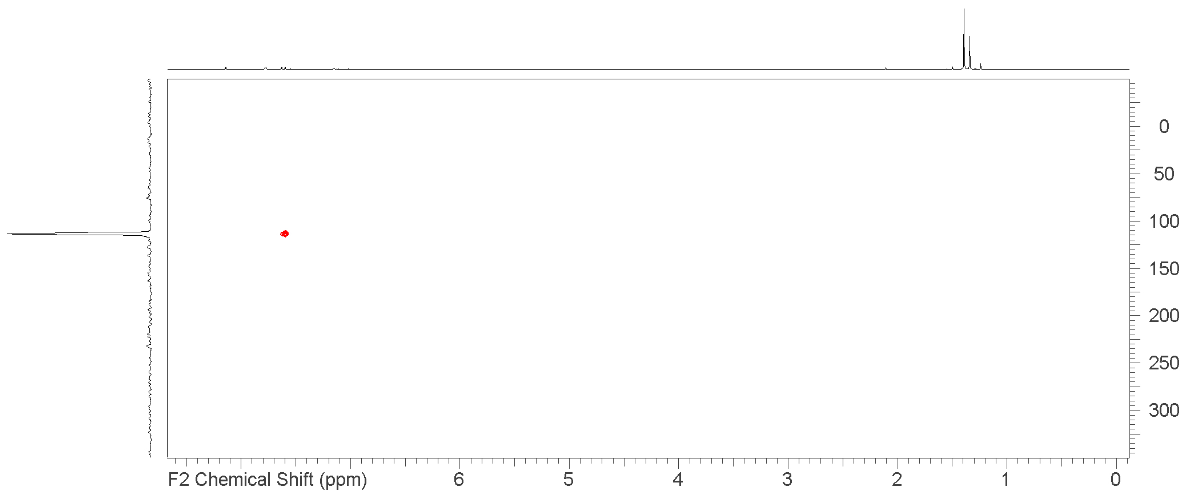

Figure S9: ^{1}H-^{15}N HMBC NMR spectrum of **1Br**.

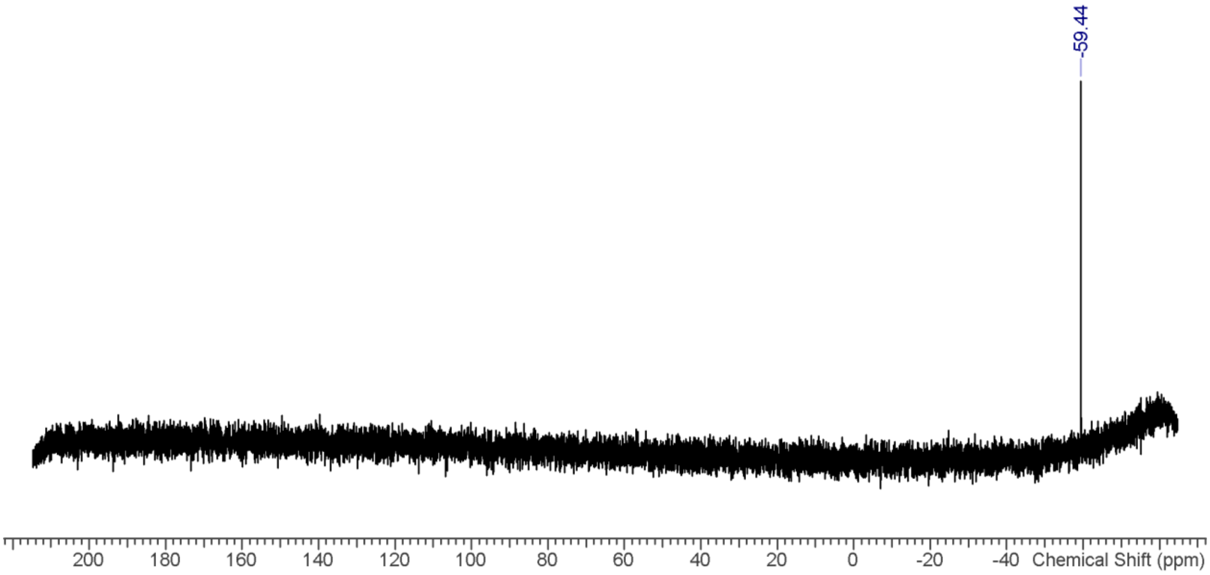

Figure S10: ^{29}Si{^{1}H} NMR spectrum of **1Br**.

2.3 $RSiI_3$ (**1I**)

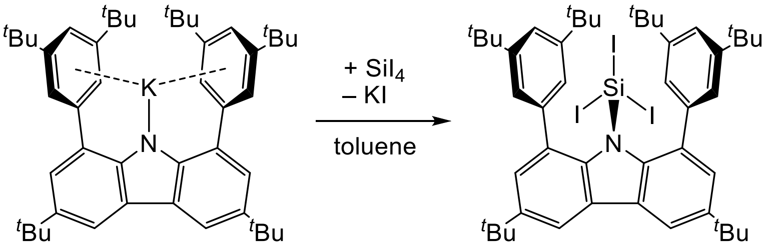

A solution of RK (2.05 g, 2.95 mmol) in toluene was added to a suspension of SiI_4 (2.06 g, 3.84 mmol) in toluene. The mixture was sonicated for 30 minutes, turning dark yellow, and then stirred for one day at 40 °C, affording a yellow suspension. The mixture was filtered through a sintered disk (G4). All volatiles of the filtrate were removed in vacuo. The yellow residue was then washed twice with 20 ml hexane, allowing the isolation of $RSiI_3$ as a pale yellow solid. Recrystallisation from toluene at ambient temperature afforded single crystals suitable for structure elucidation (41%).

^{1}H NMR (C_6D_6): 1.34 (s, 18 H, carb-^{t}Bu), 1.43 (s, 36 H, carb-^{t}Bu), 7.62 (m, 4 H, *p*-C*H*/C^{2,7}*H*), 7.86 (d, 4 H, J_{HH} = 1.3 Hz, *o*-C*H*), 8.09 (d, 2 H, J_{HH} = 2.1 Hz, C^{4,5}*H*). **^{13}C NMR** (C_6D_6): 31.75 (s, carb-C(CH$_3$)$_3$), 31.99 (s, Ar-C(CH$_3$)$_3$), 34.80 (s, carb-C(CH$_3$)$_3$), 35.17 (s, Ar-C(CH$_3$)$_3$), 115.85 (s, CH), 122.80 (s, CH), 126.48 (s, $\nu_{1/2}$ = 15.6 Hz, *C*H), 128.86 (s, CH), 131.94 (s), 135.09 (s), 142.03 (s), 145.41 (s), 148.04 (s), 150.84 (s). **^{15}N NMR** (C_6D_6): +109.1 (s). **^{29}Si NMR** (C_6D_6): –199.3 (s). **EA** found (calc. for $C_{48}H_{64}NSiI_3$): C 53.91 (54.19), H 5.73 (6.06), N 1.37 (1.32).

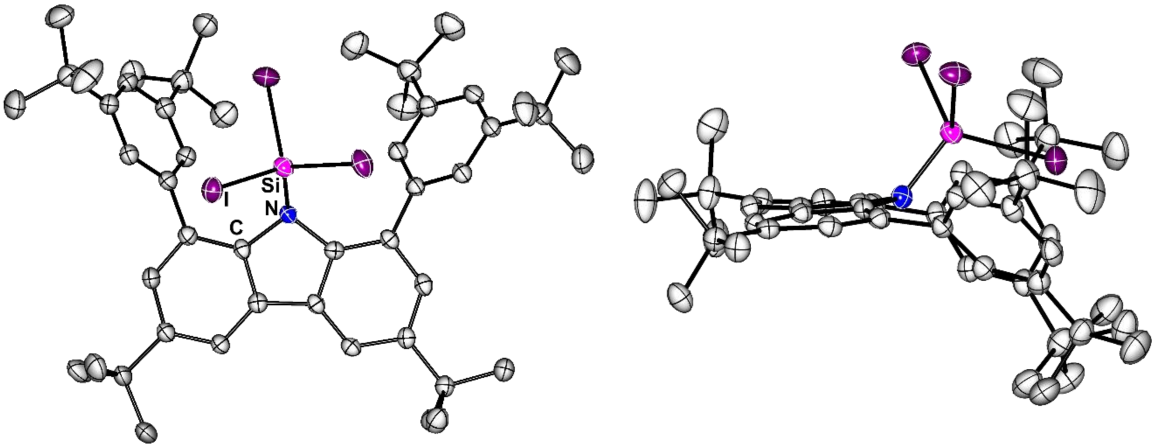

Figure S11: Molecular structure of **1I**. I1–Si1 2.4555(8); I2–Si1 2.4262(8); I3–Si1 2.4211(8); Si1–N1 1.770(2); C1–N1–C12 104.6(2); C1–N1–Si1 115.6(2); C12–N1–Si1 120.8(2); angle sum at N 341°.

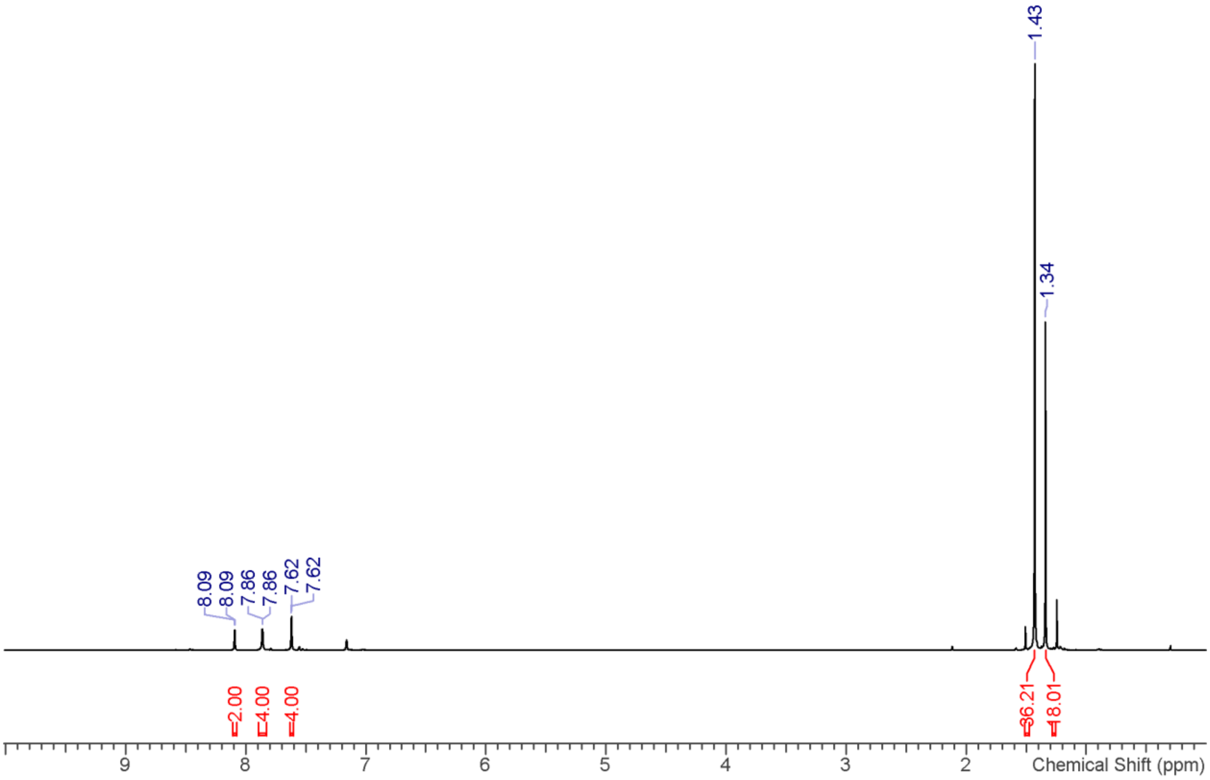

Figure S12: ^{1}H NMR spectrum of **1l**.

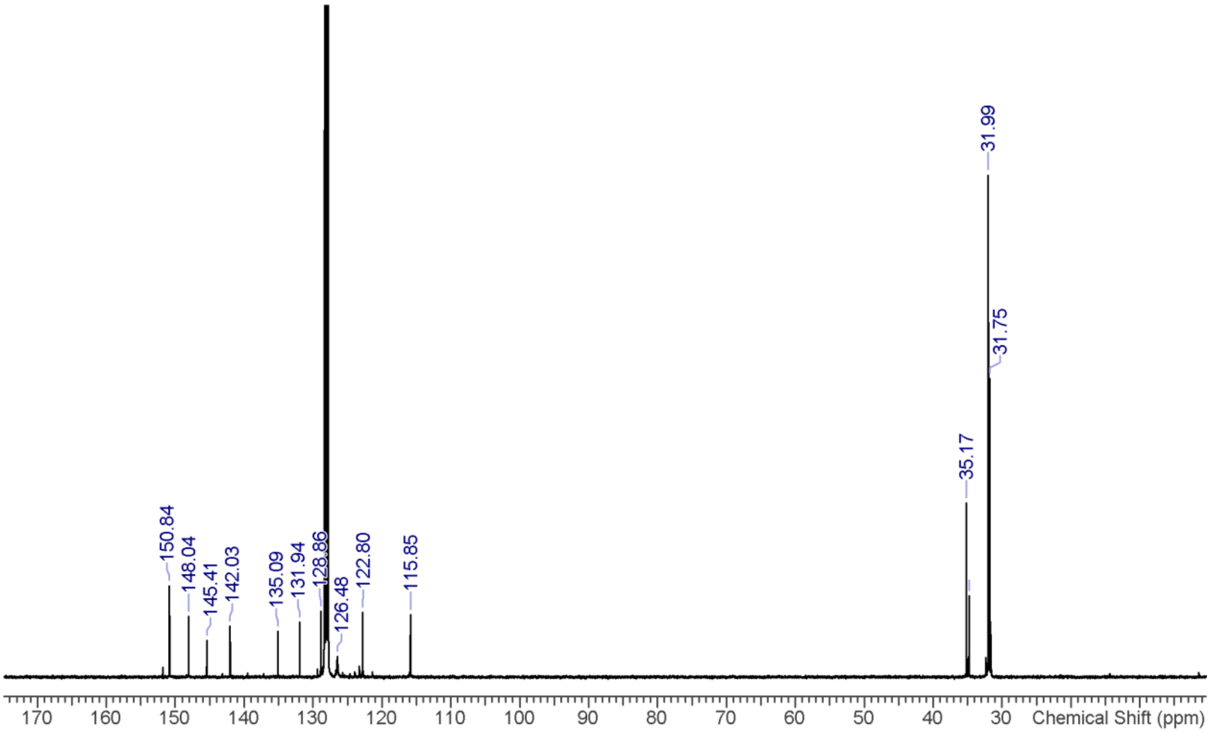

Figure S13: $^{13}C\{^{1}H\}$ NMR spectrum of **1l**.

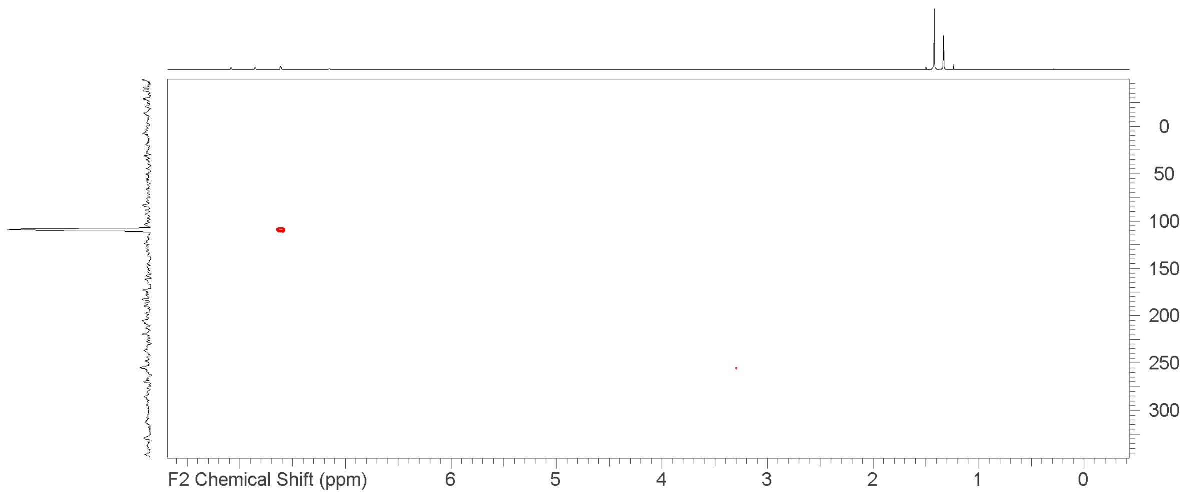

Figure S14: ^{1}H-^{15}N HMBC NMR spectrum of **1l**.

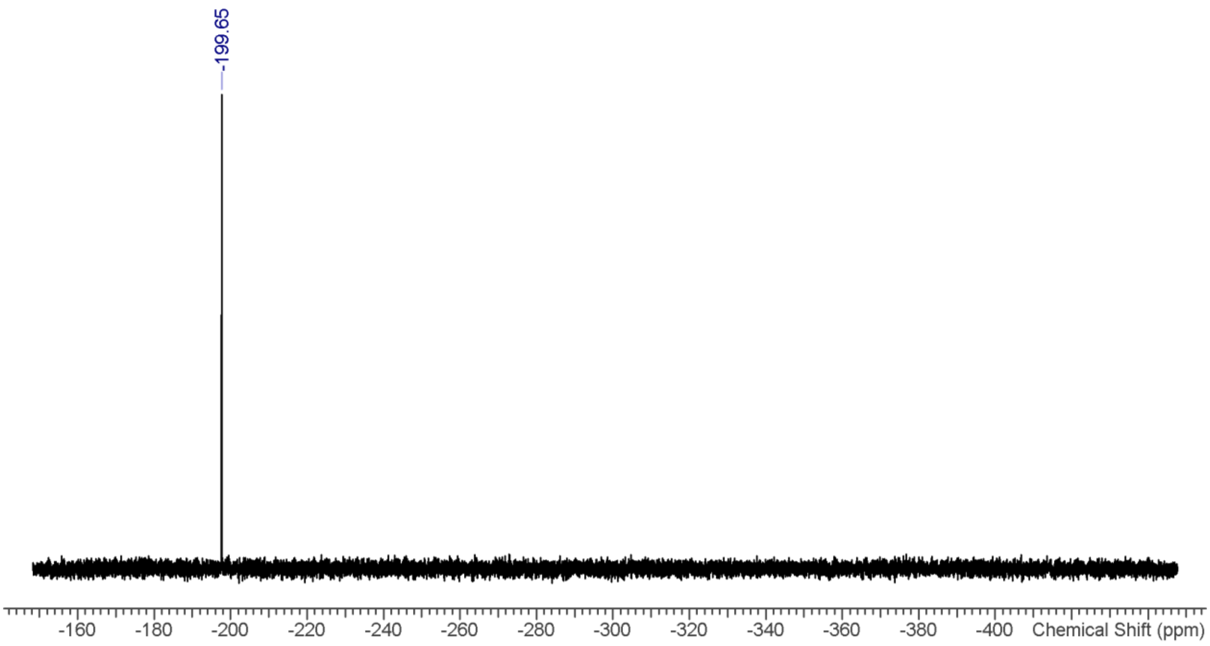

Figure S15: $^{29}Si\{^{1}H\}$ NMR spectrum of **1l**.

2.4 RSiBr (**2Br**)

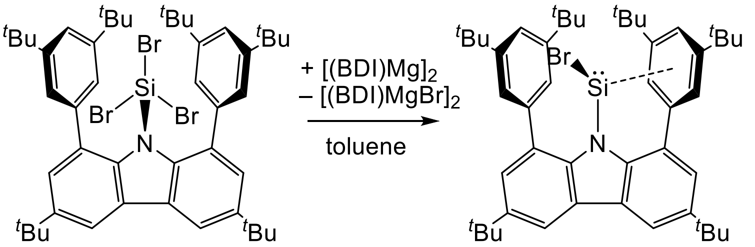

$RSiBr_3$ (1250 mg, 1.35 mmol) and [(^{Mes}BDI)Mg]$_2$ (998 mg, 1.39 mmol) were combined as solids. To the mixture 40 ml of toluene were added. The mixture was sonicated for 1 hour and then stirred at 35 °C overnight. Afterwards, volatiles were removed in vacuo. The mixture was then extracted with 50 + 10 ml of hexane. The solution was concentrated to approx. 5 ml and left undisturbed overnight, resulting in the deposition of yellow crystals RSiBr. The supernatant was removed via syringe and the solid was dried in vacuo (516 mg, 0.81 mmol, 50%).

^{1}H NMR (C_6D_6): 1.27 (s, 18 H, carb-^{t}Bu), 1.44 (s, 36 H, Ar-^{t}Bu), 7.53 (s, $\nu_{1/2}$ = 7.1 Hz, 4 H, *o*-C*H*), 7.56 (t, J_{HH} = 1.7 Hz, 2 H, *p*-C*H*), 7.63 (d, J_{HH} = 2.1 Hz, 2 H, $C^{2,7}$*H*), 8.41 (d, J_{HH} = 2.1 Hz, 2 H, $C^{4,5}$*H*). **^{13}C NMR** (C_6D_6): 31.40 (s, $\nu_{1/2}$ = 4.6 Hz, Ar-C(CH$_3$)$_3$), 32.21 (s, Carb-C(CH$_3$)$_3$), 34.95 (s, Carb-*C*(CH$_3$)$_3$), 35.45 (s, Carb-*C*(CH$_3$)$_3$), 116.24 (s, *C*H), 123.64 (s, *C*H), 124.93 (s, *C*H), 127.21 (s), 127.82 (s), 128.06 (s), 128.18 (s), 128.30 (s), 144.09 (s), 144.53 (s), 145.34 (s), 155.56 (s, $\nu_{1/2}$ = 50 Hz, *m-C*). **^{15}N NMR** (C_6D_6): 145.4 (s). **^{29}Si NMR** (C_6D_6): 129.2 (s). **EA** found (calc. for $C_{48}H_{64}NSiBr$): C 75.17 (75.56), C 8.56 (8.45), C 2.05 (1.84). **EA** found (calc. for $C_{48}H_{64}NSiBr$): C 75.01 (75.56), H 8.30 (8.45), N 2.02 (1.84).

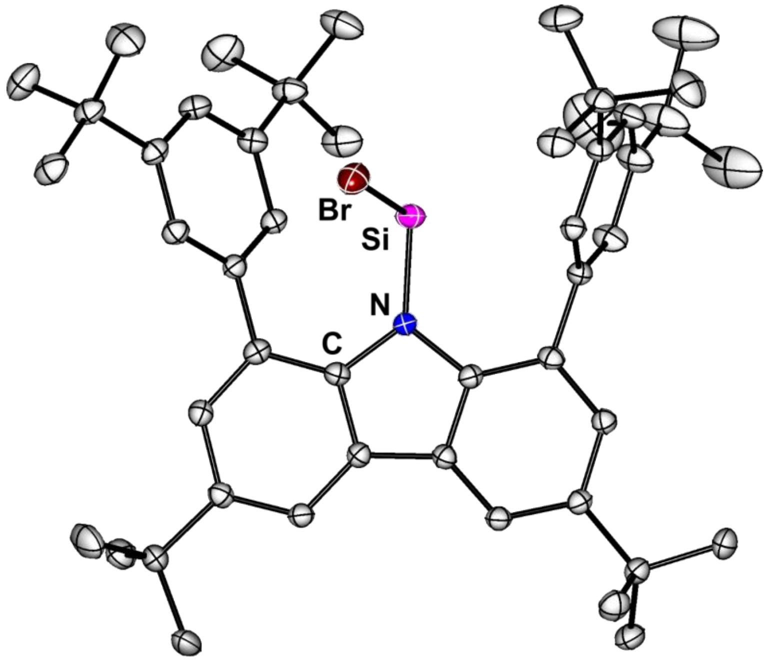

Figure S16: Molecular structure of **2Br**.

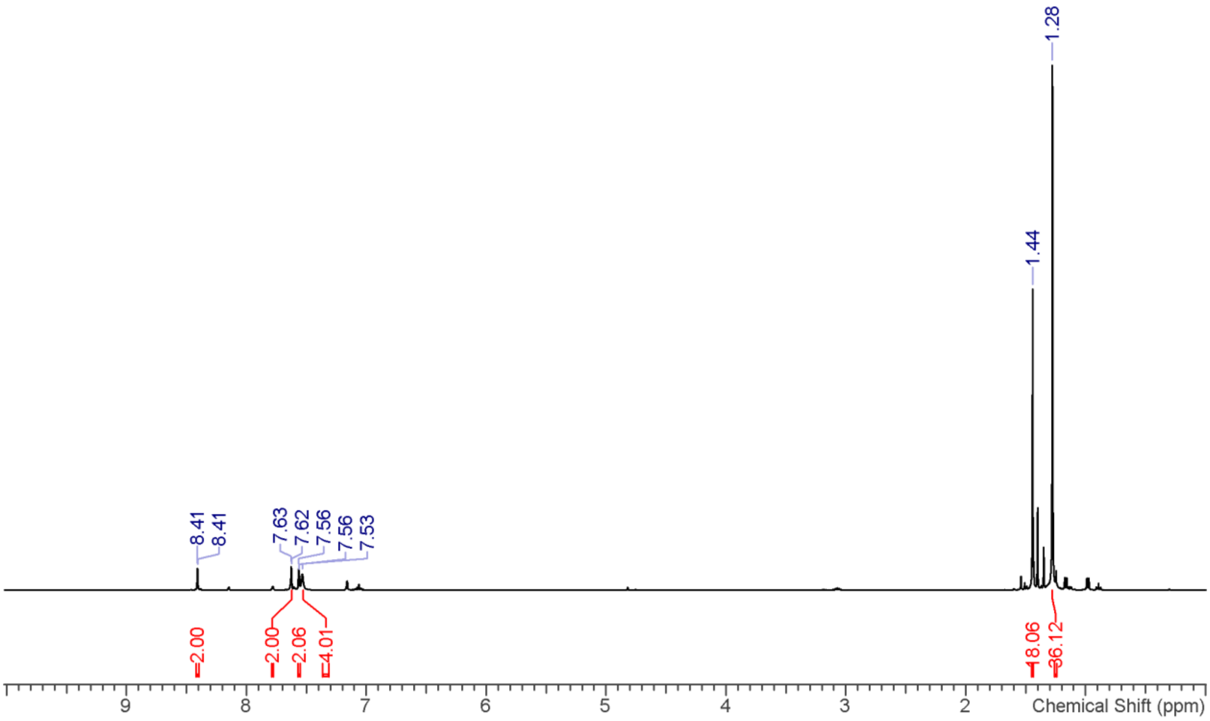

Figure S17: ^{1}H NMR spectrum of **2Br**.

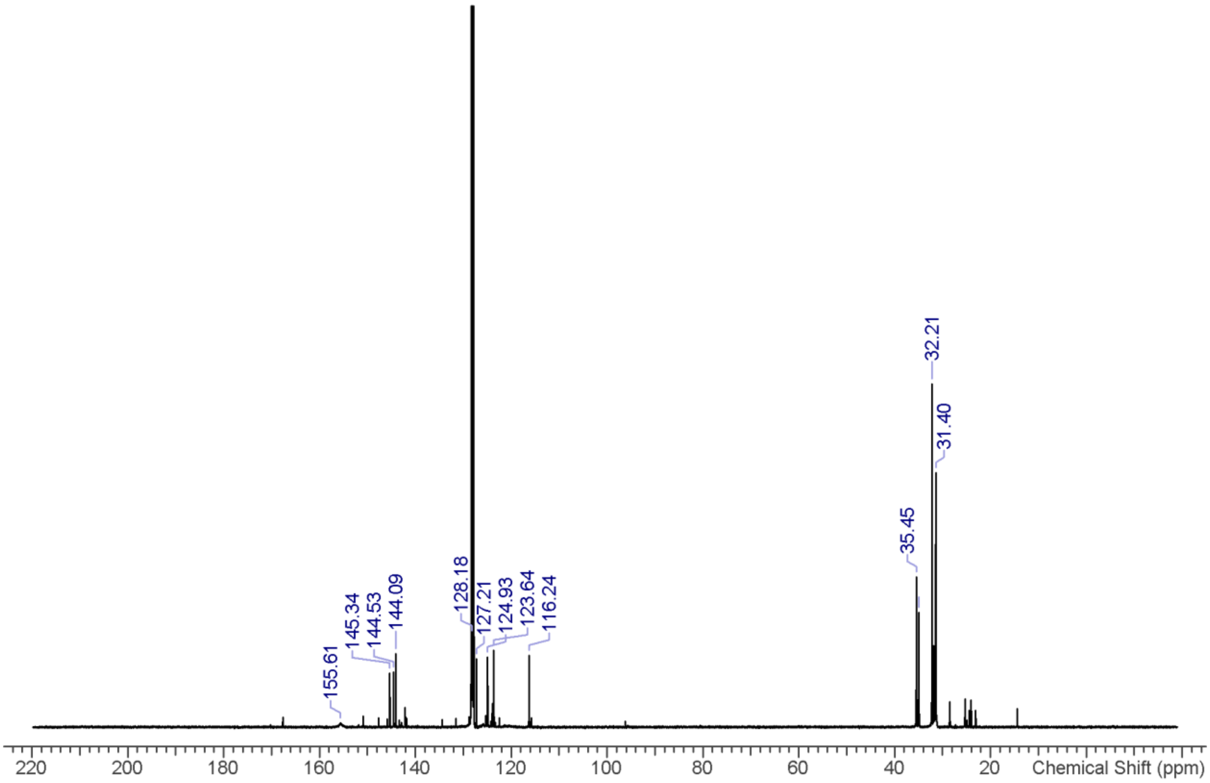

Figure S18: ^{13}C{^{1}H} NMR spectrum of **2Br**.

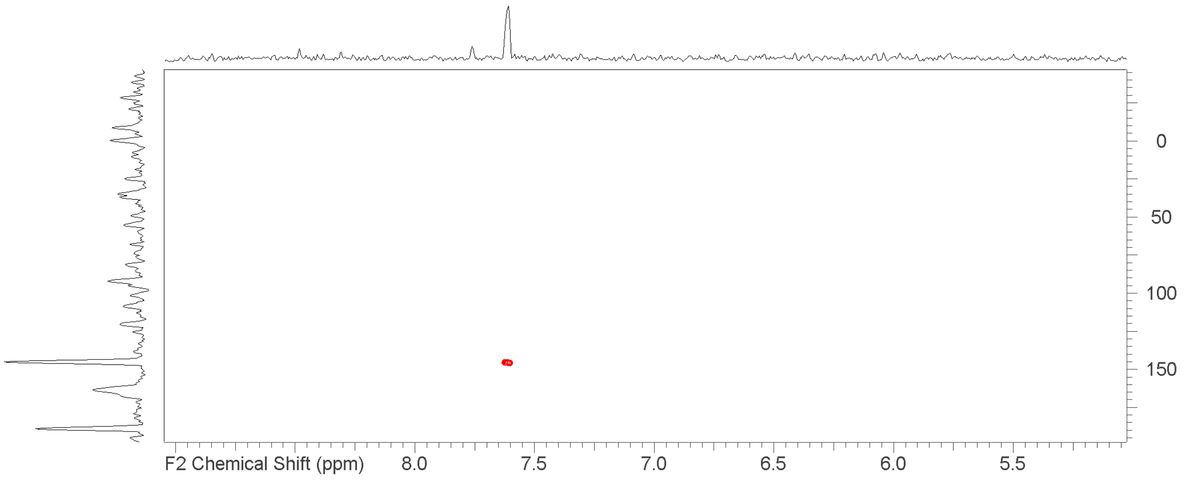

Figure S19: ^{1}H-^{15}N HMBC NMR spectrum of **2Br**.

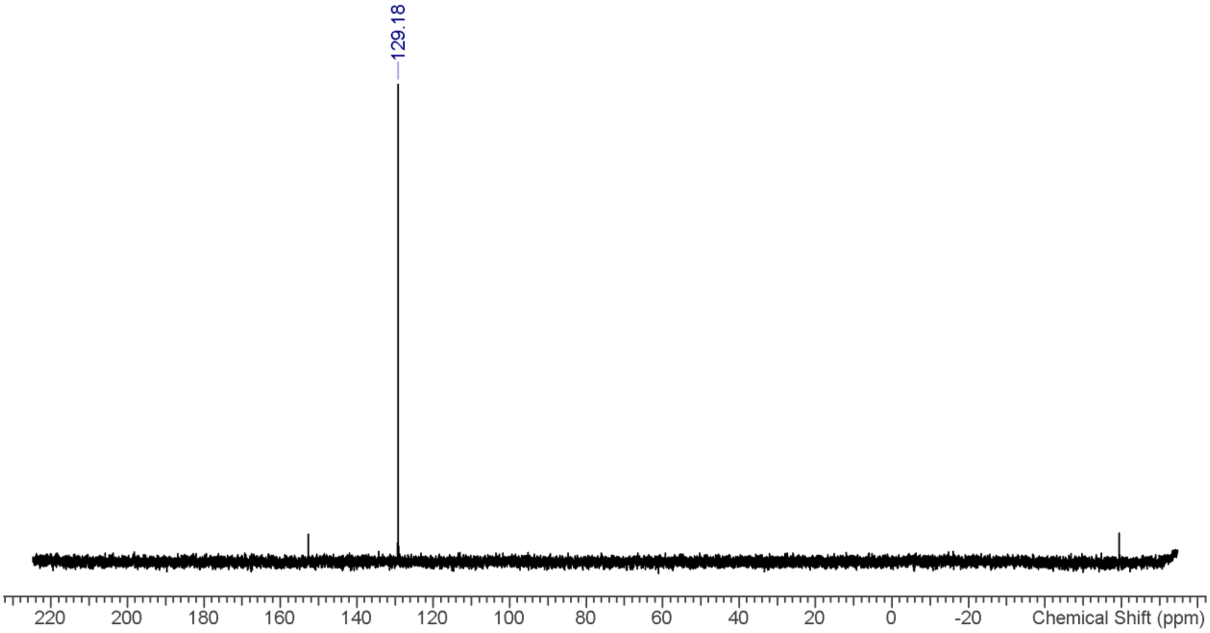

Figure S20: $^{29}Si\{^{1}H\}$ NMR spectrum of **2Br**.

2.5 RSiI (**2I**)

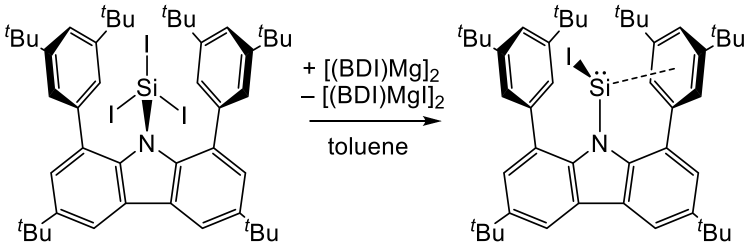

$RSiI_3$ (1700 mg, 1.60 mmol) and [(^{Mes}BDI)Mg]$_2$ (1450 mg, 1.64 mmol) were combined as solids. To the mixture 40 ml of toluene were added. The mixture was stirred overnight at room temperature, then volatiles were removed in vacuo. The mixture was then extracted with 60 + 10 ml of hexane. The solution was concentrated to approx. 5 ml and left undisturbed overnight, resulting in the deposition of orange RSiI. The supernatant was removed via syringe and the solid was dried in vacuo (830 mg, 1.02 mmol, 64%).

^{1}H NMR (C_6D_6): 1.27 (s, 18 H, $\nu_{1/2}$ = 16 Hz, Ar-^{t}Bu), 1.31 (s, 18 H, $\nu_{1/2}$ = 16 Hz, Ar-^{t}Bu), 1.43 (s, 18 H, carb-^{t}Bu), 7.51 (s, 2 H, $\nu_{1/2}$ = 22 Hz, *o*-C*H*), 7.59 (t, 2 H, J_{HH} = 1.6 Hz, *p*-C*H*), 7.63 (d, 2 H, J_{HH} = 1.8 Hz, $C^{2,7}H$), 7.64 (s, 2 H, $\nu_{1/2}$ = 22 Hz *o*-C*H*), 8.38 (d, 2 H, J_{HH} = 1.8 Hz, $C^{4,5}H$). **^{13}C NMR** (C_6D_6; br = $\nu_{1/2}$ = 13 Hz): 31.14 (br s, Ar-C(CH_3)$_3$), 31.68 (br s, Ar-C(CH_3)$_3$), 32.16 (s, Carb-C(CH_3)$_3$), 34.95 (s, Carb-*C*(CH_3)$_3$), 35.46 (s, $\nu_{1/2}$ = 8.5 Hz, Ar-*C*(CH_3)$_3$), 116.34 (s), 118.25 (br s), 123.70 (s), 125.35 (s), 126.51 (br s), 127.64 (s), 144.36 (s), 144.57 (s), 145.29 (s), 154.98 (br s, *m*-*C*), 156.42 (br s, *m*-*C*). **^{15}N NMR** (C_6D_6): +139.6. **^{29}Si NMR** (C_6D_6): +152.8 (s). **EA** found (calc. for $C_{48}H_{64}NSiI$): 71.48 (71.17) 8.15 (7.96) 1.81 (1.73).

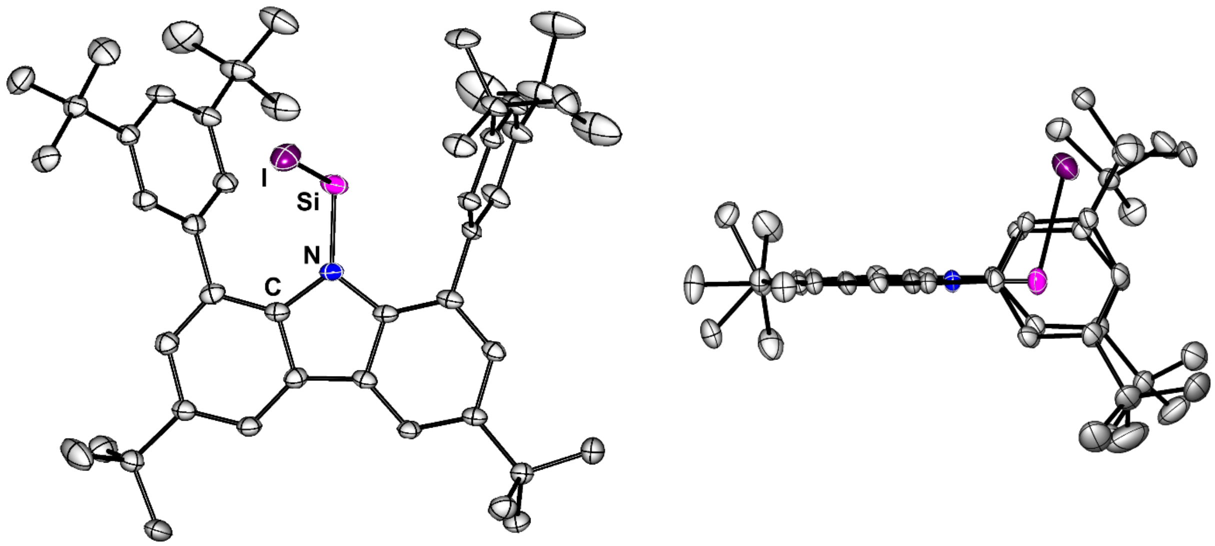

Figure S21: Molecular structure of **2I**.

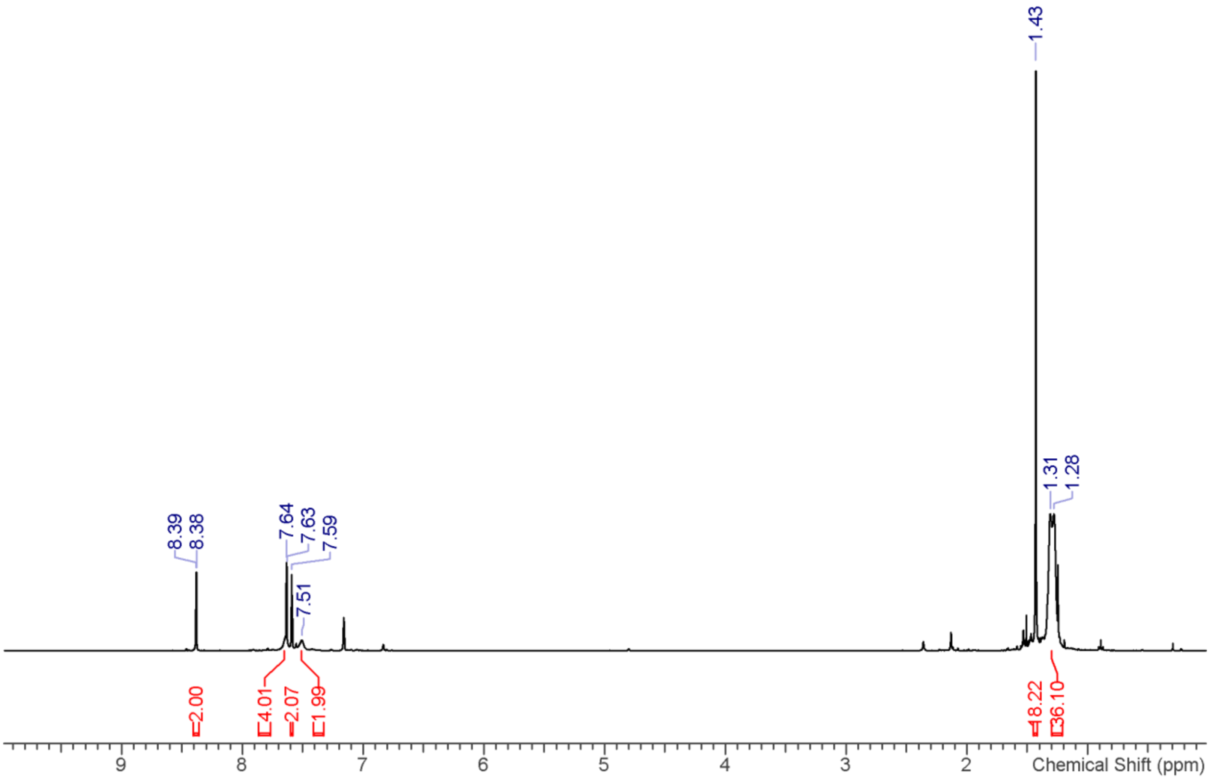

Figure S22: ^{1}H NMR spectrum of **2l**.

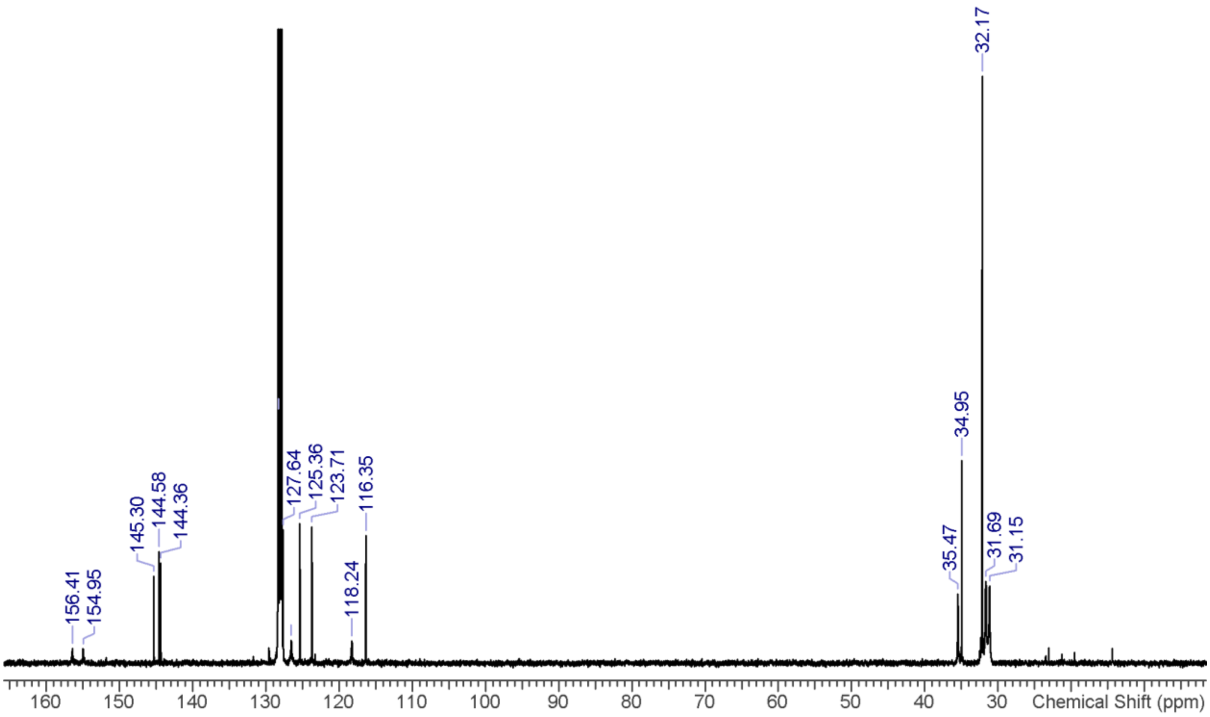

Figure S23: $^{13}C\{^{1}H\}$ NMR spectrum of **2l**.

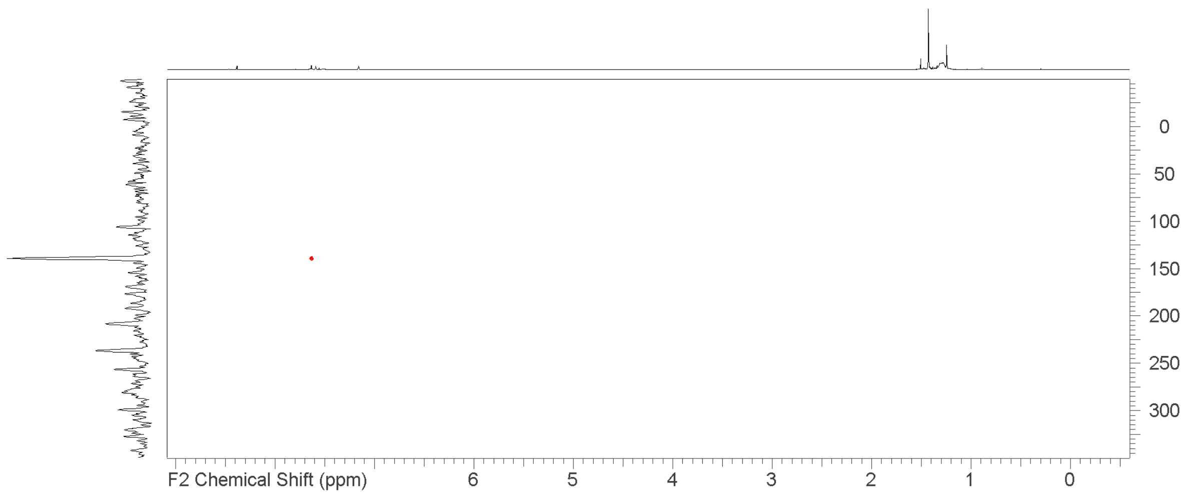

Figure S24: ^{1}H-^{15}N HMBC NMR spectrum of **2l**.

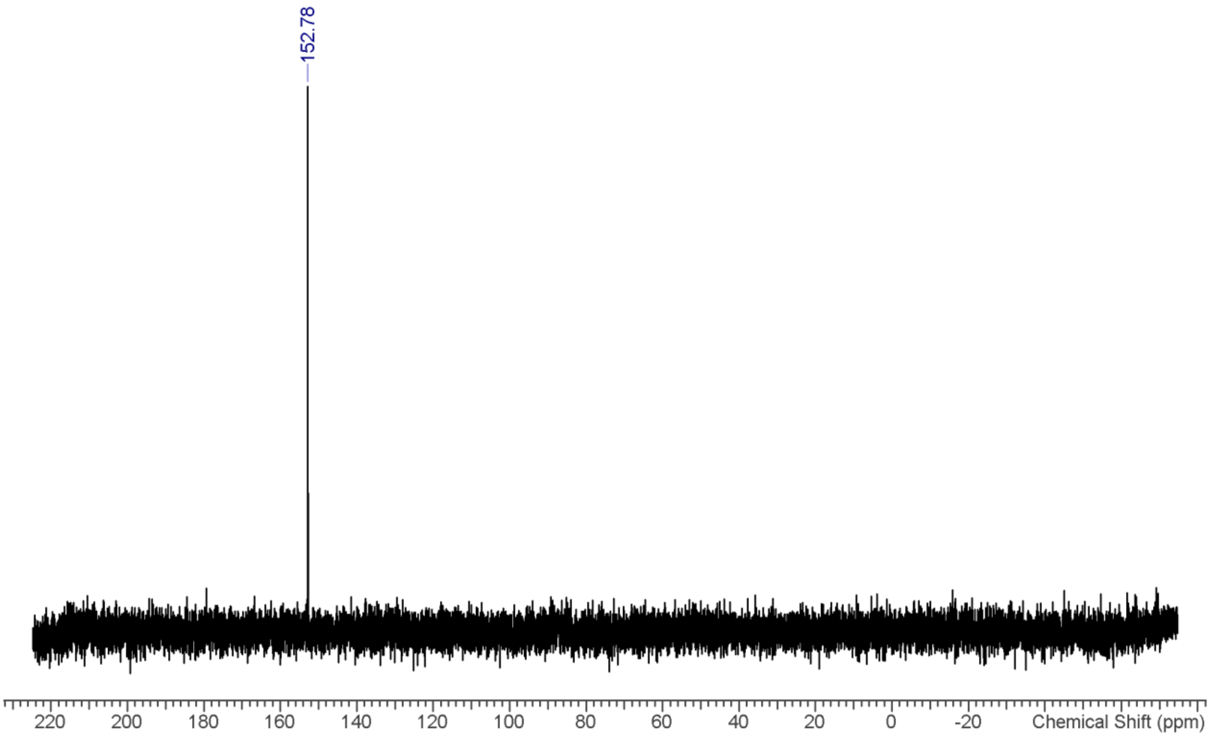

Figure S25: $^{29}Si\{^{1}H\}$ NMR spectrum of **2l**.

2.5.1 R_2Si

As a by-product of the reduction reaction, small amounts of R_2Si were isolated by fractional crystallisation of the product mixture of **2I**.

^{1}H NMR (C_6D_6): 1.34 (s, 18 H, $\nu_{1/2}$ = 4.4 Hz), 1.37 (s, 36 H, $\nu_{1/2}$ = 13.2 Hz), 1.47 (s, 36 H, $\nu_{1/2}$ = 9.5 Hz), 1.53 (s, 18 H, $\nu_{1/2}$ = 4.3 Hz), 7.05 (s, 2 H, $\nu_{1/2}$ = 5.6 Hz), 7.10 (s, 2 H, $\nu_{1/2}$ = 6.0 Hz), 7.26 (s, 2 H, $\nu_{1/2}$ = 5.5 Hz), 7.42 (s, 4 H, $\nu_{1/2}$ = 7.3 Hz), 7.75 (s, 2 H, $\nu_{1/2}$ = 6.9 Hz), 7.91 (s, 2 H, $\nu_{1/2}$ = 5.6 Hz). **^{13}C NMR** (C_6D_6): 32.07 (s, $\nu_{1/2}$ = 5.0 Hz), 32.23 (s, $\nu_{1/2}$ = 7.1 Hz), 32.49 (s, $\nu_{1/2}$ = 7.8 Hz), 34.50 (s, $\nu_{1/2}$ = 4.5 Hz), 34.78 (s, $\nu_{1/2}$ = 4.5 Hz), 35.09 (s, $\nu_{1/2}$ = 5.4 Hz), 35.31 (s, $\nu_{1/2}$ = 5.0 Hz), 114.72 (s, $\nu_{1/2}$ = 6.0 Hz), 115.64 (s, $\nu_{1/2}$ = 5.7 Hz), 120.59 (s, $\nu_{1/2}$ = 7.3 Hz), 121.54 (s, $\nu_{1/2}$ = 7.4 Hz), 123.83 (s, $\nu_{1/2}$ = 6.8 Hz), 126.80 (s, $\nu_{1/2}$ = 6.4 Hz), 129.96 (s, $\nu_{1/2}$ = 7.4 Hz), 130.29 (s, $\nu_{1/2}$ = 7.3 Hz), 130.64 (s, $\nu_{1/2}$ = 5.3 Hz), 132.59 (s, $\nu_{1/2}$ = 4.9 Hz), 139.90 (s, $\nu_{1/2}$ = 4.8 Hz), 141.09 (s, $\nu_{1/2}$ = 5.2 Hz), 141.37 (s, $\nu_{1/2}$ = 4.9 Hz), 143.72 (s, $\nu_{1/2}$ = 5.2 Hz), 144.74 (s, $\nu_{1/2}$ = 4.9 Hz), 145.47 (s, $\nu_{1/2}$ = 4.8 Hz), 149.81 (s, $\nu_{1/2}$ = 4.6 Hz), 150.32 (s, $\nu_{1/2}$ = 13.3 Hz). **^{15}N NMR** (C_6D_6): n. obs. **^{29}Si NMR** (C_6D_6): +147.5 (s). **EA** found (calc. for $C_{96}H_{128}N_2Si$): no satisfactory analysis could be obtained.

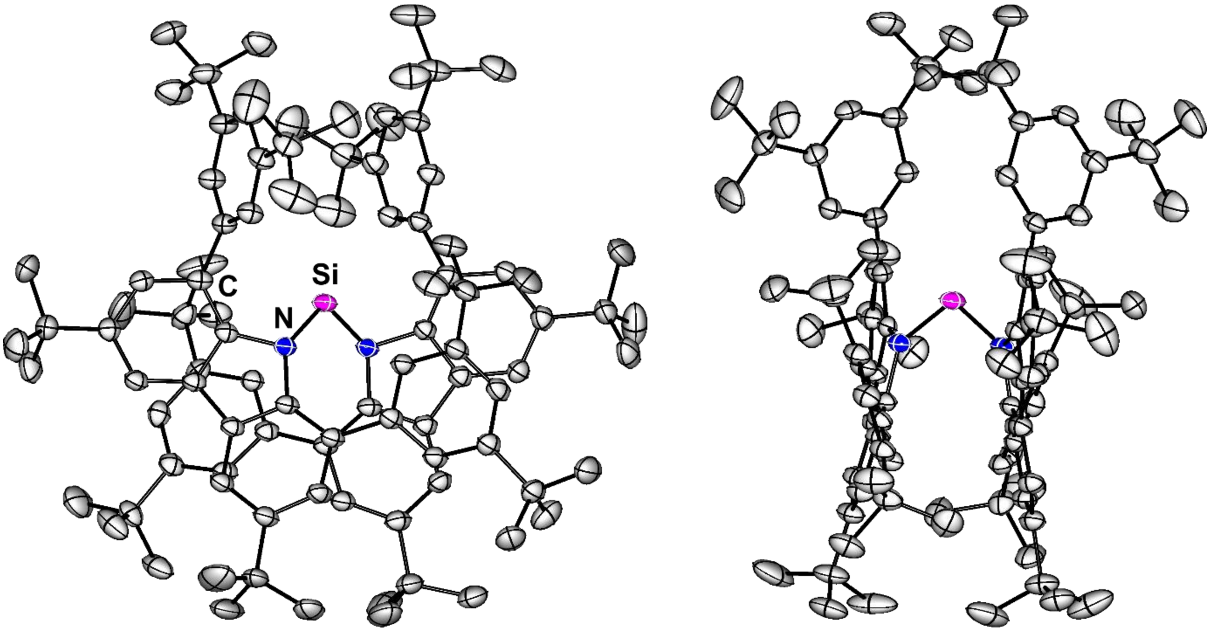

Figure S26: Molecular structure of $\mathbf{R_2Si}$. $V_{bur,3.5}$ 84.6%, $V_{bur,6.0}$ 77.8%.

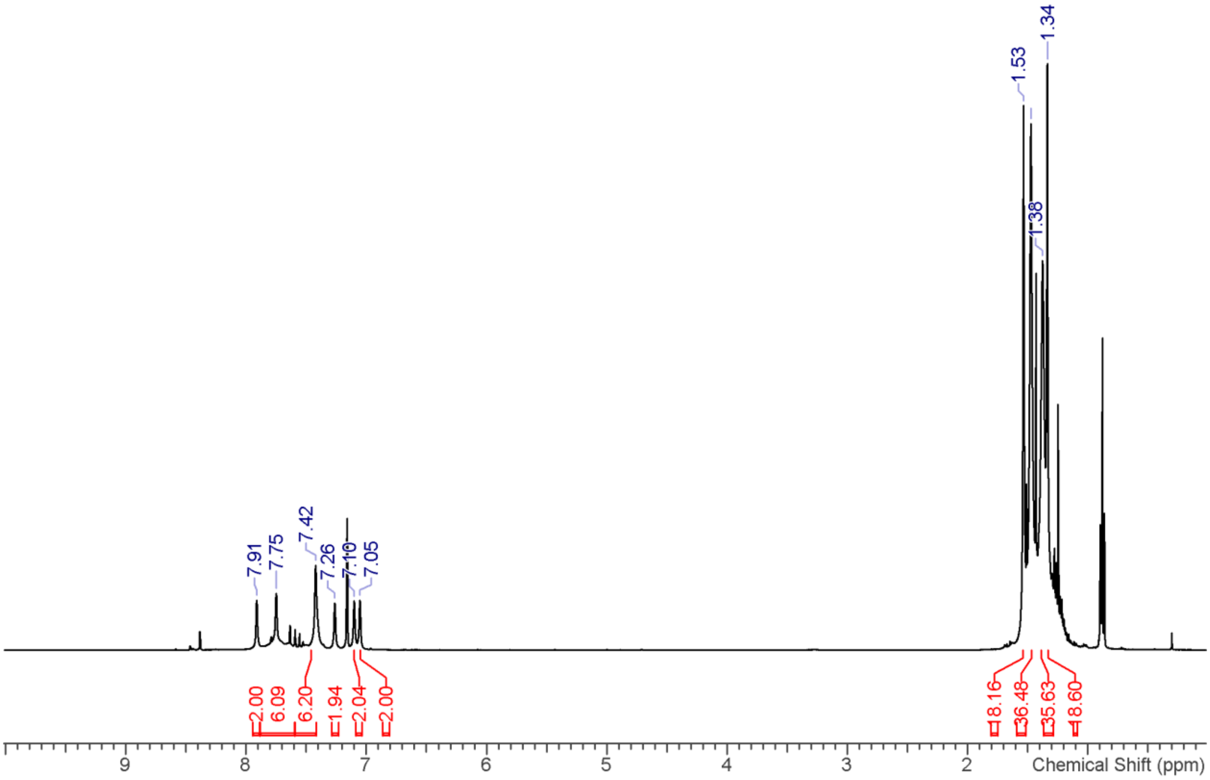

Figure S27: ^{1}H NMR spectrum of **R_2Si**.

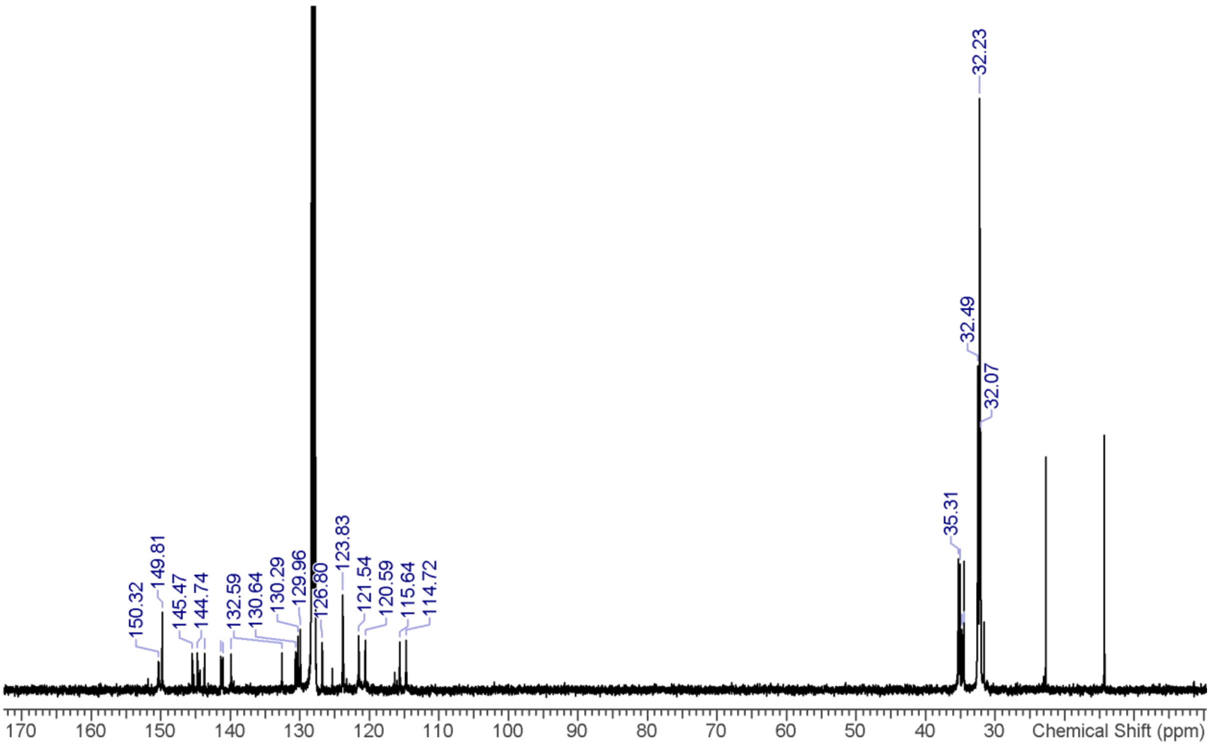

Figure S28: $^{13}C\{^{1}H\}$ NMR spectrum of **R_2Si**.

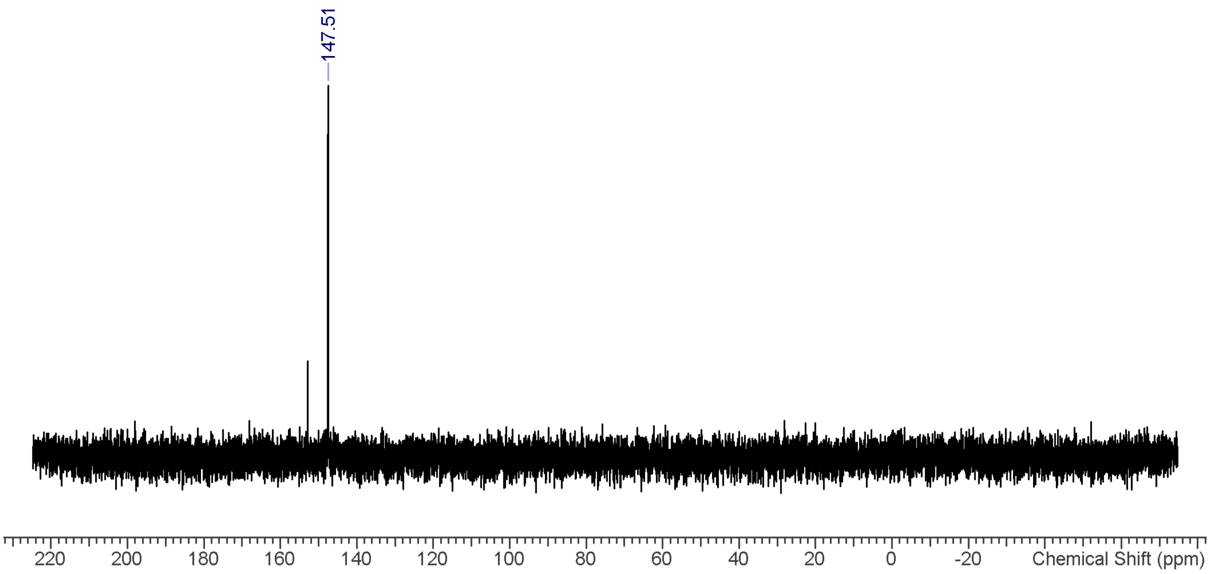

Figure S29: $^{29}Si\{^1H\}$ NMR spectrum of **R_2Si**.

2.5.2 RSiI-II (2I-II)

Presumably a catalytic amount of HI formed on one occasion due to partial hydrolysis, and caused decomposition of the whole batch of **2I** within minutes at the extraction with hexane and crystallisation step. The product was isolated after workup as pale yellow crystalline material (**RSiI-II**).

^{1}H NMR (C_6D_6): 1.19 (s, 9 H, ^{t}Bu), 1.33 (s, 9 H, ^{t}Bu), 1.41 (s, 9 H, ^{t}Bu), 1.44 (s, 9 H, ^{t}Bu), 1.46 (s, 9 H, ^{t}Bu), 1.49 (s, 9 H, ^{t}Bu), 5.42 (s, 1 H, J_{HSi} = 299 Hz, Si*H*), 7.49 (app. t, J_{HH} = 1.6 Hz, 1 H, Ar-C*H*), 7.65 (d, J_{HH} = 1.5 Hz, 1 H, Ar-C*H*), 7.72 (app. t, J_{HH} = 1.9 Hz, 1 H, Ar-C*H*), 7.75 (d, J_{HH} = 2.0 Hz, 1 H, Carb-C*H*), 7.96 (app. t, J_{HH} = 1.6 Hz, 1 H, Ar-C*H*), 8.26 (br s, 1 H, Ar-C*H*), 8.37 (d, J_{HH} = 1.7 Hz, 1 H, Carb-C*H*), 8.40 (d, J_{HH} = 1.8 Hz, 2 H, Carb-C*H*). **^{13}C NMR** (C_6D_6): 31.19 (s, C(CH$_3$)$_3$), 31.64 (s, C(CH$_3$)$_3$), 31.90 (s, C(CH$_3$)$_3$), 32.01 (s, C(CH$_3$)$_3$), 32.07 (s, C(CH$_3$)$_3$), 32.13 (s, C(CH$_3$)$_3$), 35.00 (s, C(CH$_3$)$_3$), 35.01 (s, C(CH$_3$)$_3$), 35.19 (s, C(CH$_3$)$_3$), 35.30 (s, C(CH$_3$)$_3$), 35.37 (s, C(CH$_3$)$_3$), 38.25 (s, C(CH$_3$)$_3$), 116.44 (s, *C*H), 117.46 (s, *C*H), 121.34 (s, *C*H), 121.42 (s, *C*H), 122.08 (s), 122.38 (s, *C*H), 122.91 (s, *C*H), 124.16 (s), 124.30 (s, *C*H), 125.25 (s, *C*H), 126.00 (s, *C*H), 127.82 (s), 127.94 (s), 128.06 (s), 128.18 (s), 128.90 (s), 129.59 (s), 131.82 (s), 136.43 (s), 139.64 (s), 140.62 (s), 141.26 (s), 142.81 (s), 143.47 (s), 145.39 (s), 145.54 (s), 151.58 (s), 152.17 (s), 154.01 (s), 156.69 (s). **^{15}N NMR** (C_6D_6): +112.0. **^{29}Si{^{1}H} NMR** (C_6D_6): –46.2 (s). **EA** found (calc. for $C_{48}H_{64}NSiI$): 71.63 (71.17) 8.03 (7.96) 1.88 (1.73).

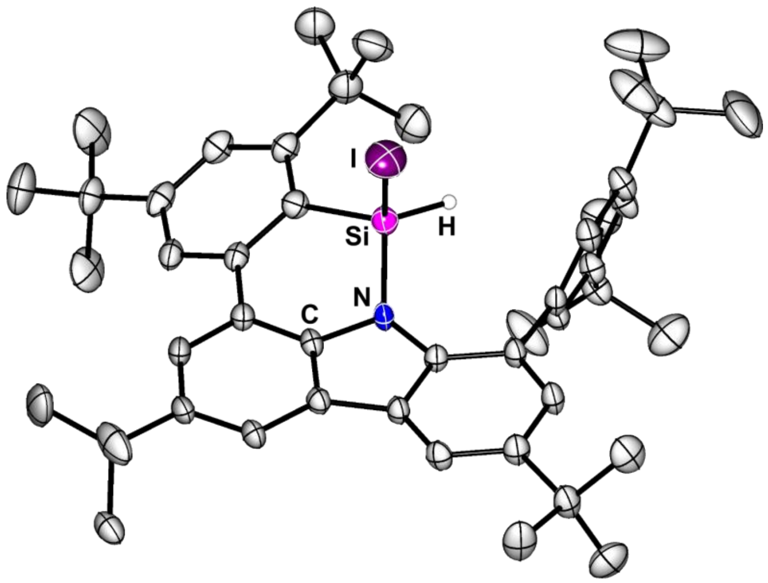

Figure S30: Molecular structure of **2I-II**.

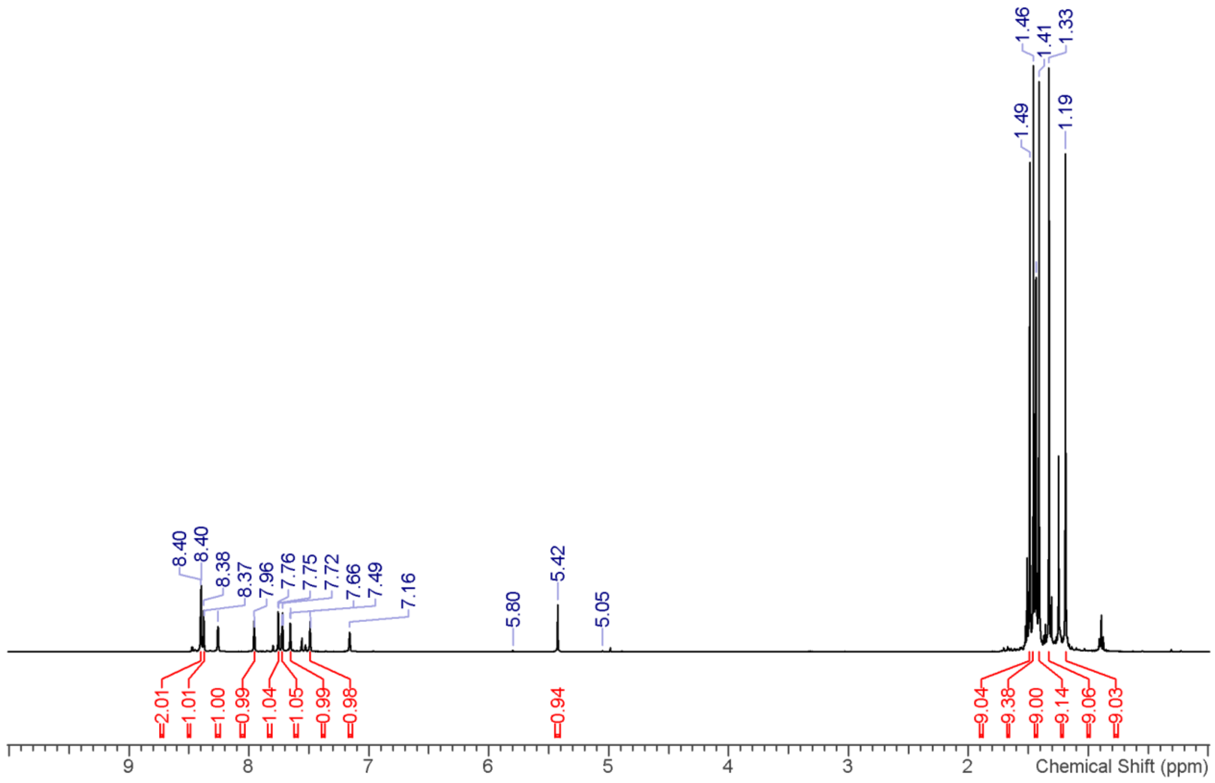

Figure S31: ¹H NMR spectrum of **2I-II**.

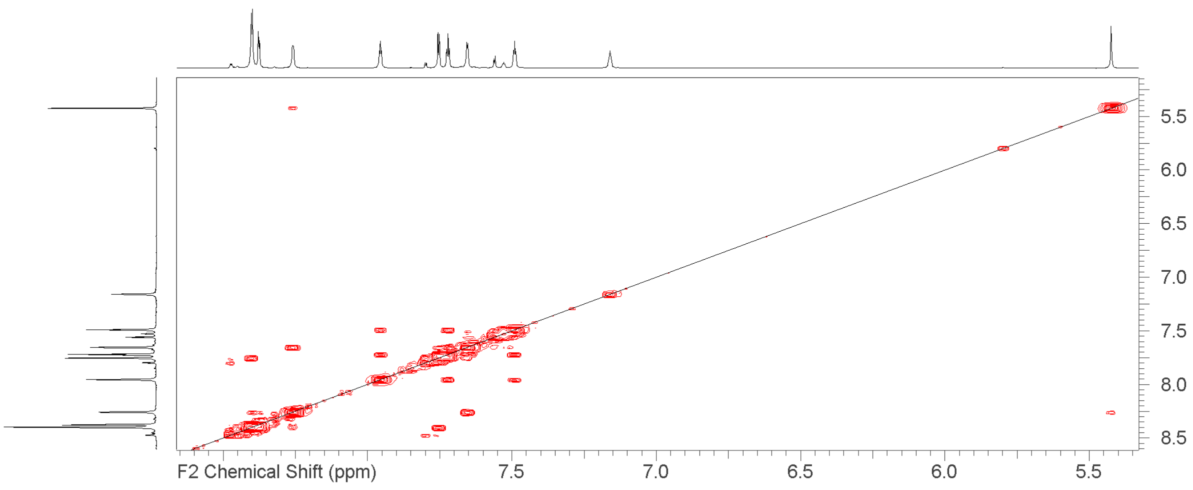

Figure S32: $^{1}H,^{1}H$ COSY NMR spectrum of **2l-II**.

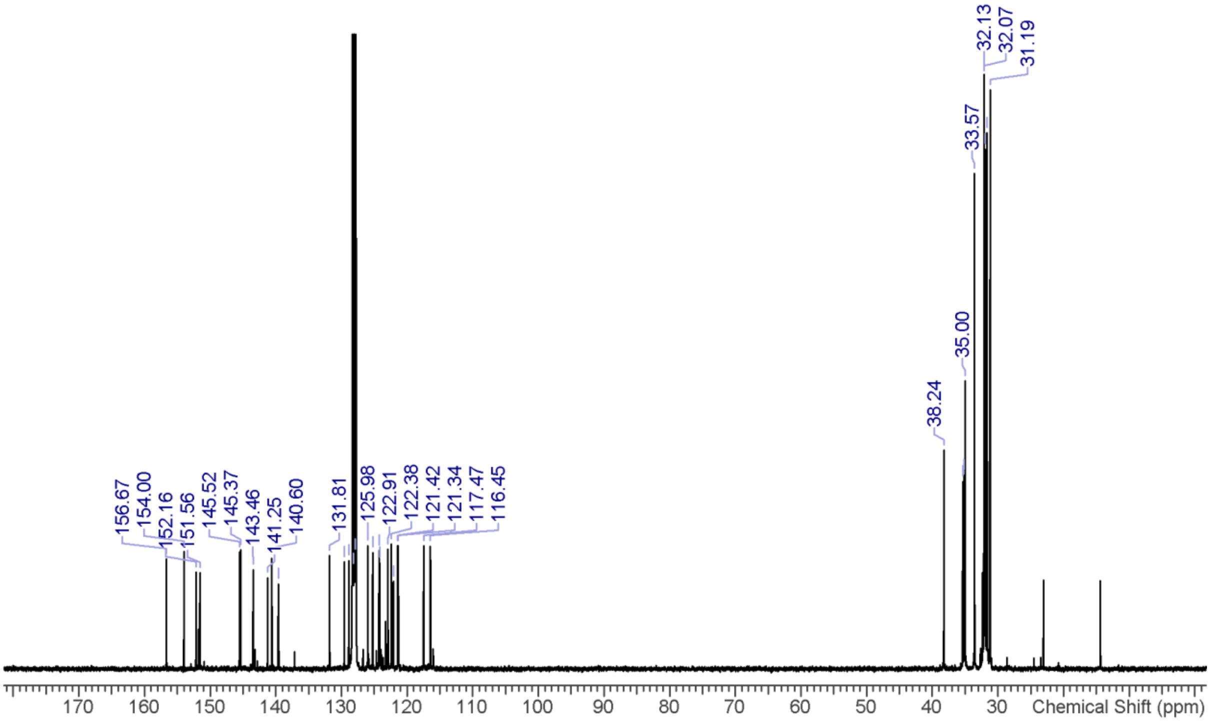

Figure S33: $^{13}C\{^{1}H\}$ NMR spectrum of **2l-II**.

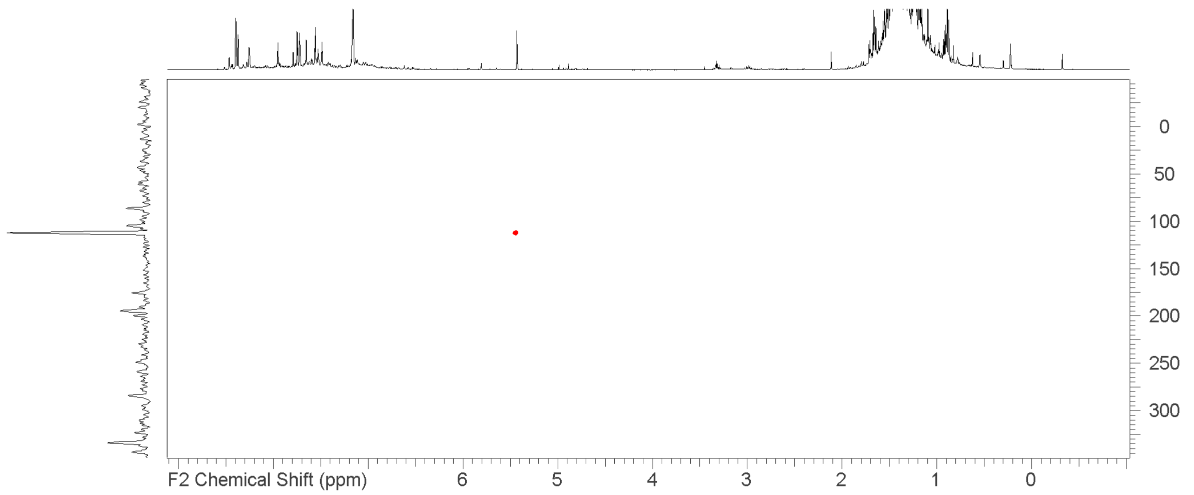

Figure S34: $^{1}H,^{15}N$ HMBC NMR spectrum of **2I-II**.

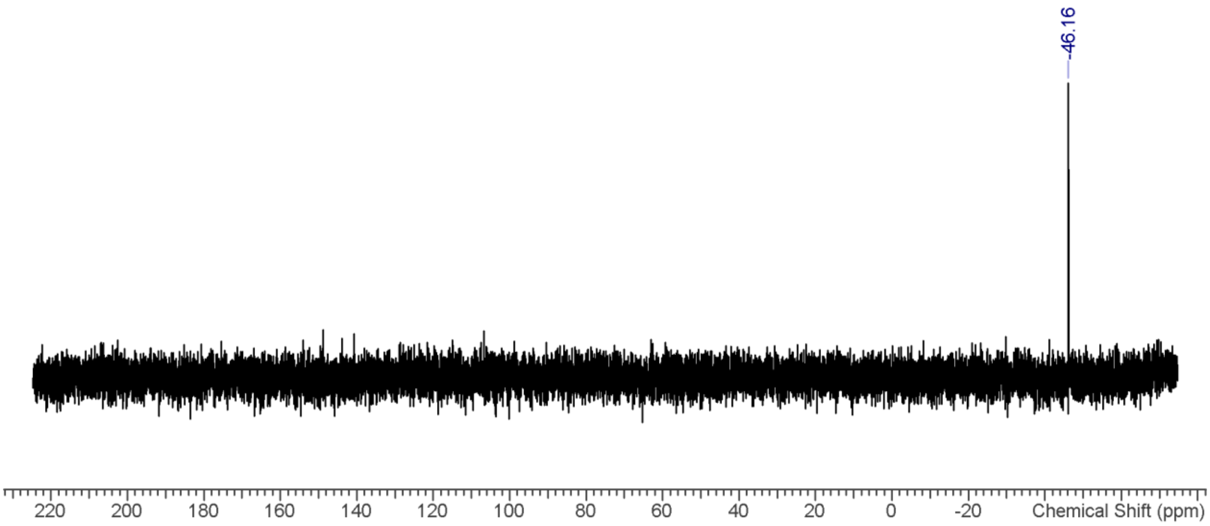

Figure S35: $^{29}Si\{^{1}H\}$ NMR spectrum of **2I-II**.

2.6 [RSi][Al(OC_4F_9)$_4$] (**3**)

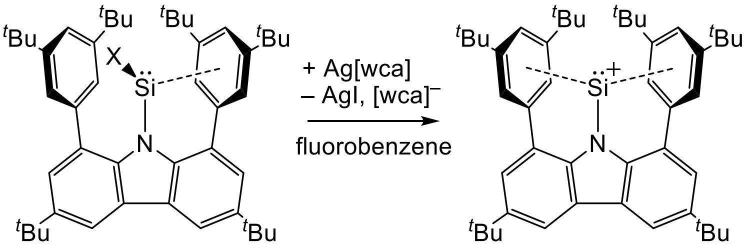

A mixture of Ag[Al(OC_4F_9)$_4$] (560 mg, 0.521 mmol) and RSiI (425 mg, 0.525 mmol) was treated with 10 ml of fluorobenzene and then stirred at ambient temperature for 30 minutes. The initially orange mixture rapidly darkened. Afterwards, the suspension was filtered, affording an orange solution. The solution was slowly concentrated at ambient temperature to about 1 ml and left undisturbed overnight, which resulted in the deposition of X-ray quality orange crystals. The supernatant was transferred to another flask. In both fractions, volatiles were removed in vacuo, affording 486 mg crystalline and 272 mg amorphous product (combined 758 mg, 0.434 mmol, 83%).

The crystals darkened quickly in the perfluorinated ether. Solutions turned black upon exposure to air.

^{1}H NMR (C_6H_5F/C_6D_6): 1.13 (s, 36 H, Ar-^{t}Bu), 1.38 (s, 18 H, Carb-^{t}Bu), 7.19 (d, J_{HH} = 1.9 Hz, 4 H, *o*-C*H*), 7.43 (d, J_{HH} = 1.7 Hz, 2 H, $C^{2,7}$*H*), 7.62 (t, J_{HH} = 1.9 Hz, 2 H, *o*-C*H*), 8.11 (d, J_{HH} = 1.9 Hz, 2 H, $C^{4,5}$*H*). **^{13}C{^{1}H} NMR** (C_6H_5F/C_6D_6): 30.96 (s, Ar-C(CH_3)$_3$), 31.55 (s, Carb-C(CH_3)$_3$), 35.59 (s, Ar-*C*(CH_3)$_3$), 36.18 (s, Carb-*C*(CH_3)$_3$), 118.34 (s, *o*-CH), 120.82 (s, $C^{4,5}$H), 121.99 (s), 122.70 (s), 123.74 (s), 126.94 (s, $C^{1,8}$), 129.42 (s, *p*-CH), 144.06 (s, Ar-*C*-*ipso*, calc. 143.5), 146.13 (s, N*C*, calc. 144.0), 152.34 (s, calc. 151.7), 164.26 (m-C, calc. 164.8). **^{15}N NMR** (C_6H_5F/C_6D_6): 206.2 (s). **^{29}Si NMR** (C_6H_5F/C_6D_6): 56.8 (s). **EA** found (calc. for $C_{64}H_{64}NO_4F_{36}AlSi$): C 47.16 (46.58), H 3.97 (3.91), N 1.12 (0.85).

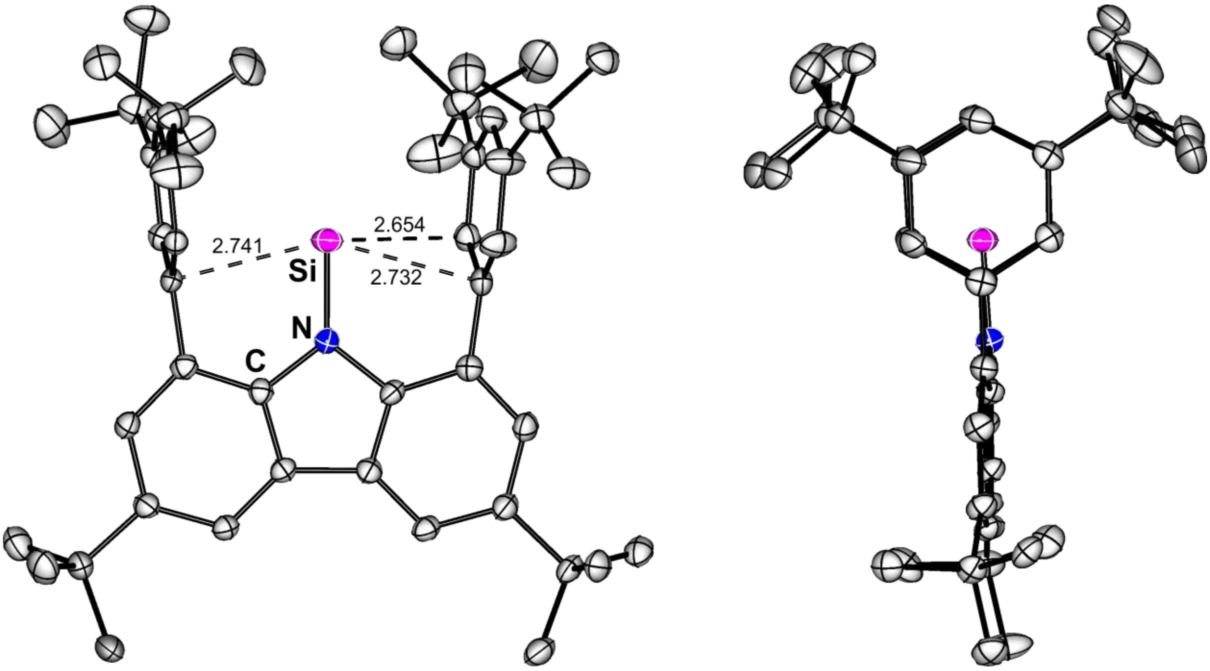

Figure S36: Molecular structure of **3**. Anion omitted.

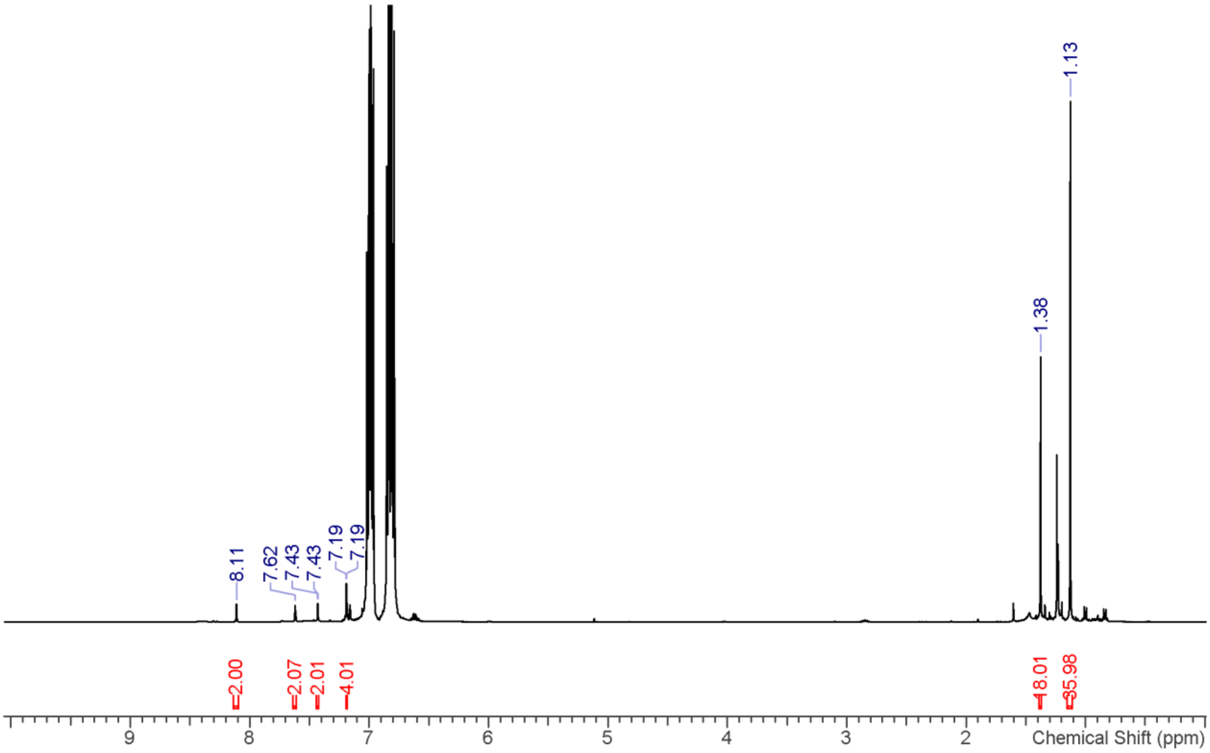

Figure S37: ^{1}H NMR spectrum of **3**.

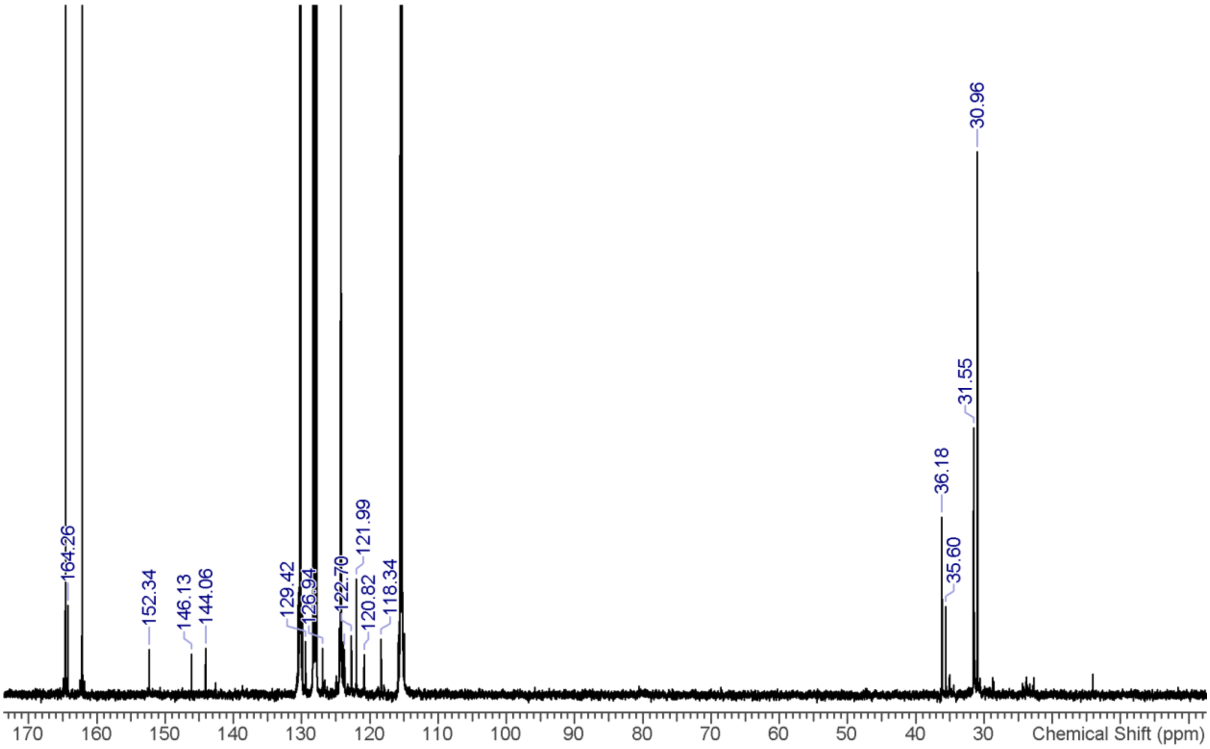

Figure S38: ^{13}C{^{1}H} NMR spectrum of **3**.

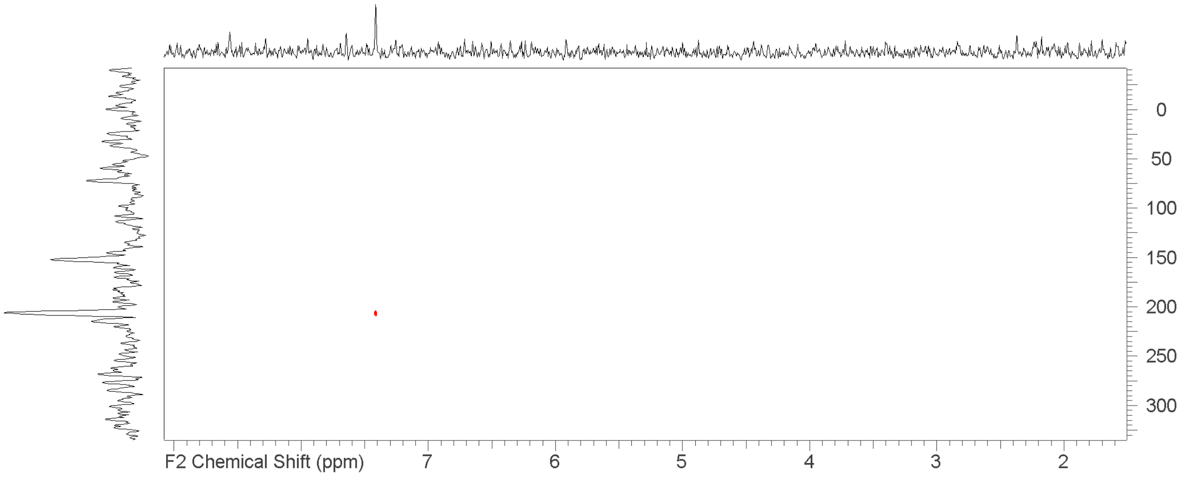

Figure S39: ^{1}H-^{15}N HMBC NMR spectrum of **3**.

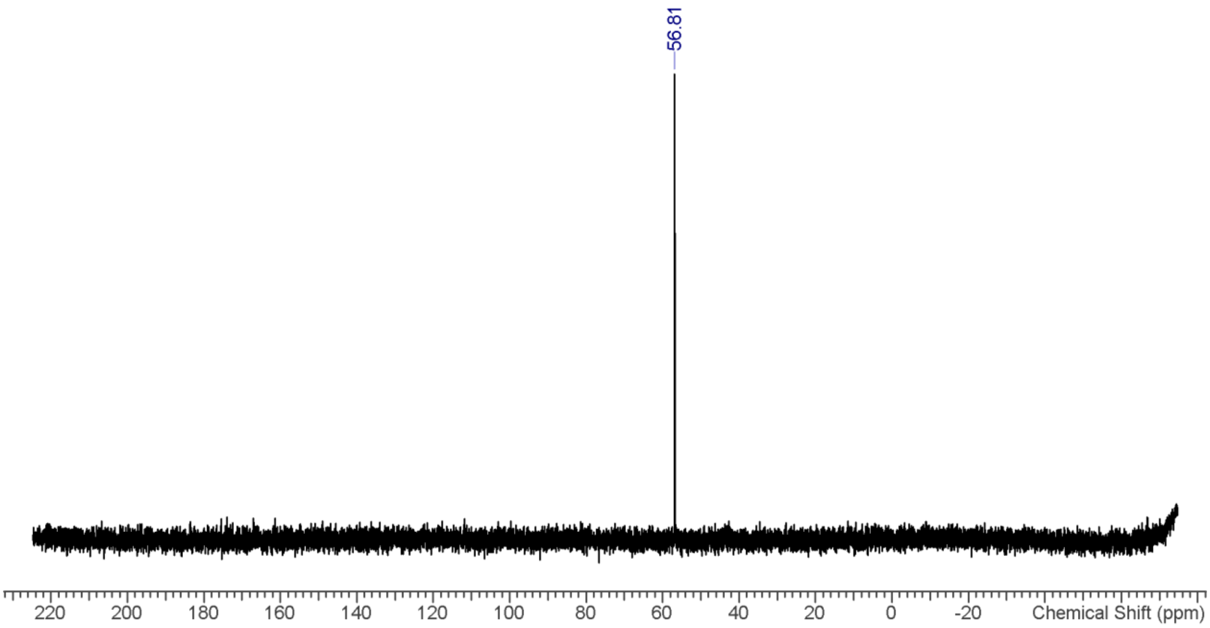

Figure S40: $^{29}Si\{^{1}H\}$ NMR spectrum of **3**.

2.7 [RSi(H)(NH^{t}Bu)NH$_2$^{t}Bu][Al(OC$_4$F$_9$)$_4$] (**6**)

A solution of ^{t}BuNH$_2$ in 1.1 ml PhF (2.5 mg, 0.034 mmol) was added to a solution of [RSi][Al(OC$_4$F$_9$)$_4$] (30 mg, 0.017 mmol) in 0.5 ml PhF. The mixture was concenctrated to approx. 0.1 ml. Afterwards 0.5 ml C_6D_6 were added to study the product by NMR spectroscopy. Attempts of large scale reactions and isolation of the product failed.

^{1}H NMR (C_6H_5F/C_6D_6): 0.39 (s, 9 H, $\nu_{1/2}$ = 2.6 Hz, ^{t}BuNH), 0.64 (s, 9 H, $\nu_{1/2}$ = 10.2 Hz, ^{t}BuNH$_2$), 1.17 (s, 18 H, $\nu_{1/2}$ = 12.9 Hz, Carb-^{t}Bu), 1.32 (s, 36 H, $\nu_{1/2}$ = 16.1 Hz, Carb-^{t}Bu), 1.59 (^{1}H, br s, $\nu_{1/2}$ = 8.4 Hz, N*H*), 3.03 (2 H, br s, $\nu_{1/2}$ = 20.9 Hz, N*H*$_2$), 5.20 (^{1}H, J(^{1}H-^{29}Si) = 296 Hz, d-J$_{HH}$ = 7.6, t-J$_{HH}$ = 2.1 Hz, 1 H, Si*H*), 7.25-7.60 (br singlets), 8.29 (d, 2 H, C^{4,5}*H*). **^{29}Si NMR** (C_6H_5F/C_6D_6): –29.4 (s).

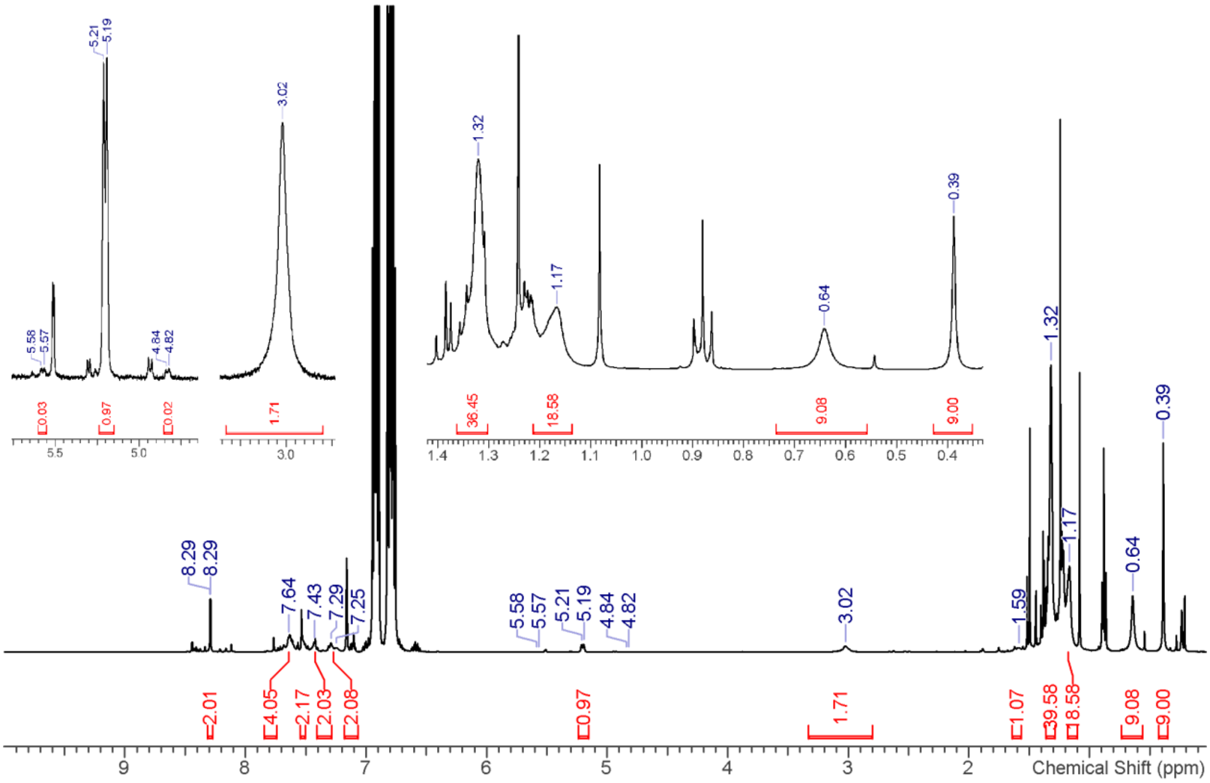

Figure S41: ^{1}H NMR spectrum of **6**.

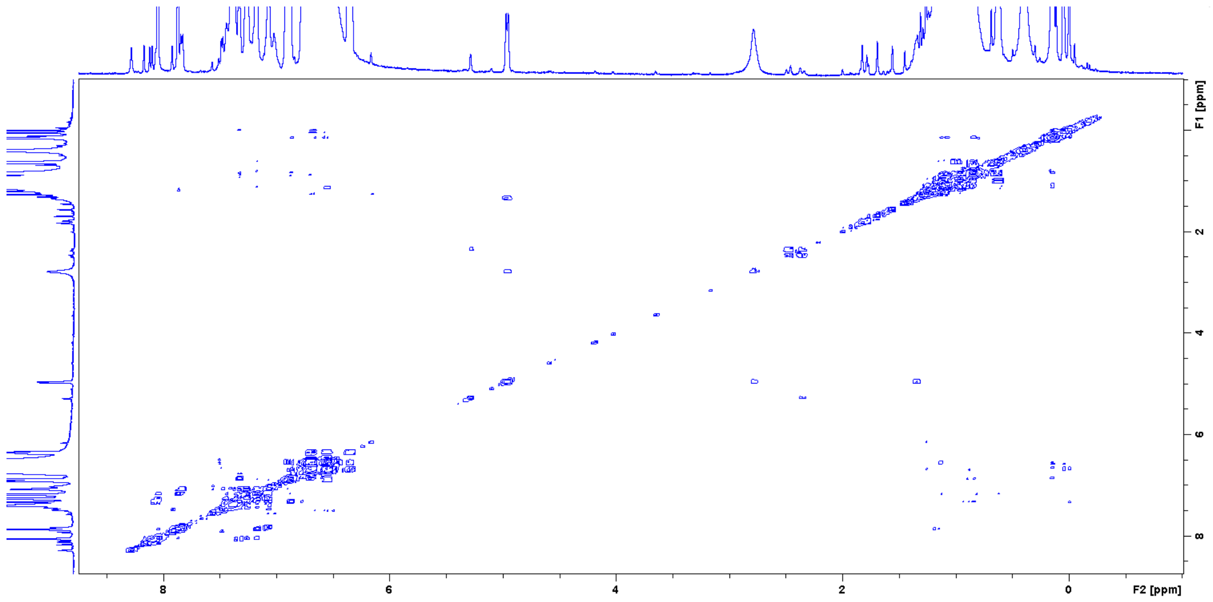

Figure S42: $^{1}H,^{1}H$ COSY NMR spectrum of **6**.

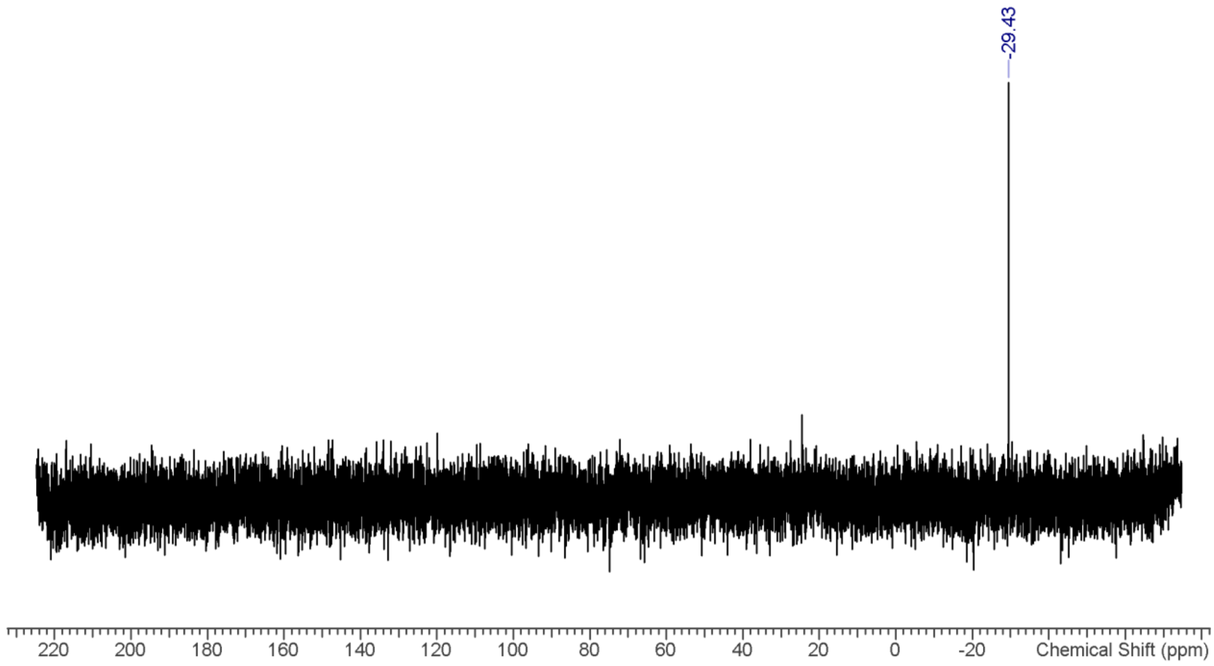

Figure S43: $^{29}Si\{^{1}H\}$ NMR spectrum of **6**.

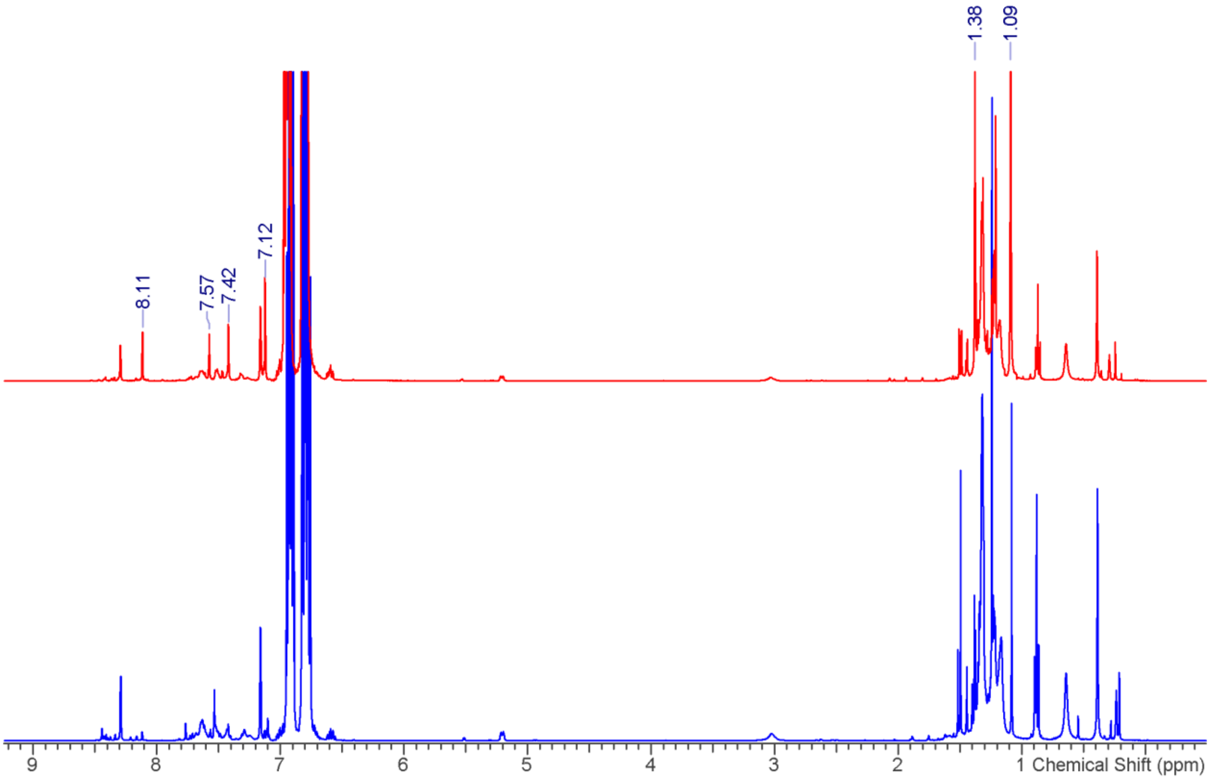

Figure S44: ^{1}H NMR spectrum of **6** (blue) and after addition of one equivalent of **3** (red), with labelled resonances of **3**.

3 Crystallographic Details

Table S1: Crystallographic details for **1Cl**, **1Br** and **1I**.

	***$RSiCl_3$* (1Cl)**	***$RSiBr_3$* (1Br)**	***$RSiI_3$* (1I)**
CCDC #	*2010372*	*2010373*	*2010374*
Empirical formula	$C_{48}H_{64}Cl_3NSi$	$C_{48}H_{64}Br_3NSi$	$C_{48}H_{64}I_3NSi$
FW [g mol^{-1}]	*789.44*	*922.82*	*1063.79*
Wavelength [Å]	*1.34143*	*0.71073*	*1.34143*
Temperature [K]	*150(2)*	*200(2)*	*150(2)*
Crystal system	*triclinic*	*triclinic*	*triclinic*
Space group	*P-1*	*P-1*	*P-1*
a [Å]	*12.7126(14)*	*12.8212(3)*	*12.9764(5)*
b [Å]	*18.9549(15)*	*19.0956(5)*	*19.4140(8)*
c [Å]	*19.902(2)*	*20.1700(5)*	*20.3179(11)*
α *[°]*	*78.755(7)*	*77.972(2)*	*77.090(4)*
β *[°]*	*85.476(9)*	*85.054(2)*	*84.729(4)*
γ *[°]*	*79.024(8)*	*78.257(2)*	*76.058(3)*
V [Å^3]	*4613.3(8)*	*4723.7(2)*	*4838.3(4)*
Z	*4*	*4*	*4*
ρ_{calc} *(g·cm^{-3})*	*1.137*	*1.298*	*1.460*
μ	*1.483*	*2.618*	*10.502*
F(000)	*1696*	*1912*	*2128*
reflections collected	*58892*	*40838*	*58474*
independent reflections	*21767*	*18571*	*22512*
reflectionsGT (I > 2σ(I))	*15116*	*11721*	*16863*
R_{int}	*0.0548*	*0.0696*	*0.0359*
parameters	*1025*	*1127*	*1018*
restraints	*168*	*642*	*19*
GooF	*0.896*	*1.019*	*0.921*
R1	*0.0413*	*0.0513*	*0.0314*
R1 (all)	*0.0614*	*0.1015*	*0.0434*
wR2	*0.0991*	*0.1114*	*0.0780*
wR2 (all)	*0.1048*	*0.1338*	*0.0802*
weight factors	*0.0664*<br>*0*	*0.0642*<br>*2.9856*	*0.0500*<br>*0*

Table S2: Crystallographic details for **2Br**, **2I** and **RSiI-II**.

	RSiBr (2Br)	***RSiI (2I)***	***[RSi][Al(OC₄F₉)₄] (3)***
CCDC #	*2010375*	*2010376*	*2010377*
Empirical formula	$C_{48}H_{64}BrNSi$	$C_{48}H_{64}INSi$	$C_{70}H_{69}AlF_{37}NO_4Si$
FW [g mol^{-1}]	*763.00*	*809.99*	*1746.33*
Wavelength [Å]	*1.34143*	*0.71073*	*1.34143*
Temperature [K]	*130(2)*	*200(2)*	*130(2)*
Crystal system	*triclinic*	*triclinic*	*monoclinic*
Space group	*P-1*	*P-1*	$P2_1/c$
a [Å]	*12.2093(3)*	*12.2929(3)*	*13.1750(4)*
b [Å]	*12.5518(3)*	*12.5641(4)*	*29.0444(14)*
c [Å]	*15.2564(4)*	*15.2526(5)*	*20.9179(9)*
α *[°]*	*91.101(2)*	*91.146(2)*	*90*
β *[°]*	*105.162(2)*	*104.331(2)*	*104.239(3)*
γ *[°]*	*98.015(2)*	*97.459(2)*	*90*
V [Å^3]	*2230.80(10)*	*2259.80(12)*	*7758.5(6)*
Z	*2*	*2*	*4*
ρ_{calc} *(g·cm^{-3})*	*1.136*	*1.190*	*1.495*
μ	*1.169*	*0.766*	*1.036*
F(000)	*816*	*852*	*3552*
reflections collected	*29997*	*20028*	*49426*
independent reflections	*9912*	*10344*	*15909*
reflectionsGT (I > 2σ(I))	*8585*	*8482*	*7676*
R_{int}	*0.0172*	*0.0283*	*0.0836*
parameters	*478*	*509*	*1487*
restraints	*0*	*206*	*2467*
GooF	*1.086*	*1.035*	*0.848*
R1	*0.0360*	*0.0452*	*0.0483*
R1 (all)	*0.0412*	*0.0568*	*0.1170*
wR2	*0.1088*	*0.1269*	*0.1096*
wR2 (all)	*0.1110*	*0.1342*	*0.1233*
weight factors	*0.0686*<br>*0.7491*	*0.0801*<br>*1.6226*	*0.0636*<br>*0*

Table S3: Crystallographic details for **R_2Si** and **3**.

	R_2Si	***RSiI decomp. (2I-II)***
CCDC #	*2010378*	*2010379*
Empirical formula	$C_{108}H_{138}F_2N_2Si$	$C_{48}H_{64}INSi$
FW [g mol^{-1}]	*1530.29*	*809.99*
Wavelength [Å]	*1.34143*	*1.34143*
Temperature [K]	*150(2)*	*150(2)*
Crystal system	*monoclinic*	*monoclinic*
Space group	$P2_1/n$	$P2_1/c$
a [Å]	*11.7681(7)*	*13.9792(7)*
b [Å]	*26.1310(7)*	*15.5182(5)*
c [Å]	*29.9458(14)*	*20.8315(8)*
α *[°]*	*90*	*90*
β *[°]*	*90.230(4)*	*101.480(4)*
γ *[°]*	*90*	*90*
V [Å^3]	*9208.6(7)*	*4428.6(3)*
Z	*4*	*4*
ρ_{calc} $(g \cdot cm^{-3})$	*1.104*	*1.215*
μ	*0.396*	*4.116*
F(000)	*3328*	*1704*
reflections collected	*54145*	*30224*
independent reflections	*18839*	*10335*
reflectionsGT ($I > 2\sigma(I)$)	*13945*	*7079*
R_{int}	*0.0305*	*0.0459*
parameters	*1367*	*652*
restraints	*1500*	*798*
GooF	*1.017*	*0.975*
R1	*0.0543*	*0.0527*
R1 (all)	*0.0743*	*0.0768*
wR2	*0.1484*	*0.1497*
wR2 (all)	*0.1595*	*0.1614*
weight factors	*0.0913*<br>*1.4673*	*0.1083*<br>*0*

4 Computational Details

All computations wer performed using Gaussian16[42] utilizing the PBE1PBE level of theory, Def2SVP basis sets and empirical dispersion correction (GD3). No solvent corrections were applied. All optimized molecular structures where checked to be minima on the energy hypersurface and possess no imaginary vibrational frequencies. Natural Bond Orbital Theory was applied to study the electronic states.[43] The SambVca server[44] was used to investigate buried volume[45] and steric maps.[46] Cone angles were calculated with Solid-G.[47] It is interesting to note that by utilising the NBO formalism,[43] the species $[RSi]^+$ is described as donor-acceptor compound with $[R]^- \rightarrow [Si]^{2+}$ and the Wiberg bond index (WBI) for the Si–N bond amounts to only 0.6573, indicating rather low covalency.

Table S4: Overview table, energies, NMR data.

	^{15}N	$\delta(^{15}N)$	^{29}Si	$\delta(^{29}Si)$	G [a.u.]
$SiMe_4$	–	–	407	0	
NH_3	270	0	–	–	
RSi^+	43	+227	354	+53	-2207.089360
RSiH	129	+141	287	+120	-2207.868345
RSiF	98	+172	350	+57	-2307.027861
RSiCl	107	+163	290	+117	-2667.260453
RSiBr	109	+161	270	+137	-4780.902478
RSiI	113	+157	238	+169	-2505.064687
R_2Si	86, 87	+183, +184	244	+163	-4125.326107
$RSiI_3$	130	+140	336	+72	-3100.601070
$RSiBr_3$	134	+136	370	+36	-9928.123486
$RSiCl_3$	139	+131	398	+9	-3587.205451
RSiI-II	138	+132	419	–8	-2505.104305
$RSi(NH_2{}^tBu)^+$	122, 165	+105, +148	355	+52	-2420.396296
$RSi(H)(NH^tBu)^+$	150, 185	+85, +120	435	–28	-2420.412581
$RSi(H)(NH^tBu)(NH_2{}^tBu)^+$	152, 185, 186	+84,+85, +118	443	–36	-2633.731465

4.1 Isomerisation of **2X** and [RSi]$^+$

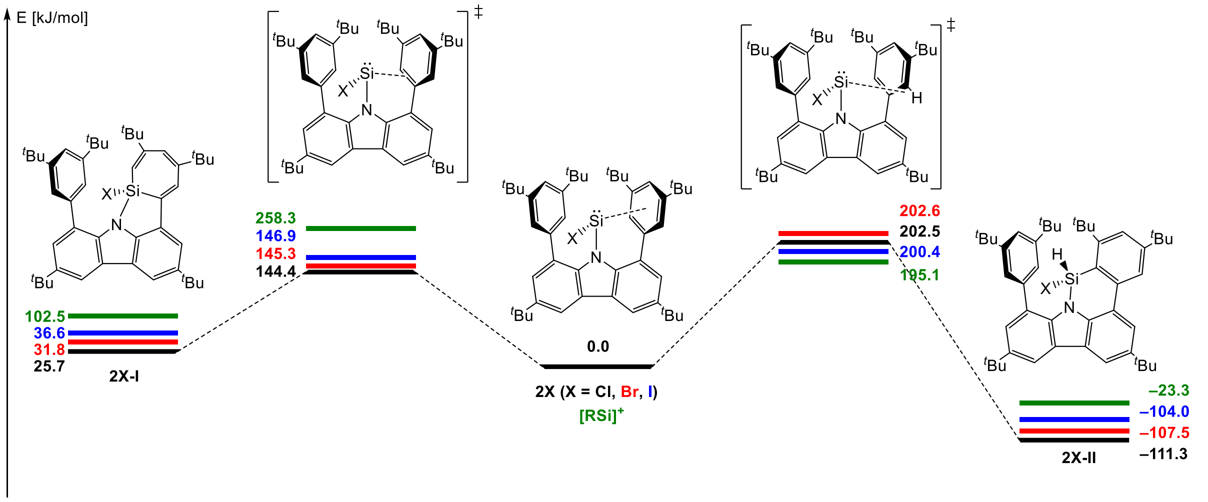

Figure S45: Computed energy profiles for the insertion of the Si atom into C-C and C-H bonds of **2X** and [RSi]$^+$ (energy not to scale).

Table S5: Computed energies for the insertion of the Si atom into C-C and C-H bonds of **2X** and [RSi]$^+$.

		G [a.u.]	ΔG [a.u.]	ΔG [kJ/mol]
RSiCl		-2667.260453	0	0.0
	TS	-2667.183335	0.077118	202.5
RSiCl-II	CH	-2667.302833	-0.042380	-111.3
	TS	-2667.205447	0.055006	144.4
RSiCl-I	CC	-2667.250670	0.009783	25.7
RSiBr		-4780.902478	0	0.0
	TS	-4780.825307	0.077171	202.6
RSiBr-II	CH	-4780.943407	-0.040930	-107.5
	TS	-4780.847145	0.055333	145.3
RSiBr-I	CC	-4780.890357	0.012121	31.8
RSiI		-2505.064687	0	0.0
	TS	-2504.988344	0.076343	200.4
RSiI-II	CH	-2505.104305	-0.039620	-104.0
	TS	-2505.008724	0.055963	146.9
RSiI-I	CC	-2505.050739	0.013948	36.6
RSi$^+$		-2207.089360	0	0.0
	TS	-2207.015033	0.074327	195.1
RSi$^+$-II	CH	-2207.098222	-0.008860	-23.3
	TS	-2206.990968	0.098392	258.3
RSi$^+$-I	CC	-2207.050320	0.039040	102.5

4.2 Influence of arene on ^{29}Si NMR of $[RSi]^+$

Table S6: Computed data for removal of arenes on $[RSi]^+$.

	no arene	*one arenes*	*two arenes*
	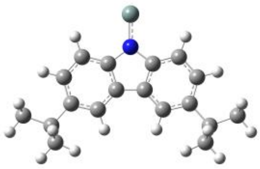	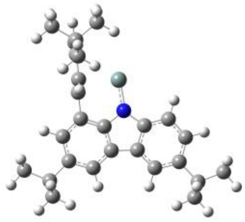	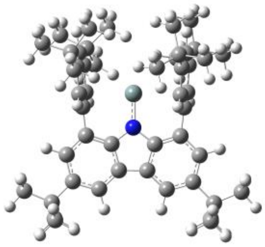
^{29}Si	*+604*	*+75*	*+53*
^{14}N	*+245*	*+231*	*+227*

4.3 F⁻/H⁻ affinities

Table S7: Fluoride and hydride affinities.

	**adduct**	ΣG [a.u.]	G_{adduct} [a.u.]	ΔG [a.u.]	ΔG [kJ/mol]
RSi^+		-2207.089360			
**FIA**	F^-	-99.609113			
RSiI	$RSiFI^-$	-2604.673806	-2604.870891	-0.197085	-517.4
RSi^+	RSiF	-2306.698473	-2307.027861	-0.329388	-864.8
$RSi(H)NH_2^+$	$RSi(H)(F)NH_2$	-2363.175626	-2363.512564	-0.336938	-884.6
$RSi(H)NH^tBu^+$	$RSi(H)(F)NH^tBu$	-2520.021694	-2520.365008	-0.343314	-901.4
Cp^*Si^+	Cp*SiF	-777.853934	-778.176414	-0.32248	-846.7
**HIA**	H^-	-0.489898			
RSiI	$RSiHI^-$	-2505.554591	-2505.714683	-0.160092	-420.3
RSi^+	RSiH	-2207.579258	-2207.868345	-0.289087	-759.0
$RSi(H)NH_2^+$	$RSi(H)(H)NH_2$	-2264.056411	-2264.362326	-0.305915	-803.2
$RSi(H)NH^tBu^+$	$RSi(H)(H)NH^tBu$	-2420.902479	-2421.215562	-0.313083	-822.0
Cp^*Si^+	Cp*SiH	-678.734719	-679.009487	-0.274768	-721.4

4.4 Reaction pathway of **3** with $^{t}BuNH_2$

Table S8: Activation of NH_3 by **3^M**.

	G [a.u.]	ΔG [a.u.]	ΔG [kJ/mol]
$RSi(NH_3)^+$	-1322.394361	0	0
TS	-1322.315733	0.078628	**206.4**
$RSi(H)NH_2^+$	-1322.416725	-0.02236	**-58.7**

Table S9: Activation of $^{t}BuNH_2$ by **3**.

	G [a.u.]	ΣG [a.u.]	ΔG [a.u.]	ΔG [kJ/mol]	ΔΔG [kJ/mol]
$^{t}BuNH_2$	-213.275656				
RSi^+	-2207.08936				
$RSi^+ + 2\ ^{t}BuNH_2$		-2633.64			
$RSi(^{t}BuNH_2)^+ + ^{t}BuNH_2$	-2420.396296	-2633.67	-0.03128	**-82.1**	**-82.1**
$RSi(H)NH^{t}Bu^+ + ^{t}BuNH_2$	-2420.412581	-2633.69	-0.01629	**-124.9**	**-42.8**
$RSi(H)(NH^{t}Bu)(NH_2{}^{t}Bu)^+$	-2633.731465	-2633.73	-0.04323	**-238.4**	**-113.5**

4.5 Orbital depictions of $[RSi]^+$

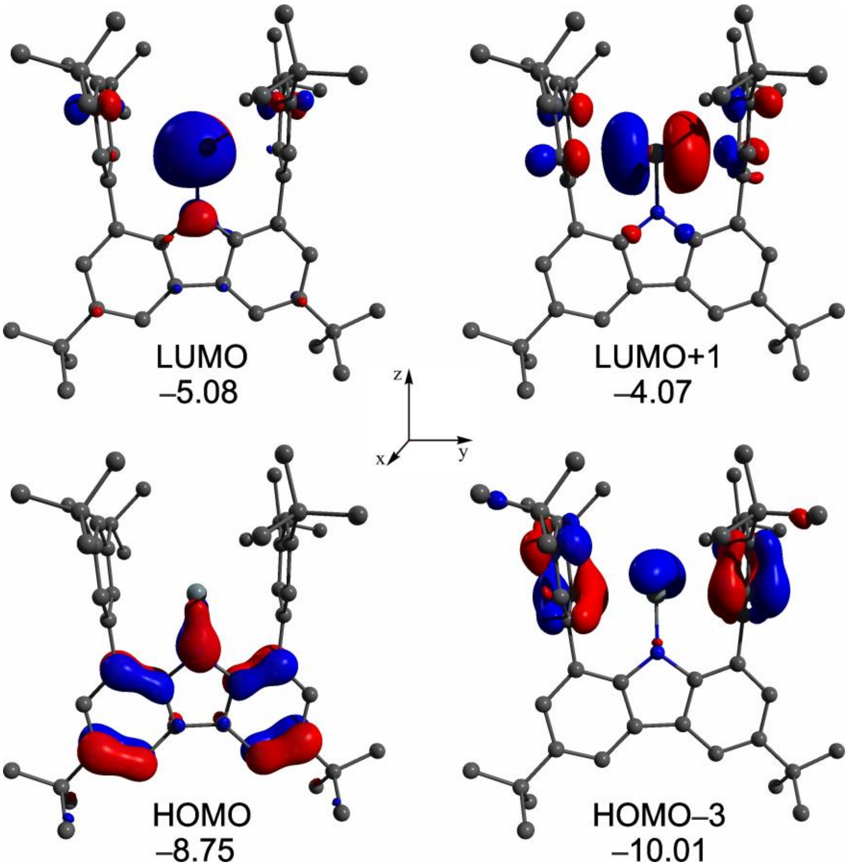

Figure S46: Orbital depictions of $[RSi]^+$ (isovalue 0.05, energies in eV).

4.6 Isodesmic Reactions

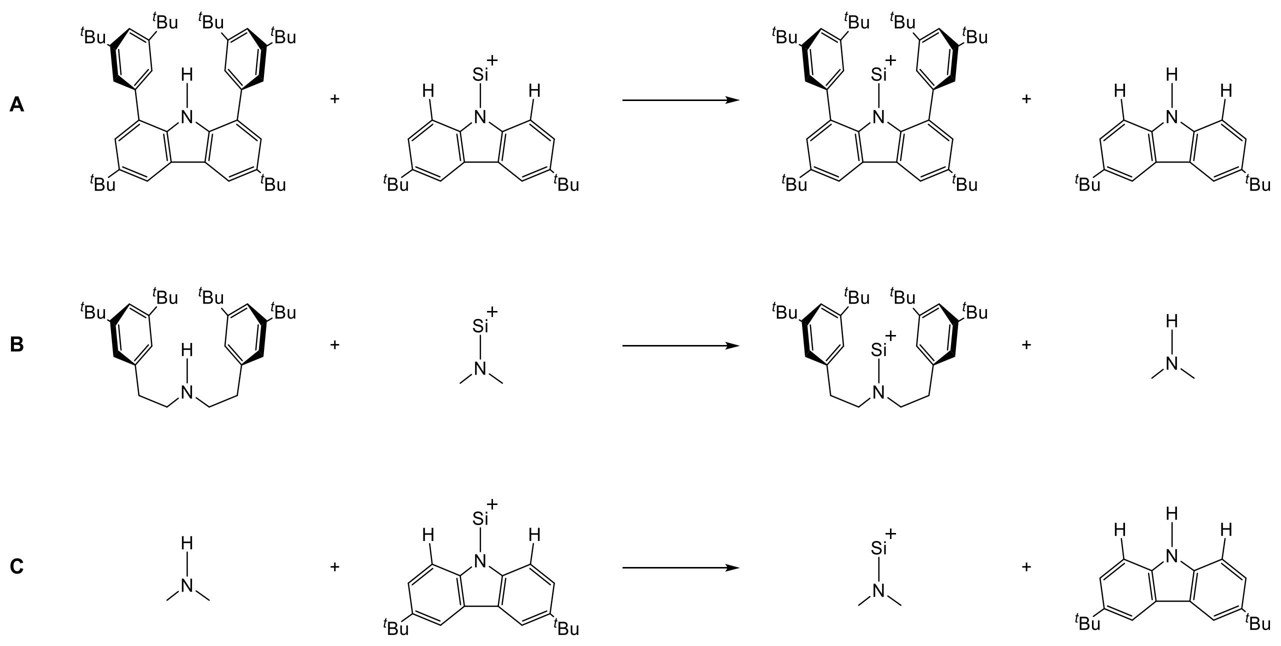

Figure S47: Isodesmic reactions.

Table S10: Data for isodesmic reactions.

G [a.u.]	1	2	3	4	**Δ_RG [a.u.]**	**Δ_RG [kJ/mol]**
**A**	-1918.617233	-1118.482503	-2207.089360	-830.079379	-0.069003	-181.2
**B**	-1302.481736	-423.247495	-1590.963892	-134.832191	-0.066851	-175.5
**C**	-134.832191	-1118.482503	-423.247495	-830.079379	-0.012180	-32.0

The stabilization by the arene substituents can be estimated by the above isodesmic reactions. On the carbazole scaffold, the arenes contribute -181.2 kJ/mol. Transition to a model with similar arene stabilization but a less rigid alkyl backbone provides similar stabilization energy of -175.5 kJ/mol. Due to delocalization of the N lone pair on the carbazole scaffold, the π-donating ability of the carbazolyl moiety is diminished compared to amide substituents by -32.0 kJ/mol.

4.7 Optimized structures

4.7.1 $RSiCl_3$

```
0 1
 Si                 -0.11970700   -0.29313000   -1.61194500
 N                   0.00954600    0.77255500   -0.21402000
 C                  -1.08554100    1.64368200    0.05946400
 C                  -2.44996700    1.35740500    0.16426000
 C                  -3.31464800    2.44613400    0.34993300
 H                  -4.37609100    2.21042300    0.43064500
 C                  -2.88071400    3.77349900    0.48213000
 C                  -1.50282500    4.01250700    0.45421800
 H                  -1.10727000    5.02078200    0.59874400
 C                  -0.61800400    2.95900800    0.24716300
 C                   0.82764100    2.89799300    0.22489300
 C                   1.79129500    3.88203200    0.41768700
 H                   1.47307900    4.91613900    0.56965800
 C                   3.14574000    3.53694800    0.43182800
 C                   3.46840100    2.17945100    0.30785100
 H                   4.50805800    1.86704100    0.40504500
 C                   2.52508600    1.15245700    0.12822000
 C                   1.18471800    1.54734100    0.02019500
 C                  -3.01583300   -0.01582100    0.18257800
 C                  -2.72982300   -0.86787400    1.25480900
 H                  -2.05007500   -0.51054000    2.02892700
 C                  -3.32028900   -2.13157100    1.34231600
 C                  -4.16252500   -2.52914900    0.29745100
 H                  -4.61282200   -3.52341300    0.33930700
 C                  -4.45370500   -1.71219800   -0.79975200
 C                  -3.88937800   -0.43533400   -0.82345700
 H                  -4.09085200    0.25019700   -1.64533500
 C                  -3.11004600   -3.06702500    2.53721700
 C                  -2.04619000   -2.53754700    3.50093000
 H                  -2.33669500   -1.57155800    3.94082300
 H                  -1.07304800   -2.41106600    3.00510800
 H                  -1.90592600   -3.24792100    4.33016900
 C                  -2.67553000   -4.45465800    2.04144100
 H                  -3.45627700   -4.93857000    1.43659900
 H                  -2.46427200   -5.11781300    2.89547900
 H                  -1.76798800   -4.38689800    1.42515200
 C                  -4.43818300   -3.19177000    3.30074400
 H                  -5.23660800   -3.59508700    2.65946500
 H                  -4.77099400   -2.21023100    3.67217500
 H                  -4.32464700   -3.86618500    4.16508700
 C                  -5.32341700   -2.24869600   -1.94003800
 C                  -4.59937700   -3.44572000   -2.57746800
 H                  -5.18488100   -3.84754700   -3.42017300
 H                  -4.44677000   -4.26081200   -1.85400100
 H                  -3.60947400   -3.14569000   -2.95457400
 C                  -6.68638100   -2.69653500   -1.39308600
 H                  -7.31335500   -3.09758900   -2.20560400
 H                  -7.22180200   -1.85168100   -0.93270100
 H                  -6.58719400   -3.48492300   -0.63192200
 C                  -5.55877700   -1.19594000   -3.02514500
 H                  -6.18591700   -1.61837000   -3.82522100
 H                  -4.61381500   -0.86566200   -3.48272900
```

H	-6.07763400	-0.30969800	-2.62827400
C	-3.85122000	4.94054200	0.69354400
C	-5.31263100	4.48747100	0.68010000
H	-5.58481400	4.01404100	-0.27576400
H	-5.97261000	5.35730400	0.82037000
H	-5.52853700	3.77498800	1.49086200
C	-3.65498500	5.96832300	-0.43168300
H	-3.85422100	5.51593400	-1.41538100
H	-2.62958100	6.36683100	-0.44861700
H	-4.34201500	6.81964500	-0.29939300
C	-3.56234700	5.60107100	2.05033900
H	-4.24712300	6.44752800	2.22026900
H	-2.53360900	5.98715100	2.10648400
H	-3.69512300	4.88153200	2.87297200
C	4.20903700	4.62280700	0.62502000
C	4.08016800	5.65570600	-0.50514800
H	4.22958200	5.18274100	-1.48800200
H	4.83399300	6.45088300	-0.38809800
H	3.08922800	6.13378300	-0.51187600
C	3.99184300	5.31264100	1.98056300
H	2.99919000	5.78271400	2.04618900
H	4.74567500	6.10099600	2.13738200
H	4.07469500	4.58902000	2.80613000
C	5.62961100	4.05428900	0.59565500
H	5.80245800	3.33762200	1.41312200
H	6.35887100	4.87052100	0.71288000
H	5.84707800	3.54835700	-0.35771800
C	3.01326400	-0.24729100	0.17180700
C	4.15701300	-0.60290000	-0.55972900
H	4.58880200	0.14275600	-1.22722900
C	4.70546200	-1.87756200	-0.47114000
C	4.10286400	-2.78902900	0.41099900
H	4.54017800	-3.78298100	0.50543200
C	2.97220600	-2.47289600	1.16338400
C	2.41594300	-1.19301100	1.00523500
H	1.52483300	-0.90376400	1.56659800
C	5.91020600	-2.31983200	-1.30611100
C	6.46473600	-1.18154300	-2.16514600
H	5.71946800	-0.81150400	-2.88582600
H	7.33275800	-1.53768500	-2.74098800
H	6.79984800	-0.33204900	-1.55007200
C	7.03096000	-2.81445800	-0.37970100
H	6.70903300	-3.66389800	0.24083700
H	7.36359100	-2.01203300	0.29683700
H	7.89920200	-3.14606000	-0.97152300
C	5.46762400	-3.45942400	-2.23781200
H	5.10355900	-4.32941000	-1.67087800
H	6.30843300	-3.79480800	-2.86636300
H	4.65251500	-3.12774200	-2.89954800
C	2.33669200	-3.47660300	2.13088300
C	3.23602800	-4.69084200	2.37723500
H	2.76232400	-5.35910300	3.11274100
H	4.21940900	-4.39538900	2.77488300
H	3.39634700	-5.27903500	1.46107400
C	1.01374900	-3.96422200	1.52879700
H	0.51498500	-4.67271300	2.20925500

H	1.18237000	-4.46938700	0.56528200
H	0.32761400	-3.12681800	1.34178500
C	2.06787000	-2.80299200	3.48482400
H	1.37221200	-1.95618900	3.39648900
H	3.00118000	-2.42563700	3.93064300
H	1.61837100	-3.52391500	4.18609700
Cl	1.62482400	-0.38394600	-2.67044200
Cl	-1.49173700	0.46326200	-2.94191800
Cl	-0.66971900	-2.19783400	-1.13138300

4.7.2 $RSiBr_3$

0 1

Br	-1.83196200	-0.41413300	-2.49617000
Br	0.72223000	-2.24944500	-0.79303500
Br	1.46502300	0.63640000	-2.83933200
Si	0.04810200	-0.22656300	-1.35601500
N	-0.08131300	0.85357000	0.04481700
C	-1.26437600	1.61711100	0.28995200
C	-2.59868900	1.20947400	0.44313000
C	-3.54670800	2.22962200	0.63953800
H	-4.57887100	1.90875400	0.77721400
C	-3.23716000	3.59205900	0.73179000
C	-1.88820200	3.95221000	0.66717600
H	-1.57732400	4.99199900	0.79322700
C	-0.92018500	2.97548000	0.46178300
C	0.52437100	3.05631400	0.45318600
C	1.39145500	4.13057300	0.62055300
H	0.97844400	5.13470500	0.74272900
C	2.77339800	3.91740100	0.63570300
C	3.22643500	2.59404700	0.53884000
H	4.29247700	2.37827900	0.61494900
C	2.38045600	1.48381700	0.39302600
C	1.00870600	1.74291100	0.29027500
C	-3.06567200	-0.19583400	0.49713400
C	-2.36338500	-1.15931900	1.22737500
H	-1.44495300	-0.85891600	1.73158200
C	-2.84020500	-2.47118400	1.33109800
C	-4.01836100	-2.79628000	0.65305400
H	-4.38874500	-3.82246200	0.71129000
C	-4.74382500	-1.86172700	-0.09560500
C	-4.25583500	-0.55723300	-0.14760700
H	-4.77293500	0.20178400	-0.73444300
C	-2.13382100	-3.54386300	2.16453400
C	-0.89790000	-2.98704800	2.86800100
H	-1.15572900	-2.17392600	3.56419200
H	-0.16393600	-2.60027200	2.14580700
H	-0.40676100	-3.78023100	3.45160700
C	-1.69720000	-4.69669100	1.24750700
H	-2.55686300	-5.16923100	0.74896400
H	-1.17724800	-5.47521600	1.82897000
H	-1.01351800	-4.33632300	0.46436500
C	-3.09944700	-4.07497100	3.23502100
H	-3.99422300	-4.53546200	2.79033200
H	-3.43470500	-3.26298200	3.89874800

H	-2.60240800	-4.84022600	3.85282600
C	-6.01001800	-2.30115100	-0.83659000
C	-5.63573400	-3.38380200	-1.86099600
H	-6.52792400	-3.71483600	-2.41672500
H	-5.19468200	-4.26821300	-1.37742300
H	-4.90121900	-2.99934600	-2.58551900
C	-7.02131600	-2.86910900	0.17066000
H	-7.93770800	-3.19593000	-0.34669300
H	-7.30108700	-2.10913200	0.91659600
H	-6.61758600	-3.73714100	0.71276300
C	-6.67322500	-1.14110100	-1.58205800
H	-7.57995000	-1.49716300	-2.09481400
H	-6.00747200	-0.71230000	-2.34675200
H	-6.97452700	-0.33383600	-0.89667500
C	-4.30661000	4.66840500	0.94309500
C	-5.71952900	4.08192100	0.98009600
H	-5.96814400	3.55671700	0.04494100
H	-6.45445100	4.89072400	1.11239600
H	-5.84996200	3.37755700	1.81594200
C	-4.23372900	5.67808600	-0.21286400
H	-4.41594300	5.18246600	-1.17890300
H	-3.24908700	6.16593900	-0.26836600
H	-4.99131300	6.46779500	-0.08340000
C	-4.04734300	5.39026500	2.27437500
H	-4.80585300	6.17156700	2.44344100
H	-3.05987800	5.87527300	2.29151300
H	-4.08814100	4.68391500	3.11785200
C	3.72696000	5.10631200	0.79125900
C	3.50277000	6.08282900	-0.37381800
H	3.70041600	5.59287700	-1.33970500
H	4.17595300	6.95067800	-0.28440200
H	2.47019500	6.46216500	-0.39684600
C	3.44064700	5.81848500	2.12225200
H	2.40803000	6.19520800	2.17150500
H	4.11635800	6.67927300	2.25186700
H	3.58867000	5.13499300	2.97255400
C	5.19522900	4.67572300	0.78134800
H	5.43028000	4.00046800	1.61827900
H	5.84285300	5.56057000	0.87852900
H	5.46531600	4.16756900	-0.15710200
C	2.99285100	0.12971200	0.45149400
C	3.84805600	-0.30303200	-0.56488600
H	4.00286700	0.35421100	-1.41873800
C	4.45900600	-1.55739400	-0.50885000
C	4.24177800	-2.33174000	0.63473800
H	4.72477900	-3.30949000	0.70211000
C	3.44307000	-1.90654200	1.70301900
C	2.79516900	-0.67394600	1.58025100
H	2.14211500	-0.30178700	2.37032700
C	5.31265800	-2.10935300	-1.65382700
C	5.47138800	-1.09675200	-2.79007500
H	4.50025100	-0.82254800	-3.22977100
H	6.08909300	-1.53000600	-3.59171400
H	5.96776900	-0.17505000	-2.44895700
C	6.70931400	-2.47804000	-1.13297800
H	6.66587400	-3.24061700	-0.34108900

H	7.22022000	-1.59445200	-0.71991300
H	7.32942600	-2.88207600	-1.94925700
C	4.61976100	-3.36100800	-2.21603300
H	4.51497100	-4.14586700	-1.45169400
H	5.20049100	-3.78139400	-3.05302100
H	3.61126900	-3.11565100	-2.58346500
C	3.32927100	-2.79099500	2.94890400
C	4.73334100	-3.02778800	3.52808100
H	4.67393500	-3.65712000	4.43070700
H	5.20896700	-2.07441400	3.80565700
H	5.39493500	-3.53573300	2.81074600
C	2.70587700	-4.13997100	2.55798300
H	2.59610400	-4.78614700	3.44398200
H	3.32922200	-4.67824800	1.82823900
H	1.71156500	-4.00185400	2.10979100
C	2.47471100	-2.14052200	4.03844600
H	1.44786200	-1.94799500	3.69934400
H	2.90891500	-1.18627700	4.37421100
H	2.41486900	-2.80567500	4.91364000

4.7.3 $RSiI_3$

0 1

I	-2.03434200	-0.12442600	-2.54220000
I	0.74548700	-2.35693700	-0.82139100
I	1.59498300	1.06529600	-2.76244900
Si	0.02727700	-0.06386100	-1.22032700
N	-0.09461800	0.86625600	0.30257900
C	-1.27156200	1.63116800	0.57673300
C	-2.60659400	1.22439000	0.72307300
C	-3.54755900	2.24471100	0.95469400
H	-4.58096000	1.92714000	1.08915900
C	-3.23020500	3.60163900	1.08644800
C	-1.87896600	3.95653900	1.03174900
H	-1.56180400	4.98956600	1.19273000
C	-0.91845300	2.98055700	0.79430500
C	0.52737200	3.04838000	0.79795300
C	1.40068500	4.10952700	1.00504600
H	0.99505600	5.11330900	1.15201900
C	2.78089200	3.88330900	1.02658100
C	3.22234500	2.55953300	0.89863600
H	4.28552500	2.33232300	0.98015300
C	2.36879500	1.46050200	0.71036100
C	1.00080200	1.73527800	0.59884700
C	-3.08563300	-0.17870700	0.74515300
C	-2.38292600	-1.17222100	1.43442800
H	-1.44236400	-0.90271000	1.91466600
C	-2.89276900	-2.47231000	1.53691600
C	-4.09700100	-2.75882300	0.88820200
H	-4.49240600	-3.77566500	0.94557600
C	-4.81975000	-1.79633900	0.17350000
C	-4.30242200	-0.50341900	0.13008100
H	-4.82107700	0.27749000	-0.42554000
C	-2.20430900	-3.57083900	2.35189400
C	-0.92872300	-3.06160000	3.01874600

H	-1.13685000	-2.24140300	3.72317900
H	-0.20226200	-2.69906500	2.27651400
H	-0.44996700	-3.87327600	3.58703000
C	-1.84128600	-4.74526400	1.43077600
H	-2.73267300	-5.18558000	0.95933400
H	-1.33709300	-5.54110800	2.00253000
H	-1.16621700	-4.41742300	0.62623700
C	-3.16028400	-4.05798400	3.45200500
H	-4.08483400	-4.48384700	3.03450500
H	-3.44425300	-3.23006900	4.11996500
H	-2.67718800	-4.83970800	4.06025400
C	-6.11596000	-2.19298500	-0.53903900
C	-5.79584700	-3.26541300	-1.59211800
H	-6.71121200	-3.56805300	-2.12595600
H	-5.35956000	-4.16694900	-1.13666000
H	-5.07512800	-2.88437100	-2.33217400
C	-7.11248100	-2.75540300	0.48586000
H	-8.05037400	-3.05092500	-0.01138400
H	-7.35313400	-2.00303400	1.25285600
H	-6.71510000	-3.64274200	1.00071100
C	-6.77178000	-1.00462700	-1.24549900
H	-7.69975200	-1.33044900	-1.74003500
H	-6.11678100	-0.57696900	-2.02015500
H	-7.03602400	-0.20330300	-0.53817000
C	-4.29341800	4.67665800	1.33219500
C	-5.70972500	4.09751800	1.35096500
H	-5.96088200	3.60249600	0.40014700
H	-6.43993400	4.90621600	1.50768200
H	-5.84474300	3.36887300	2.16499100
C	-4.21449400	5.72153200	0.20831300
H	-4.39931900	5.25735200	-0.77271900
H	-3.22705700	6.20519400	0.16803200
H	-4.96753700	6.51112200	0.36251700
C	-4.02979700	5.35472600	2.68547800
H	-4.78383800	6.13460200	2.87924200
H	-3.03961100	5.83338400	2.71761600
H	-4.07459900	4.62240400	3.50630700
C	3.74372300	5.05892600	1.21881900
C	3.54015500	6.06156500	0.07219700
H	3.74111200	5.58912500	-0.90170600
H	4.22145800	6.92023800	0.18544700
H	2.51171400	6.45189700	0.04789400
C	3.45013100	5.74515600	2.56174600
H	2.42069300	6.13072600	2.60873400
H	4.13240100	6.59654700	2.71643900
H	3.58319400	5.04244000	3.39875200
C	5.20788000	4.61454600	1.21451000
H	5.42751000	3.91802100	2.03810700
H	5.86265500	5.49068200	1.33879500
H	5.48323400	4.12549700	0.26748500
C	2.98111700	0.10521300	0.73618400
C	3.88558700	-0.27727300	-0.25819500
H	4.06513200	0.41287300	-1.08057400
C	4.52030400	-1.52046800	-0.21870200
C	4.27279700	-2.33733200	0.88856400
H	4.77512000	-3.30601100	0.94480700

C	3.42433600	-1.96283200	1.93747800
C	2.75481100	-0.74032500	1.82864000
H	2.06714000	-0.40568600	2.60597300
C	5.44357300	-2.01007400	-1.33787900
C	5.61297300	-0.96200400	-2.43988900
H	4.65117200	-0.70731400	-2.91106600
H	6.27673100	-1.35234100	-3.22663200
H	6.06385300	-0.03416600	-2.05499500
C	6.82859300	-2.33782400	-0.76097400
H	6.77880700	-3.12258700	0.00863700
H	7.28358800	-1.44645700	-0.30183400
H	7.50169300	-2.69489000	-1.55695700
C	4.82969500	-3.27265700	-1.96325200
H	4.71699100	-4.08010700	-1.22391900
H	5.46871000	-3.64975000	-2.77803800
H	3.83290700	-3.05682800	-2.37816400
C	3.28403800	-2.88837800	3.15054200
C	4.67409600	-3.14599100	3.75422100
H	4.59172400	-3.80080400	4.63661500
H	5.14541800	-2.20257600	4.07042100
H	5.35155100	-3.63587400	3.03926700
C	2.67024500	-4.22301300	2.69987600
H	2.54557800	-4.90158400	3.55932400
H	3.30845200	-4.73235300	1.96205600
H	1.68368200	-4.06976300	2.23930400
C	2.40660000	-2.27328800	4.24235500
H	1.38685000	-2.07026600	3.88937800
H	2.83339300	-1.32983800	4.61607300
H	2.32954700	-2.96573600	5.09474000

4.7.4 RSiCl

0 1

C	1.30434800	1.53005000	-0.23165100
N	0.10710900	0.82767200	-0.12334600
C	2.14893100	3.81341000	-0.26191100
C	1.07547000	2.92496200	-0.20425100
C	-0.35553000	3.09271500	-0.08789300
C	-1.18973100	4.20764500	-0.01946600
C	-2.57388700	4.05073200	0.10275700
C	-3.08681400	2.74116000	0.16278900
C	-2.28122000	1.60152900	0.10108200
C	-0.90186200	1.78770500	-0.03662800
C	2.82110800	-0.43354100	-0.41921900
C	2.06421300	-1.19984000	-1.33222900
C	2.20910800	-2.59845900	-1.39651200
C	3.06315500	-3.20619900	-0.47209400
C	3.81323700	-2.47484600	0.45660900
C	3.69467000	-1.07965600	0.44962400
C	4.71004500	-3.14648000	1.49806500
C	1.46158900	-3.45390300	-2.42309800
C	4.62838700	4.32902400	-0.39102300
C	-3.47490100	5.28974700	0.17109000
C	-2.85468600	0.23282400	0.17708800
C	-2.91041300	-0.44346400	1.40365300

C	-3.43585600	-1.73993900	1.49201200
C	-3.87236600	-2.34593900	0.30897100
C	-3.83327700	-1.70087200	-0.93404100
C	-3.32763800	-0.39914200	-0.97803200
C	-3.55951500	-2.42339100	2.85779500
C	-4.30869000	-2.37604900	-2.22441200
C	3.46151700	3.33516100	-0.34136400
C	3.65703800	1.94262800	-0.38254800
C	2.60466800	1.02288500	-0.34638400
C	4.48203300	5.22468400	-1.63087600
H	3.54012400	5.79312000	-1.61472200
H	5.30988400	5.95039300	-1.68459500
H	4.49278700	4.62242000	-2.55249100
C	4.60839500	5.19860600	0.87569500
H	5.43869900	5.92330600	0.86075700
H	3.67093900	5.76722800	0.96651400
H	4.71049900	4.57743400	1.77894900
C	5.98557500	3.62581200	-0.46586800
H	6.16058000	2.98107400	0.40924800
H	6.07772900	3.00832300	-1.37259600
H	6.79215700	4.37466900	-0.49232000
C	4.76693800	-4.66544900	1.32334400
H	5.16110800	-4.95049900	0.33544500
H	5.43212600	-5.10098900	2.08436400
H	3.77672100	-5.12945100	1.44878100
C	4.14536100	-2.83790900	2.89485200
H	4.13061200	-1.75733500	3.09904300
H	3.11097400	-3.20149300	2.98793200
H	4.75990400	-3.32263300	3.67046200
C	6.13753500	-2.59146600	1.37931600
H	6.79168400	-3.06035000	2.13145100
H	6.55935300	-2.79685000	0.38314300
H	6.17201100	-1.50428600	1.54218100
C	-3.07945000	6.14025800	1.38819000
H	-2.03276400	6.47444100	1.33047500
H	-3.71406900	7.03893900	1.45534200
H	-3.19579100	5.56697700	2.32090200
C	-4.95478100	4.92340700	0.30430800
H	-5.30852300	4.33119700	-0.55366400
H	-5.15347000	4.35037100	1.22302500
H	-5.56299900	5.84011200	0.34761600
C	-3.29878200	6.11712900	-1.11167400
H	-3.93529100	7.01655400	-1.08314800
H	-2.25803800	6.44863700	-1.24408600
H	-3.57679900	5.52770900	-1.99910900
C	-5.39314400	-1.51109400	-2.88438400
H	-5.02036200	-0.50859300	-3.14097500
H	-5.74560300	-1.98321600	-3.81543000
H	-6.25813700	-1.38647000	-2.21467800
C	-4.89215200	-3.76782800	-1.97086800
H	-5.76166300	-3.73117100	-1.29651500
H	-5.22800300	-4.20824600	-2.92215800
H	-4.14699800	-4.45169900	-1.53624300
C	2.47751300	-4.28034900	-3.22658200
H	3.05343600	-4.96401700	-2.58553800
H	1.95620000	-4.89110700	-3.98080200

H	3.19218500	-3.62653500	-3.75010100
C	0.49684900	-4.39626300	-1.68424600
H	1.03033800	-5.06273600	-0.98999700
H	-0.24266900	-3.82257900	-1.10409000
H	-0.04559800	-5.02718300	-2.40671400
C	0.65562200	-2.59789200	-3.40245800
H	-0.10884900	-1.99913200	-2.88527400
H	1.30284500	-1.91888400	-3.97894700
H	0.13204700	-3.24751500	-4.12030200
C	-3.11173200	-2.52188400	-3.17591600
H	-2.66434500	-1.54721500	-3.42152900
H	-2.32601600	-3.14314200	-2.72053900
H	-3.42373900	-2.99703100	-4.12009700
C	-4.10753200	-3.84760100	2.74339800
H	-4.17121400	-4.30048800	3.74456600
H	-5.11841600	-3.86868200	2.30769600
H	-3.45304500	-4.48745100	2.13143800
C	-4.52407700	-1.60030000	3.72714000
H	-5.51869600	-1.53402300	3.25920900
H	-4.64085700	-2.06782100	4.71815200
H	-4.15620600	-0.57496400	3.88167500
C	-2.18591400	-2.49033600	3.53769200
H	-1.46961000	-3.06609400	2.93337200
H	-1.74649000	-1.49421200	3.68859700
H	-2.27497200	-2.97175400	4.52479300
H	4.66513300	1.53625200	-0.47294800
H	1.95165200	4.88808600	-0.23467300
H	-0.74638000	5.20580800	-0.06195500
H	-4.16031800	2.57989100	0.26671200
H	-3.27194900	0.14670300	-1.92264700
H	-4.26937000	-3.35702400	0.35862100
H	-2.54900200	0.07215400	2.29649300
H	4.24045600	-0.47375000	1.17544500
H	3.14866900	-4.29227500	-0.48321100
H	1.48717500	-0.66878000	-2.09244400
Si	-0.14337000	-0.99106100	0.03733300
Cl	0.73585400	-1.26205200	1.97728600

4.7.5 RSiCl-I

0 1

C	2.37861200	0.10382800	0.03233400
N	1.08308200	-0.32902500	-0.19251400
C	4.63213300	-0.60598000	0.31811800
C	3.29711500	-0.94716300	0.08428700
C	2.49823000	-2.14427300	-0.13246600
C	2.79850800	-3.50441600	-0.17359400
C	1.78029600	-4.45039600	-0.33861400
C	0.45338600	-3.99025800	-0.41986000
C	0.10398600	-2.63566400	-0.38281200
C	1.14706400	-1.71375400	-0.27876200
C	1.48076500	2.27066400	-0.01041500
C	1.29943100	3.59944800	0.18515700
C	0.06843200	4.33709200	-0.03679400
C	-1.20755400	3.88642300	0.18162300

C	-1.81532900	2.65751000	0.67982700
C	-1.39000300	1.39628100	0.39122100
C	-3.10485600	2.81477100	1.52686200
C	0.20172100	5.79100700	-0.56304300
C	6.48621200	1.06442600	0.74651000
C	2.13589800	-5.94114000	-0.39769400
C	-1.32346100	-2.22146500	-0.28563600
C	-2.04844100	-1.71144400	-1.36421300
C	-3.40880900	-1.38726300	-1.22951000
C	-4.00173700	-1.55970500	0.02430000
C	-3.29927600	-2.04502000	1.13705000
C	-1.95546700	-2.37573700	0.95649900
C	-4.20727100	-0.88798500	-2.43953200
C	-4.00670400	-2.29301600	2.47417700
C	5.00647900	0.74213000	0.48898000
C	4.03065700	1.75809200	0.41276300
C	2.68488600	1.45780900	0.17233200
C	6.94425700	0.36130100	2.03393000
H	6.83695400	-0.73143100	1.96483400
H	8.00516300	0.57999700	2.23739600
H	6.35179900	0.70083500	2.89759200
C	7.33073500	0.56970700	-0.43823800
H	8.39724000	0.79323200	-0.27282600
H	7.23704400	-0.51678500	-0.58346400
H	7.01808700	1.05947300	-1.37338700
C	6.73416000	2.56564000	0.91205100
H	6.45630200	3.12915800	0.00807800
H	6.17497800	2.98150200	1.76432200
H	7.80389900	2.74708700	1.09826100
C	-3.42809200	4.27038400	1.88323200
H	-3.71210900	4.86757800	1.00336500
H	-4.28453100	4.29388100	2.57464700
H	-2.58095200	4.76934200	2.37828500
C	-2.91117800	2.05003200	2.84502200
H	-2.71225100	0.98330700	2.67354400
H	-2.06580500	2.46119200	3.41794300
H	-3.81732400	2.12685000	3.46701000
C	-4.29080800	2.22562400	0.75033600
H	-5.21985500	2.31616900	1.33646300
H	-4.43884000	2.75559700	-0.20389600
H	-4.13277300	1.16196100	0.52130100
C	3.07317500	-6.18894500	-1.58991900
H	4.00446300	-5.60928100	-1.50425600
H	3.34658800	-7.25485500	-1.65241600
H	2.58779000	-5.90276600	-2.53574900
C	0.89920900	-6.82600200	-0.56937100
H	0.19865700	-6.71908300	0.27309200
H	0.35696000	-6.59484100	-1.49904500
H	1.20191300	-7.88349300	-0.61565600
C	2.84207200	-6.35048300	0.90415700
H	3.10798700	-7.41978900	0.87989400
H	3.76941000	-5.78046800	1.06413900
H	2.18980900	-6.17866000	1.77439200
C	-4.55041100	-3.73120700	2.45694900
H	-3.73417100	-4.45846400	2.32744000
H	-5.07168000	-3.96254600	3.40036000

H	-5.26067700	-3.87315700	1.62777700
C	-5.17418100	-1.32060700	2.68488300
H	-5.97877100	-1.46627700	1.94928000
H	-5.61742300	-1.47873000	3.68010600
H	-4.84184400	-0.27347300	2.62296700
C	-0.47306000	5.85068900	-1.94380100
H	-1.54881100	5.62942600	-1.88337600
H	-0.35986900	6.85425300	-2.38479200
H	-0.02227300	5.11689000	-2.62983400
C	-0.46766500	6.78667400	0.39485600
H	-1.54308400	6.59092100	0.51543200
H	-0.00571400	6.74166400	1.39349800
H	-0.35904100	7.81523000	0.01523700
C	1.66090700	6.22511600	-0.74491000
H	2.20422800	6.27610200	0.21119200
H	2.21022600	5.55170900	-1.42024900
H	1.68758300	7.23329200	-1.18607500
C	-3.04076000	-2.13756500	3.65526500
H	-2.24117200	-2.89211500	3.64344400
H	-2.56819000	-1.14331100	3.66036100
H	-3.58529000	-2.26001300	4.60425500
C	-5.68096500	-0.64896200	-2.10143300
H	-6.21396400	-0.30438700	-3.00071000
H	-6.17909400	-1.56680800	-1.75246200
H	-5.80353700	0.12661200	-1.32952600
C	-4.13464700	-1.94169700	-3.55558800
H	-4.55721600	-2.90161600	-3.21996100
H	-4.70363800	-1.60525200	-4.43715900
H	-3.09898600	-2.12410800	-3.87795400
C	-3.60885800	0.43268300	-2.94104000
H	-3.61946900	1.19759700	-2.14942300
H	-2.56635000	0.31449100	-3.26349700
H	-4.18788900	0.81364600	-3.79778400
H	4.32225400	2.80251000	0.52631600
H	5.39167100	-1.39006100	0.36547500
H	3.83832900	-3.82245300	-0.06480500
H	-0.36597200	-4.70582900	-0.49635500
H	-1.36240400	-2.77218200	1.78180200
H	-5.05542600	-1.31640400	0.14241900
H	-1.54248400	-1.58652000	-2.32425900
H	-1.95010400	0.53625200	0.77493200
H	-1.96539400	4.65133400	-0.00208200
H	2.16705000	4.19221100	0.49302000
Si	0.12763000	1.11460300	-0.56393500
Cl	-0.11724400	1.23996900	-2.63170700

4.7.6 RSiCl-I-TS

0 1

C	-0.71567600	2.02729500	-0.15013400
N	0.18155800	0.96816800	-0.08642000
C	-0.92630600	4.39384500	-0.02561600
C	-0.10093000	3.26417100	0.03620500
C	1.30742700	2.95224900	0.21955500
C	2.44515700	3.74569700	0.39714000

C	3.70617800	3.15584300	0.48719800
C	3.79154800	1.75071800	0.39705200
C	2.68411900	0.91826500	0.23573700
C	1.43213000	1.53783500	0.14474900
C	-2.37145400	0.38313000	-0.53987900
C	-3.50321100	-0.17070300	-1.21708200
C	-4.00389000	-1.43132400	-1.01647800
C	-3.71216800	-2.15117400	0.19371800
C	-2.96240100	-1.70459500	1.25574000
C	-1.95903800	-0.68518200	1.09575500
C	-2.30515100	4.25646800	-0.26426500
C	-2.86541600	2.96916200	-0.44009900
C	-2.07233400	1.82246900	-0.38490700
H	-3.93515300	2.85429400	-0.61681400
H	-0.49414400	5.38794600	0.11155000
H	2.32594900	4.82814200	0.45216700
H	4.76859700	1.26565800	0.46468800
H	-1.73471300	-0.04561800	1.95799000
H	-4.34972100	-3.01873700	0.38106700
H	-3.98255600	0.49327900	-1.94087800
Si	-0.78004600	-0.49645400	-0.27850200
Cl	-0.13008900	-2.01507400	-1.46893000
C	2.82283100	-0.55218600	0.11715700
C	3.52659400	-1.09731100	-0.96041100
C	2.23507900	-1.40089400	1.06267900
C	3.64475700	-2.48057500	-1.11756100
H	3.95890200	-0.41017400	-1.68815400
C	2.34776200	-2.79061700	0.94768100
H	1.70946100	-0.94847400	1.90516100
C	3.05166900	-3.29753500	-0.14888900
H	3.13170100	-4.38135700	-0.26006200
C	4.99087900	3.96943800	0.67727900
C	4.71349600	5.47265500	0.74323400
H	4.24584900	5.84281600	-0.18213100
H	4.05681900	5.73009500	1.58849900
H	5.65843300	6.02088200	0.87920500
C	5.67148600	3.54696600	1.98861300
H	5.00883400	3.72676400	2.84928600
H	5.93506800	2.47879900	1.98720900
H	6.59980200	4.12031900	2.14403500
C	5.93993000	3.70637600	-0.50236300
H	5.47229800	4.00109600	-1.45456700
H	6.87164000	4.28300000	-0.38414100
H	6.21388200	2.64352400	-0.57881800
C	-3.17175300	5.52397000	-0.31972300
C	-4.64554000	5.20927100	-0.58743600
H	-4.78679700	4.69883600	-1.55251600
H	-5.08047800	4.57885700	0.20326200
H	-5.22492600	6.14478900	-0.62042800
C	-3.07882300	6.26530000	1.02307300
H	-2.04595000	6.56469300	1.25522700
H	-3.69498400	7.17894700	1.00393700
H	-3.43516700	5.62829000	1.84737100
C	-2.66719800	6.43773400	-1.44757700
H	-1.62213300	6.74369200	-1.29082600
H	-2.72272300	5.92576500	-2.42071200

H	-3.27787700	7.35338900	-1.50567700
C	-5.04101100	-2.04824000	-1.96419700
C	-3.28532400	-2.25822300	2.66323500
C	1.72393000	-3.76030500	1.95478400
C	4.35529300	-3.11906500	-2.31440200
C	-5.29978000	-1.16791800	-3.18860600
H	-5.99480700	-1.67701400	-3.87409600
H	-5.75874000	-0.20566900	-2.91363500
H	-4.37099600	-0.96120400	-3.74203600
C	-6.38073700	-2.26754700	-1.24404900
H	-6.78218900	-1.31350400	-0.86861100
H	-7.12109600	-2.70395300	-1.93373300
H	-6.28795300	-2.95147100	-0.38754200
C	-4.48780100	-3.39647300	-2.45371800
H	-4.31438100	-4.09346800	-1.61992400
H	-5.19414300	-3.87544100	-3.15114500
H	-3.52768800	-3.25658700	-2.97434900
C	-4.79087900	-2.13206900	2.94686200
H	-5.39447800	-2.71999800	2.24077100
H	-5.02251400	-2.49013200	3.96323400
H	-5.11662800	-1.08325700	2.86974100
C	-2.54543700	-1.50596200	3.77324200
H	-1.45355100	-1.55973700	3.65933600
H	-2.83708800	-0.44482000	3.81173000
H	-2.79583800	-1.94985200	4.74906700
C	-2.87661300	-3.73767400	2.71751200
H	-3.11245700	-4.16605200	3.70552700
H	-3.40786500	-4.33096800	1.95824500
H	-1.79862400	-3.85779100	2.54042900
C	1.00646500	-3.01827500	3.08149000
H	0.54927200	-3.73746800	3.77809700
H	1.69596700	-2.38455500	3.66018500
H	0.20118200	-2.38094600	2.68795200
C	0.70211800	-4.64905500	1.22788100
H	1.17284500	-5.24301000	0.43028500
H	0.23217100	-5.35190700	1.93451400
H	-0.09045200	-4.04215700	0.76470900
C	2.82465100	-4.63569900	2.57198700
H	2.39147300	-5.33816000	3.30214500
H	3.35168700	-5.22930500	1.81018800
H	3.57264500	-4.01771400	3.09236300
C	4.97846300	-2.07203200	-3.23985400
H	4.21925300	-1.39934200	-3.66698800
H	5.72663000	-1.45762600	-2.71562400
H	5.48678900	-2.57166300	-4.07877000
C	5.47186500	-4.04860700	-1.81590100
H	5.08225700	-4.85537700	-1.17747800
H	5.98825900	-4.51901600	-2.66799700
H	6.21730900	-3.48827500	-1.23059200
C	3.32718800	-3.92891200	-3.12055400
H	3.80404200	-4.39357200	-3.99867700
H	2.87794800	-4.73235400	-2.51764400
H	2.51024100	-3.28095700	-3.47395900

4.7.7 RSiCl-II

0 1

C	1.11444300	1.58132200	-0.02192400
N	-0.04131500	0.80658600	-0.07396000
C	1.80868100	3.90790200	-0.01924300
C	0.79807500	2.95504800	-0.08815000
C	-0.64375900	3.02958200	-0.19669400
C	-1.53758400	4.09547900	-0.28261700
C	-2.91452300	3.85850000	-0.34390800
C	-3.35566600	2.52280800	-0.33801400
C	-2.48752900	1.42921700	-0.27015000
C	-1.11853200	1.69856100	-0.18218300
C	2.76394900	-0.32005900	0.09094900
C	4.07623400	-0.67823000	-0.26157900
C	4.49530300	-1.99766800	-0.29135000
C	3.55199600	-2.97919800	0.03762700
C	2.22694500	-2.69886200	0.36853500
C	1.81201000	-1.33313700	0.39004000
C	1.29034500	-3.89362500	0.66102400
C	5.91926100	-2.41130400	-0.66939300
C	4.24506500	4.55686600	0.24981400
C	-3.88468200	5.04317700	-0.42397800
C	-2.97827600	0.02988500	-0.33119600
C	-3.51817400	-0.59450000	0.79340700
C	-3.86837000	-1.95017600	0.76618700
C	-3.67166800	-2.65295400	-0.42831600
C	-3.15857400	-2.04953000	-1.58564300
C	-2.81637400	-0.69711000	-1.51433800
C	-4.40620900	-2.61893300	2.03532800
C	-3.01596400	-2.80772900	-2.91010700
C	3.13962500	3.49942600	0.13936000
C	3.41234500	2.12533700	0.19810500
C	2.43056600	1.12321800	0.10145300
C	4.25122500	5.41748100	-1.02311000
H	3.29033000	5.93149600	-1.17451300
H	5.03626100	6.18874200	-0.96426800
H	4.44313300	4.79908800	-1.91360900
C	3.97401400	5.44810800	1.47185100
H	4.75509400	6.21953900	1.56921700
H	3.00460500	5.96279700	1.39519500
H	3.96274900	4.85143600	2.39698000
C	5.63343600	3.93328500	0.41104900
H	5.70632100	3.32542300	1.32607900
H	5.89954700	3.29797300	-0.44806200
H	6.39188600	4.72802500	0.48162700
C	2.06315200	-5.21411900	0.79974300
H	2.81928200	-5.16741500	1.59843800
H	1.35618600	-6.01714500	1.05706100
H	2.56010100	-5.51281800	-0.13507300
C	0.30619900	-4.07628900	-0.50658000
H	-0.33846500	-3.20191600	-0.65761800
H	0.85115900	-4.26113100	-1.44512700
H	-0.35216700	-4.94001400	-0.31794800
C	0.53728900	-3.70363500	1.98517600
H	-0.04529400	-4.60916600	2.21717200

H	1.23631500	-3.52226400	2.81532700
H	-0.18113100	-2.87579400	1.97703500
C	-3.70405900	5.92988100	0.81793800
H	-2.67953300	6.32371200	0.89391000
H	-4.39125400	6.79075000	0.78137900
H	-3.91271200	5.36264000	1.73814800
C	-5.34598900	4.59255100	-0.48184900
H	-5.54912300	3.97438000	-1.36977300
H	-5.63198000	4.01623300	0.41140400
H	-6.00502300	5.47291000	-0.53346100
C	-3.58340600	5.86244600	-1.68845500
H	-4.26885200	6.72214200	-1.76426400
H	-2.55536900	6.25449300	-1.68650600
H	-3.70439800	5.24615000	-2.59273700
C	-4.13337500	-2.33198500	-3.85279000
H	-4.06312900	-1.24931600	-4.03821300
H	-4.06924600	-2.84982700	-4.82373500
H	-5.12527000	-2.53412500	-3.41975500
C	-3.14032100	-4.32212800	-2.72229000
H	-4.13589400	-4.61426500	-2.35558000
H	-2.98693300	-4.83092400	-3.68627300
H	-2.38681900	-4.70441100	-2.01618800
C	6.55270600	-3.16391100	0.51126100
H	5.98548700	-4.06972500	0.77248500
H	7.58008400	-3.47372200	0.26130900
H	6.59530100	-2.52496300	1.40677100
C	5.86874100	-3.33014300	-1.90012000
H	5.28314200	-4.24151700	-1.70779300
H	5.41226200	-2.81262900	-2.75794900
H	6.88559000	-3.64137000	-2.18841700
C	6.80065500	-1.20571700	-1.00243500
H	6.40982800	-0.63733600	-1.86042400
H	6.89399200	-0.51851300	-0.14735900
H	7.81378400	-1.54652800	-1.26510500
C	-1.65272900	-2.51775600	-3.55531000
H	-1.52321800	-1.45307900	-3.79672800
H	-0.82661800	-2.81483700	-2.89252800
H	-1.55326000	-3.07992100	-4.49725400
C	-4.84551200	-4.06369500	1.78759600
H	-5.24132400	-4.49646200	2.71914600
H	-5.63909200	-4.12561700	1.02672000
H	-4.00628100	-4.69788700	1.46281900
C	-5.61737400	-1.83072400	2.55622300
H	-6.41957900	-1.79764300	1.80267000
H	-6.01986900	-2.30518600	3.46544200
H	-5.35409400	-0.79437600	2.81330900
C	-3.29747400	-2.62507800	3.09933500
H	-2.43648700	-3.22144200	2.76053700
H	-2.92790200	-1.61265100	3.31736200
H	-3.66761200	-3.06484400	4.03962000
H	4.44299200	1.80980100	0.34682000
H	1.55207000	4.96878300	-0.06944200
H	-1.14856500	5.11665100	-0.29269900
H	-4.42073200	2.29927500	-0.40631000
H	-2.39766100	-0.18193100	-2.38120000
H	-3.92930900	-3.70923700	-0.46120100

H	-3.61693000	-0.01269900	1.71254900
H	-1.02187000	-1.63957600	0.37019400
H	3.87885000	-4.01619500	0.01874900
H	4.76909800	0.11114000	-0.54364200
Si	0.07457400	-0.74399100	0.75570900
Cl	-0.19377000	-0.29733600	2.78302400

4.7.8 RSiCl-II-TS

0 1

C	-1.29066000	1.53813500	0.22285800
N	-0.10071800	0.82680400	0.16910800
C	-2.08370100	3.84635800	0.31104800
C	-1.03749800	2.92557400	0.21179700
C	0.39129000	3.07384500	0.09999500
C	1.20796000	4.20233700	-0.02005500
C	2.58586400	4.05331800	-0.15036300
C	3.10602500	2.74290400	-0.13530000
C	2.32771900	1.59343300	0.00293300
C	0.93557900	1.76925700	0.10151800
C	-2.91391100	-0.38603700	0.09554300
C	-4.13832800	-0.71038400	-0.50228900
C	-4.53812300	-2.02929700	-0.69306200
C	-3.68492200	-3.02865000	-0.22253000
C	-2.44243600	-2.78110900	0.37050300
C	-2.01038800	-1.42474600	0.44169100
C	-3.39610300	3.39152900	0.43025700
C	-3.61711900	2.00162600	0.39864400
C	-2.60270800	1.04671000	0.26528400
H	-4.64213400	1.63867600	0.49205000
H	-1.85138000	4.91179100	0.31012000
H	0.74314500	5.18889900	-0.01965400
H	4.18544300	2.59330300	-0.21892000
H	-1.02259400	-1.32172500	1.60314400
H	-4.00709100	-4.06508100	-0.32775200
H	-4.76655500	0.10282800	-0.86044800
Si	0.05110200	-0.90249100	0.58512000
Cl	0.63996700	-1.94886200	-1.13729100
C	2.98863900	0.26645900	0.03443900
C	3.63104300	-0.21976700	-1.10832000
C	2.98713500	-0.49729400	1.20779200
C	4.25973400	-1.46508900	-1.10435300
H	3.59888100	0.39153400	-2.01023900
C	3.62519700	-1.74536800	1.25499900
H	2.52507000	-0.07547500	2.10046300
C	4.24422300	-2.19976500	0.08906900
H	4.74031900	-3.17228900	0.10651000
C	-4.59419500	4.33385700	0.58781500
C	-5.29512300	4.04577000	1.92447900
H	-6.16327900	4.71160100	2.05755500
H	-5.65828100	3.00851500	1.98066300
H	-4.60745000	4.20477800	2.76938700
C	-5.58095200	4.10819000	-0.56859400
H	-5.96309900	3.07628800	-0.58675600
H	-6.44825100	4.78149700	-0.47362000

H	-5.09888700	4.30368900	-1.53891700
C	-4.16985200	5.80378300	0.57441900
H	-3.48105700	6.03703600	1.40083700
H	-3.67835100	6.07792900	-0.37177200
H	-5.05432300	6.44934700	0.68800700
C	3.54396900	5.23872900	-0.30036700
C	4.53829300	5.24258200	0.87118500
H	5.23925700	6.08825400	0.78026300
H	4.00998800	5.33627100	1.83261100
H	5.13333300	4.31781100	0.90565500
C	2.80173300	6.57656900	-0.30516000
H	2.25189600	6.74149000	0.63425300
H	3.52078200	7.40265200	-0.41747000
H	2.08646900	6.64326400	-1.13942100
C	4.31144500	5.10798600	-1.62516800
H	4.90017400	4.17942800	-1.66693800
H	3.61816000	5.10346600	-2.48048400
H	5.00906000	5.95170200	-1.75241800
C	-5.85625800	-2.40886800	-1.36977300
C	-6.64814100	-1.17952800	-1.82056300
H	-7.58344900	-1.49754700	-2.30583800
H	-6.91982600	-0.53302800	-0.97181200
H	-6.08597000	-0.57476100	-2.54848700
C	-6.71981900	-3.20673900	-0.37997900
H	-6.94234500	-2.60975300	0.51789500
H	-7.67559900	-3.48993100	-0.84906900
H	-6.22221400	-4.13184400	-0.05288100
C	-5.55333400	-3.27197000	-2.60473700
H	-4.93055000	-2.72088300	-3.32597600
H	-5.01913100	-4.19618700	-2.33840500
H	-6.48956900	-3.56018800	-3.10885700
C	-1.71796300	-4.04623600	0.88911300
C	-0.47452200	-3.80123300	1.74438100
H	0.34882400	-3.34020200	1.18201100
H	-0.68482900	-3.17859400	2.62722000
H	-0.10161300	-4.77007600	2.11211600
C	-1.31126400	-4.92115100	-0.30909700
H	-0.60396000	-4.38735400	-0.95875500
H	-0.82656700	-5.84478200	0.04612000
H	-2.18145000	-5.21060500	-0.91662900
C	-2.69191300	-4.83378000	1.79033300
H	-3.02864900	-4.21730400	2.63821100
H	-3.58479800	-5.18584400	1.25587100
H	-2.18356400	-5.72249900	2.19546600
C	3.70148700	-2.57356800	2.54178800
C	2.71261100	-2.06964700	3.59694300
H	2.73050000	-2.73323000	4.47514600
H	2.96608100	-1.05812800	3.94858900
H	1.68473600	-2.04420300	3.20236200
C	3.38297400	-4.04756700	2.25184400
H	4.11169900	-4.50533900	1.56719400
H	3.40439600	-4.62999500	3.18649400
H	2.38514700	-4.15632400	1.80330900
C	5.12885700	-2.46569200	3.10137000
H	5.22637500	-3.04912800	4.03160200
H	5.86950800	-2.84557100	2.38075700

H	5.38499400	-1.41841900	3.32431200
C	4.91374200	-2.06541800	-2.35144600
C	4.16520800	-3.35533400	-2.72413300
H	3.10132200	-3.14683300	-2.91457800
H	4.59965600	-3.80538100	-3.63150500
H	4.21758000	-4.10377600	-1.91921900
C	6.38717200	-2.38582100	-2.05937700
H	6.49649400	-3.10254100	-1.23183400
H	6.86749400	-2.82757800	-2.94726400
H	6.94116100	-1.47365900	-1.78836900
C	4.85511700	-1.11196200	-3.54653400
H	3.81787200	-0.87818700	-3.83078600
H	5.37716800	-0.16501900	-3.33947400
H	5.34129500	-1.57647000	-4.41821400

4.7.9 RSiBr

0 1

C	-1.22857300	1.61700600	0.32877000
N	-0.05384800	0.87670100	0.22164600
C	-2.00032600	3.92620900	0.33597700
C	-0.95642500	3.00336300	0.28243200
C	0.47776400	3.12542500	0.14999100
C	1.34238700	4.21458500	0.05474500
C	2.71969200	4.01542800	-0.08169800
C	3.19322600	2.69073000	-0.12287200
C	2.35656300	1.57520500	-0.03138000
C	0.98457100	1.80416400	0.11277000
C	-2.81209800	-0.29480600	0.52748600
C	-2.05982200	-1.09453900	1.41694700
C	-2.26696200	-2.48609600	1.48829300
C	-3.17839600	-3.05434400	0.59483000
C	-3.92987200	-2.28938300	-0.30591800
C	-3.74479800	-0.90161200	-0.30810100
C	-4.91165500	-2.91784000	-1.29666300
C	-1.52958100	-3.37423200	2.49418600
C	-4.46148700	4.52147900	0.47537400
C	3.65546100	5.22591000	-0.18404800
C	2.90257300	0.19336000	-0.07940400
C	3.00540700	-0.48769800	-1.29976500
C	3.53092200	-1.78535800	-1.36184000
C	3.92072400	-2.38706600	-0.16044600
C	3.83438700	-1.73733800	1.07765800
C	3.32684700	-0.43588800	1.09675300
C	3.71182900	-2.47325700	-2.71905400
C	4.26958600	-2.40438500	2.38649900
C	-3.32698100	3.49081500	0.42890400
C	-3.56609500	2.10605900	0.48338900
C	-2.54386300	1.15205800	0.45012700
C	-4.27833700	5.42510200	1.70450800
H	-3.31904600	5.96318400	1.67608000
H	-5.08238900	6.17724500	1.75569800
H	-4.30190000	4.83336100	2.63267500
C	-4.42254700	5.37638600	-0.80087500
H	-5.22963100	6.12687600	-0.78855300

H	-3.46841600	5.91438300	-0.90378100
H	-4.55001600	4.74910800	-1.69664300
C	-5.83966100	3.86238000	0.56658200
H	-6.04134900	3.21507200	-0.30087100
H	-5.94486200	3.25706300	1.48008900
H	-6.62189800	4.63667900	0.59098400
C	-5.01733800	-4.43473500	-1.12687700
H	-5.36841800	-4.71208100	-0.12073700
H	-5.73918200	-4.83891500	-1.85272200
H	-4.05366500	-4.93476000	-1.30833100
C	-4.42638700	-2.62143000	-2.72524200
H	-4.37807300	-1.54098900	-2.92480800
H	-3.41787000	-3.03008200	-2.88900700
H	-5.11153900	-3.07227200	-3.46105200
C	-6.30578500	-2.30762900	-1.08457000
H	-7.02439400	-2.74447700	-1.79609000
H	-6.67152800	-2.50366600	-0.06464600
H	-6.30614200	-1.21862800	-1.23912000
C	3.26836500	6.06555800	-1.41135000
H	2.23269000	6.43120600	-1.34618100
H	3.92783800	6.94391600	-1.50318500
H	3.35541900	5.47247100	-2.33481900
C	5.12201200	4.81378600	-0.32989300
H	5.47036000	4.22775600	0.53449700
H	5.29092800	4.21788900	-1.23997000
H	5.75598800	5.71124100	-0.39905000
C	3.52076000	6.08100600	1.08549500
H	4.18302500	6.96047300	1.03231600
H	2.49209200	6.44568200	1.22502400
H	3.79310200	5.49966700	1.97999100
C	5.33381200	-1.53496400	3.07310900
H	4.95346100	-0.53079300	3.31147600
H	5.65821500	-2.00078200	4.01746600
H	6.21844500	-1.41463000	2.42876700
C	4.86054500	-3.79766000	2.15986900
H	5.75144500	-3.76493900	1.51386100
H	5.16541300	-4.23266000	3.12401700
H	4.12974900	-4.48389500	1.70505900
C	-2.55516400	-4.17599600	3.31042300
H	-3.16119800	-4.84006300	2.67674800
H	-2.03806500	-4.80556900	4.05187500
H	-3.24156200	-3.50533300	3.85020100
C	-0.61021200	-4.34006100	1.72821300
H	-1.17909100	-4.98051600	1.03754900
H	0.13749200	-3.78632000	1.13929000
H	-0.07581300	-4.99613300	2.43409500
C	-0.68037900	-2.55275600	3.46640400
H	0.09541900	-1.97558800	2.94192000
H	-1.29620000	-1.85753900	4.05788000
H	-0.16681500	-3.22447000	4.17095600
C	3.04516700	-2.54416000	3.30273100
H	2.59090900	-1.56785200	3.52834400
H	2.27347100	-3.16883700	2.82846000
H	3.32860600	-3.01265300	4.25917700
C	4.24842500	-3.89940000	-2.57718300
H	4.35248300	-4.35576400	-3.57337800

H	5.23998900	-3.92327500	-2.09926200
H	3.56613400	-4.53463700	-1.99133700
C	4.71768300	-1.65644000	-3.54664900
H	5.69084100	-1.59340500	-3.03512800
H	4.87629100	-2.12682100	-4.53048900
H	4.36160100	-0.62984900	-3.71951500
C	2.36973600	-2.53604900	-3.45918100
H	1.62495600	-3.10726200	-2.88541200
H	1.94287300	-1.53798300	-3.63270800
H	2.49954700	-3.02097000	-4.44005200
H	-4.58631500	1.73336000	0.58182900
H	-1.76911400	4.99358100	0.29391900
H	0.92831900	5.22563600	0.08596100
H	4.26062400	2.49633000	-0.23269800
H	3.23935200	0.11555500	2.03579600
H	4.32040900	-3.39784700	-0.19111600
H	2.68137000	0.02526200	-2.20822000
H	-4.28982500	-0.27193300	-1.01394900
H	-3.31375600	-4.13513800	0.61162700
H	-1.44941500	-0.58913500	2.16896100
Si	0.13902000	-0.94532500	0.05969300
Br	-0.77585500	-1.22492100	-2.05267800

4.7.10 RSiBr-I

0 1

C	-2.37285100	-0.05624500	0.19261700
N	-1.07930300	0.38018300	-0.03888600
C	-4.63282500	0.64357500	0.45087200
C	-3.29929900	0.98877500	0.21531200
C	-2.50948800	2.18548500	-0.03514200
C	-2.82080500	3.54084600	-0.12305200
C	-1.81042100	4.48863000	-0.32297900
C	-0.47950200	4.03724600	-0.38641500
C	-0.11928100	2.68795200	-0.29868000
C	-1.15492600	1.76171300	-0.16628600
C	-1.46692100	-2.22022400	0.16793300
C	-1.28095600	-3.54762400	0.36911600
C	-0.05541600	-4.28732900	0.12887800
C	1.22586200	-3.83402600	0.31058000
C	1.84577900	-2.60232800	0.78517000
C	1.41176400	-1.34158600	0.50601000
C	3.15387400	-2.75471500	1.60484400
C	-0.19989600	-5.75295700	-0.36175800
C	-6.47726300	-1.03008800	0.90773400
C	-2.17848600	5.97318300	-0.43638400
C	1.31031600	2.28822700	-0.17198900
C	2.07280700	1.81205000	-1.23990800
C	3.43271700	1.50224500	-1.07245700
C	3.98757200	1.65554300	0.20137400
C	3.24737600	2.10959500	1.30267000
C	1.90435700	2.42475600	1.09042400
C	4.27158100	1.04162000	-2.27017500
C	3.90934700	2.34445000	2.66518300
C	-4.99888100	-0.70327900	0.64841500

C	-4.01672200	-1.71417100	0.59289300
C	-2.67195000	-1.40952500	0.35154100
C	-6.94338100	-0.30462400	2.17976900
H	-6.84245000	0.78717100	2.08941800
H	-8.00353300	-0.52579200	2.38445800
H	-6.35146000	-0.62344300	3.05165800
C	-7.32150800	-0.56429800	-0.28887800
H	-8.38692800	-0.79193500	-0.12217700
H	-7.23498000	0.51979000	-0.45497200
H	-7.00268200	-1.06997200	-1.21340400
C	-6.71592500	-2.52933300	1.10218100
H	-6.43276100	-3.10866400	0.20992800
H	-6.15573400	-2.92477700	1.96346200
H	-7.78484400	-2.71398400	1.28990600
C	3.50881900	-4.21039200	1.92851200
H	3.77842100	-4.78857700	1.03146200
H	4.38376200	-4.23058800	2.59644700
H	2.68379100	-4.73079100	2.43858700
C	2.96831900	-2.01762000	2.93972100
H	2.74722700	-0.95228400	2.78882900
H	2.13959300	-2.45379000	3.51846600
H	3.88553600	-2.08848100	3.54607600
C	4.31736500	-2.13141700	0.82127600
H	5.25696000	-2.21881300	1.39080000
H	4.45799900	-2.63986900	-0.14563700
H	4.14017000	-1.06600900	0.61635100
C	-3.11981000	6.16893800	-1.63507900
H	-4.04632300	5.58554300	-1.52617100
H	-3.40184700	7.22957400	-1.73655900
H	-2.63374000	5.85168200	-2.57058100
C	-0.94943000	6.86113200	-0.64356700
H	-0.24689400	6.79245500	0.20121800
H	-0.40648100	6.59912200	-1.56461900
H	-1.26106500	7.91352200	-0.72972300
C	-2.88555900	6.42466300	0.85093300
H	-3.16030000	7.49012700	0.78766900
H	-3.80784300	5.85336900	1.03388100
H	-2.23023400	6.29052600	1.72547800
C	4.37701700	3.80853000	2.71256800
H	3.52712100	4.49713100	2.58842800
H	4.86312900	4.03164900	3.67650000
H	5.09804300	4.01716300	1.90712900
C	5.12092100	1.42741700	2.87239500
H	5.93538900	1.64439700	2.16569400
H	5.52860200	1.56985300	3.88498400
H	4.84719500	0.36725200	2.76214300
C	0.47148500	-5.85973500	-1.74109900
H	1.54752000	-5.63734600	-1.69311200
H	0.35702200	-6.87842100	-2.14545800
H	0.01794100	-5.15156000	-2.45158200
C	0.46419500	-6.72510600	0.62392900
H	1.54128300	-6.53273700	0.73527100
H	0.00552200	-6.64669400	1.62204500
H	0.34670500	-7.76382000	0.27578000
C	-1.66253000	-6.18125200	-0.53012500
H	-2.20307900	-6.21158100	0.42836100

H	-2.20980000	-5.51675400	-1.21594600
H	-1.69702400	-7.19730900	-0.95225900
C	2.92347700	2.09711900	3.81419100
H	2.08399600	2.80695300	3.80502700
H	2.50585700	1.07952700	3.77434000
H	3.43601200	2.21647600	4.78127600
C	5.73068700	0.77919200	-1.88901900
H	6.29203800	0.45801900	-2.77965700
H	6.22385400	1.68135200	-1.49511300
H	5.82001400	-0.02049100	-1.13744000
C	4.24455800	2.13746700	-3.34714100
H	4.66285500	3.08011300	-2.96103100
H	4.84019100	1.82999800	-4.22172500
H	3.22130100	2.34125900	-3.69556600
C	3.68343300	-0.25449500	-2.84232600
H	3.65529100	-1.04760300	-2.07940800
H	2.65569500	-0.11414100	-3.20242300
H	4.29218500	-0.61027900	-3.68917800
H	-4.30255000	-2.75856200	0.72033800
H	-5.39809800	1.42306000	0.47662400
H	-3.86317500	3.85409900	-0.02545000
H	0.33401600	4.75662900	-0.48577100
H	1.28322700	2.79749600	1.90624700
H	5.04028700	1.42214900	0.34471100
H	1.59587000	1.70192200	-2.21655000
H	1.97989500	-0.47924900	0.87242300
H	1.97793400	-4.60222100	0.11847400
H	-2.14398800	-4.13776400	0.69394500
Si	-0.12625200	-1.06442400	-0.41872400
Br	0.08295600	-1.22749500	-2.65044200

4.7.11 RSiBr-I-TS

0 1

C	-0.81214400	2.09502600	-0.09160400
N	0.12317100	1.07033700	-0.02200200
C	-1.10710900	4.45445300	-0.00158400
C	-0.24124500	3.35615700	0.06918600
C	1.17943700	3.09854100	0.23820700
C	2.28740600	3.93824200	0.38757700
C	3.57140200	3.39927600	0.46864600
C	3.70851300	1.99734500	0.40125000
C	2.63230800	1.11934300	0.26893500
C	1.35565800	1.68818400	0.18259100
C	-2.40972600	0.38968900	-0.46395700
C	-3.52907400	-0.20215700	-1.12647400
C	-3.97880200	-1.48248200	-0.92623700
C	-3.64365900	-2.19615100	0.27540400
C	-2.90431900	-1.72325000	1.33468400
C	-1.94220300	-0.66761000	1.17177900
C	-2.48196900	4.26416500	-0.22644300
C	-2.99602200	2.95525700	-0.38156300
C	-2.16138500	1.83881600	-0.31685300
H	-4.06169000	2.79940100	-0.55123900
H	-0.70973000	5.46535900	0.11666600

H	2.12633600	5.01593100	0.42771000
H	4.70401400	1.55090500	0.46619200
H	-1.72905500	-0.02524300	2.03474900
H	-4.24229900	-3.09078600	0.46382500
H	-4.04607300	0.44783300	-1.83689700
Si	-0.78233400	-0.43382700	-0.21642400
Br	-0.04668200	-2.03504600	-1.51316700
C	2.83806400	-0.34557500	0.18614600
C	3.58654800	-0.88285900	-0.86514900
C	2.28587000	-1.19749100	1.15006000
C	3.78669500	-2.26110500	-0.97644600
H	3.98973100	-0.19489300	-1.60861300
C	2.48196500	-2.58104800	1.08245700
H	1.72423500	-0.75037900	1.97179800
C	3.22980200	-3.08046200	0.01174100
H	3.37751600	-4.16044500	-0.06110800
C	4.82638800	4.26430300	0.62685400
C	4.49251200	5.75682700	0.66930700
H	3.99843400	6.09129400	-0.25591200
H	3.83793800	6.00490400	1.51898700
H	5.41744400	6.34316700	0.78175900
C	5.53924600	3.89222400	1.93630300
H	4.88138800	4.06318000	2.80243700
H	5.84262800	2.83476800	1.95074000
H	6.44731700	4.50258900	2.06875900
C	5.76961700	4.01556100	-0.56053000
H	5.27903000	4.27487000	-1.51148700
H	6.68025200	4.62892800	-0.46521100
H	6.08259900	2.96252700	-0.62157500
C	-3.39447200	5.49848300	-0.29203800
C	-4.85759100	5.12691900	-0.54494800
H	-4.98668900	4.59930400	-1.50246300
H	-5.26382300	4.49129500	0.25677700
H	-5.47096800	6.04020200	-0.58551100
C	-3.31933600	6.26009100	1.04049100
H	-2.29636400	6.59948800	1.26121100
H	-3.96828900	7.15057600	1.01429400
H	-3.64668600	5.62134100	1.87539900
C	-2.93141400	6.41526500	-1.43515300
H	-1.89734900	6.76160300	-1.28990400
H	-2.97452600	5.88884100	-2.40117400
H	-3.57582900	7.30698300	-1.50065100
C	-5.01134700	-2.12976600	-1.85902400
C	-3.19514000	-2.29618900	2.74155900
C	1.90945800	-3.55192400	2.11856800
C	4.55538400	-2.89406600	-2.13997900
C	-5.31723100	-1.25489100	-3.07651700
H	-6.00495600	-1.78483100	-3.75353300
H	-5.80398000	-0.30932100	-2.79170700
H	-4.40382300	-1.01580300	-3.64235200
C	-6.33135800	-2.39071300	-1.11651500
H	-6.75392800	-1.45006100	-0.73059200
H	-7.07057100	-2.84652300	-1.79480800
H	-6.20328800	-3.07408300	-0.26413600
C	-4.42737500	-3.45967900	-2.36231300
H	-4.21064900	-4.15070700	-1.53374300

H	-5.13566100	-3.96102400	-3.04182000
H	-3.48612600	-3.28941500	-2.90762700
C	-4.70470100	-2.25394300	3.02898300
H	-5.27718500	-2.87469600	2.32515500
H	-4.91315600	-2.62445700	4.04588400
H	-5.08847400	-1.22488400	2.95294600
C	-2.49669700	-1.50550100	3.85172000
H	-1.40339100	-1.50229100	3.73999200
H	-2.84348400	-0.46096400	3.88804200
H	-2.72514200	-1.96086100	4.82764600
C	-2.70565000	-3.75110700	2.79107600
H	-2.91544400	-4.19476400	3.77826000
H	-3.20540500	-4.36998600	2.03077600
H	-1.62307800	-3.81186100	2.61147100
C	1.11806900	-2.81830700	3.20019800
H	0.69383400	-3.53976300	3.91505500
H	1.74958500	-2.11774900	3.76787200
H	0.28178300	-2.25229000	2.76453800
C	0.96896700	-4.54308700	1.41507000
H	1.49617100	-5.13605300	0.65282200
H	0.53643300	-5.24660700	2.14454400
H	0.14399700	-4.01558300	0.91267600
C	3.05941200	-4.31754500	2.78970300
H	2.66524500	-5.01978800	3.54186300
H	3.64286700	-4.90063400	2.06156300
H	3.75009800	-3.62505800	3.29525200
C	5.11683000	-1.84244700	-3.09903700
H	4.31856400	-1.23858900	-3.55679300
H	5.81673800	-1.16020500	-2.59242300
H	5.66635600	-2.33731300	-3.91447600
C	5.72675200	-3.72282000	-1.59166500
H	5.38467900	-4.52957400	-0.92648700
H	6.28761300	-4.18819500	-2.41804700
H	6.42212600	-3.08907900	-1.01978300
C	3.59912200	-3.80518300	-2.92600500
H	4.12223400	-4.27245100	-3.77591700
H	3.19382900	-4.61176500	-2.29673300
H	2.74770400	-3.22934500	-3.32093800

4.7.12 RSiBr-II

0 1

C	-1.11028600	1.59174200	0.16528400
N	0.04132900	0.80974800	0.21860500
C	-1.79327500	3.92064800	0.15941600
C	-0.78687900	2.96354600	0.23108100
C	0.65583500	3.03031700	0.33954000
C	1.55693000	4.09040000	0.42121800
C	2.93256200	3.84415200	0.47991700
C	3.36536600	2.50569100	0.47934700
C	2.48963900	1.41781300	0.41779000
C	1.12286500	1.69682700	0.32764300
C	-2.76464800	-0.30272100	0.03364100
C	-4.07531200	-0.66135400	0.39062100
C	-4.49649400	-1.98056300	0.40979200

C	-3.55673800	-2.96067400	0.06632400
C	-2.23304100	-2.67999000	-0.27000600
C	-1.81696100	-1.31500900	-0.28088300
C	-1.29880000	-3.87226300	-0.57806700
C	-5.91898400	-2.39551100	0.79185900
C	-4.22581800	4.58085100	-0.11606100
C	3.91058500	5.02286300	0.55246000
C	2.96625700	0.01458400	0.49184900
C	3.54085000	-0.61477400	-0.61246500
C	3.87108800	-1.97539100	-0.57438200
C	3.62589000	-2.67603200	0.61237600
C	3.08063900	-2.06668400	1.75186000
C	2.75296700	-0.71162600	1.66762000
C	4.44004400	-2.65116700	-1.82602900
C	2.89171200	-2.82092400	3.07283000
C	-3.12579300	3.51807600	-0.00284200
C	-3.40491600	2.14537400	-0.06489900
C	-2.42727500	1.13951400	0.03273200
C	-4.23077400	5.44183700	1.15660200
H	-3.26779300	5.95128300	1.31015800
H	-5.01192700	6.21685100	1.09560600
H	-4.42784900	4.82469600	2.04684400
C	-3.94749400	5.47027800	-1.33780400
H	-4.72479500	6.24521900	-1.43744300
H	-2.97594100	5.98055900	-1.25892300
H	-3.93657800	4.87311800	-2.26261100
C	-5.61679000	3.96392200	-0.28037700
H	-5.69072000	3.35681700	-1.19581300
H	-5.88761700	3.32953900	0.57794800
H	-6.37128700	4.76230000	-0.35219600
C	-2.07143000	-5.19298900	-0.71372800
H	-2.83421600	-5.14495500	-1.50598100
H	-1.36598600	-5.99472200	-0.97911100
H	-2.56034800	-5.49424000	0.22452200
C	-0.30142100	-4.05773600	0.57746300
H	0.34108800	-3.18146400	0.72817500
H	-0.83535400	-4.25184900	1.52046400
H	0.35908700	-4.91682100	0.37574400
C	-0.56000900	-3.67491700	-1.90902900
H	0.02144200	-4.57830300	-2.15213600
H	-1.26700500	-3.48890200	-2.73130300
H	0.15842800	-2.84647500	-1.90165800
C	3.73276700	5.90470800	-0.69334200
H	2.71071300	6.30500600	-0.76874500
H	4.42580200	6.76110600	-0.66262700
H	3.93528800	5.33161000	-1.61128200
C	5.36907300	4.56302400	0.60903500
H	5.57043800	3.94798600	1.49953400
H	5.64908500	3.98046500	-0.28206100
H	6.03390500	5.43933700	0.65463600
C	3.61759300	5.85005100	1.81370000
H	4.30884500	6.70556600	1.88378600
H	2.59218100	6.24889900	1.81225500
H	3.73667700	5.23731800	2.72065200
C	3.99439900	-2.36296900	4.04148400
H	3.93822100	-1.27890200	4.22354300

H	3.89804200	-2.87807500	5.01123400
H	4.99276300	-2.58285600	3.63263600
C	2.99526300	-4.33746000	2.88928900
H	3.99492300	-4.64665500	2.54867000
H	2.80880200	-4.84251600	3.84939600
H	2.25406800	-4.70791900	2.16429500
C	-6.55969200	-3.13758700	-0.39154600
H	-5.99533900	-4.04215800	-0.66307200
H	-7.58622100	-3.44784300	-0.13869800
H	-6.60596600	-2.49137500	-1.28163800
C	-5.86307200	-3.32448800	2.01473100
H	-5.27961200	-4.23494600	1.81181000
H	-5.40143100	-2.81457700	2.87435000
H	-6.87876100	-3.63686500	2.30585100
C	-6.79697200	-1.19155800	1.13941700
H	-6.40116600	-0.63103700	2.00028000
H	-6.89344100	-0.49703200	0.29062900
H	-7.80928800	-1.53316700	1.40415800
C	1.51908900	-2.50664700	3.68581000
H	1.40412000	-1.44039600	3.92754700
H	0.70337300	-2.78605900	3.00291200
H	1.38676600	-3.06899800	4.62356000
C	4.82763700	-4.10957300	-1.57232400
H	5.24464200	-4.54828400	-2.49175500
H	5.59085300	-4.19954600	-0.78372500
H	3.95867700	-4.72031800	-1.28253700
C	5.69262700	-1.89396600	-2.29200900
H	6.46607600	-1.89259400	-1.50827100
H	6.11564000	-2.37048600	-3.19081800
H	5.46947900	-0.84749000	-2.54628800
C	3.37475200	-2.61881400	-2.93314100
H	2.47879400	-3.18173500	-2.62906500
H	3.05268600	-1.59356900	-3.16696900
H	3.76531700	-3.07321700	-3.85805800
H	-4.43612200	1.83375800	-0.21864000
H	-1.53232800	4.98047700	0.20919600
H	1.17509600	5.11427500	0.42889300
H	4.42894200	2.27560900	0.54912800
H	2.30803400	-0.19222000	2.51865500
H	3.86973500	-3.73529900	0.65354400
H	3.67993100	-0.03370100	-1.52691900
H	1.02453600	-1.61687300	-0.32609600
H	-3.88522300	-3.99729000	0.07776900
H	-4.76450200	0.12727200	0.68403900
Si	-0.08529000	-0.71488500	-0.65816100
Br	0.16050700	-0.15783300	-2.83104900

4.7.13 RSiBr-II-TS

0 1

C	-1.34230800	1.61284200	0.25292600
N	-0.14920000	0.90607700	0.22920500
C	-2.14309900	3.91985700	0.30786200
C	-1.09351200	3.00080100	0.23551900
C	0.33619600	3.15326200	0.14917400

C	1.14608500	4.28694200	0.03568200
C	2.52653200	4.14682900	-0.07280300
C	3.05322500	2.83988500	-0.04444000
C	2.28195400	1.68417600	0.08838800
C	0.88656300	1.85073100	0.16870500
C	-2.96151700	-0.31766100	0.13049000
C	-4.18656800	-0.65543200	-0.45833700
C	-4.58962400	-1.97763200	-0.61502900
C	-3.74027500	-2.96595000	-0.11508200
C	-2.49511700	-2.70703000	0.46641500
C	-2.05625000	-1.34982700	0.49597000
C	-3.45638900	3.46256900	0.40577700
C	-3.67259600	2.07208800	0.38325600
C	-2.65363200	1.11793600	0.27705200
H	-4.69813800	1.70816800	0.46460100
H	-1.91315400	4.98579600	0.30308700
H	0.67384100	5.26989700	0.02301500
H	4.13459500	2.69688400	-0.11263200
H	-1.05240600	-1.24294100	1.64411200
H	-4.06801500	-4.00355800	-0.18681600
H	-4.81412000	0.14925100	-0.83604400
Si	0.00255500	-0.83490700	0.59864300
Br	0.61458900	-1.92000300	-1.28892800
C	2.96609600	0.36909400	0.12671700
C	3.67041000	-0.07787200	-0.99594400
C	2.94226300	-0.41429600	1.28659000
C	4.33945000	-1.30202400	-0.98611600
H	3.65704700	0.54837300	-1.88803100
C	3.61422200	-1.64373200	1.33869200
H	2.43225800	-0.02251400	2.16661600
C	4.29571600	-2.05849100	0.19260900
H	4.82091400	-3.01568200	0.21564000
C	-4.65975100	4.40274300	0.53295800
C	-5.38336400	4.12565000	1.85982800
H	-6.25450300	4.79144700	1.97183800
H	-5.74625300	3.08845300	1.91899500
H	-4.71063900	4.29314100	2.71506400
C	-5.62525100	4.16283600	-0.63840200
H	-6.00424600	3.12971200	-0.65301900
H	-6.49589200	4.83457500	-0.56549300
H	-5.12665200	4.35002200	-1.60197800
C	-4.23923500	5.87369800	0.51259900
H	-3.56671000	6.11733900	1.34935800
H	-3.73081100	6.13966800	-0.42696000
H	-5.12742300	6.51780000	0.60306900
C	3.47958500	5.33721800	-0.21412000
C	4.45677900	5.35186400	0.97166100
H	5.15348500	6.20176200	0.88770000
H	3.91402400	5.44558500	1.92499900
H	5.05717600	4.43109200	1.01814000
C	2.72932800	6.67039300	-0.23540000
H	2.16480400	6.83570500	0.69519900
H	3.44481300	7.50047600	-0.34073200
H	2.02592700	6.72919700	-1.08027900
C	4.26693100	5.20533300	-1.52709800
H	4.86176300	4.28017400	-1.55610900

H	3.58616400	5.19282300	-2.39233100
H	4.96125300	6.05268500	-1.64801300
C	-5.90767700	-2.37159100	-1.28315400
C	-6.69434900	-1.15278000	-1.77018600
H	-7.62920100	-1.48127800	-2.24928500
H	-6.96665400	-0.48223300	-0.94049900
H	-6.12811400	-0.57014900	-2.51286900
C	-6.77599800	-3.13835500	-0.27313400
H	-6.99867000	-2.51546700	0.60693000
H	-7.73159400	-3.43162000	-0.73633700
H	-6.28233600	-4.05554200	0.08111000
C	-5.60464100	-3.27024500	-2.49252800
H	-4.97767100	-2.74207800	-3.22714100
H	-5.07465200	-4.18852900	-2.19866400
H	-6.54061800	-3.56899600	-2.99094900
C	-1.78383900	-3.96173200	1.02868500
C	-0.51552800	-3.70766300	1.84322600
H	0.30007300	-3.27647200	1.24621900
H	-0.69363400	-3.05202000	2.70899500
H	-0.14816800	-4.66930900	2.23474500
C	-1.42342200	-4.90610200	-0.13098300
H	-0.71464600	-4.42603300	-0.82019200
H	-0.95569100	-5.82276200	0.26261600
H	-2.31082800	-5.20393500	-0.70847500
C	-2.75684600	-4.67874100	1.98819200
H	-3.05652000	-4.01349100	2.81279900
H	-3.67155400	-5.02865200	1.49034400
H	-2.26341300	-5.56139900	2.42402800
C	3.65866200	-2.49841000	2.60948400
C	2.64551900	-2.01609800	3.65118600
H	2.64678800	-2.69485900	4.51792700
H	2.88809300	-1.00975200	4.02489600
H	1.62616800	-1.98798500	3.23567200
C	3.34407800	-3.96424200	2.27580700
H	4.08752700	-4.40526500	1.59595100
H	3.34235000	-4.57058000	3.19549500
H	2.35716000	-4.05879100	1.80070500
C	5.07119900	-2.40718800	3.20804300
H	5.14275000	-3.01129000	4.12735000
H	5.83037500	-2.77346100	2.49987000
H	5.32379000	-1.36585600	3.46087900
C	5.07352600	-1.85391900	-2.21100700
C	4.40369200	-3.17047900	-2.63595000
H	3.34157800	-3.00771100	-2.87535900
H	4.90090600	-3.58638300	-3.52722600
H	4.45173500	-3.92837300	-1.83971300
C	6.54540200	-2.11202700	-1.85563800
H	6.64911100	-2.83577300	-1.03346700
H	7.08564600	-2.51763100	-2.72621000
H	7.04384400	-1.18009200	-1.54672800
C	5.02536400	-0.88532500	-3.39434100
H	3.99206000	-0.69104600	-3.71982500
H	5.49701800	0.07949800	-3.15199000
H	5.56801100	-1.31516400	-4.25034500

4.7.14 RSiI

0 1

C	-1.17698700	1.67570500	0.45321900
N	-0.01455200	0.91570900	0.34557300
C	-1.91073000	3.99705700	0.43420500
C	-0.88333300	3.05633000	0.38318900
C	0.55023300	3.15390100	0.22520500
C	1.42649000	4.22912900	0.09078600
C	2.79753000	4.00782200	-0.07182900
C	3.25170400	2.67628600	-0.09119500
C	2.40323600	1.57353800	0.04422800
C	1.03733000	1.82515000	0.20396300
C	-2.79736900	-0.20739300	0.65364800
C	-2.03365000	-1.03687800	1.50763600
C	-2.28427800	-2.42284400	1.58026900
C	-3.24811100	-2.95557000	0.72176100
C	-4.01684900	-2.16009700	-0.13811300
C	-3.78546500	-0.77969800	-0.14266000
C	-5.08096300	-2.74762000	-1.06723500
C	-1.54143700	-3.33966200	2.55586600
C	-4.36032400	4.63456900	0.59199900
C	3.74716200	5.20211200	-0.22323800
C	2.94638900	0.18905500	0.03278600
C	3.13006500	-0.49672400	-1.17478200
C	3.68804100	-1.78168100	-1.19862600
C	4.03146000	-2.36515600	0.02570600
C	3.86434500	-1.71056100	1.25279200
C	3.32006800	-0.42415300	1.23535800
C	3.95473700	-2.47534700	-2.53876000
C	4.26262800	-2.35256200	2.58583700
C	-3.24360500	3.58486100	0.54525200
C	-3.50502300	2.20533200	0.61239500
C	-2.49928700	1.23297200	0.57972200
C	-4.14896800	5.54817600	1.80916200
H	-3.18103600	6.06945200	1.76491500
H	-4.93953500	6.31445400	1.86020100
H	-4.17294700	4.96714900	2.74406400
C	-4.32055700	5.47465200	-0.69404400
H	-5.11530400	6.23815700	-0.68183500
H	-3.35909800	5.99613600	-0.81252500
H	-4.46720000	4.83978600	-1.58147200
C	-5.74814400	3.99952300	0.70483600
H	-5.97055300	3.34774300	-0.15417400
H	-5.85312100	3.40469700	1.62525700
H	-6.51699600	4.78704200	0.73045500
C	-5.20565400	-4.26549900	-0.92066100
H	-5.49525800	-4.55808700	0.10057500
H	-5.98215400	-4.64050000	-1.60449100
H	-4.26593100	-4.77877900	-1.17590300
C	-4.70634100	-2.43063600	-2.52399700
H	-4.64314100	-1.34782200	-2.70549900
H	-3.72805600	-2.86407200	-2.78082900
H	-5.46357000	-2.84498900	-3.20872400
C	-6.44033000	-2.11329500	-0.73220600
H	-7.22291100	-2.52022300	-1.39213600

H	-6.72782500	-2.32283200	0.30984800
H	-6.42756500	-1.02141400	-0.86509300
C	3.34376800	6.01760700	-1.46154800
H	2.31474000	6.39892000	-1.38232100
H	4.01261300	6.88434200	-1.58898300
H	3.40219900	5.40165400	-2.37219900
C	5.20429200	4.76592300	-0.39159700
H	5.56432500	4.19642700	0.47902800
H	5.34431200	4.14562300	-1.29018600
H	5.84853200	5.65244000	-0.49719400
C	3.65240000	6.08876200	1.02811000
H	4.32556800	6.95696100	0.93927100
H	2.63232300	6.47169800	1.18121400
H	3.93642900	5.52493300	1.93014700
C	5.27738300	-1.44895700	3.30274000
H	4.86314600	-0.45194000	3.51309100
H	5.57595400	-1.89545100	4.26482900
H	6.18327700	-1.31381800	2.69176400
C	4.89653100	-3.73285900	2.39920000
H	5.81255400	-3.68440500	1.79044900
H	5.17160400	-4.15041000	3.37988800
H	4.20245400	-4.44157600	1.92187200
C	-2.56025000	-4.14587600	3.37624600
H	-3.18064100	-4.79779100	2.74404600
H	-2.03531900	-4.78935500	4.09990900
H	-3.23311900	-3.47864000	3.93688800
C	-0.64870700	-4.30106900	1.75366900
H	-1.23902100	-4.91575700	1.05758700
H	0.09861300	-3.74621300	1.16535700
H	-0.11339200	-4.98101700	2.43586300
C	-0.66677000	-2.54640700	3.52870100
H	0.10962000	-1.97177700	3.00268800
H	-1.26454300	-1.85273100	4.14028600
H	-0.15186900	-3.23632500	4.21430000
C	3.00788400	-2.51319900	3.45549600
H	2.52229800	-1.54604700	3.65341900
H	2.27046700	-3.16103400	2.95834600
H	3.26477300	-2.96535300	4.42718300
C	4.52455500	-3.88406500	-2.35753900
H	4.69074100	-4.34483600	-3.34322100
H	5.49164700	-3.87510300	-1.83134000
H	3.83302000	-4.53472200	-1.80015800
C	4.97540900	-1.63744000	-3.32609600
H	5.92132300	-1.54299200	-2.77018700
H	5.19310500	-2.11135700	-4.29684700
H	4.59981700	-0.62273500	-3.52564000
C	2.65162400	-2.58272700	-3.34048100
H	1.89563600	-3.16673700	-2.79449300
H	2.20876300	-1.59810800	-3.54767200
H	2.83911700	-3.07635900	-4.30759300
H	-4.53099000	1.85150100	0.71944700
H	-1.66226200	5.05962100	0.37399500
H	1.02640600	5.24606600	0.10948000
H	4.31432100	2.46468100	-0.21462900
H	3.17517200	0.13311400	2.16396400
H	4.46065800	-3.36425200	0.02293000

H	2.84331500	0.00268900	-2.10303600
H	-4.34314100	-0.12852400	-0.81838400
H	-3.41770200	-4.03136300	0.73868700
H	-1.39727700	-0.55420200	2.25356700
Si	0.14445700	-0.90307900	0.16626000
I	-0.80303200	-1.18066800	-2.19662200

4.7.15 RSiI-I

0 1

C	-2.35964400	0.01984800	0.38786900
N	-1.06358900	0.44593000	0.14863000
C	-4.61989100	0.73145500	0.60805400
C	-3.28418800	1.06680300	0.37184700
C	-2.49193400	2.25243600	0.07818900
C	-2.80065200	3.60285700	-0.07208500
C	-1.78857000	4.53797600	-0.31911600
C	-0.45809400	4.08245700	-0.36072700
C	-0.10070200	2.73873200	-0.20537800
C	-1.13826000	1.82192800	-0.03288500
C	-1.46853800	-2.15064400	0.38178800
C	-1.29157000	-3.47981800	0.57942800
C	-0.08271200	-4.23222200	0.29980900
C	1.20946000	-3.79311800	0.43623200
C	1.85984200	-2.57164300	0.89548900
C	1.42951600	-1.30390800	0.64211200
C	3.19103200	-2.74343600	1.67309700
C	-0.25951900	-5.69889300	-0.17759100
C	-6.47162500	-0.92480400	1.09821900
C	-2.15441700	6.01576400	-0.50498600
C	1.32608300	2.33959800	-0.04705700
C	2.12708900	1.90826100	-1.10537900
C	3.48235300	1.59953900	-0.90340700
C	3.99481700	1.71369300	0.39226100
C	3.21591600	2.12666300	1.48300300
C	1.87698800	2.43566900	1.23820600
C	4.36180200	1.18490600	-2.08847800
C	3.83020100	2.32925900	2.87272000
C	-4.99097800	-0.60896000	0.83856200
C	-4.01242200	-1.62406400	0.80963300
C	-2.66517200	-1.32901300	0.56899500
C	-6.94107200	-0.16692400	2.34997200
H	-6.83562000	0.92197400	2.23348200
H	-8.00298200	-0.37928100	2.55484200
H	-6.35450800	-0.46649800	3.23224900
C	-7.30875000	-0.48576400	-0.11341000
H	-8.37553400	-0.70657100	0.05363200
H	-7.21872000	0.59377800	-0.30522100
H	-6.98684900	-1.01441800	-1.02389600
C	-6.71590000	-2.41811100	1.32807300
H	-6.43170600	-3.01981100	0.45108600
H	-6.15992300	-2.79432500	2.20062900
H	-7.78606000	-2.59470200	1.51642500
C	3.56528600	-4.20611700	1.93686400
H	3.80472200	-4.75330900	1.01213400

H	4.46432300	-4.24099100	2.57133100
H	2.76329200	-4.74808600	2.46094500
C	3.03246300	-2.05401900	3.03655900
H	2.79676600	-0.98702900	2.92556800
H	2.22246100	-2.51765800	3.62047300
H	3.96507100	-2.13518300	3.61772000
C	4.33092500	-2.08519600	0.88351500
H	5.28486300	-2.18776800	1.42594500
H	4.44923500	-2.55760900	-0.10438000
H	4.14499800	-1.01403900	0.72157200
C	-3.09859300	6.15350400	-1.70950400
H	-4.02611800	5.57849200	-1.56935400
H	-3.37855700	7.20841200	-1.86293900
H	-2.61574100	5.78891800	-2.62928300
C	-0.92439000	6.89001700	-0.75956900
H	-0.21998200	6.86327100	0.08601600
H	-0.38399800	6.58077000	-1.66739400
H	-1.23460900	7.93719900	-0.89828700
C	-2.85700200	6.53196800	0.76021600
H	-3.13011200	7.59345200	0.64472500
H	-3.77971100	5.97214500	0.97392100
H	-2.19937400	6.44040400	1.63850900
C	4.24487300	3.80542500	2.98999200
H	3.37544300	4.46940700	2.86718800
H	4.69530100	4.00664200	3.97582600
H	4.98079400	4.06965700	2.21491400
C	5.06709000	1.44747400	3.08142000
H	5.89335500	1.72143200	2.40895100
H	5.43933000	1.56351800	4.11086100
H	4.83580500	0.38347900	2.92187000
C	0.38780700	-5.83317400	-1.56589000
H	1.46777800	-5.62723600	-1.53984600
H	0.25137700	-6.85555100	-1.95365900
H	-0.06707400	-5.12804900	-2.27849800
C	0.40362400	-6.67122600	0.80872500
H	1.48552100	-6.49530500	0.89932200
H	-0.03670200	-6.57318500	1.81337900
H	0.26245500	-7.71180800	0.47515500
C	-1.73175100	-6.10337600	-0.32000500
H	-2.25715300	-6.12220400	0.64711700
H	-2.27875500	-5.43176100	-0.99914500
H	-1.78993500	-7.12004100	-0.73803000
C	2.81985900	2.00479700	3.98081700
H	1.95646400	2.68527200	3.97160100
H	2.43913400	0.97615100	3.89057700
H	3.29866400	2.10458900	4.96724900
C	5.80112800	0.88216300	-1.66462800
H	6.39135200	0.58995300	-2.54651600
H	6.29284500	1.75832500	-1.21437100
H	5.84902700	0.05030600	-0.94490200
C	4.39021100	2.33280500	-3.10989800
H	4.80689600	3.24847800	-2.66209400
H	5.01343600	2.05983100	-3.97672500
H	3.38302000	2.56947200	-3.48373300
C	3.77967400	-0.07251200	-2.74665000
H	3.70423900	-0.90007100	-2.02455600

H	2.77174000	0.10295600	-3.14628300
H	4.41950600	-0.39939400	-3.58211800
H	-4.30311600	-2.66497700	0.95328000
H	-5.38357900	1.51288800	0.60556400
H	-3.84235800	3.92250700	0.01051600
H	0.35651700	4.79524500	-0.49281800
H	1.22684000	2.77636600	2.04549700
H	5.04404800	1.48228100	0.56180400
H	1.68318400	1.83123800	-2.10057100
H	2.01798900	-0.44859800	0.99250400
H	1.94472200	-4.56988900	0.21660300
H	-2.15326000	-4.06073600	0.92325700
Si	-0.13010300	-1.01006400	-0.24101300
I	0.01973500	-1.22467300	-2.70501800

4.7.16 RSiI-I-TS

0 1

C	-0.90886800	2.16650600	-0.02703100
N	0.06024900	1.17519900	0.05398500
C	-1.28051000	4.51692400	0.01059900
C	-0.37857500	3.44932200	0.09674900
C	1.05197000	3.24277700	0.24867000
C	2.13111500	4.12461400	0.35896300
C	3.43456800	3.63350300	0.43038400
C	3.61887800	2.23623600	0.39714500
C	2.57287700	1.31666200	0.30521800
C	1.27504200	1.83761200	0.22286300
C	-2.45050100	0.40526400	-0.37192400
C	-3.55218500	-0.22762500	-1.02193900
C	-3.94660600	-1.52711100	-0.82198300
C	-3.57672300	-2.22606500	0.37699700
C	-2.85463700	-1.72069100	1.43504300
C	-1.92976000	-0.63687600	1.26350700
C	-2.65048800	4.27692600	-0.19606300
C	-3.12174900	2.94896000	-0.32015400
C	-2.24993400	1.86219200	-0.23931800
H	-4.18248100	2.75441200	-0.48046200
H	-0.91518000	5.54271000	0.10088300
H	1.93176800	5.19651100	0.37629500
H	4.63019700	1.82671200	0.46083400
H	-1.72127400	0.01028900	2.12405700
H	-4.13906600	-3.14336000	0.56856700
H	-4.10329800	0.40249100	-1.72454300
Si	-0.79001400	-0.36610800	-0.14103500
I	0.01443000	-2.08994400	-1.61724400
C	2.84073000	-0.13974100	0.27281800
C	3.65240000	-0.67574000	-0.73159900
C	2.30194600	-0.98427900	1.25115100
C	3.93000600	-2.04402400	-0.78119500
H	4.04472900	0.00428800	-1.48801000
C	2.57694300	-2.35612900	1.24740600
H	1.69266400	-0.53738800	2.03826500
C	3.38514100	-2.85468500	0.22105300
H	3.59530200	-3.92652400	0.19783200

C	4.66081200	4.54530300	0.54435700
C	4.27534200	6.02590700	0.55327600
H	3.75395400	6.31771000	-0.37139600
H	3.62709200	6.27398500	1.40778900
H	5.18079200	6.64693700	0.63343800
C	5.40784900	4.23363800	1.85054000
H	4.75853200	4.40428800	2.72314900
H	5.74914500	3.18833800	1.88737000
H	6.29551300	4.87906400	1.95125300
C	5.59248000	4.29750400	-0.65231100
H	5.07765000	4.51447300	-1.60105200
H	6.48280600	4.94392900	-0.58882300
H	5.94058400	3.25447300	-0.69127700
C	-3.60398500	5.47870300	-0.27951000
C	-5.05600800	5.05354500	-0.51094000
H	-5.17566300	4.50198700	-1.45610500
H	-5.43375200	4.42169400	0.30752100
H	-5.69989500	5.94488600	-0.56466400
C	-3.54278300	6.27077500	1.03587600
H	-2.52976900	6.64842900	1.23981300
H	-4.22121200	7.13853600	0.99690700
H	-3.84149900	5.63957400	1.88710100
C	-3.18155100	6.38559600	-1.44599700
H	-2.15851400	6.76932700	-1.31762600
H	-3.21515400	5.83737000	-2.40019200
H	-3.85603900	7.25371500	-1.52445200
C	-4.96089500	-2.21321700	-1.74727300
C	-3.12947000	-2.29399500	2.84497300
C	2.03297100	-3.31388500	2.31088400
C	4.77680200	-2.67673800	-1.88955700
C	-5.30025800	-1.35436100	-2.96735900
H	-5.97019300	-1.91091900	-3.64085700
H	-5.81974800	-0.42551900	-2.68541000
H	-4.39658500	-1.08546000	-3.53546200
C	-6.26792300	-2.51374400	-0.99679600
H	-6.71899800	-1.58559000	-0.61285600
H	-6.99533300	-2.99673300	-1.66904000
H	-6.11275400	-3.18835000	-0.14190000
C	-4.33643400	-3.52544200	-2.24798200
H	-4.08986800	-4.20442700	-1.41783500
H	-5.03311500	-4.05373500	-2.91901400
H	-3.40580500	-3.32644700	-2.80175600
C	-4.64151500	-2.32134800	3.12133400
H	-5.17866700	-2.97732300	2.42167300
H	-4.83921700	-2.68981600	4.14109300
H	-5.07387300	-1.31299200	3.03037500
C	-2.47961000	-1.45927900	3.95237300
H	-1.38635000	-1.40891400	3.85379000
H	-2.87289900	-0.43082100	3.97023500
H	-2.69943800	-1.91190100	4.93154100
C	-2.57223000	-3.72358000	2.91175300
H	-2.76907600	-4.16846100	3.90107700
H	-3.03615400	-4.37137400	2.15275700
H	-1.48663500	-3.73501800	2.74112400
C	1.17626200	-2.58080100	3.34181300
H	0.77387500	-3.29416600	4.07710100

H	1.75463100	-1.82360300	3.89320400
H	0.32190200	-2.08009600	2.86339800
C	1.16665400	-4.38605300	1.63198700
H	1.74203700	-4.97538600	0.90243800
H	0.76267400	-5.08513200	2.38181200
H	0.32104700	-3.92927600	1.09563600
C	3.20710600	-3.98379100	3.04026200
H	2.83413400	-4.67245100	3.81542200
H	3.83835400	-4.56576800	2.35221000
H	3.84696500	-3.23212000	3.52784700
C	5.30826900	-1.63366500	-2.87466700
H	4.49197600	-1.09825200	-3.38306200
H	5.94906700	-0.89022300	-2.37604800
H	5.91382600	-2.12772700	-3.64992100
C	5.97511100	-3.40711200	-1.26475700
H	5.65770100	-4.20487100	-0.57689000
H	6.59403500	-3.87075300	-2.04964300
H	6.60846200	-2.70775300	-0.69735000
C	3.90852100	-3.67835100	-2.66720800
H	4.49230000	-4.15081900	-3.47359100
H	3.52537500	-4.47885400	-2.01663700
H	3.04252100	-3.17247600	-3.12197100

4.7.17 RSiI-II

0 1

C	-1.10422300	1.58096700	0.34220900
N	0.04319500	0.79115500	0.39117300
C	-1.77524800	3.91230400	0.34553100
C	-0.77344700	2.95031300	0.41527900
C	0.67032200	3.00841900	0.52210200
C	1.57914400	4.06180500	0.60380100
C	2.95348100	3.80535000	0.65424400
C	3.37762400	2.46411300	0.64776000
C	2.49385500	1.38267900	0.58789000
C	1.12920300	1.67254000	0.50345100
C	-2.76351300	-0.30500200	0.17572000
C	-4.07264000	-0.66743600	0.53318600
C	-4.49601600	-1.98638800	0.52922300
C	-3.55959500	-2.96161000	0.16283200
C	-2.23731000	-2.67704600	-0.17571200
C	-1.81983300	-1.31312400	-0.16277100
C	-1.30446300	-3.86217300	-0.51125300
C	-5.91735800	-2.40619400	0.91011800
C	-4.20328500	4.58594200	0.06415100
C	3.93992100	4.97720200	0.72389600
C	2.95659700	-0.02494500	0.65921500
C	3.55855200	-0.64920700	-0.43334200
C	3.87404200	-2.01358300	-0.39819900
C	3.58997700	-2.72221200	0.77507500
C	3.01777500	-2.11797800	1.90402500
C	2.70157500	-0.76030100	1.82114300
C	4.46864400	-2.68494100	-1.64024500
C	2.79110700	-2.88013300	3.21443700
C	-3.10926700	3.51690800	0.17612400

C	-3.39503400	2.14616400	0.10213500
C	-2.42190700	1.13580800	0.19610300
C	-4.20794500	5.44133700	1.34055300
H	-3.24286600	5.94508300	1.49961800
H	-4.98478400	6.22070200	1.28020500
H	-4.41139400	4.82144900	2.22745300
C	-3.91598700	5.47898900	-1.15289900
H	-4.68917900	6.25806300	-1.25218500
H	-2.94228200	5.98422200	-1.06831900
H	-3.90441200	4.88550700	-2.08004300
C	-5.59687800	3.97701400	-0.10752900
H	-5.67128100	3.37538600	-1.02651800
H	-5.87334800	3.33933100	0.74654600
H	-6.34704800	4.77964300	-0.17716300
C	-2.07481700	-5.18298500	-0.65567200
H	-2.84485500	-5.12755400	-1.44038300
H	-1.36997400	-5.97980700	-0.93698300
H	-2.55458300	-5.49605600	0.28347200
C	-0.29234700	-4.05953800	0.62903200
H	0.34470100	-3.18070600	0.78917100
H	-0.81386000	-4.27535100	1.57424500
H	0.37323900	-4.90890000	0.40430700
C	-0.58279500	-3.64272600	-1.84788600
H	0.00186100	-4.53859000	-2.11056500
H	-1.29978700	-3.44908600	-2.65965500
H	0.13124300	-2.80997900	-1.83568600
C	3.76168300	5.86193900	-0.51983000
H	2.74212100	6.26958400	-0.58927300
H	4.46098200	6.71330800	-0.49166200
H	3.95513300	5.28860100	-1.43956200
C	5.39550200	4.50732400	0.77229000
H	5.59721100	3.88935500	1.66068500
H	5.66704100	3.92454400	-0.12127400
H	6.06646700	5.37903400	0.81603600
C	3.65902000	5.80443600	1.98783100
H	4.35653400	6.65502800	2.05557400
H	2.63644600	6.21046300	1.99223900
H	3.77853100	5.18958300	2.89329100
C	3.88134200	-2.44584000	4.20790800
H	3.83716500	-1.36245500	4.39722400
H	3.75863200	-2.96694400	5.17146800
H	4.88420200	-2.67713700	3.81674200
C	2.87696100	-4.39654500	3.02039700
H	3.87909600	-4.71731300	2.69831900
H	2.66345400	-4.90655900	3.97221300
H	2.14618200	-4.75068100	2.27704200
C	-6.56379400	-3.12784000	-0.28278900
H	-6.00198600	-4.02869000	-0.57141000
H	-7.58974800	-3.44073500	-0.03090400
H	-6.61264800	-2.46707500	-1.16198700
C	-5.85730200	-3.35544600	2.11712700
H	-5.27544400	-4.26285300	1.89665200
H	-5.39176700	-2.86030100	2.98325100
H	-6.87207900	-3.67182100	2.40710300
C	-6.79253800	-1.20724600	1.28118200
H	-6.39296200	-0.66186700	2.14998400

H	-6.89113000	-0.49839500	0.44457200
H	-7.80431000	-1.55207300	1.54380000
C	1.41184300	-2.55068600	3.80400100
H	1.30934900	-1.48569300	4.05660200
H	0.60521800	-2.80837400	3.10211700
H	1.25222000	-3.12167400	4.73220000
C	4.82918400	-4.15098400	-1.39069100
H	5.26545800	-4.58642700	-2.30269200
H	5.56908500	-4.25929500	-0.58243100
H	3.94430400	-4.75237900	-1.13104100
C	5.74353800	-1.94057300	-2.06430000
H	6.49592500	-1.95928100	-1.26054000
H	6.18345800	-2.41281000	-2.95723600
H	5.54188000	-0.88814700	-2.31190400
C	3.43448700	-2.62709600	-2.77550800
H	2.51941100	-3.17356000	-2.49889700
H	3.13976100	-1.59445600	-3.01347100
H	3.84076300	-3.08451800	-3.69213000
H	-4.42653700	1.83959500	-0.06022600
H	-1.50964600	4.97070000	0.40037500
H	1.20494400	5.08840600	0.61638000
H	4.43990900	2.22679700	0.71268300
H	2.23573600	-0.24466400	2.66313700
H	3.82380800	-3.78383200	0.81393200
H	3.72872300	-0.06154900	-1.33839800
H	1.02874500	-1.60621400	-0.28049700
H	-3.88949200	-3.99789300	0.15696500
H	-4.75829700	0.11760600	0.84450500
Si	-0.09485600	-0.69713600	-0.54597700
I	0.12142200	0.03680900	-2.91357900

4.7.18 RSiI-II-TS

0 1

C	-1.39054300	1.67982000	0.30226500
N	-0.19692000	0.97564000	0.30916900
C	-2.19210900	3.98772600	0.31148100
C	-1.14283800	3.06752300	0.26405700
C	0.28720500	3.22015700	0.19022400
C	1.09163000	4.35571700	0.06267500
C	2.47322900	4.22016500	-0.03326900
C	3.00381200	2.91655300	0.02509800
C	2.23842100	1.75784900	0.17437400
C	0.84041400	1.91897900	0.23930300
C	-3.01191600	-0.25305400	0.20067800
C	-4.25157900	-0.60028700	-0.35051200
C	-4.66111800	-1.92353500	-0.47426500
C	-3.80201300	-2.90463300	0.02338200
C	-2.54055700	-2.64007700	0.56375900
C	-2.09495700	-1.28204000	0.55492000
C	-3.50638900	3.53161700	0.40424500
C	-3.72158600	2.14152300	0.40204500
C	-2.70214100	1.18419900	0.31966600
H	-4.74814300	1.78120900	0.48269100
H	-1.96190200	5.05344300	0.29233900

H	0.61422600	5.33564200	0.02790200
H	4.08651800	2.77790100	-0.02851800
H	-1.06414400	-1.18954200	1.67629800
H	-4.13561500	-3.94193400	-0.01776700
H	-4.88845300	0.19742600	-0.72654300
Si	-0.04380500	-0.78319700	0.59005200
I	0.57104100	-1.86448800	-1.57409700
C	2.94536000	0.45655000	0.25149400
C	3.73122700	0.03225400	-0.82561100
C	2.88223800	-0.32556400	1.41047900
C	4.44414000	-1.16580300	-0.77136200
H	3.74941700	0.65645300	-1.71900000
C	3.59321000	-1.52949900	1.50686500
H	2.30732800	0.04872000	2.25738600
C	4.35656700	-1.92199800	0.40502100
H	4.91558700	-2.85840700	0.46431400
C	-4.71114100	4.47317100	0.50580500
C	-5.44557400	4.21799200	1.83111800
H	-6.31770600	4.88530000	1.92477600
H	-5.80887400	3.18183300	1.90457400
H	-4.78000000	4.39989400	2.68899500
C	-5.66685900	4.21334000	-0.66930600
H	-6.04578000	3.18007700	-0.66961000
H	-6.53806700	4.88608500	-0.61495500
H	-5.16035700	4.38418000	-1.63178000
C	-4.29075300	5.94373100	0.46427200
H	-3.62567300	6.20181300	1.30265000
H	-3.77420100	6.19394500	-0.47518000
H	-5.17983700	6.58893300	0.53605400
C	3.42252600	5.41130400	-0.19124600
C	4.39043400	5.45429500	1.00140600
H	5.08432800	6.30519900	0.90529400
H	3.84000700	5.56525800	1.94846800
H	4.99412600	4.53717100	1.07130300
C	2.66676300	6.74041300	-0.24573000
H	2.09453500	6.92245900	0.67701600
H	3.37942200	7.57132800	-0.36293700
H	1.96950400	6.77851200	-1.09687000
C	4.22046600	5.25570000	-1.49521800
H	4.81926400	4.33264600	-1.50051100
H	3.54645500	5.22237500	-2.36517000
H	4.91222500	6.10331400	-1.62832000
C	-5.99518900	-2.32613700	-1.10354000
C	-6.79044800	-1.11454300	-1.59455300
H	-7.73660500	-1.44958000	-2.04608700
H	-7.04259500	-0.42844900	-0.77121000
H	-6.24026400	-0.54692500	-2.36056900
C	-6.84107500	-3.07217500	-0.05948300
H	-7.04205000	-2.43277400	0.81393100
H	-7.80771600	-3.37157300	-0.49495200
H	-6.34124600	-3.98404600	0.29981800
C	-5.72171500	-3.24767500	-2.30279500
H	-5.11018900	-2.73485900	-3.06089400
H	-5.18733200	-4.16185900	-2.00417100
H	-6.66952800	-3.55288000	-2.77416900
C	-1.82590500	-3.89151900	1.13195900

C	-0.51154200	-3.64681200	1.87244600
H	0.27873200	-3.23984600	1.22566000
H	-0.63052000	-2.97333600	2.73432100
H	-0.14226300	-4.60873200	2.26157200
C	-1.54397200	-4.88365700	-0.00967600
H	-0.86007300	-4.44520300	-0.75035100
H	-1.07534300	-5.79533000	0.39396800
H	-2.46326600	-5.18380700	-0.53311700
C	-2.76742300	-4.55055500	2.16205500
H	-3.00593900	-3.85156000	2.97869800
H	-3.71598900	-4.88731100	1.72163700
H	-2.27693900	-5.43339000	2.60099200
C	3.59339600	-2.38140400	2.78021500
C	2.52842400	-1.91252600	3.77484700
H	2.50380800	-2.58756200	4.64416300
H	2.73726400	-0.90023400	4.15293800
H	1.52790500	-1.90493000	3.31558300
C	3.31377900	-3.85102100	2.43226500
H	4.09108000	-4.27924000	1.78270700
H	3.28183700	-4.45963300	3.34999000
H	2.34929800	-3.95765000	1.91551900
C	4.97557600	-2.27089900	3.44278400
H	5.01268000	-2.87345700	4.36511700
H	5.77198300	-2.62749500	2.77161900
H	5.20221700	-1.22611900	3.70578100
C	5.28215800	-1.68446100	-1.94323100
C	4.72079200	-3.04146500	-2.39614500
H	3.66861300	-2.94438400	-2.70496300
H	5.29869700	-3.43202600	-3.24936600
H	4.76259900	-3.79075200	-1.59151500
C	6.74112700	-1.85215100	-1.49251200
H	6.83333400	-2.56187500	-0.65677100
H	7.35898300	-2.23189500	-2.32222700
H	7.16321400	-0.89000500	-1.16311600
C	5.25211700	-0.72909600	-3.13790000
H	4.23058300	-0.59295100	-3.52453400
H	5.65713100	0.26153400	-2.87970300
H	5.86620600	-1.13588800	-3.95607400

4.7.19 RSi^+

1 1

C	1.71010000	-1.19720000	-0.10352200
N	0.93128200	-0.03422000	-0.01876900
C	3.98073500	-1.96574300	-0.11932000
C	3.08331400	-0.90175700	-0.07074500
C	3.17577300	0.55432100	0.03140400
C	4.20025600	1.49632600	0.08595800
C	3.90315000	2.86898300	0.17215100
C	2.55567300	3.28047900	0.20570700
C	1.50754300	2.35860500	0.15726000
C	1.85096000	1.02064800	0.06873400
C	-0.28305200	-2.57158100	-0.19856100
C	-0.97495000	-2.30015900	-1.39630400
C	-2.37988500	-2.31877400	-1.44040100

C	-3.06099900	-2.59740800	-0.25013500
C	-2.41580400	-2.81700100	0.97289400
C	-1.01081900	-2.77929300	0.98434400
C	-3.23469100	-3.15024500	2.22106300
C	-3.16704900	-2.03974100	-2.72003100
C	4.52965200	-4.43645200	-0.24132200
C	5.05788200	3.87642200	0.22916100
C	0.04715300	2.62366400	0.19474200
C	-0.66231400	2.41576500	1.39335700
C	-2.06135700	2.55217100	1.44696000
C	-2.72896200	2.87945400	0.25948800
C	-2.06575800	3.06103400	-0.96130900
C	-0.66774800	2.90514600	-0.97884200
C	-2.79377000	2.37009000	2.77569900
C	-2.79633100	3.47828000	-2.23812200
C	3.51197100	-3.29034200	-0.19234100
C	2.12312900	-3.52801300	-0.21734900
C	1.19911100	-2.48106300	-0.17577000
C	5.41705300	-4.26798800	-1.48497700
H	5.97418600	-3.31937700	-1.47013400
H	6.15469900	-5.08367300	-1.53943400
H	4.81489600	-4.29086200	-2.40629000
C	5.39922100	-4.39272000	1.02534100
H	6.13604300	-5.21078600	1.00922600
H	5.95677800	-3.44829500	1.11244200
H	4.78407600	-4.50587300	1.93129900
C	3.85489100	-5.80794400	-0.31399300
H	3.22300200	-6.00362200	0.56612800
H	3.23690800	-5.91610700	-1.21870700
H	4.62233100	-6.59535600	-0.34667300
C	-4.39781800	-2.15980500	2.38111500
H	-5.10185700	-2.19610900	1.53724400
H	-4.97054200	-2.40038500	3.28935600
H	-4.03408300	-1.12585500	2.47599200
C	-2.38408100	-3.10823200	3.49287600
H	-1.58856600	-3.86828100	3.48667400
H	-1.92000500	-2.12089800	3.64348800
H	-3.01749100	-3.31370200	4.36814300
C	-3.79487800	-4.57221700	2.04276100
H	-4.39268400	-4.85745500	2.92226300
H	-4.44318400	-4.64395000	1.15629700
H	-2.98351700	-5.30706100	1.92842400
C	5.92050700	3.58326800	1.46708500
H	6.35290400	2.57196800	1.43992500
H	6.75576200	4.29825800	1.52669700
H	5.32899600	3.67234400	2.39127100
C	4.56234800	5.32136400	0.31934800
H	3.95766600	5.60524600	-0.55584200
H	3.96579100	5.49681100	1.22789900
H	5.42333800	6.00496100	0.35701700
C	5.91119200	3.73687500	-1.04165200
H	6.74570000	4.45487600	-1.02016200
H	6.34439600	2.73045500	-1.14101400
H	5.31268900	3.93682900	-1.94374500
C	-2.43708900	4.94880200	-2.51619700
H	-1.35429600	5.08137200	-2.66233300

H	-2.94565200	5.29698900	-3.42852900
H	-2.74824900	5.59726500	-1.68313300
C	-4.31556100	3.35653000	-2.09816900
H	-4.71816800	4.03302100	-1.32951700
H	-4.79655800	3.62887000	-3.04892900
H	-4.62303600	2.32816900	-1.85197300
C	-3.90839500	-3.32094900	-3.13383400
H	-4.60170900	-3.66313400	-2.35089200
H	-4.49692400	-3.14056100	-4.04659700
H	-3.20113400	-4.13855400	-3.34004300
C	-4.18208800	-0.91771800	-2.44596700
H	-4.94281600	-1.21478300	-1.70936100
H	-3.67928300	-0.01675100	-2.06146000
H	-4.71069600	-0.64788200	-3.37290900
C	-2.25475600	-1.60345900	-3.86810900
H	-1.68717900	-0.69269800	-3.61928700
H	-1.53899900	-2.39036000	-4.15013300
H	-2.85998300	-1.38123000	-4.75912200
C	-2.34674500	2.61069300	-3.42284700
H	-1.27219100	2.70800700	-3.63520700
H	-2.56618300	1.54750700	-3.24486800
H	-2.88334200	2.91593200	-4.33369200
C	-4.29867500	2.17402700	2.57277500
H	-4.78201300	1.98696700	3.54283000
H	-4.78071900	3.06413300	2.14211900
H	-4.51259000	1.31411200	1.91854900
C	-2.55806500	3.63268300	3.62207000
H	-2.95517800	4.52689100	3.11826500
H	-3.06193700	3.53724500	4.59646700
H	-1.48604800	3.79770900	3.80939100
C	-2.24076100	1.14547500	3.52031300
H	-2.30503200	0.23977300	2.89674200
H	-1.19272900	1.27432200	3.82731000
H	-2.82305700	0.96671900	4.43658500
H	1.74458300	-4.54826100	-0.27477500
H	5.05419000	-1.76751500	-0.09422400
H	5.24003800	1.16423400	0.05762200
H	2.30941300	4.33960100	0.27691300
H	-0.10588700	3.03700700	-1.90559400
H	-3.81094100	2.98537300	0.28375100
H	-0.09078300	2.18964500	2.29626400
H	-0.45451400	-2.94863700	1.90674500
H	-4.15104000	-2.63546800	-0.27473000
H	-0.38874900	-2.12634500	-2.30016200
Si	-0.81807800	0.07678100	-0.02180000

4.7.20 RSi^{+}-I

1 1

C	2.37542400	0.08103700	-0.01648800
N	1.06268700	-0.39887500	-0.03193200
C	4.65417900	-0.56364500	0.05864500
C	3.31049500	-0.94759000	0.02577700
C	2.52132300	-2.17836600	0.04477900
C	2.84493400	-3.53479900	0.12610600

C	1.82936900	-4.49606300	0.15342200
C	0.48717800	-4.06633100	0.06754800
C	0.12069700	-2.72467700	-0.03282700
C	1.16123200	-1.79845600	-0.01291800
C	1.41188300	2.21593200	0.05466100
C	1.23900100	3.57332500	0.12409600
C	0.03257600	4.34602000	0.18877700
C	-1.27388300	3.92294400	0.41608400
C	-2.01542300	2.71046700	0.61737800
C	-1.59790500	1.39790300	0.41955600
C	5.01155600	0.80263300	0.05317700
C	4.00987500	1.79307900	0.03337000
C	2.65075700	1.44998800	0.00917600
H	4.29767800	2.84643500	0.04789300
H	5.43046400	-1.32764000	0.09336700
H	3.89296500	-3.82961000	0.17339600
H	-0.31395600	-4.80865000	0.05047200
H	-2.32260300	0.58496800	0.53103700
H	-1.94141600	4.77466500	0.48646200
H	2.16021100	4.16376500	0.11629600
Si	0.06730900	0.98608600	0.06062300
C	-1.27582000	-2.26298300	-0.22130900
C	-1.60646700	-1.59757900	-1.40855100
C	-2.26301900	-2.48033000	0.74351100
C	-2.91394700	-1.16858800	-1.66311500
H	-0.82777800	-1.46742700	-2.16508400
C	-3.57900500	-2.04604900	0.53876700
H	-1.98749000	-3.00451000	1.66065500
C	-3.87546500	-1.39805800	-0.67045700
H	-4.89933400	-1.07776100	-0.85094900
C	-3.24291800	-0.50977700	-3.00707800
C	-4.66093300	-2.35001300	1.58020400
C	-2.39372800	0.76043300	-3.17473200
H	-2.60924000	1.49024900	-2.37926100
H	-1.31499300	0.53780400	-3.15104000
H	-2.60692100	1.23949900	-4.14297600
C	-4.72007000	-0.12671200	-3.12098400
H	-4.90845400	0.33618500	-4.10104900
H	-5.37671500	-1.00626000	-3.04237300
H	-5.02244000	0.59938400	-2.35169500
C	-2.91429000	-1.49827000	-4.13770700
H	-3.50062300	-2.42371900	-4.03293800
H	-3.15281300	-1.05200300	-5.11573300
H	-1.84959200	-1.77476400	-4.14892900
C	-5.92082000	-1.50703700	1.36050300
H	-6.41930900	-1.74055200	0.40803200
H	-6.64786300	-1.71005700	2.16068200
H	-5.69666500	-0.42857600	1.37705100
C	-5.02844200	-3.83813600	1.45402300
H	-4.15513200	-4.48238900	1.63795300
H	-5.80797700	-4.10577700	2.18470400
H	-5.40963300	-4.06674400	0.44708500
C	-4.14373600	-2.07571300	2.99955500
H	-3.29000500	-2.71431700	3.26783100
H	-3.83294200	-1.02639500	3.11927000
H	-4.93840700	-2.27597700	3.73411200

C	2.12168500	-5.99635200	0.25364300
C	6.48041800	1.24864400	0.07901400
C	1.56928600	-6.70273900	-0.99480300
H	1.77387700	-7.78337100	-0.94160100
H	0.48079300	-6.57698800	-1.09409900
H	2.03912900	-6.31148200	-1.91024700
C	1.44014200	-6.55829300	1.51145300
H	0.34801600	-6.42727700	1.48354900
H	1.64093700	-7.63706900	1.60264600
H	1.81713800	-6.06343000	2.41974100
C	3.62137800	-6.28398100	0.34770900
H	4.16418600	-5.93374400	-0.54375100
H	4.07404400	-5.81951800	1.23737500
H	3.78583400	-7.36892700	0.42476800
C	7.44485200	0.06085000	0.10997500
H	7.30603000	-0.56030400	1.00822400
H	7.33894200	-0.58000600	-0.77884600
H	8.48134200	0.42868500	0.12508500
C	6.77608400	2.07688100	-1.18154400
H	6.15120800	2.98091200	-1.23935500
H	7.82781700	2.40254000	-1.18485200
H	6.59953500	1.48606900	-2.09343900
C	6.72893700	2.10309100	1.33222400
H	7.78086200	2.42587500	1.36963000
H	6.10649000	3.01049000	1.34659000
H	6.51494000	1.53246400	2.24895600
C	0.27933500	5.86761600	0.03614300
C	-3.49141000	2.86441500	1.07059300
C	-4.40380100	2.33723800	-0.04764900
H	-4.21154500	1.27833500	-0.26887800
H	-5.45767600	2.42851300	0.25667100
H	-4.26973000	2.91185800	-0.97708900
C	-3.69726600	2.03231300	2.34820200
H	-4.73969400	2.12637500	2.68764100
H	-3.50141800	0.96323800	2.18759800
H	-3.04275400	2.38137300	3.16143400
C	-3.90273600	4.30559800	1.38656000
H	-3.27626300	4.75791100	2.17054500
H	-3.88705000	4.95754400	0.49998600
H	-4.93760100	4.30426900	1.75803100
C	0.99817600	6.12018100	-1.30238300
H	1.17560700	7.19858700	-1.43025600
H	1.97655700	5.62200800	-1.36676300
H	0.38682100	5.77483400	-2.15017500
C	-1.00346100	6.70313500	0.03143000
H	-1.68688900	6.41388400	-0.78174900
H	-1.54726800	6.64952100	0.98682500
H	-0.74161300	7.75911800	-0.12618900
C	1.15095100	6.35631200	1.20755400
H	2.13773100	5.87169600	1.24208100
H	1.32641100	7.43847000	1.11179500
H	0.65204200	6.17765400	2.17224600

4.7.21 RSi⁺-I-TS

1 1

C	-1.29954700	1.74575500	-0.56661100
N	-1.09455800	0.40017400	-0.46737100
C	-2.93235000	3.45137500	-0.51240400
C	-2.65691900	2.07518800	-0.51903600
C	-3.34055200	0.78518300	-0.29926100
C	-4.63499100	0.30805400	-0.07288900
C	-4.85865800	-1.04569600	0.25091900
C	-3.76582400	-1.92893300	0.39127000
C	-2.45172600	-1.49497000	0.18155600
C	-2.30671100	-0.17280900	-0.20926500
C	1.04895300	1.84085800	-0.09385300
C	2.20826100	2.51959100	0.39869200
C	3.47655700	2.01427300	0.44885900
C	3.84308900	0.82478600	-0.28711000
C	3.09674100	0.16602900	-1.22413500
C	1.65563100	0.32061100	-1.36802000
C	-1.87539000	4.38320600	-0.48330200
C	-0.52660600	3.94887800	-0.39757700
C	-0.20259600	2.59011500	-0.41932600
H	0.26418600	4.69434800	-0.28968900
H	-3.96396400	3.80190500	-0.49422300
H	-5.48283900	0.99405800	-0.12839800
H	-3.93583000	-2.95769800	0.70949000
H	1.25055900	0.28615000	-2.39020700
H	4.89702600	0.55620200	-0.23543300
H	2.03240100	3.54090600	0.74238000
Si	0.52760500	0.03410300	-0.00341400
C	-1.18203300	-2.23998300	0.34763900
C	-0.07856500	-1.63530100	1.11080000
C	-0.96409700	-3.45934400	-0.25564300
C	1.11181400	-2.40764200	1.36726100
H	-0.41513400	-0.98807000	1.93648500
C	0.27102700	-4.14120800	-0.14237000
H	-1.77144000	-3.89071200	-0.84968500
C	1.27554000	-3.60127000	0.67473800
H	2.19947100	-4.15925900	0.80667400
C	-2.14136800	5.89624900	-0.49989200
C	4.61719400	2.76364200	1.14688500
C	3.74966900	-0.75967200	-2.26715000
C	5.21299600	-1.06416600	-1.94246800
H	5.61677700	-1.76039700	-2.69248600
H	5.84036100	-0.16116000	-1.96678900
H	5.32564000	-1.53921900	-0.95539200
C	2.98737900	-2.09298900	-2.33819200
H	3.00600500	-2.60930700	-1.36544700
H	1.93364500	-1.95399600	-2.62418200
H	3.44857100	-2.75821200	-3.08464800
C	3.69204300	-0.05657200	-3.63419500
H	4.24285500	0.89531800	-3.60800200
H	4.14923400	-0.69648100	-4.40472300
H	2.66152700	0.15507500	-3.95660500
C	4.13410100	4.03691800	1.84354000
H	4.97400000	4.51096900	2.37262400

H	3.74414700	4.77585300	1.12696300
H	3.35017700	3.82498100	2.58690900
C	5.69506500	3.15488200	0.12208400
H	6.50578900	3.70989100	0.61879700
H	6.14873500	2.28013300	-0.36661800
H	5.27234400	3.79902100	-0.66395400
C	5.22672000	1.83720000	2.21277600
H	4.48130000	1.57844800	2.98077200
H	5.61165500	0.90015300	1.78251800
H	6.06841600	2.33885200	2.71475700
C	-1.57663600	6.53238600	0.78033700
H	-1.76794800	7.61680700	0.78359900
H	-2.04810300	6.10030800	1.67647400
H	-0.48898600	6.39201000	0.87032300
C	-3.63484000	6.22158500	-0.57449300
H	-3.77388900	7.31259800	-0.59972500
H	-4.10025400	5.81046800	-1.48347500
H	-4.18420600	5.84281600	0.30116700
C	-1.45457500	6.51118200	-1.72992600
H	-0.36520800	6.35603800	-1.71511800
H	-1.84398100	6.07007400	-2.66032800
H	-1.63437300	7.59711400	-1.76559100
C	-7.11938600	-1.26902600	-0.79954700
H	-7.14879200	-0.20336300	-1.07116500
H	-6.69566500	-1.82134200	-1.65244700
H	-8.15931500	-1.60294000	-0.65915300
C	-6.30269100	-1.51669800	0.47869200
C	-6.38048600	-3.00814300	0.81308800
H	-5.85023500	-3.25089900	1.74710800
H	-7.43180000	-3.30115600	0.95082300
H	-5.96999500	-3.63297300	0.00454200
C	-6.91768300	-0.72964200	1.64670200
H	-7.95391300	-1.05710300	1.82457100
H	-6.34589300	-0.88801800	2.57410300
H	-6.94361800	0.35210800	1.44810100
C	2.13081700	-1.96920200	2.41232600
C	0.46731200	-5.44299100	-0.90544100
C	0.30300400	-5.14587100	-2.40830100
H	0.44593600	-6.07353500	-2.98273300
H	-0.69587100	-4.75542700	-2.65130200
H	1.05024000	-4.41455600	-2.75215400
C	1.85084200	-6.05412300	-0.67731500
H	2.01913300	-6.31754800	0.37806900
H	1.94158500	-6.98101400	-1.26153400
H	2.65764000	-5.38096600	-1.00599700
C	-0.60321600	-6.45149500	-0.45103100
H	-0.47061400	-7.39714200	-0.99779000
H	-0.51670000	-6.66647300	0.62484700
H	-1.62531500	-6.09755900	-0.64886300
C	3.56687000	-2.22317100	1.93282200
H	4.27423300	-1.87728900	2.70078600
H	3.77625200	-3.28819800	1.76088400
H	3.77653500	-1.66648500	1.00718600
C	2.00802600	-0.49131800	2.78982400
H	2.23972400	0.18327500	1.94466200
H	1.02252800	-0.22443500	3.19956500

H	2.74595200	-0.25513800	3.56984700
C	1.83911000	-2.82704700	3.66256800
H	1.95392100	-3.89973600	3.44889300
H	2.54490500	-2.55929800	4.46365900
H	0.81802000	-2.65942200	4.03790600

4.7.22 RSi⁺-II

1 1

C	0.76204300	1.71485600	0.18594700
N	-0.30416900	0.81113400	0.21470500
C	1.16719000	4.09781300	0.16485400
C	0.27307000	3.03453500	0.17336400
C	-1.18041400	2.93989200	0.14482400
C	-2.21582500	3.86972400	0.09964800
C	-3.54903000	3.43859300	0.01370300
C	-3.80745700	2.05739200	-0.07564900
C	-2.79013000	1.10024500	-0.04228200
C	-1.49105400	1.57073000	0.12078100
C	2.61030900	0.00363000	0.17599200
C	3.96847200	-0.22500300	-0.07451100
C	4.55185100	-1.48111300	0.02694300
C	3.73847600	-2.54365000	0.45031800
C	2.37618700	-2.41531400	0.68776300
C	1.77266200	-1.12923500	0.48524900
C	2.54776300	3.84139600	0.16471900
C	2.98391700	2.51007500	0.16246900
C	2.12251300	1.39489700	0.16449100
H	4.05874100	2.33611600	0.18677200
H	0.78698700	5.11911800	0.16313800
H	-1.98254900	4.93606500	0.12381900
H	-4.82914400	1.70156300	-0.20861600
H	-1.10398000	-1.67136500	1.03304800
H	4.20201700	-3.51426400	0.59409700
H	4.59319800	0.61543900	-0.37116400
Si	-0.02033600	-0.84832600	0.48020100
C	-4.67284100	4.47976300	-0.01393400
C	3.59062000	4.96508000	0.17356400
C	2.93857500	6.34931200	0.16060000
H	2.32429100	6.50650500	-0.73941300
H	2.30834600	6.51587700	1.04773800
H	3.71818600	7.12522900	0.16385100
C	4.45052400	4.84554800	1.44183300
H	4.99113300	3.88794100	1.48739600
H	5.20188100	5.64986400	1.46881300
H	3.83180100	4.92641400	2.34866100
C	4.48213700	4.83828000	-1.07196100
H	5.23282400	5.64352500	-1.08580400
H	5.02580900	3.88165700	-1.09680000
H	3.88640700	4.91160600	-1.99471900
C	-4.61352500	5.31159800	1.27730400
H	-3.65641600	5.84395400	1.38250600
H	-5.41442000	6.06715500	1.27950600
H	-4.74416500	4.67395400	2.16513900
C	-4.48067500	5.39812100	-1.23138700

H	-4.51012300	4.82269600	-2.16940100
H	-5.28240000	6.15188500	-1.26854500
H	-3.52234600	5.93743500	-1.19614400
C	-6.05721100	3.83491700	-0.10899000
H	-6.17900900	3.25304700	-1.03556900
H	-6.26301700	3.17453100	0.74754900
H	-6.82954500	4.61817900	-0.11190200
C	-2.96397300	-0.35338400	-0.26864300
C	-3.58530800	-1.19395200	0.64918700
C	-2.31811300	-0.90779200	-1.38816400
C	-3.54962700	-2.59120500	0.48515200
H	-4.06285300	-0.75208500	1.52647900
C	-2.30532700	-2.29078600	-1.61549000
H	-1.87541100	-0.22549900	-2.11834400
C	-2.91172400	-3.10783300	-0.64936200
H	-2.89051400	-4.18585500	-0.79092500
C	1.59387800	-3.63894500	1.20451800
C	6.03589200	-1.65861300	-0.29083500
C	0.94159800	-3.30179400	2.55775900
H	0.22003100	-2.47401700	2.51165400
H	0.39787500	-4.17740900	2.94455200
H	1.70716000	-3.02258800	3.29703300
C	0.55173300	-4.08909500	0.17177400
H	-0.18825400	-3.31945500	-0.08884900
H	1.04433400	-4.39868400	-0.76194500
H	-0.02219000	-4.94695800	0.55578200
C	2.50688100	-4.84656100	1.45491500
H	2.99049400	-5.20308800	0.53346300
H	3.28677000	-4.62991500	2.19985000
H	1.90236100	-5.67800300	1.84588800
C	6.85953500	-0.77031400	0.65663900
H	7.93314800	-0.88959000	0.44494000
H	6.61678700	0.29656200	0.54086600
H	6.68741400	-1.04484400	1.70858200
C	6.28183900	-1.23257300	-1.74779800
H	6.01652100	-0.17923400	-1.92273900
H	7.34681700	-1.35181300	-1.99934900
H	5.69602100	-1.84972600	-2.44608200
C	6.49579700	-3.10823900	-0.12245800
H	6.37835100	-3.46178400	0.91344500
H	5.95090000	-3.79370400	-0.78970500
H	7.56350900	-3.18703400	-0.37394800
C	-4.17798900	-3.49056400	1.55143500
C	-1.70567800	-2.83837800	-2.91265000
C	-5.66519300	-3.13349700	1.70058700
H	-6.13279400	-3.77150200	2.46615700
H	-5.81207700	-2.08756900	2.00797300
H	-6.20471000	-3.28690700	0.75363600
C	-2.61066500	-2.38756800	-4.07304500
H	-2.66383700	-1.29094800	-4.14597900
H	-2.22233500	-2.77124700	-5.02928100
H	-3.63548700	-2.76723200	-3.94387800
C	-1.63356300	-4.36689300	-2.91787200
H	-2.63195100	-4.82527700	-2.85804900
H	-1.17443200	-4.71237600	-3.85578300
H	-1.02545100	-4.75625300	-2.08777800

C	-0.29169400	-2.27563600	-3.12653900
H	-0.28451400	-1.17782600	-3.19859900
H	0.38790400	-2.57062900	-2.31141400
H	0.13216100	-2.66242600	-4.06574900
C	-3.45032600	-3.25435300	2.88564200
H	-2.38059600	-3.50534700	2.80088800
H	-3.52935100	-2.20949200	3.22134100
H	-3.88389400	-3.88903600	3.67384900
C	-4.06579900	-4.97468300	1.19740400
H	-4.53121200	-5.57965200	1.98934500
H	-4.58212500	-5.21373100	0.25524800
H	-3.01711600	-5.29983500	1.11089200

4.7.23 RSi^{+}-II-TS

1 1

C	-0.95204500	1.67414600	-0.12586800
N	0.13387100	0.84745600	-0.02379400
C	-1.49811900	4.02975900	-0.15389000
C	-0.54583400	3.03610400	-0.13292200
C	0.90667100	3.02174100	-0.04355600
C	1.88251300	4.01506300	-0.04091600
C	3.23608000	3.65918800	0.04210700
C	3.56723000	2.29217300	0.14403300
C	2.61373400	1.27486400	0.17010300
C	1.27720100	1.66891100	0.05127100
C	-2.70924800	-0.09100200	-0.09594600
C	-4.02859200	-0.43262400	0.25665800
C	-4.49603400	-1.72579500	0.13626300
C	-3.62568800	-2.70203300	-0.43236400
C	-2.30984400	-2.47640100	-0.74638700
C	-1.75823700	-1.13983200	-0.46490500
C	-2.88333600	3.69781000	-0.17522300
C	-3.24750400	2.36123500	-0.14102200
C	-2.30768100	1.29110700	-0.11551800
H	-4.30444000	2.10883900	-0.18237000
H	-1.18798100	5.07675900	-0.14317400
H	1.58528500	5.06110000	-0.11342800
H	4.61530800	1.99811300	0.23191200
H	-0.78376400	-1.54710300	0.73797900
H	-4.04809300	-3.68556000	-0.62541700
H	-4.68753400	0.34350300	0.63950100
Si	0.11239900	-0.89938700	-0.43047200
C	2.93755500	-0.16252400	0.32010900
C	3.48101800	-0.88457500	-0.75407800
C	2.58841400	-0.82413400	1.49866200
C	3.65481200	-2.26764800	-0.66613000
H	3.74078000	-0.34294100	-1.66409100
C	2.75771600	-2.21048200	1.62717900
H	2.17078300	-0.23902800	2.32167700
C	3.28231000	-2.89962600	0.53119200
H	3.41426700	-3.97897900	0.60436400
C	-5.90145000	-2.14103700	0.54603400
C	-1.48164400	-3.59755600	-1.40587400
C	-0.79076600	-3.06388800	-2.67320600

H	-0.00983200	-2.31775800	-2.44541200
H	-0.29457100	-3.88893900	-3.20593200
H	-1.51332100	-2.59567200	-3.35916400
C	-0.43633000	-4.17815700	-0.43313600
H	0.37200400	-3.47595300	-0.18150800
H	-0.91189100	-4.50665900	0.50404000
H	0.04297300	-5.05463000	-0.89556100
C	-2.37902400	-4.76157300	-1.84694300
H	-2.84267100	-5.27989300	-0.99423200
H	-3.17304500	-4.43478600	-2.53562300
H	-1.76657000	-5.50549400	-2.37648700
C	-6.69824900	-0.97994400	1.14264800
H	-7.69732000	-1.33181300	1.43805800
H	-6.21545700	-0.56956800	2.04287600
H	-6.84197600	-0.16353400	0.41811200
C	-6.64748700	-2.66489300	-0.69448000
H	-6.72408800	-1.88739800	-1.46970600
H	-6.15778500	-3.54338600	-1.13968200
H	-7.66780600	-2.96580700	-0.41198800
C	-5.79393200	-3.25896200	1.59869400
H	-5.26032700	-2.90939100	2.49565400
H	-6.80154800	-3.57885000	1.90510000
H	-5.26869800	-4.14543900	1.21338000
C	-3.90986000	4.82884100	-0.23463100
C	4.35885900	4.69910600	0.03743300
C	-3.67334600	5.64949900	-1.51324700
H	-3.79106300	5.02535800	-2.41224200
H	-4.40102500	6.47337300	-1.57241700
H	-2.66839400	6.09684000	-1.54121400
C	-5.34702300	4.30552500	-0.25099300
H	-6.04726900	5.15249700	-0.29221800
H	-5.54582500	3.67467300	-1.13125500
H	-5.58556500	3.72892200	0.65631300
C	-3.73217600	5.72729100	1.00086700
H	-3.89076200	5.15956700	1.93046800
H	-2.73087200	6.18090800	1.04772900
H	-4.46293100	6.55014600	0.97496800
C	3.82060100	6.12558100	-0.08986400
H	3.26155600	6.27240100	-1.02695700
H	3.16713200	6.39593400	0.75395700
H	4.65887100	6.83771500	-0.09420700
C	5.29280000	4.42355000	-1.15219300
H	6.10706100	5.16430400	-1.17347600
H	5.75489200	3.42688200	-1.09350300
H	4.74792400	4.48871100	-2.10655000
C	5.14840300	4.59226900	1.35235800
H	4.49798100	4.77896100	2.22065100
H	5.60674200	3.60043500	1.48053800
H	5.95971700	5.33643000	1.36794100
C	4.22201700	-3.10446400	-1.81550300
C	2.36872600	-2.90456600	2.93519000
C	4.55388400	-2.24947600	-3.03984400
H	3.66239300	-1.74251900	-3.44089400
H	5.31431300	-1.48648300	-2.81359700
H	4.95760400	-2.88722100	-3.84020000
C	5.50783900	-3.80127800	-1.34329800

H	5.32433600	-4.47059700	-0.48964800
H	5.93298000	-4.40927400	-2.15699800
H	6.26562500	-3.06430800	-1.03646000
C	3.18358600	-4.15966700	-2.22821300
H	3.57184600	-4.77434600	-3.05515400
H	2.93305300	-4.83880100	-1.39948500
H	2.25134000	-3.68139500	-2.56639800
C	0.88270100	-2.64416000	3.22952900
H	0.59317800	-3.11674000	4.18104000
H	0.65834600	-1.56992100	3.31264200
H	0.24677400	-3.06684000	2.43548900
C	2.58598200	-4.41766400	2.87283600
H	3.64241800	-4.67456800	2.70229300
H	2.28774000	-4.87528600	3.82782600
H	1.98297900	-4.88675200	2.07951300
C	3.22660800	-2.33179200	4.07446500
H	2.96994200	-2.81810500	5.02856900
H	4.29724600	-2.50002400	3.88270900
H	3.07348800	-1.24944800	4.19966000

4.7.24 RSi⁺, one arene

1 1

C	0.60838400	0.48546900	-0.00001500
N	0.49683100	-0.91203200	-0.00015400
C	2.16785900	2.28788100	0.00018700
C	1.93633600	0.91425800	0.00011100
C	2.73452300	-0.30754200	-0.00001100
C	4.10445900	-0.54718900	0.00000100
C	4.59731800	-1.86052000	-0.00019800
C	3.66868900	-2.91683200	-0.00040000
C	2.28949200	-2.70664000	-0.00040100
C	1.83078600	-1.39751400	-0.00018100
C	-1.78289100	0.61306300	-0.00001500
C	-2.36528100	0.18310500	1.21636000
C	-3.60065400	-0.50112000	1.23353600
C	-4.19530300	-0.79548100	0.00004600
C	-3.60089800	-0.50081000	-1.23346200
C	-2.36551300	0.18345100	-1.21636400
C	-4.27848400	-0.92825700	-2.53198700
C	-4.27794000	-0.92898400	2.53207600
C	1.38961800	4.69988400	0.00023400
C	6.11062300	-2.09648100	-0.00018000
C	1.09027400	3.19587000	0.00019700
C	-0.23061000	2.70398400	0.00011900
C	-0.48370300	1.33027000	0.00001200
C	2.20167500	5.05080700	1.25727800
H	3.16173900	4.51485300	1.29365300
H	2.42497100	6.12871200	1.27591600
H	1.64205900	4.80305800	2.17235400
C	2.20151100	5.05083300	-1.25691300
H	2.42483100	6.12873400	-1.27555600
H	3.16154700	4.51483500	-1.29341700
H	1.64176300	4.80311500	-2.17191600
C	0.11361700	5.54448700	0.00034800

H	-0.50260900	5.36181100	-0.89368600
H	-0.50249000	5.36171900	0.89444300
H	0.37839100	6.61201300	0.00037200
C	-4.43371000	-2.45868900	-2.52511400
H	-5.05635800	-2.81106500	-1.68944200
H	-4.91508800	-2.78908200	-3.45772300
H	-3.45444000	-2.95712100	-2.45321600
C	-3.46910300	-0.52129000	-3.76452700
H	-3.34583500	0.57020300	-3.83812200
H	-2.47273200	-0.99001900	-3.77980700
H	-3.99463600	-0.84990300	-4.67250900
C	-5.66263900	-0.26122600	-2.60484600
H	-6.16844900	-0.55868600	-3.53563200
H	-6.31287100	-0.55667900	-1.76815500
H	-5.57768400	0.83601600	-2.60028100
C	6.71864200	-1.45296600	-1.25675600
H	6.54386200	-0.36714500	-1.29154000
H	7.80784100	-1.61289200	-1.27591000
H	6.29351100	-1.89301400	-2.17187500
C	6.46387900	-3.58522600	-0.00048700
H	6.07850500	-4.10040800	0.89291100
H	6.07859700	-4.10001400	-0.89415100
H	7.55736900	-3.70474500	-0.00045500
C	6.71852000	-1.45351000	1.25673500
H	7.80771500	-1.61345500	1.27593600
H	6.54374600	-0.36770200	1.29196300
H	6.29328700	-1.89394300	2.17162200
C	-5.66259100	-0.26304300	2.60502700
H	-6.31270800	-0.55902000	1.76843300
H	-6.16808500	-0.56091800	3.53584900
H	-5.57852200	0.83426700	2.60046900
C	-4.43201100	-2.45955100	2.52513700
H	-5.05434500	-2.81229900	1.68938600
H	-3.45236000	-2.95723500	2.45325500
H	-4.91316900	-2.79034900	3.45771700
C	-3.46883900	-0.52139700	3.76460300
H	-2.47217200	-0.98948400	3.77998600
H	-3.34627900	0.57018500	3.83805400
H	-3.99418200	-0.85027300	4.67259900
H	-1.07297000	3.39495900	0.00014300
H	3.19319900	2.66307500	0.00025700
H	4.79335300	0.30017000	0.00017200
H	4.02473700	-3.94679300	-0.00055600
H	-1.87265300	0.46938500	-2.14691400
H	-5.15363900	-1.31712100	0.00007500
H	-1.87208000	0.46868800	2.14683500
Si	-1.08461400	-1.65125300	-0.00006000
H	1.60590500	-3.55923700	-0.00054300

4.7.25 RSi^+, no arene

1 1

C	1.14834700	1.63581700	-0.00192700
N	-0.00001200	2.49523900	-0.00166300
C	1.67598600	-0.71878700	0.00081200

C	0.72893900	0.29540400	0.00006800
C	-0.72897600	0.29542500	0.00003800
C	-1.67604700	-0.71874200	0.00084800
C	-3.04805300	-0.41132900	-0.00042500
C	-3.42622700	0.94351400	-0.00290100
C	-2.49283700	1.97946400	-0.00387200
C	-1.14834700	1.63585300	-0.00197300
C	4.07173400	-1.54732000	0.00046500
C	-4.07181800	-1.54720800	0.00047500
C	3.04800000	-0.41141200	-0.00048200
C	3.42620800	0.94341900	-0.00298400
C	2.49284200	1.97939400	-0.00391300
C	3.86216400	-2.40407300	1.26024500
H	2.85855100	-2.85367200	1.29578400
H	4.59231000	-3.22746600	1.27945200
H	3.99931900	-1.80679200	2.17457500
C	3.85976000	-2.40861100	-1.25579000
H	4.58982900	-3.23210500	-1.27338400
H	2.85607300	-2.85831100	-1.28783300
H	3.99522200	-1.81465800	-2.17253700
C	5.51116800	-1.02903000	-0.00185200
H	5.73263200	-0.42828200	-0.89748600
H	5.73428500	-0.42486700	0.89107200
H	6.20772300	-1.87994300	-0.00087300
C	-3.86048600	-2.40775900	-1.25640900
H	-2.85682600	-2.85746700	-1.28917900
H	-4.59061000	-3.23120300	-1.27416400
H	-3.99634200	-1.81323900	-2.17272900
C	-5.51123900	-1.02887900	-0.00078900
H	-5.73383800	-0.42517000	0.89257200
H	-5.73319000	-0.42766300	-0.89598800
H	-6.20781500	-1.87977500	0.00012000
C	-3.86164700	-2.40471300	1.25963000
H	-4.59178000	-3.22811900	1.27867400
H	-2.85802800	-2.85435700	1.29444200
H	-3.99838100	-1.80799300	2.17438900
H	4.48271500	1.20935400	-0.00465500
H	1.34489800	-1.75903900	0.00226900
H	-1.34498600	-1.75900300	0.00234200
H	-4.48272900	1.20947200	-0.00449600
Si	0.00029700	4.18772700	0.00550100
H	-2.84383100	3.01648200	-0.00663400
H	2.84389000	3.01639900	-0.00669600

4.7.26 RSi(NH$_2$^{t}Bu)$^+$

1 1

Si	-0.28394800	-0.90073300	-0.01326500
N	0.01787700	0.84029400	-0.31982800
C	1.22650700	1.48599100	-0.59647200
C	2.48060500	0.90227200	-0.79440300
C	3.57229300	1.76309000	-0.95706600
H	4.55014100	1.30950900	-1.12146700
C	3.44726900	3.16332200	-0.94045400

C	2.17420600	3.71326000	-0.73631100
H	2.04225300	4.79697600	-0.70124300
C	1.06492800	2.88687700	-0.55933400
C	-0.33588200	3.12684100	-0.26575100
C	-1.11015600	4.27290300	-0.08800600
H	-0.63952400	5.25487000	-0.17426800
C	-2.47727700	4.16720900	0.19684500
C	-3.04696200	2.88126800	0.27117700
H	-4.11531100	2.76973500	0.45740100
C	-2.30260200	1.71259600	0.09215700
C	-0.93756000	1.85707500	-0.15232900
C	2.61002500	-0.56314800	-0.73835700
C	1.67707300	-1.37962100	-1.42078800
H	1.05760400	-0.90684300	-2.18855000
C	1.76966100	-2.79411900	-1.36742500
C	2.72482300	-3.34503900	-0.51782000
H	2.78160000	-4.43121100	-0.42934600
C	3.65458400	-2.56300000	0.19363500
C	3.59444700	-1.17171400	0.04979200
H	4.30673300	-0.52935100	0.56666000
C	0.86797800	-3.70334800	-2.20434100
C	1.75024000	-4.65829100	-3.02503300
H	2.35740100	-5.31750100	-2.38770500
H	2.43271100	-4.10069500	-3.68436100
H	1.11728900	-5.30134600	-3.65549300
C	-0.00554100	-2.90489500	-3.17435900
H	-0.70109400	-2.23376300	-2.64666900
H	-0.61538800	-3.59477600	-3.77547000
H	0.59838200	-2.30882600	-3.87577800
C	-0.03993200	-4.51616700	-1.26532800
H	-0.70185600	-3.85606200	-0.68255500
H	0.54429100	-5.13145000	-0.56435400
H	-0.67621400	-5.19594300	-1.85287300
C	4.69193200	-3.25086000	1.08099500
C	3.96679000	-4.11857100	2.12237300
H	4.69891900	-4.62123700	2.77244200
H	3.34762300	-4.89990900	1.65778000
H	3.31420000	-3.50621100	2.76554900
C	5.58401900	-4.13880400	0.19733100
H	6.34239300	-4.64413800	0.81471900
H	6.10742000	-3.53984600	-0.56314300
H	5.00642200	-4.91668200	-0.32350600
C	5.57866200	-2.24619100	1.81915700
H	6.29900300	-2.78401600	2.45263600
H	4.99366300	-1.58435900	2.47766200
H	6.15869200	-1.61913100	1.12547000
C	4.65010500	4.09352600	-1.13317200
C	5.95561800	3.31842200	-1.32381000
H	6.19294300	2.69097200	-0.45039300
H	6.78932500	4.02395900	-1.45569600
H	5.92627500	2.67578000	-2.21725700
C	4.79812900	4.99053300	0.10584000
H	4.96055900	4.38744300	1.01272500
H	3.90808300	5.61547300	0.27213800
H	5.65892000	5.66706400	-0.01244400
C	4.41798300	4.96487600	-2.37752500

H	5.27109700	5.64334100	-2.53453100
H	3.51421500	5.58508100	-2.28220100
H	4.30543300	4.34331100	-3.27907300
C	-3.30645900	5.44085900	0.39980600
C	-2.72835400	6.23391500	1.58240300
H	-2.76211000	5.64051500	2.50926300
H	-3.30890300	7.15535000	1.74614300
H	-1.68238800	6.52812900	1.40881200
C	-3.24215800	6.29575500	-0.87553800
H	-2.21201300	6.59557100	-1.11949900
H	-3.83384100	7.21605500	-0.74969800
H	-3.64581700	5.74583200	-1.73956700
C	-4.77520300	5.13335400	0.69948300
H	-5.25965200	4.59242900	-0.12795600
H	-5.32817000	6.07342200	0.84426900
H	-4.89170800	4.53868500	1.61872700
C	-2.89619800	0.34993000	0.05692600
C	-3.05663900	-0.41975700	1.21575100
H	-2.81269200	0.03382900	2.17662900
C	-3.55587200	-1.73343100	1.15109900
C	-3.89819100	-2.23246600	-0.10474700
H	-4.30255700	-3.24076600	-0.17564700
C	-3.76745700	-1.48714200	-1.28921500
C	-3.24830800	-0.19596700	-1.18884500
H	-3.11604300	0.42590100	-2.07475000
C	-3.68187200	-2.57784000	2.42327700
C	-4.40505400	-1.78140800	3.51876600
H	-3.86492600	-0.86434500	3.79759400
H	-4.50600900	-2.39154100	4.42950200
H	-5.41435600	-1.49004700	3.19125900
C	-4.45655700	-3.87568000	2.18101900
H	-3.94293900	-4.53972300	1.46932000
H	-5.46962500	-3.67735200	1.79971500
H	-4.55937300	-4.42911000	3.12623700
C	-2.26787600	-2.94497200	2.90452700
H	-1.71798300	-3.49377500	2.12198600
H	-2.31342400	-3.58200400	3.80176300
H	-1.69814200	-2.04140700	3.17006500
C	-4.26387300	-2.06950700	-2.61462700
C	-5.79993800	-1.98416600	-2.61279200
H	-6.20793900	-2.39788000	-3.54848600
H	-6.13800500	-0.94056600	-2.52337200
H	-6.23145500	-2.55145900	-1.77396900
C	-3.84026900	-3.53848300	-2.75835900
H	-4.15225000	-3.92376400	-3.74090000
H	-4.30366700	-4.18582800	-1.99982200
H	-2.74940400	-3.64961700	-2.67825000
C	-3.72600100	-1.28972800	-3.81756100
H	-2.62510100	-1.27153400	-3.83283300
H	-4.08753200	-0.25097600	-3.83479300
H	-4.06607100	-1.76339500	-4.75037100
N	0.79011700	-1.12064600	1.76564400
H	1.70876100	-1.48455000	1.47051500
C	1.01633900	-0.17573400	2.93184800
C	-0.27326200	0.57251000	3.24174600
H	-0.10227300	1.25462500	4.08630700

H	-0.60825700	1.17809000	2.38724700
H	-1.08043400	-0.11363100	3.53518400
C	2.12505800	0.81039600	2.59197500
H	3.06577800	0.29385400	2.35289400
H	1.85853300	1.45901800	1.74865700
H	2.31102400	1.45163100	3.46480300
H	0.29527000	-1.93797500	2.13639800
C	1.43244200	-1.03821500	4.12400300
H	1.60680800	-0.40627600	5.00665800
H	0.65064000	-1.76874600	4.38887500
H	2.36576700	-1.58526000	3.91629600

4.7.27 RSi(H)NH^{t}Bu$^+$

1 1

C	-1.45129000	1.23026900	-0.45791900
N	-0.84769200	0.00533000	-0.11505100
C	-3.64678900	2.22006000	-0.71137400
C	-2.85672900	1.09744900	-0.46449500
C	-3.13993500	-0.30526700	-0.19812400
C	-4.28219100	-1.10551100	-0.14981900
C	-4.17929000	-2.49024200	0.06024600
C	-2.90080700	-3.06233300	0.21081800
C	-1.74287100	-2.28127400	0.19377000
C	-1.88914200	-0.91397500	-0.00292000
C	0.65896300	2.45363300	-0.91075700
C	1.24746300	1.66260100	-1.91169900
C	2.63182900	1.54355000	-2.01617600
C	3.42056900	2.25384300	-1.09537400
C	2.87430700	3.07543400	-0.10698800
C	1.47519500	3.16648200	-0.03391000
C	3.73652900	3.85303700	0.89256500
C	3.30406300	0.63488700	-3.04957300
C	-3.93910900	4.69102900	-1.19118800
C	-5.45741700	-3.33694000	0.10615400
C	-0.34629500	-2.74670000	0.24602000
C	0.51299000	-2.23119700	1.29016400
C	1.87627000	-2.69603000	1.35719700
C	2.37383700	-3.39070500	0.26045100
C	1.56600100	-3.81369200	-0.80687600
C	0.18926600	-3.51319200	-0.76977100
C	2.77861800	-2.46932200	2.57059600
C	2.12368600	-4.61948000	-1.97302500
C	-3.05054500	3.46414500	-0.95565800
C	-1.64447300	3.53795400	-0.99909500
C	-0.81910400	2.43287600	-0.77583400
C	-4.83491300	4.44342200	-2.41489100
H	-5.49110000	3.57117500	-2.27631800
H	-5.48005100	5.31667600	-2.59925600
H	-4.22970900	4.26944000	-3.31783300
C	-4.81322300	4.91925900	0.05218600
H	-5.46050400	5.79868800	-0.09085700
H	-5.46565400	4.05813700	0.26079300
H	-4.19160200	5.09458200	0.94401300
C	-3.12004000	5.95891700	-1.44223900

H	-2.46863400	6.20302900	-0.58882200
H	-2.49414200	5.87330800	-2.34375700
H	-3.79707200	6.81280800	-1.59357500
C	5.23291700	3.64568200	0.65157100
H	5.53581100	3.98560100	-0.35028100
H	5.81230500	4.22598300	1.38506800
H	5.52589600	2.58990500	0.76171700
C	3.40860600	3.37850300	2.31741000
H	2.35037300	3.54533100	2.57151100
H	3.63612800	2.30553500	2.43653500
H	4.01163500	3.92671100	3.05781700
C	3.42522200	5.35239100	0.76807000
H	4.03617200	5.93148800	1.47799500
H	3.64623100	5.71487400	-0.24719500
H	2.36870800	5.57349000	0.98085600
C	-6.34459500	-2.84380300	1.26008500
H	-6.63427200	-1.78976000	1.13510100
H	-7.27040400	-3.43782500	1.31177300
H	-5.82463100	-2.93787900	2.22598600
C	-5.16015500	-4.82146000	0.32853300
H	-4.54989500	-5.24560000	-0.48398700
H	-4.64246600	-4.99964800	1.28385000
H	-6.10314900	-5.38721000	0.35741200
C	-6.21119500	-3.19021700	-1.22501000
H	-7.13204900	-3.79386100	-1.21066800
H	-6.50315500	-2.14805500	-1.42226200
H	-5.59250100	-3.53163200	-2.06920400
C	1.45456000	-6.00600900	-1.95884500
H	0.36275700	-5.94245800	-2.07562300
H	1.84317900	-6.61046100	-2.79237200
H	1.66823700	-6.54198300	-1.02161400
C	3.64064200	-4.79876700	-1.88861000
H	3.94378900	-5.36628700	-0.99563800
H	3.99087600	-5.36356100	-2.76452000
H	4.16829300	-3.83242300	-1.88850400
C	4.32154800	1.44136900	-3.86959500
H	5.11577600	1.86904200	-3.24025300
H	4.80574700	0.79679000	-4.61975400
H	3.82884900	2.27058100	-4.39948400
C	4.02578800	-0.50209100	-2.30567500
H	4.79398200	-0.11809300	-1.61731400
H	3.31403600	-1.10053500	-1.71367600
H	4.52528600	-1.17477800	-3.02156300
C	2.28814000	0.01786200	-4.01285400
H	1.54872900	-0.60670000	-3.48813600
H	1.74295700	0.78878100	-4.57813100
H	2.80570300	-0.62353500	-4.74224900
C	1.79047000	-3.89277500	-3.28771600
H	0.70815300	-3.77261300	-3.43982100
H	2.25279200	-2.89543600	-3.31853100
H	2.18067100	-4.47349600	-4.13698400
C	3.80586800	-1.37002400	2.24083200
H	4.51252300	-1.26463400	3.07802900
H	4.38375000	-1.61140700	1.33603000
H	3.32713600	-0.39264200	2.08653100
C	3.53023600	-3.77284300	2.89453400

H	4.24693900	-4.05875500	2.11185600
H	4.10402900	-3.63688700	3.82333600
H	2.83285700	-4.61039100	3.04662400
C	1.97427300	-2.06753000	3.80790500
H	1.44772900	-1.11760700	3.65406700
H	1.24438300	-2.84401300	4.08612500
H	2.65342200	-1.93322200	4.66245100
H	-1.15753700	4.48199100	-1.24460500
H	-4.73438200	2.12029900	-0.71087600
H	-5.26311000	-0.64734200	-0.29343100
H	-2.79499900	-4.13800500	0.35229100
H	-0.47491700	-3.85601200	-1.56458900
H	3.42364800	-3.67595200	0.26027200
H	0.00518700	-2.01858400	2.23991300
H	1.00011200	3.78300700	0.73179300
H	4.50434600	2.15847400	-1.16120300
H	0.58551400	1.12463100	-2.59058100
Si	0.63937000	-0.16335700	0.79904600
N	0.70840000	0.76222300	2.22246700
H	1.57078000	1.29879300	2.29047700
H	1.91233700	-0.07857000	0.06884100
C	-0.29449400	1.18730800	3.22557400
C	-0.95137300	2.50370600	2.79999100
H	-0.19524800	3.29341400	2.67486000
H	-1.49415800	2.39604600	1.85096300
H	-1.66351700	2.84274700	3.56722300
C	-1.36325100	0.10793800	3.38530000
H	-0.93537000	-0.83257700	3.76615800
H	-2.11892900	0.43627600	4.11267200
H	-1.89324300	-0.09356800	2.44157600
C	0.43129400	1.39801700	4.55630000
H	1.21828200	2.16299900	4.45834200
H	-0.27325300	1.74585000	5.32554300
H	0.89793100	0.47018900	4.91691800

4.7.28 RSi(H)(NH^{t}Bu)NH$_2$^{t}Bu$^+$

1 1

Si	-0.51070300	-0.49394500	0.97384500
N	-0.23911100	0.68202300	-0.32682000
C	0.77972100	1.67678800	-0.36343000
C	2.17925400	1.56845200	-0.27768400
C	2.90759400	2.77177400	-0.24440400
H	3.99263800	2.68422100	-0.19670800
C	2.33635700	4.04541000	-0.36157000
C	0.95762800	4.11061300	-0.58041200
H	0.46376900	5.06922800	-0.75396200
C	0.19739100	2.94565200	-0.59192200
C	-1.21255500	2.73223500	-0.83144900
C	-2.25672900	3.60925700	-1.11270500
H	-2.04992200	4.67872900	-1.19218200
C	-3.55670900	3.12054300	-1.28012800
C	-3.75175200	1.73383500	-1.17689700
H	-4.74809100	1.31534100	-1.32095200
C	-2.72302400	0.82255400	-0.90527000

C	-1.43933700	1.34823500	-0.71604900
C	2.95529700	0.30659100	-0.39456600
C	2.61756500	-0.63547900	-1.37898700
H	1.72365800	-0.45601900	-1.97616200
C	3.43844800	-1.73919800	-1.63088000
C	4.58940100	-1.89238900	-0.84689800
H	5.24146800	-2.74617300	-1.04206600
C	4.95012300	-0.99376800	0.16354300
C	4.11205100	0.10596000	0.37774500
H	4.35905400	0.84500400	1.14133200
C	3.14549400	-2.75103800	-2.74083100
C	1.86291600	-2.40390000	-3.49520600
H	1.93885100	-1.43357500	-4.00912600
H	0.99454400	-2.36808500	-2.82174900
H	1.65580400	-3.16815700	-4.25877100
C	2.99392500	-4.15054100	-2.12450000
H	3.90861300	-4.47300900	-1.60497200
H	2.78026000	-4.89254600	-2.90945100
H	2.16607800	-4.17943600	-1.39999200
C	4.31268900	-2.75235700	-3.74047700
H	5.26130700	-3.04767700	-3.26811900
H	4.45289400	-1.75496000	-4.18423200
H	4.11294500	-3.46434200	-4.55630900
C	6.24706900	-1.21442900	0.94804700
C	6.23399800	-2.60841500	1.59444300
H	7.16901000	-2.77888800	2.14992800
H	6.14505600	-3.41036900	0.84696200
H	5.39810600	-2.71510900	2.30295600
C	7.42929600	-1.11537900	-0.03023900
H	8.38060300	-1.26454100	0.50387800
H	7.45949700	-0.12684800	-0.51330000
H	7.36900700	-1.87519100	-0.82350400
C	6.44287400	-0.16848600	2.04784500
H	7.37919100	-0.36950500	2.58895300
H	5.62810200	-0.18604300	2.78896300
H	6.51470100	0.85089500	1.63907600
C	3.16849500	5.32994100	-0.31766000
C	4.65145100	5.04937800	-0.06564800
H	4.81322100	4.52455100	0.88896800
H	5.20525700	5.99871500	-0.01715300
H	5.10065000	4.45027200	-0.87269600
C	2.64354800	6.22139500	0.81877800
H	2.73016000	5.71310100	1.79178000
H	1.58724300	6.49310000	0.67338800
H	3.22235700	7.15679700	0.87032700
C	3.03361200	6.06858300	-1.65823800
H	3.62605400	6.99674800	-1.64464000
H	1.99057500	6.34507700	-1.87299800
H	3.39455500	5.44490500	-2.49036700
C	-4.70525000	4.09411600	-1.56064100
C	-4.81623700	5.08096000	-0.38753300
H	-5.01696300	4.54967400	0.55606400
H	-5.63942100	5.79166700	-0.56090100
H	-3.89473200	5.66739400	-0.25433100
C	-4.41359700	4.86295300	-2.85845900
H	-3.48050200	5.44237300	-2.79347000

H	-5.22887000	5.57087700	-3.07496100
H	-4.32337200	4.17451800	-3.71265300
C	-6.04723500	3.37568900	-1.71511900
H	-6.03975600	2.66745800	-2.55779300
H	-6.84097800	4.11174000	-1.91162900
H	-6.32704800	2.82665900	-0.80265600
C	-3.02571100	-0.62341500	-0.76325900
C	-3.84758900	-1.04054000	0.29266400
H	-4.28600700	-0.27169100	0.92914800
C	-4.06804200	-2.39485400	0.54714800
C	-3.49217800	-3.31784600	-0.33619100
H	-3.65380500	-4.38140200	-0.15302100
C	-2.74152000	-2.93865900	-1.45477000
C	-2.49459400	-1.57246600	-1.64173300
H	-1.88586300	-1.22209000	-2.47604600
C	-4.87503800	-2.88815200	1.75135500
C	-5.49245600	-1.73327200	2.54336500
H	-4.72752700	-1.06228400	2.96701800
H	-6.07604300	-2.12950000	3.38751000
H	-6.17211800	-1.12825200	1.92437300
C	-6.01027400	-3.80652100	1.27477600
H	-5.63097000	-4.69073300	0.74207400
H	-6.68985800	-3.27052500	0.59501100
H	-6.59856200	-4.16501500	2.13368700
C	-3.93381200	-3.66862100	2.68341600
H	-3.47755400	-4.53131900	2.17520400
H	-4.48267800	-4.04720300	3.55989400
H	-3.11767100	-3.02383900	3.05009600
C	-2.17354400	-4.00793200	-2.39340600
C	-3.20390300	-5.11852700	-2.64358900
H	-2.80046900	-5.84898000	-3.36132100
H	-4.13509200	-4.70816900	-3.06288900
H	-3.45845700	-5.67448700	-1.72969100
C	-0.92943100	-4.61042900	-1.72176700
H	-0.46156000	-5.37035300	-2.36778100
H	-1.18767700	-5.08956800	-0.76440800
H	-0.17887900	-3.83171000	-1.51706900
C	-1.78818300	-3.41725400	-3.75280800
H	-0.98839300	-2.66828900	-3.67800500
H	-2.65370600	-2.94373700	-4.24077800
H	-1.42108100	-4.21468200	-4.41633700
H	-0.83497100	-1.85500400	0.52284500
N	-1.54432100	-0.02903900	2.24370300
H	-2.37036500	-0.62165000	2.28626800
N	1.17107200	-0.67855100	1.79469300
H	1.23241600	0.09990700	2.46043700
H	1.93539000	-0.50519400	1.11491100
C	1.51555400	-1.97032300	2.53001800
C	-1.70601600	1.19747000	3.04531500
C	-2.03287000	0.78020900	4.48123800
H	-2.18710200	1.66198600	5.12064300
H	-2.95588400	0.17949600	4.51419800
H	-1.22032800	0.17778600	4.91510900
C	-2.84963600	2.05181700	2.48952000
H	-3.00326900	2.94803300	3.10934000
H	-2.64698900	2.37356300	1.45881800

H	-3.79279500	1.48361200	2.49019800
C	-0.41019200	2.00656000	3.03157500
H	-0.54969500	2.95223400	3.57362900
H	0.40631700	1.47460300	3.55136700
H	-0.09174200	2.26950000	2.01006300
C	0.35093900	-2.41859200	3.40382300
H	0.04068400	-1.63464900	4.10839700
H	-0.52635300	-2.71507000	2.81192200
H	0.67034100	-3.29525300	3.98503100
C	1.85566300	-3.01957200	1.47877700
H	2.13800400	-3.95613800	1.97944300
H	0.99830300	-3.23716100	0.82523300
H	2.70259200	-2.70169700	0.85240100
C	2.73339400	-1.65256000	3.39237500
H	3.56560900	-1.27859500	2.77881300
H	2.49444400	-0.90223100	4.16348500
H	3.07416500	-2.56095300	3.90847500

4.7.29 model $R^{M}Si(NH_3)^+$

1 1

C	1.21996600	1.58805100	-0.23097400
N	0.04138500	0.84161300	-0.11726500
C	1.99333500	3.88620800	-0.24721000
C	0.94277900	2.97173600	-0.19674300
C	-0.49749100	3.09375900	-0.07365600
C	-1.37940800	4.17093900	0.00691000
C	-2.75596800	3.95231000	0.14299900
C	-3.21713600	2.62360400	0.21440600
C	-2.35961200	1.52305800	0.14503300
C	-1.00015300	1.77829100	-0.01813800
C	2.76633800	-0.33937900	-0.38705000
C	2.00439800	-1.12950800	-1.28771500
C	2.18386300	-2.53045900	-1.35263800
C	3.04807400	-3.11796500	-0.42249500
C	3.80922600	-2.36553300	0.48561800
C	3.66519400	-0.96754200	0.47322200
C	4.76601200	-3.01406300	1.48703800
C	1.48026900	-3.40050100	-2.39557200
C	4.45804100	4.47279200	-0.37138300
C	-3.70742300	5.15239300	0.21722500
C	-2.81223100	0.11293000	0.24675400
C	-2.65800800	-0.58456000	1.45945800
C	-3.05465200	-1.92617900	1.57883700
C	-3.54799600	-2.56018900	0.43228600
C	-3.70736000	-1.90121800	-0.79410700
C	-3.34389400	-0.55134400	-0.86311500
C	-3.03872500	-2.61796400	2.94725600
C	-4.26077100	-2.60373100	-2.03620600
C	3.32219900	3.44468800	-0.32041700
C	3.56177200	2.06054800	-0.35549000
C	2.53043800	1.11324400	-0.32904500
C	4.28711000	5.34966200	-1.62181600
H	3.33101700	5.89407200	-1.61480600
H	5.09386900	6.09712800	-1.67838700

H	4.32060900	4.74088000	-2.53840600
C	4.40075900	5.35183000	0.88780300
H	5.20934000	6.09915600	0.86916900
H	3.44887400	5.89769300	0.96735400
H	4.51814800	4.74518700	1.79911600
C	5.83562600	3.80939900	-0.43221500
H	6.02847100	3.18037400	0.45091100
H	5.95532300	3.19000500	-1.33454800
H	6.61792900	4.58234800	-0.46054300
C	4.83416400	-4.53386500	1.32650500
H	5.19207800	-4.82514800	0.32739300
H	5.53812000	-4.95110700	2.06123000
H	3.85874900	-5.01407200	1.50049700
C	4.27698800	-2.69420100	2.91006200
H	4.24509200	-1.61128500	3.10392000
H	3.27067200	-3.10947200	3.08703600
H	4.95244000	-3.14260500	3.65464000
C	6.17523800	-2.43535600	1.28151500
H	6.87813700	-2.89666300	1.99177100
H	6.53959600	-2.63725700	0.26296100
H	6.20784800	-1.34807000	1.44395800
C	-3.33216700	6.02197500	1.42738900
H	-2.30356900	6.40604700	1.35588500
H	-4.00545500	6.89063200	1.49792500
H	-3.41374200	5.45012700	2.36466500
C	-5.16856500	4.72351600	0.36786800
H	-5.50888000	4.11580200	-0.48476900
H	-5.33607900	4.14991900	1.29260100
H	-5.81296500	5.61408300	0.41353800
C	-3.57690500	5.97841700	-1.07210100
H	-4.25287900	6.84730100	-1.04036100
H	-2.55461500	6.35923100	-1.21541700
H	-3.83766700	5.37509300	-1.95526600
C	-5.50282200	-1.84707100	-2.53248500
H	-5.27219600	-0.80823400	-2.81089500
H	-5.91774000	-2.34320500	-3.42340300
H	-6.28633000	-1.82401700	-1.75972800
C	-4.65695300	-4.05444000	-1.75403400
H	-5.44369500	-4.12482900	-0.98729100
H	-5.05183700	-4.51464400	-2.67180000
H	-3.79762500	-4.66080200	-1.42777700
C	2.54244800	-4.19254600	-3.17612200
H	3.11533500	-4.87251000	-2.52893500
H	2.05545900	-4.80541700	-3.94992200
H	3.25422600	-3.51745600	-3.67509300
C	0.53190100	-4.37651000	-1.67773300
H	1.07180500	-5.03344700	-0.97918400
H	-0.24524400	-3.83546300	-1.11485400
H	0.02814400	-5.02019400	-2.41531700
C	0.67444500	-2.56582000	-3.39354700
H	-0.12714600	-1.99218300	-2.90368200
H	1.31465200	-1.86966900	-3.95691500
H	0.19174100	-3.23076900	-4.12462200
C	-3.17956500	-2.60126800	-3.12800700
H	-2.86730800	-1.58095400	-3.39719700
H	-2.28694500	-3.15202800	-2.79468500

H	-3.55825900	-3.08583600	-4.04139800
C	-3.43459000	-4.09357500	2.85739100
H	-3.39055500	-4.55213900	3.85644400
H	-4.46279000	-4.21947500	2.48794200
H	-2.75940300	-4.66218100	2.19899400
C	-4.04366800	-1.89373400	3.85916500
H	-5.05458800	-1.92496100	3.42620200
H	-4.08119800	-2.37496900	4.84905400
H	-3.77533700	-0.83687400	4.00704800
C	-1.64213300	-2.54349600	3.57955500
H	-0.91128300	-3.10842200	2.97496800
H	-1.30359000	-1.50237600	3.71233800
H	-1.64589400	-2.99942200	4.58118200
H	4.58330200	1.68823600	-0.43948200
H	1.77019500	4.95511800	-0.21973000
H	-0.98523200	5.18867100	-0.03794200
H	-4.28164700	2.42277600	0.33728700
H	-3.45566300	0.00851200	-1.79411500
H	-3.84612400	-3.60344300	0.50148400
H	-2.29631200	-0.02728000	2.32876800
H	4.23819600	-0.34653200	1.16546900
H	3.16334200	-4.20084800	-0.43478600
H	1.47832300	-0.61203600	-2.09571800
Si	-0.17940600	-0.94511800	-0.15395500
N	0.77781600	-1.20440300	1.62636600
H	1.14117800	-0.32355000	2.00165100
H	0.12454200	-1.60394900	2.30784800
H	1.57598400	-1.84541000	1.53171200

4.7.30 model $R^MSi(NH_3)^+$ TS

1 1

C	1.08388300	1.36633700	-0.12136200
N	-0.01669400	0.50419200	-0.08843400
C	1.63620600	3.72047600	-0.03782800
C	0.66935000	2.71019700	-0.06854400
C	-0.77791600	2.68379700	-0.02384600
C	-1.75521700	3.68102000	0.04119700
C	-3.09258200	3.30535500	0.08558700
C	-3.46049500	1.95269900	0.06539600
C	-2.50649800	0.93586200	-0.00089700
C	-1.16234500	1.32395800	-0.04275100
C	2.76279500	-0.43086000	-0.20783000
C	2.08973700	-1.27209200	-1.14348600
C	2.40064500	-2.65996600	-1.20922800
C	3.25586100	-3.22338800	-0.28349300
C	3.86372500	-2.40013500	0.68117500
C	3.63467500	-1.02720900	0.71172200
C	-2.84871800	-0.50770200	-0.03414300
C	-2.97116000	-1.23848900	1.15740700
C	-3.15379800	-2.62551500	1.12141600
C	-3.24215400	-3.28990400	-0.10477900
C	-3.15149500	-2.56604100	-1.29134100
C	2.98391400	3.36787400	-0.04650200
C	3.38173400	2.02591000	-0.09312200

C	2.43444900	0.99834500	-0.15001800
H	4.44480200	1.77479800	-0.12037700
H	1.34020800	4.77095100	0.00219000
H	-1.47153900	4.73549100	0.05812200
H	-4.51745500	1.67815200	0.09739400
H	-3.39665200	-4.37098200	-0.13073700
H	-2.91238800	-0.71506100	2.11533000
H	4.12739200	-0.40227900	1.45994400
H	3.47644000	-4.29236500	-0.31055900
H	1.61371400	-0.79963500	-2.01380900
Si	0.12622200	-1.26258700	-0.07671000
N	0.46926800	-1.66150500	1.65739700
H	0.64685000	-1.01448800	2.43369700
H	-0.82800200	-2.06972000	0.97607000
H	0.77960900	-2.60788300	1.87929900
C	-2.94745300	-1.18528000	-1.25693400
H	-2.86404400	-0.61818700	-2.18760300
H	3.74698400	4.14817500	-0.02222900
H	-3.87056900	4.06971500	0.13545500
H	1.93490200	-3.27365600	-1.98440100
H	4.54289900	-2.84490600	1.41365100
H	-3.23737100	-3.07804300	-2.25260300
H	-3.25376100	-3.18416300	2.05558900

4.7.31 model $R^MSi(H)NH_2^+$

1 1

C	0.44295500	1.91583100	-0.02086600
N	-0.38446900	0.78472800	0.16705200
C	0.25693700	4.34032300	-0.12077500
C	-0.34549300	3.08976500	-0.00081400
C	-1.72920800	2.66806100	0.12688400
C	-2.96725900	3.31200800	0.12462800
C	-4.15884700	2.57409500	0.18854500
C	-4.08060700	1.16990200	0.26092700
C	-2.85313900	0.50403400	0.28838700
C	-1.69515200	1.27004700	0.21983900
C	2.62773700	0.75394500	-0.42361000
C	2.23962100	-0.18528200	-1.39641100
C	2.91413100	-1.40119400	-1.53421500
C	3.98683300	-1.65543600	-0.66213700
C	4.42385000	-0.72865300	0.28980400
C	3.72872900	0.48726300	0.38600400
C	5.59533400	-1.00575500	1.23539000
C	2.54026500	-2.44177200	-2.59474600
C	2.29305100	5.82963700	-0.38112300
C	-5.50218300	3.31367500	0.17318700
C	-2.64839100	-0.95260600	0.35271800
C	-1.82798400	-1.45872400	1.42594900
C	-1.65463200	-2.87112700	1.57592300
C	-2.04069500	-3.67779900	0.50487300
C	-2.72368100	-3.18188500	-0.61528200
C	-3.06282000	-1.81293200	-0.64781600
C	-1.12466600	-3.51363300	2.85458000

C	-3.11210400	-4.07429300	-1.78801600
C	1.64464400	4.44571100	-0.26884300
C	2.39119700	3.25739700	-0.33950600
C	1.82165200	1.98174000	-0.24473500
C	1.72722300	6.55458500	-1.61227800
H	0.63773300	6.69279300	-1.54441600
H	2.18205800	7.55269000	-1.71059800
H	1.93850000	5.99079200	-2.53390100
C	1.97312300	6.63721300	0.88658800
H	2.42912600	7.63784300	0.82734900
H	0.89034000	6.77485500	1.02546500
H	2.36653900	6.13571000	1.78426800
C	3.81388900	5.74481500	-0.52704300
H	4.28191100	5.24591800	0.33575700
H	4.11118700	5.20944900	-1.44188800
H	4.23676300	6.75853400	-0.58941900
C	6.24529100	-2.36510100	0.97064900
H	6.64808000	-2.43467800	-0.05130300
H	7.08435900	-2.51654300	1.66571900
H	5.54033600	-3.19721400	1.12313700
C	5.07872000	-0.98792500	2.68354200
H	4.64190600	-0.01444500	2.95340500
H	4.31105400	-1.76329100	2.84054300
H	5.90215300	-1.18621000	3.38707300
C	6.65930700	0.08761200	1.05262000
H	7.51088800	-0.09361100	1.72654300
H	7.03845500	0.09882400	0.01944300
H	6.26582400	1.08979900	1.27820100
C	-5.57162300	4.25578500	1.38531900
H	-4.76697000	5.00597400	1.37057500
H	-6.52981800	4.79836400	1.39310800
H	-5.49029700	3.69343900	2.32827200
C	-6.69142400	2.35313000	0.24105300
H	-6.71569600	1.66620100	-0.61925300
H	-6.68628900	1.75540600	1.16577200
H	-7.63058100	2.92575900	0.22774300
C	-5.61457100	4.13025700	-1.12386000
H	-6.57474300	4.66850400	-1.15392200
H	-4.81356700	4.87932600	-1.21110200
H	-5.56180100	3.47681300	-2.00831900
C	-4.63528000	-4.00421800	-1.98983500
H	-4.98256700	-2.98660300	-2.22021400
H	-4.92527900	-4.64845400	-2.83336900
H	-5.17180900	-4.35493100	-1.09517900
C	-2.70966000	-5.53384900	-1.57040000
H	-3.20913700	-5.97503200	-0.69428500
H	-3.00438000	-6.12883000	-2.44675900
H	-1.62127500	-5.64835400	-1.45009200
C	3.75487100	-2.68730500	-3.50422700
H	4.61744300	-3.07636700	-2.94364400
H	3.50534400	-3.42359200	-4.28413700
H	4.06816000	-1.75654400	-4.00087300
C	2.14594900	-3.75759100	-1.90319900
H	2.96502800	-4.16248700	-1.29048800
H	1.27114500	-3.62097200	-1.24693000
H	1.88726400	-4.52067600	-2.65416400

C	1.37336700	-1.97879200	-3.47009000
H	0.45886400	-1.79702300	-2.88469200
H	1.61544900	-1.05678000	-4.01975700
H	1.13765900	-2.75330900	-4.21538400
C	-2.39140900	-3.54845300	-3.04302400
H	-2.66937600	-2.51077100	-3.27955800
H	-1.29835600	-3.58896000	-2.91953300
H	-2.65782100	-4.17092500	-3.91058700
C	0.14176900	-4.33073500	2.54967900
H	0.48301000	-4.83511000	3.46613600
H	-0.03766300	-5.10845900	1.79274800
H	0.95369500	-3.67950700	2.19847800
C	-2.22967100	-4.45463200	3.37593800
H	-2.44887800	-5.27204900	2.67387000
H	-1.90263800	-4.90826000	4.32383400
H	-3.16500800	-3.90692700	3.56730700
C	-0.81641200	-2.48727500	3.94645500
H	-0.02855000	-1.78945000	3.63370600
H	-1.71264600	-1.91905400	4.24075500
H	-0.45811800	-3.01009700	4.84503100
H	3.46647200	3.30347000	-0.51488200
H	-0.36779800	5.23600200	-0.10114800
H	-3.00239000	4.40144900	0.05668700
H	-4.99069200	0.57194300	0.31193400
H	-3.63273200	-1.40157400	-1.48242900
H	-1.84733500	-4.74737900	0.56677800
H	-1.73380100	-0.82066500	2.31299400
H	4.01775700	1.23112500	1.13240500
H	4.51303500	-2.60560700	-0.75154100
H	1.41920100	0.07661000	-2.06701600
Si	-0.00826300	-0.89666400	0.47144800
N	1.24060000	-1.14850000	1.59227800
H	2.06458000	-1.64097100	1.24854200
H	-0.03024700	-1.85634800	-0.64457300
H	1.52849800	-0.36902900	2.17642700

4.7.32 $[RSi(H)NH_2]^+$+F^-

0 1

Si	0.14174000	-0.61239300	1.42098500
N	-0.02022500	0.73599400	0.25013400
C	1.05420400	1.63307700	0.08441900
C	2.42788900	1.36956800	0.04926500
C	3.28804400	2.46801900	-0.05972800
H	4.35587400	2.24844300	-0.08727600
C	2.84198800	3.79649500	-0.17192900
C	1.46252100	4.02018100	-0.19901800
H	1.06378800	5.03059300	-0.31950000
C	0.57842400	2.95060600	-0.07461100
C	-0.86367800	2.86233500	-0.09920900
C	-1.84804400	3.83202400	-0.26802900
H	-1.54941300	4.87780800	-0.37531800
C	-3.19538400	3.46430700	-0.31240300
C	-3.49530400	2.09710400	-0.22946600
H	-4.52923000	1.77010900	-0.34161700

C	-2.53297600	1.08629000	-0.07367700
C	-1.19320000	1.49274100	0.05056700
C	2.98190500	-0.00956000	0.03664700
C	2.85963900	-0.79043100	-1.11734300
H	2.33501100	-0.36293300	-1.97273800
C	3.42093300	-2.07036400	-1.18388900
C	4.06181400	-2.55628900	-0.03834800
H	4.49512200	-3.55790000	-0.06608900
C	4.17861000	-1.81285800	1.14257400
C	3.65058500	-0.52028300	1.15207200
H	3.70841400	0.10508700	2.04128300
C	3.42750300	-2.89249400	-2.47762500
C	2.37310300	-2.40059400	-3.47377200
H	2.57102100	-1.37281300	-3.81198400
H	1.36192600	-2.43026800	-3.04143800
H	2.37541000	-3.04211300	-4.36848400
C	3.16160000	-4.37619200	-2.18725500
H	3.96197900	-4.83348600	-1.58762300
H	3.10238500	-4.94192100	-3.13039000
H	2.21498100	-4.51150200	-1.64450500
C	4.81749300	-2.74778000	-3.11930100
H	5.60596200	-3.10855800	-2.44094400
H	5.03377400	-1.69403400	-3.35341400
H	4.87720300	-3.32823300	-4.05464700
C	4.84457000	-2.43695500	2.37237300
C	4.05799100	-3.69398200	2.77661200
H	4.51034400	-4.16230500	3.66572800
H	4.04422800	-4.44509500	1.97257900
H	3.01386500	-3.43944900	3.01617000
C	6.29353700	-2.81881600	2.03591900
H	6.78510900	-3.27379600	2.91095800
H	6.87422900	-1.93146000	1.73982100
H	6.34552600	-3.54427700	1.21007500
C	4.85606400	-1.47678200	3.56389300
H	5.32397300	-1.96568500	4.43229200
H	3.83676500	-1.17908500	3.85371600
H	5.43157300	-0.56370200	3.34717800
C	3.80747700	4.98120200	-0.29248100
C	5.27246900	4.54213200	-0.24004500
H	5.51194400	4.03113200	0.70513000
H	5.92819600	5.42316500	-0.31711200
H	5.52754500	3.86609400	-1.07051100
C	3.55617100	5.95632200	0.86816000
H	3.72136100	5.46186300	1.83785400
H	2.52574800	6.34210800	0.86163200
H	4.23735500	6.82026100	0.80198500
C	3.56786200	5.70090300	-1.62867600
H	4.25196900	6.55860600	-1.73418000
H	2.53911200	6.08292400	-1.70856800
H	3.73816800	5.01954500	-2.47658300
C	-4.27570800	4.53708600	-0.48748800
C	-4.18826800	5.53370800	0.67868600
H	-4.34933200	5.02542000	1.64198700
H	-4.95280400	6.32047400	0.57325000
H	-3.20563000	6.02671200	0.72191800
C	-4.04841700	5.27789100	-1.81421200

H	-3.06355100	5.76741000	-1.84499000
H	-4.81391800	6.05763700	-1.95805600
H	-4.10251700	4.58201300	-2.66563400
C	-5.68557000	3.94222200	-0.50455800
H	-5.82705300	3.24621700	-1.34564800
H	-6.42828300	4.74745900	-0.61383800
H	-5.91325700	3.40487300	0.42912400
C	-2.97610900	-0.32537000	-0.12835300
C	-4.10688700	-0.73348000	0.59615200
H	-4.61318800	0.00694000	1.21589000
C	-4.55077100	-2.05406500	0.56554900
C	-3.84267100	-2.96596500	-0.23437900
H	-4.18450500	-4.00025100	-0.26763800
C	-2.72619700	-2.59499900	-0.98699500
C	-2.29922600	-1.26108100	-0.91349100
H	-1.42328500	-0.93021900	-1.47618100
C	-5.75751200	-2.53980200	1.37410800
C	-6.40981600	-1.40934600	2.17263600
H	-5.71458300	-0.97004500	2.90451500
H	-7.27431600	-1.79898400	2.73157800
H	-6.77414900	-0.60324000	1.51740700
C	-6.80830100	-3.12340200	0.41696100
H	-6.41296500	-3.97395900	-0.15792700
H	-7.14949900	-2.36247800	-0.30181600
H	-7.68508000	-3.48037300	0.98071100
C	-5.29674300	-3.62520200	2.35962800
H	-4.85787000	-4.49025400	1.84042500
H	-6.14758300	-3.99007000	2.95713500
H	-4.53763100	-3.22962000	3.05270200
C	-1.95684300	-3.59651800	-1.85346500
C	-2.68087000	-4.94029400	-1.96640800
H	-2.11299800	-5.61288900	-2.62727500
H	-3.68969400	-4.82519300	-2.39223800
H	-2.77279100	-5.44313900	-0.99159600
C	-0.58109800	-3.83908900	-1.21702500
H	-0.00808200	-4.56711000	-1.81243500
H	-0.68214800	-4.23774000	-0.19546900
H	0.01073200	-2.91397300	-1.15572000
C	-1.77504000	-3.03050700	-3.26958500
H	-1.21067100	-2.08681400	-3.26939700
H	-2.74887700	-2.83928200	-3.74615800
H	-1.21961000	-3.74545200	-3.89720200
H	0.74504500	-1.80587600	0.80413400
N	-1.27940300	-1.03815500	2.25345600
H	-1.89851400	-1.72378800	1.83238500
H	-1.82354400	-0.31662800	2.71490900
F	1.14859900	0.01016200	2.53866600

4.7.33 $[RSi(H)NH_2]^{+}$+H^{-}

0 1

Si	0.14174000	-0.61239300	1.42098500
N	-0.02022500	0.73599400	0.25013400
C	1.05420400	1.63307700	0.08441900
C	2.42788900	1.36956800	0.04926500

C	3.28804400	2.46801900	-0.05972800
H	4.35587400	2.24844300	-0.08727600
C	2.84198800	3.79649500	-0.17192900
C	1.46252100	4.02018100	-0.19901800
H	1.06378800	5.03059300	-0.31950000
C	0.57842400	2.95060600	-0.07461100
C	-0.86367800	2.86233500	-0.09920900
C	-1.84804400	3.83202400	-0.26802900
H	-1.54941300	4.87780800	-0.37531800
C	-3.19538400	3.46430700	-0.31240300
C	-3.49530400	2.09710400	-0.22946600
H	-4.52923000	1.77010900	-0.34161700
C	-2.53297600	1.08629000	-0.07367700
C	-1.19320000	1.49274100	0.05056700
C	2.98190500	-0.00956000	0.03664700
C	2.85963900	-0.79043100	-1.11734300
H	2.33501100	-0.36293300	-1.97273800
C	3.42093300	-2.07036400	-1.18388900
C	4.06181400	-2.55628900	-0.03834800
H	4.49512200	-3.55790000	-0.06608900
C	4.17861000	-1.81285800	1.14257400
C	3.65058500	-0.52028300	1.15207200
H	3.70841400	0.10508700	2.04128300
C	3.42750300	-2.89249400	-2.47762500
C	2.37310300	-2.40059400	-3.47377200
H	2.57102100	-1.37281300	-3.81198400
H	1.36192600	-2.43026800	-3.04143800
H	2.37541000	-3.04211300	-4.36848400
C	3.16160000	-4.37619200	-2.18725500
H	3.96197900	-4.83348600	-1.58762300
H	3.10238500	-4.94192100	-3.13039000
H	2.21498100	-4.51150200	-1.64450500
C	4.81749300	-2.74778000	-3.11930100
H	5.60596200	-3.10855800	-2.44094400
H	5.03377400	-1.69403400	-3.35341400
H	4.87720300	-3.32823300	-4.05464700
C	4.84457000	-2.43695500	2.37237300
C	4.05799100	-3.69398200	2.77661200
H	4.51034400	-4.16230500	3.66572800
H	4.04422800	-4.44509500	1.97257900
H	3.01386500	-3.43944900	3.01617000
C	6.29353700	-2.81881600	2.03591900
H	6.78510900	-3.27379600	2.91095800
H	6.87422900	-1.93146000	1.73982100
H	6.34552600	-3.54427700	1.21007500
C	4.85606400	-1.47678200	3.56389300
H	5.32397300	-1.96568500	4.43229200
H	3.83676500	-1.17908500	3.85371600
H	5.43157300	-0.56370200	3.34717800
C	3.80747700	4.98120200	-0.29248100
C	5.27246900	4.54213200	-0.24004500
H	5.51194400	4.03113200	0.70513000
H	5.92819600	5.42316500	-0.31711200
H	5.52754500	3.86609400	-1.07051100
C	3.55617100	5.95632200	0.86816000
H	3.72136100	5.46186300	1.83785400

H	2.52574800	6.34210800	0.86163200
H	4.23735500	6.82026100	0.80198500
C	3.56786200	5.70090300	-1.62867600
H	4.25196900	6.55860600	-1.73418000
H	2.53911200	6.08292400	-1.70856800
H	3.73816800	5.01954500	-2.47658300
C	-4.27570800	4.53708600	-0.48748800
C	-4.18826800	5.53370800	0.67868600
H	-4.34933200	5.02542000	1.64198700
H	-4.95280400	6.32047400	0.57325000
H	-3.20563000	6.02671200	0.72191800
C	-4.04841700	5.27789100	-1.81421200
H	-3.06355100	5.76741000	-1.84499000
H	-4.81391800	6.05763700	-1.95805600
H	-4.10251700	4.58201300	-2.66563400
C	-5.68557000	3.94222200	-0.50455800
H	-5.82705300	3.24621700	-1.34564800
H	-6.42828300	4.74745900	-0.61383800
H	-5.91325700	3.40487300	0.42912400
C	-2.97610900	-0.32537000	-0.12835300
C	-4.10688700	-0.73348000	0.59615200
H	-4.61318800	0.00694000	1.21589000
C	-4.55077100	-2.05406500	0.56554900
C	-3.84267100	-2.96596500	-0.23437900
H	-4.18450500	-4.00025100	-0.26763800
C	-2.72619700	-2.59499900	-0.98699500
C	-2.29922600	-1.26108100	-0.91349100
H	-1.42328500	-0.93021900	-1.47618100
C	-5.75751200	-2.53980200	1.37410800
C	-6.40981600	-1.40934600	2.17263600
H	-5.71458300	-0.97004500	2.90451500
H	-7.27431600	-1.79898400	2.73157800
H	-6.77414900	-0.60324000	1.51740700
C	-6.80830100	-3.12340200	0.41696100
H	-6.41296500	-3.97395900	-0.15792700
H	-7.14949900	-2.36247800	-0.30181600
H	-7.68508000	-3.48037300	0.98071100
C	-5.29674300	-3.62520200	2.35962800
H	-4.85787000	-4.49025400	1.84042500
H	-6.14758300	-3.99007000	2.95713500
H	-4.53763100	-3.22962000	3.05270200
C	-1.95684300	-3.59651800	-1.85346500
C	-2.68087000	-4.94029400	-1.96640800
H	-2.11299800	-5.61288900	-2.62727500
H	-3.68969400	-4.82519300	-2.39223800
H	-2.77279100	-5.44313900	-0.99159600
C	-0.58109800	-3.83908900	-1.21702500
H	-0.00808200	-4.56711000	-1.81243500
H	-0.68214800	-4.23774000	-0.19546900
H	0.01073200	-2.91397300	-1.15572000
C	-1.77504000	-3.03050700	-3.26958500
H	-1.21067100	-2.08681400	-3.26939700
H	-2.74887700	-2.83928200	-3.74615800
H	-1.21961000	-3.74545200	-3.89720200
H	0.74504500	-1.80587600	0.80413400
N	-1.27940300	-1.03815500	2.25345600

H	-1.89851400	-1.72378800	1.83238500
H	-1.82354400	-0.31662800	2.71490900
F	1.14859900	0.01016200	2.53866600

4.7.34 RSiI+F⁻

-1 1

C	-1.36860300	1.37232800	0.36689700
N	-0.21563500	0.62350700	0.12879300
C	-2.05498800	3.68589600	0.79685900
C	-1.05666800	2.73853600	0.58565000
C	0.38273100	2.82312700	0.55763400
C	1.26018300	3.89241200	0.72033200
C	2.63921000	3.67713000	0.68579900
C	3.08531500	2.35167000	0.53957800
C	2.23390600	1.25259200	0.38797500
C	0.85085300	1.50104100	0.33590200
C	-3.11855400	-0.47598500	0.38171600
C	-2.40675700	-1.45823600	1.08411200
C	-2.79214300	-2.79958400	1.05250500
C	-3.92299000	-3.14474400	0.29969500
C	-4.66032800	-2.19256800	-0.41155800
C	-4.24423300	-0.86041600	-0.35134600
C	-5.86829600	-2.56263900	-1.27891000
C	-1.94285800	-3.85154700	1.77415300
C	-4.49161800	4.35044600	1.03413100
C	3.60152000	4.86198000	0.82932200
C	2.83032500	-0.10977300	0.41834200
C	3.45142100	-0.66385400	-0.69822200
C	3.99655700	-1.94957900	-0.65583300
C	3.96560900	-2.64164300	0.56003900
C	3.38854300	-2.09646500	1.71340500
C	2.80415200	-0.83095900	1.61405500
C	4.51136900	-2.58458600	-1.95004800
C	3.41551400	-2.81682500	3.06700000
C	-3.39820300	3.29834000	0.81357500
C	-3.68523200	1.93314100	0.64949700
C	-2.71006600	0.94702900	0.44113400
C	-4.28361900	5.03367700	2.39456900
H	-3.30201200	5.52705600	2.45426900
H	-5.05747700	5.80012100	2.57070000
H	-4.33488000	4.29675200	3.21116700
C	-4.41339700	5.40260000	-0.08299600
H	-5.18696000	6.17746900	0.05325700
H	-3.43399800	5.90347700	-0.09984000
H	-4.56150700	4.93474500	-1.06851300
C	-5.89606100	3.74221800	1.01619800
H	-6.11073600	3.24580300	0.05730000
H	-6.03126000	3.00276300	1.82050400
H	-6.64906600	4.53368800	1.16014700
C	-6.20001100	-4.05509400	-1.20917100
H	-6.44018900	-4.37288700	-0.18245700
H	-7.07624900	-4.27269200	-1.84047600
H	-5.36629900	-4.67469900	-1.57340300
C	-5.55645100	-2.20548300	-2.74069900

H	-5.36425600	-1.12963700	-2.86347200
H	-4.65810800	-2.73846800	-3.08769300
H	-6.40066600	-2.47721100	-3.39656000
C	-7.10019800	-1.77544300	-0.80689500
H	-7.97773800	-2.01829000	-1.42922500
H	-7.34220000	-2.01846100	0.23974900
H	-6.93637900	-0.68935800	-0.86766100
C	3.35466700	5.85381000	-0.31837500
H	2.32218300	6.23400400	-0.30886900
H	4.03506600	6.71910500	-0.24130800
H	3.51829200	5.36819700	-1.29260800
C	5.06807500	4.42805500	0.77646000
H	5.31685400	3.73398200	1.59391900
H	5.31025200	3.93253900	-0.17600600
H	5.72328800	5.30876400	0.87406300
C	3.35892500	5.56546300	2.17337500
H	4.03393500	6.43049600	2.28941700
H	2.32498300	5.93198000	2.25797700
H	3.53628800	4.87400900	3.01190800
C	4.34315700	-2.04183400	4.01624400
H	3.99331900	-1.00808000	4.15772900
H	4.38584900	-2.52727200	5.00626400
H	5.36572400	-1.99434900	3.60986200
C	3.93176500	-4.25282900	2.94926900
H	4.97032000	-4.28740300	2.58606700
H	3.90934100	-4.74084700	3.93690000
H	3.30923700	-4.84868800	2.26384100
C	-2.64219200	-5.21218400	1.84107400
H	-2.80350500	-5.64167900	0.84062000
H	-2.01654200	-5.92319200	2.40366200
H	-3.61813800	-5.14524500	2.34817000
C	-0.62318700	-4.01710600	1.00337000
H	-0.81543600	-4.35693200	-0.02573500
H	-0.06156100	-3.07589600	0.91879300
H	0.02093400	-4.76530100	1.49600300
C	-1.65711300	-3.39954800	3.21345300
H	-1.09469200	-2.45616300	3.24526500
H	-2.59455000	-3.25345900	3.77342600
H	-1.05452200	-4.15697600	3.74064100
C	2.00339900	-2.86953600	3.66418800
H	1.58908500	-1.86509800	3.83278500
H	1.31702400	-3.39932000	2.98983100
H	2.01151200	-3.39338800	4.63468800
C	5.22200100	-3.91711100	-1.70128500
H	5.59818900	-4.32269600	-2.65401500
H	6.08118200	-3.80123100	-1.02087700
H	4.54243300	-4.66874900	-1.27106200
C	5.49805100	-1.63942300	-2.65045900
H	6.36550400	-1.42460300	-2.00567500
H	5.86718200	-2.09540100	-3.58416400
H	5.02588500	-0.68284200	-2.91606800
C	3.30049100	-2.83248000	-2.86529200
H	2.56753300	-3.48704900	-2.36993800
H	2.77659900	-1.89496200	-3.10528300
H	3.62013700	-3.30624500	-3.80940400
H	-4.71691900	1.58819900	0.72833500

H	-1.77181800	4.73057300	0.95421300
H	0.85331200	4.89601900	0.87548700
H	4.15336200	2.13172900	0.56915400
H	2.32299200	-0.36973800	2.48013100
H	4.39769900	-3.63918900	0.60758000
H	3.42632000	-0.09603600	-1.62962600
H	-4.77045800	-0.09420500	-0.92525700
H	-4.22736300	-4.18822600	0.25641700
H	-1.51617000	-1.15209700	1.63612600
Si	-0.41236900	-0.60400500	-1.35708400
I	1.03295100	0.82607200	-3.17029000
F	0.75691300	-1.69573800	-0.87074100

4.7.35 RSiI+H⁻

-1 1

C	1.38337100	1.34649700	-0.39972500
N	0.23412400	0.57796400	-0.21346900
C	2.04079100	3.68413200	-0.76678700
C	1.05687900	2.70951000	-0.61569000
C	-0.38155800	2.77385200	-0.62983900
C	-1.25781800	3.84287200	-0.79747800
C	-2.63644700	3.62843300	-0.77099200
C	-3.07841500	2.30449700	-0.61003400
C	-2.22795100	1.20299500	-0.45663300
C	-0.84069800	1.44401900	-0.42399900
C	3.18816800	-0.45046000	-0.37426000
C	2.61419900	-1.39958300	-1.23232000
C	3.01484500	-2.73222000	-1.20842300
C	4.01709200	-3.10399600	-0.29748700
C	4.62972500	-2.18192400	0.55221400
C	4.20694700	-0.84897700	0.48821700
C	5.70471100	-2.57983200	1.56897500
C	2.31932100	-3.79254800	-2.06771100
C	4.46978100	4.41193300	-0.86253300
C	-3.60004200	4.80986600	-0.93065100
C	-2.87077700	-0.13722700	-0.41973900
C	-3.61942400	-0.52986600	0.69071000
C	-4.27232000	-1.76387300	0.72483400
C	-4.19485800	-2.58083100	-0.41001000
C	-3.47942300	-2.20461600	-1.55315200
C	-2.81026100	-0.97701900	-1.53353700
C	-4.97211400	-2.21271300	2.01123700
C	-3.43738500	-3.07178000	-2.81686300
C	3.39153800	3.33123300	-0.72128600
C	3.70011400	1.96950700	-0.56331200
C	2.73944200	0.95958500	-0.42081500
C	4.31584700	5.12212600	-2.21630400
H	3.32610300	5.59225800	-2.31641100
H	5.07754500	5.91176800	-2.33390500
H	4.42882200	4.40662200	-3.04564900
C	4.31045200	5.43679100	0.27137000
H	5.07384500	6.22954800	0.19280800
H	3.32102300	5.91717800	0.24601200
H	4.41593600	4.94980600	1.25305000

C	5.88490900	3.83385800	-0.78553300
H	6.06135600	3.32018300	0.17200400
H	6.07697200	3.11549300	-1.59731700
H	6.62635800	4.64425700	-0.87345000
C	6.07005400	-4.06300400	1.47856700
H	6.45821900	-4.32757600	0.48242800
H	6.85124100	-4.30160000	2.21783600
H	5.20396300	-4.70819000	1.69146300
C	5.17864700	-2.29736300	2.98505200
H	4.93338500	-1.23423900	3.12246700
H	4.25903700	-2.86946300	3.18008900
H	5.93195000	-2.57499500	3.74162300
C	6.97687800	-1.75623100	1.31790200
H	7.75986000	-2.02001400	2.04844100
H	7.37121700	-1.94363100	0.30666100
H	6.78579800	-0.67664000	1.40661400
C	-3.36047100	5.81623100	0.20571500
H	-2.32839900	6.19748700	0.19709100
H	-4.04123000	6.67983300	0.11419100
H	-3.52926700	5.34270900	1.18505000
C	-5.06604800	4.37385500	-0.87986500
H	-5.30864100	3.66824400	-1.68925500
H	-5.31277200	3.89040300	0.07767500
H	-5.72256800	5.25178100	-0.99270500
C	-3.35197500	5.49757700	-2.28195000
H	-4.02680700	6.36086600	-2.41159100
H	-2.31757800	5.86296900	-2.36592600
H	-3.52497600	4.79593700	-3.11288500
C	-4.16496300	-2.33172200	-3.95040000
H	-3.69676400	-1.35764500	-4.15645400
H	-4.14371600	-2.92382800	-4.88115600
H	-5.21706000	-2.14584700	-3.68300700
C	-4.11344300	-4.43020800	-2.61543100
H	-5.18227200	-4.32663700	-2.37241900
H	-4.04009600	-5.02423300	-3.54040400
H	-3.63279600	-5.00474000	-1.80846300
C	3.31903900	-4.83555100	-2.58486300
H	3.79244700	-5.40304900	-1.77006400
H	2.80380700	-5.56453900	-3.23109500
H	4.11861100	-4.35926600	-3.17417100
C	1.26397900	-4.48464700	-1.18938700
H	1.73511000	-4.97630900	-0.32428300
H	0.54203400	-3.75263200	-0.79547300
H	0.71262400	-5.24839200	-1.76407700
C	1.62760600	-3.16583200	-3.28170300
H	0.82690400	-2.47521000	-2.98570600
H	2.34468200	-2.60953000	-3.90561100
H	1.16592200	-3.95072600	-3.90162900
C	-1.98022800	-3.32110500	-3.22507000
H	-1.45532500	-2.38250500	-3.45151500
H	-1.42524200	-3.82203900	-2.41823100
H	-1.92966200	-3.95492300	-4.12620700
C	-5.84032600	-3.45529200	1.79615000
H	-6.35031000	-3.72119400	2.73585800
H	-6.61220900	-3.28488200	1.02833700
H	-5.24158300	-4.32760900	1.49244500

C	-5.87204000	-1.09052600	2.54791400
H	-6.63930300	-0.81132100	1.80817000
H	-6.38285900	-1.41894900	3.46804800
H	-5.29538700	-0.18848900	2.79647400
C	-3.88701000	-2.53831200	3.05080800
H	-3.24148900	-3.35578000	2.69372800
H	-3.23487800	-1.67140600	3.24058800
H	-4.34439500	-2.84871300	4.00590100
H	4.74127500	1.64467300	-0.58912900
H	1.73810500	4.72438700	-0.91812300
H	-0.84843600	4.84676100	-0.94463800
H	-4.14677000	2.08417100	-0.62503900
H	-2.22683700	-0.64428200	-2.39549000
H	-4.70644100	-3.54128000	-0.40207200
H	-3.61972100	0.13028300	1.55956900
H	4.63120800	-0.10216000	1.16341700
H	4.31545100	-4.15030800	-0.24936500
H	1.81952600	-1.06366900	-1.89833200
Si	0.31650700	-0.81704800	1.10766400
I	-0.89153900	0.51671100	3.09374000
H	-0.99300800	-1.46460700	0.66666600

4.7.36 $RSi^{+}+F^{-}$

0 1

C	1.30267000	1.52514200	-0.16777600
N	0.10044900	0.83287700	-0.05644800
C	2.17378800	3.79794000	-0.19609500
C	1.08853800	2.92312200	-0.13727200
C	-0.34149100	3.10366300	-0.02184600
C	-1.17372900	4.22124800	0.04167900
C	-2.55919400	4.06932800	0.15936200
C	-3.07914200	2.76210300	0.23033200
C	-2.27518300	1.62205300	0.18010600
C	-0.89555200	1.80230600	0.03235900
C	2.77556100	-0.45897200	-0.39981500
C	2.09000500	-1.16294100	-1.41074100
C	2.17280900	-2.56191800	-1.50347300
C	2.90520300	-3.23472500	-0.51936300
C	3.57229700	-2.56709200	0.51321300
C	3.51542000	-1.16787600	0.54073600
C	4.29107100	-3.30936500	1.64117600
C	1.47027400	-3.35465300	-2.60932500
C	4.65851000	4.28403900	-0.33136600
C	-3.45533000	5.31276100	0.21625400
C	-2.81751300	0.24467600	0.28641000
C	-2.66583800	-0.47242700	1.48313700
C	-3.10092600	-1.80096800	1.59373900
C	-3.65118600	-2.39960400	0.45488400
C	-3.80837200	-1.71769000	-0.75873500
C	-3.40184000	-0.38138900	-0.81766000
C	-2.97400800	-2.53584600	2.93174200
C	-4.37027400	-2.39277700	-2.01336400
C	3.47983600	3.30376500	-0.28326500
C	3.65921100	1.90818800	-0.33616600

C	2.59547200	1.00368700	-0.29995500
C	4.51739700	5.19109100	-1.56354000
H	3.58279000	5.77111900	-1.53845800
H	5.35402800	5.90681800	-1.61591600
H	4.51605400	4.59576400	-2.48971300
C	4.65567700	5.14425100	0.94189800
H	5.49438500	5.85930900	0.92816000
H	3.72530200	5.72287700	1.04200400
H	4.75525200	4.51497300	1.83983400
C	6.00682000	3.56529500	-0.41850200
H	6.17836600	2.91154400	0.45055800
H	6.08691200	2.95374100	-1.33036200
H	6.82223100	4.30464400	-0.44331500
C	4.29866000	-4.82440200	1.42811700
H	4.80492100	-5.10407500	0.49108000
H	4.83690400	-5.31240300	2.25491900
H	3.27977100	-5.24052100	1.40880100
C	3.55273100	-3.00527700	2.95596800
H	3.57459100	-1.93215200	3.19567200
H	2.49480300	-3.30126100	2.88811200
H	4.01838500	-3.55057000	3.79272200
C	5.74581000	-2.82643200	1.73562600
H	6.26647800	-3.34275300	2.55777600
H	6.29121400	-3.03347200	0.80173900
H	5.80980800	-1.74581800	1.93013600
C	-3.06247800	6.16934100	1.42994000
H	-2.01419000	6.49892600	1.37522300
H	-3.69378800	7.07098000	1.48851800
H	-3.18562200	5.60228000	2.36559400
C	-4.93747400	4.95372300	0.34450700
H	-5.28905600	4.35623000	-0.51071400
H	-5.14366300	4.38890600	1.26666600
H	-5.54206200	5.87327500	0.37732100
C	-3.27002100	6.13166800	-1.07067400
H	-3.90295300	7.03387000	-1.05060000
H	-2.22732300	6.45798700	-1.20036100
H	-3.54630500	5.53793100	-1.95577200
C	-5.58758300	-1.60377400	-2.51826200
H	-5.32852700	-0.56515500	-2.77126900
H	-5.99942100	-2.07420300	-3.42542000
H	-6.38182100	-1.57592100	-1.75621100
C	-4.80577700	-3.83598400	-1.75098700
H	-5.59427600	-3.89359700	-0.98472500
H	-5.20907100	-4.27577000	-2.67595000
H	-3.96271500	-4.46555200	-1.42731900
C	2.51945700	-4.15160300	-3.39890000
H	3.06408800	-4.86042000	-2.75737700
H	2.03400300	-4.73055000	-4.20085500
H	3.25895400	-3.47940800	-3.86109500
C	0.45620900	-4.31902500	-1.97160600
H	0.94285600	-5.04108700	-1.29902300
H	-0.29372300	-3.76195400	-1.38843300
H	-0.06804900	-4.89110300	-2.75395200
C	0.71856800	-2.44144300	-3.58064200
H	-0.06260100	-1.85987800	-3.06742200
H	1.39613100	-1.74151900	-4.09355300

H	0.22341000	-3.04925700	-4.35334700
C	-3.27687300	-2.40816500	-3.09382100
H	-2.94623200	-1.39188500	-3.35507100
H	-2.39341000	-2.96564900	-2.74744300
H	-3.65042400	-2.88797900	-4.01288100
C	-3.51227400	-3.96634800	2.85697400
H	-3.40710100	-4.45252500	3.83883000
H	-4.57954700	-3.99216200	2.58752100
H	-2.95584900	-4.57419400	2.12691600
C	-3.78065500	-1.77042000	3.99296200
H	-4.84311200	-1.70579500	3.71089300
H	-3.71428700	-2.28259300	4.96625000
H	-3.40592600	-0.74523300	4.12956100
C	-1.49839300	-2.59737000	3.35210700
H	-0.89752600	-3.14735500	2.61300200
H	-1.04886300	-1.59911700	3.44632700
H	-1.40405200	-3.10607800	4.32496400
H	4.66148200	1.48935300	-0.43448600
H	1.99073500	4.87510700	-0.16543100
H	-0.72825900	5.21839900	-0.00316500
H	-4.15280100	2.60637700	0.34116100
H	-3.49439700	0.18982600	-1.74404400
H	-3.97062100	-3.43725400	0.51495900
H	-2.21482800	0.03731600	2.33825900
H	4.00024400	-0.60787200	1.34285400
H	2.94470400	-4.32304900	-0.55775800
H	1.57830800	-0.58432500	-2.18233000
Si	-0.16837200	-0.99910900	-0.00819600
F	0.59933500	-1.29046000	1.43409500

4.7.37 RSi^{+}+H^{-}

0 1

C	1.01483300	1.69887100	-0.12887800
N	-0.05490700	0.83368300	-0.02005000
C	1.53840300	4.07046800	-0.16917900
C	0.58923500	3.04931800	-0.11840400
C	-0.85315900	3.00081000	-0.01851000
C	-1.86186700	3.96402600	0.02083800
C	-3.20409200	3.58397000	0.12900400
C	-3.50158100	2.20955000	0.22560800
C	-2.51933000	1.21830000	0.20712900
C	-1.18903600	1.62487700	0.05144100
C	2.75309200	-0.05571000	-0.34112500
C	2.12697300	-0.86423800	-1.32350300
C	2.47778100	-2.22532900	-1.46400800
C	3.39970700	-2.75636900	-0.56176800
C	4.03765800	-1.97898800	0.41852800
C	3.71185800	-0.62366900	0.50016000
C	5.02987100	-2.57706600	1.41887300
C	1.86824700	-3.12050400	-2.54775700
C	3.92791900	4.91255300	-0.27189600
C	-4.29547600	4.66170700	0.15496700
C	-2.81573800	-0.22626000	0.36611000
C	-2.41064100	-0.89118500	1.53608900

C	-2.61906600	-2.26946600	1.69613000
C	-3.19132700	-2.96706500	0.62698700
C	-3.59609600	-2.33740600	-0.55738300
C	-3.42464400	-0.95458200	-0.65815400
C	-2.25096600	-2.95094400	3.01843600
C	-4.15870800	-3.11766400	-1.74890100
C	2.90534400	3.76968000	-0.22868600
C	3.29157000	2.41552900	-0.26174400
C	2.36992000	1.36541600	-0.23383800
C	3.67708600	5.77560100	-1.51817500
H	2.66687700	6.21146000	-1.51589300
H	4.39984600	6.60646100	-1.56694400
H	3.77977300	5.17644400	-2.43615000
C	3.77748800	5.77745900	0.98934300
H	4.50206300	6.60806600	0.97865700
H	2.77067500	6.21404700	1.06771300
H	3.95315700	5.17971900	1.89714100
C	5.36875900	4.39962200	-0.32830400
H	5.61936800	3.78752100	0.55188900
H	5.55416600	3.79706500	-1.23081200
H	6.06680800	5.25076300	-0.34974800
C	5.32190900	-4.05274400	1.13858900
H	5.75190300	-4.20174000	0.13600800
H	6.04969100	-4.43220700	1.87189000
H	4.41707300	-4.67390300	1.22266300
C	4.42717700	-2.45913600	2.82869900
H	4.24346400	-1.41076600	3.10649100
H	3.46598400	-2.99277600	2.89023300
H	5.11118100	-2.89261600	3.57597600
C	6.35438800	-1.80168300	1.35435000
H	7.07486800	-2.21744000	2.07658300
H	6.80019000	-1.86714200	0.34977200
H	6.22140500	-0.73674600	1.59462900
C	-4.06669500	5.59204200	1.35631900
H	-3.08699500	6.09024400	1.30604600
H	-4.83967800	6.37711900	1.39201900
H	-4.10602700	5.02788000	2.30098300
C	-5.69884100	4.06311600	0.27528000
H	-5.93428200	3.39990500	-0.57134000
H	-5.82055400	3.48845800	1.20632800
H	-6.44852300	4.86946000	0.28349700
C	-5.51820800	-2.53197000	-2.15753500
H	-5.44156300	-1.47299400	-2.44442800
H	-5.92709800	-3.08067900	-3.02098600
H	-6.24240100	-2.60495800	-1.33134100
C	-4.34980100	-4.60285700	-1.43374800
H	-5.04587700	-4.75714100	-0.59465100
H	-4.76814700	-5.11782900	-2.31208900
H	-3.39684200	-5.09552100	-1.18703000
C	2.99714200	-3.74132600	-3.38453400
H	3.66957100	-4.36416100	-2.77609100
H	2.57324500	-4.38197400	-4.17417300
H	3.60541200	-2.96081400	-3.86745500
C	1.05140600	-4.23333800	-1.86905100
H	1.67814600	-4.86017200	-1.21689800
H	0.24202700	-3.80152300	-1.25902100

H	0.59756500	-4.88846300	-2.62998700
C	0.94122000	-2.34332800	-3.48612000
H	0.09541500	-1.89886000	-2.93936800
H	1.47750100	-1.54433000	-4.02148000
H	0.52594800	-3.02632200	-4.24294300
C	-3.16687300	-2.99448700	-2.91776400
H	-3.02328100	-1.94652600	-3.22020700
H	-2.18044400	-3.39456400	-2.63694500
H	-3.53268000	-3.55354100	-3.79431200
C	-2.52547000	-4.45615800	2.98799500
H	-2.24673400	-4.90285300	3.95462400
H	-3.59014400	-4.67765000	2.81686400
H	-1.93718700	-4.96232500	2.20702100
C	-3.09584600	-2.32664400	4.14083300
H	-4.17106500	-2.45978600	3.94461200
H	-2.86148500	-2.80176500	5.10709200
H	-2.90451500	-1.24804100	4.24226400
C	-0.76062900	-2.74228700	3.32257000
H	-0.13076200	-3.18600000	2.53647300
H	-0.49624500	-1.67732200	3.39199900
H	-0.49939500	-3.21635100	4.28233100
H	4.34650800	2.15101100	-0.34507100
H	1.20333200	5.11082300	-0.15254300
H	-1.59012400	5.02130400	-0.03642600
H	-4.53523500	1.88139300	0.34143000
H	-3.71097300	-0.42161300	-1.56733400
H	-3.33150600	-4.04123100	0.72035800
H	-1.96476300	-0.29901600	2.33972400
H	4.16630000	0.01357300	1.26153500
H	3.64592200	-3.81489300	-0.63857300
H	1.53721200	-0.36720900	-2.09703100
Si	0.02007500	-1.02429200	-0.05634500
H	0.81099200	-1.15162400	1.24876600
C	-4.23371100	5.47894600	-1.14489100
H	-5.00859400	6.26295100	-1.14759500
H	-3.25852400	5.97263100	-1.27067100
H	-4.39575700	4.83266500	-2.02143800

4.7.38 Cp^*Si^+

1 1

C	0.23713000	1.19902600	-0.25215000
C	-1.06707400	0.59608900	-0.25209100
C	-0.89658500	-0.83052100	-0.25213000
C	0.51296300	-1.10938300	-0.25236900
C	2.69370600	0.32181200	-0.29192200
H	3.22150100	-0.51209500	0.18859700
H	3.02449900	0.36253400	-1.34229500
H	3.01060000	1.25538700	0.19065600
C	1.13822500	-2.46238000	-0.29205000
H	1.27872400	-2.76461600	-1.34243000
H	2.12419900	-2.47525300	0.19020100
H	0.50835000	-3.22190400	0.18883200
C	-1.99000600	-1.84334000	-0.29192100
H	-2.23460400	-2.06954000	-1.34236800

H	-1.69720500	-2.78539900	0.18936800
H	-2.90674400	-1.47956500	0.18989100
C	-2.36843000	1.32271500	-0.29191200
H	-2.65758600	1.48848400	-1.34230700
H	-3.17436300	0.75126600	0.18642100
H	-2.30683800	2.30552400	0.19295100
C	0.52621600	2.66126100	-0.29197200
H	1.48131900	2.90603100	0.19043400
H	0.59189100	2.98779500	-1.34241800
H	-0.26484900	3.25135100	0.18856200
Si	0.00016000	-0.00012300	1.51004700
C	1.21366600	0.14500800	-0.25227600

4.7.39 Cp*Si^{+}+F^{-}

0 1

C	-0.76845200	-1.16178700	0.29128100
C	-1.57859800	-0.02350300	0.10053700
C	-0.78617900	1.12938000	0.30575100
C	0.54088400	0.71812800	0.65130000
C	1.69459300	-1.57459100	1.10576800
H	2.65665900	-1.18408700	0.74460400
H	1.72992700	-1.60375300	2.20760100
H	1.60345900	-2.61041100	0.74916500
C	1.66269200	1.58180100	1.13513400
H	1.71812300	1.56972600	2.23633600
H	2.62845100	1.23417400	0.74137500
H	1.53350700	2.62691100	0.82000700
C	-1.26666600	2.53536600	0.17383700
H	-1.83151800	2.83874600	1.07183500
H	-0.43660900	3.24465800	0.05214600
H	-1.94115900	2.65242800	-0.68751600
C	-3.00326800	-0.00344100	-0.34943100
H	-3.66317700	0.42241300	0.42455700
H	-3.13422700	0.60826100	-1.25676900
H	-3.37442400	-1.01234200	-0.57510500
C	-1.19220500	-2.58605200	0.15083600
H	-0.34871300	-3.23502600	-0.12313900
H	-1.60326200	-2.96782300	1.10081300
H	-1.96987400	-2.70840200	-0.61639600
Si	0.70549500	0.01586800	-1.37025600
C	0.55706100	-0.72556700	0.63112400
F	2.37741700	0.06266400	-1.43141700

4.7.40 Cp*Si^{+}+H^{-}

0 1

C	1.13824400	-0.68712800	-0.26946500
C	1.13655700	0.68981900	-0.26940700
C	-0.25121400	1.14371800	-0.19965100
C	-1.09314100	-0.00138700	-0.39749000
C	-0.66980000	-2.57392100	-0.34885800
H	-1.71502000	-2.72679400	-0.04531300
H	-0.57654700	-2.90043100	-1.39885400
H	-0.04410400	-3.24133000	0.26099500

C	-2.52386200	-0.00305700	-0.82307300
H	-2.58495700	-0.00308000	-1.92526500
H	-3.05780900	-0.89008000	-0.45572000
H	-3.05990100	0.88266000	-0.45563400
C	-0.67614600	2.57215200	-0.34929000
H	-0.58316300	2.89856900	-1.39932900
H	-1.72188400	2.72249200	-0.04628500
H	-0.05243100	3.24129400	0.26069100
C	2.29974400	1.61296200	-0.15130200
H	2.25501800	2.42166000	-0.89877300
H	2.32454900	2.09146100	0.84319800
H	3.25341100	1.08483600	-0.28654000
C	2.30366400	-1.60746200	-0.15156100
H	2.32946900	-2.08628700	0.84275400
H	2.26097200	-2.41599700	-0.89933000
H	3.25606900	-1.07697100	-0.28642100
Si	-0.61561500	-0.00054600	1.64305100
C	-0.24838900	-1.14442300	-0.19968600
H	-2.15900300	-0.00200600	1.84581700

4.7.41 [RSi(H)NH^{t}Bu]$^+$+F$^-$

0 1

Si	0.02349200	-0.61624400	1.18778500
N	0.07567000	0.61405600	-0.11174900
C	1.16150400	1.50180400	-0.26547100
C	2.54115400	1.25862200	-0.20351500
C	3.38638900	2.37280100	-0.30491600
H	4.45710000	2.17144800	-0.25793200
C	2.93531200	3.68587600	-0.51573400
C	1.56007900	3.88124600	-0.66317400
H	1.15513000	4.87409100	-0.87431800
C	0.68744200	2.80227800	-0.54413500
C	-0.74849100	2.70149100	-0.66663100
C	-1.72462000	3.65699000	-0.93446000
H	-1.42324700	4.69579900	-1.08998000
C	-3.06886500	3.28280900	-1.00158000
C	-3.37261800	1.92466500	-0.82808800
H	-4.40460800	1.59196300	-0.93887500
C	-2.41967100	0.92776200	-0.56197500
C	-1.08304600	1.34538100	-0.44383700
C	3.15792800	-0.08907800	-0.14388700
C	2.88082000	-1.03193000	-1.13969200
H	2.15551400	-0.76885900	-1.91063700
C	3.55993500	-2.25534500	-1.17893200
C	4.47754100	-2.52448900	-0.15790800
H	5.00742600	-3.47980300	-0.16950500
C	4.75433800	-1.61846900	0.87301100
C	4.09314000	-0.39030500	0.85083100
H	4.26897900	0.35545700	1.62560500
C	3.38545800	-3.26420900	-2.31952500
C	2.27795100	-2.84923100	-3.29017100
H	2.50481700	-1.89242800	-3.78411000
H	1.30695900	-2.75111700	-2.78452200
H	2.16504000	-3.60944900	-4.07857000

C	3.04536500	-4.64801600	-1.74609800
H	3.85809000	-5.04314700	-1.11894700
H	2.87662300	-5.37071400	-2.56071500
H	2.13684000	-4.60743900	-1.12850800
C	4.70460000	-3.34963200	-3.10433800
H	5.53712300	-3.67635300	-2.46319500
H	4.97309300	-2.36919200	-3.52704900
H	4.61387200	-4.06925100	-3.93430400
C	5.74266900	-2.00557300	1.97725800
C	5.18896200	-3.22670200	2.72821300
H	5.87525500	-3.52910700	3.53594300
H	5.05628000	-4.09022500	2.05908800
H	4.20949200	-2.99681700	3.17537100
C	7.10278700	-2.35387700	1.35426700
H	7.82361700	-2.63820500	2.13776800
H	7.51514200	-1.49283500	0.80573700
H	7.02985400	-3.19565200	0.64960700
C	5.95096100	-0.87383900	2.98582800
H	6.67007400	-1.18985400	3.75724000
H	5.01349300	-0.60380600	3.49537500
H	6.35398700	0.03117800	2.50568300
C	3.89154200	4.87885200	-0.62346100
C	5.35421900	4.46779200	-0.44104000
H	5.52904200	4.00565200	0.54275200
H	6.00209300	5.35521400	-0.51151700
H	5.67987100	3.75792000	-1.21677100
C	3.54020900	5.90322800	0.46672300
H	3.64126100	5.45858200	1.46886400
H	2.50693600	6.26787300	0.36625000
H	4.21063700	6.77610300	0.40829500
C	3.74299100	5.53019100	-2.00692700
H	4.42064000	6.39422200	-2.10204000
H	2.71789700	5.88959900	-2.18192800
H	3.98682300	4.81276000	-2.80565800
C	-4.14367000	4.33910000	-1.27848400
C	-4.09046000	5.40853200	-0.17639300
H	-4.27609200	4.96109100	0.81248400
H	-4.85368800	6.18408400	-0.35163700
H	-3.11071300	5.90768300	-0.13747500
C	-3.87739900	4.99218500	-2.64327600
H	-2.89138200	5.47919500	-2.67732400
H	-4.63784300	5.75987100	-2.86010300
H	-3.90737000	4.24189400	-3.44841700
C	-5.55258400	3.74187900	-1.29728400
H	-5.66915300	2.99017800	-2.09301900
H	-6.29129300	4.53677800	-1.48299600
H	-5.80844600	3.26885100	-0.33643200
C	-2.88511000	-0.47643900	-0.46737400
C	-4.05722800	-0.76730300	0.25207300
H	-4.54997700	0.04900800	0.77800100
C	-4.57033100	-2.05882600	0.31940500
C	-3.89017900	-3.07086300	-0.37780900
H	-4.29003400	-4.08398700	-0.34129200
C	-2.73329800	-2.82459200	-1.11660500
C	-2.23005000	-1.51298900	-1.13514100
H	-1.32663600	-1.27704200	-1.70205400

C	-5.82575000	-2.40752200	1.12412200
C	-6.47666000	-1.16940000	1.74516000
H	-5.80511600	-0.66190100	2.45423200
H	-7.38077100	-1.46238500	2.30046700
H	-6.77879400	-0.43973100	0.97816600
C	-6.85619400	-3.07958800	0.20384500
H	-6.47059400	-4.01019600	-0.23790600
H	-7.14082200	-2.40914600	-0.62173600
H	-7.76711700	-3.33293000	0.76939400
C	-5.43771700	-3.37258800	2.25538200
H	-4.99357400	-4.30059400	1.86516500
H	-6.32291100	-3.64765000	2.85128400
H	-4.70257100	-2.90838100	2.93177700
C	-2.01241200	-3.93593800	-1.88618600
C	-2.83241000	-5.22732500	-1.94391300
H	-2.29484300	-5.98061500	-2.53993400
H	-3.81483200	-5.06559900	-2.41404600
H	-2.99454000	-5.65891700	-0.94452600
C	-0.68410600	-4.23827300	-1.18087900
H	-0.13018700	-5.02248100	-1.72061800
H	-0.85500600	-4.58499100	-0.14986600
H	-0.04229700	-3.34737200	-1.12677400
C	-1.74120300	-3.48211200	-3.32833500
H	-1.11017100	-2.58294400	-3.37197900
H	-2.68246300	-3.25633700	-3.85296700
H	-1.21888900	-4.27598700	-3.88551200
H	0.06535600	-1.98925500	0.65716200
N	-1.31054200	-0.41629300	2.22118500
H	-2.04439600	-1.11432300	2.15131500
F	1.38090300	-0.33663800	2.04202200
C	-1.56349300	0.60421700	3.24637900
C	-0.66115600	1.81959000	3.02057500
H	-0.84709900	2.57067500	3.80232800
H	0.40298000	1.54596700	3.06446400
H	-0.86349800	2.29205000	2.04744000
C	-3.02506700	1.04672400	3.15815500
H	-3.70307000	0.18845200	3.29585700
H	-3.25694900	1.78558200	3.94036100
H	-3.23143900	1.49923600	2.17660700
C	-1.28048200	0.00228900	4.62729400
H	-1.47770900	0.72793800	5.43248000
H	-1.91510700	-0.88156900	4.80104900
H	-0.22913900	-0.31606700	4.69075300

4.7.42 [RSi(H)NH^{t}Bu]$^+$+H$^-$

0 1

Si	0.04263600	-0.72523500	0.95058300
N	0.15354700	0.61007000	-0.28066300
C	1.31313500	1.39321200	-0.43676300
C	2.67080200	1.02345100	-0.42925800
C	3.61468600	2.05387400	-0.54986200
H	4.66206300	1.75105000	-0.55813100
C	3.28671800	3.40986300	-0.70352300
C	1.93154200	3.74267400	-0.76046700

H	1.61493200	4.77861200	-0.90577800
C	0.96246800	2.75000200	-0.63414900
C	-0.47962800	2.80362000	-0.65798900
C	-1.37090400	3.86641600	-0.78403900
H	-0.97755400	4.87902500	-0.90436300
C	-2.74831400	3.63561800	-0.74399500
C	-3.17938100	2.30559500	-0.61618000
H	-4.24625900	2.08022500	-0.62474300
C	-2.31413900	1.20872600	-0.50805400
C	-0.93468900	1.47451100	-0.48800900
C	3.14441600	-0.37614000	-0.33699900
C	2.57702700	-1.37413500	-1.13703500
H	1.79359500	-1.08631100	-1.83833800
C	2.99022300	-2.70446200	-1.02975300
C	4.00017100	-3.00476400	-0.10838100
H	4.32412000	-4.04323200	-0.00739400
C	4.60738500	-2.03068900	0.69245900
C	4.16312600	-0.71306600	0.56080200
H	4.57663300	0.07802100	1.18658700
C	2.35453300	-3.83118400	-1.84864800
C	1.33552400	-3.29404000	-2.85278100
H	1.80059100	-2.61297000	-3.58208500
H	0.52309200	-2.75079900	-2.34856000
H	0.88033000	-4.12575700	-3.41164600
C	1.63207100	-4.78615200	-0.88448600
H	2.32826600	-5.23880000	-0.16244900
H	1.14376000	-5.60307200	-1.44013100
H	0.85967900	-4.25029600	-0.31101300
C	3.43861000	-4.59642500	-2.62069200
H	4.18199900	-5.05025500	-1.94848600
H	3.97443100	-3.92707800	-3.31122600
H	2.98627400	-5.40896700	-3.21166000
C	5.69959500	-2.43841000	1.68671900
C	5.11542900	-3.44288500	2.69204700
H	5.88312700	-3.74978700	3.42060600
H	4.74451600	-4.35097700	2.19380900
H	4.27419300	-2.99765400	3.24551400
C	6.86273900	-3.08972100	0.92260100
H	7.65882600	-3.39558200	1.62051000
H	7.29616100	-2.38665200	0.19456900
H	6.53966500	-3.98518100	0.37103200
C	6.24580500	-1.24026900	2.46556800
H	7.03018400	-1.57439600	3.16208600
H	5.46149100	-0.74816300	3.06091800
H	6.69288800	-0.48800900	1.79763000
C	4.35003600	4.50627600	-0.82998700
C	5.77183800	3.94797300	-0.73572100
H	5.94795100	3.43917100	0.22465400
H	6.50060400	4.76932200	-0.81519500
H	5.98677400	3.23519700	-1.54661000
C	4.15816800	5.52667800	0.30281800
H	4.25968800	5.04345200	1.28689400
H	3.16496700	5.99845300	0.26339700
H	4.91199200	6.32768300	0.23339100
C	4.19778500	5.21151800	-2.18646500
H	4.95067800	6.00918900	-2.29502600

H	3.20486500	5.67239900	-2.29775500
H	4.32999400	4.49899200	-3.01524100
C	-3.72414100	4.81204200	-0.85329500
C	-3.46675700	5.78721900	0.30622300
H	-3.61071900	5.28749900	1.27682900
H	-4.15889800	6.64334400	0.25346200
H	-2.44121400	6.18524900	0.28514100
C	-3.50496700	5.53460300	-2.19113200
H	-2.47791900	5.91794300	-2.28436400
H	-4.19185100	6.39137600	-2.28546400
H	-3.68583300	4.85431300	-3.03765700
C	-5.18471100	4.36102500	-0.78464700
H	-5.44141200	3.67912400	-1.60979500
H	-5.84892200	5.23587700	-0.85932200
H	-5.41102500	3.85189000	0.16493700
C	-2.90680600	-0.15008700	-0.44362500
C	-3.77255900	-0.47846800	0.60654400
H	-3.97366200	0.28664600	1.35678500
C	-4.33472400	-1.75235300	0.71354200
C	-4.06151100	-2.66694600	-0.31004600
H	-4.49823000	-3.66608800	-0.24787900
C	-3.26040500	-2.35396800	-1.41442100
C	-2.65934600	-1.09094600	-1.44801900
H	-2.00115000	-0.80395700	-2.26849300
C	-5.18114400	-2.17904100	1.91658900
C	-5.45765600	-1.01363200	2.86953800
H	-4.53060300	-0.60354500	3.29973700
H	-6.08121000	-1.35871700	3.70828500
H	-5.99448900	-0.19346600	2.36842300
C	-6.52862200	-2.73990200	1.43954200
H	-6.40372900	-3.61781800	0.78867600
H	-7.09187000	-1.98082000	0.87504100
H	-7.14098200	-3.05144700	2.30070200
C	-4.40983200	-3.26256300	2.68787800
H	-4.21286200	-4.14439100	2.05977600
H	-4.98334300	-3.59484600	3.56831700
H	-3.43792900	-2.87797100	3.03637600
C	-3.07098000	-3.39225500	-2.52529600
C	-4.44361600	-3.88559800	-3.00911700
H	-4.31954100	-4.62044500	-3.82053500
H	-5.04815400	-3.04982200	-3.39415500
H	-5.01729700	-4.37348000	-2.20751500
C	-2.26799100	-4.57690700	-1.96692700
H	-2.11006900	-5.34087200	-2.74566900
H	-2.78943700	-5.05733000	-1.12497600
H	-1.28240100	-4.25034800	-1.60500300
C	-2.33212500	-2.81166900	-3.73295600
H	-1.31707300	-2.47714500	-3.47916600
H	-2.87606400	-1.95768700	-4.16502700
H	-2.23620900	-3.57961000	-4.51595600
H	-0.50323400	-1.94655800	0.30641600
N	-1.00484500	-0.33676500	2.25624000
H	-1.95965800	-0.66687400	2.15311200
H	1.42514400	-0.90749200	1.43609600
C	-0.86580100	0.68254100	3.29882000
C	-1.80389900	1.86456000	3.02143200

H	-1.72050000	2.62886500	3.81017400
H	-1.57461800	2.33363700	2.05314900
H	-2.85381400	1.53112300	2.99281000
C	-1.22925700	0.04576900	4.64409600
H	-0.55947200	-0.79994500	4.86030100
H	-1.15838400	0.77405900	5.46730300
H	-2.26245100	-0.33865200	4.62272000
C	0.57677200	1.18616100	3.34506300
H	0.68508500	1.94647700	4.13245000
H	1.27973800	0.36777100	3.56194400
H	0.87373600	1.65393900	2.39165500

4.7.43 R_2Si

0 1

Si	0.65346700	-0.05100500	0.01723500
N	-0.50489800	-0.90374800	-1.09055900
N	-0.40265500	0.93465300	1.11744300
C	-0.25041800	-2.29093900	-1.26516100
C	0.95897100	-2.99639200	-1.36811700
C	0.86905500	-4.39625200	-1.44500400
H	1.81398800	-4.93181400	-1.53798500
C	-0.33622600	-5.10951900	-1.49388300
C	-1.51956800	-4.36738300	-1.51456600
H	-2.48713200	-4.85856100	-1.63430500
C	-1.47294300	-2.98050700	-1.41184000
C	-2.50215800	-1.97300700	-1.52064900
C	-3.85962100	-2.09351700	-1.79745500
H	-4.29436900	-3.08878500	-1.90931900
C	-4.63089200	-0.94363600	-1.96625900
C	-3.97341500	0.29162500	-1.89829300
H	-4.54537600	1.18606800	-2.13168100
C	-2.60523800	0.46701800	-1.62726700
C	-1.87276400	-0.71353700	-1.38122800
C	2.30711200	-2.41473200	-1.60592100
C	2.53067000	-1.64215200	-2.75389900
H	1.66854400	-1.36700100	-3.36296500
C	3.82828000	-1.31821300	-3.16134400
C	4.89140800	-1.72742000	-2.34443500
H	5.91090900	-1.48118700	-2.65138500
C	4.70592200	-2.48557600	-1.18347500
C	3.39811500	-2.83108800	-0.83814400
H	3.20534400	-3.45836000	0.03029900
C	4.13245900	-0.66138300	-4.51464500
C	2.86172500	-0.25719200	-5.26564700
H	3.12977100	0.21496700	-6.22326500
H	2.26104000	0.46792600	-4.69814500
H	2.22600100	-1.12625000	-5.49242400
C	5.00719700	0.58503100	-4.32653200
H	5.24805200	1.03362900	-5.30352100
H	5.95801200	0.35134500	-3.82554700
H	4.48923600	1.34111400	-3.72140600
C	4.88910400	-1.68388200	-5.38029500
H	5.11219900	-1.25794100	-6.37205300
H	4.28860900	-2.59531700	-5.52457100

H	5.84276900	-1.98117000	-4.91893400
C	5.91615500	-2.95184700	-0.36747400
C	6.83184100	-3.80576800	-1.25743100
H	7.19779300	-3.24248600	-2.12881600
H	6.29810500	-4.69291600	-1.63219500
H	7.71087000	-4.14887700	-0.68814900
C	6.68330100	-1.72363900	0.13844900
H	7.07518200	-1.11894700	-0.69276600
H	7.54114400	-2.02918000	0.75962500
H	6.02788200	-1.07913200	0.74393700
C	5.50468100	-3.79028100	0.84454600
H	6.40058000	-4.10300400	1.40260300
H	4.96182200	-4.70160400	0.55004000
H	4.86813200	-3.21730400	1.53435200
C	-0.37833600	-6.63708300	-1.60685700
C	1.01444600	-7.26324900	-1.50326300
H	1.67236000	-6.94263600	-2.32522500
H	0.93423700	-8.36002400	-1.55416600
H	1.50454500	-7.00803300	-0.55079800
C	-1.24312700	-7.20722600	-0.47414000
H	-2.27690700	-6.83659400	-0.52642700
H	-0.83582300	-6.92501100	0.50851900
H	-1.28097500	-8.30703800	-0.53057200
C	-0.98818400	-7.02722900	-2.96187600
H	-0.38538600	-6.62762200	-3.79189600
H	-2.01106700	-6.63702600	-3.07354500
H	-1.03367800	-8.12360600	-3.06578000
C	-6.11901900	-1.05873500	-2.31291700
C	-6.81514800	-1.98608500	-1.30620200
H	-7.89556600	-2.04032100	-1.51422600
H	-6.68281900	-1.62157900	-0.27570700
H	-6.42240900	-3.01197400	-1.35202500
C	-6.25575800	-1.64875600	-3.72528200
H	-5.76992900	-0.99851300	-4.46894600
H	-7.31742300	-1.75585700	-4.00198100
H	-5.78601200	-2.64179000	-3.79252000
C	-6.82805200	0.29730400	-2.27990400
H	-6.43284800	0.98989500	-3.03850100
H	-6.73368600	0.77791300	-1.29424900
H	-7.90079200	0.16371400	-2.48740000
C	-2.04023800	1.83614500	-1.75570100
C	-2.87288100	2.95472000	-1.63234100
H	-3.89761500	2.80982100	-1.28925900
C	-2.44342100	4.24466100	-1.95379800
C	-1.10344100	4.41596800	-2.30626700
H	-0.73797700	5.41360800	-2.54464200
C	-0.21205500	3.33766600	-2.37754400
C	-0.70574100	2.05447800	-2.13132400
H	-0.06027900	1.19055500	-2.28876000
C	-3.45969000	5.39338800	-1.96534700
C	-4.05733300	5.58233100	-0.56564800
H	-4.82234400	6.37522300	-0.57451500
H	-3.27874900	5.86636500	0.15691700
H	-4.53228100	4.65996800	-0.20343800
C	-4.58755900	5.04080800	-2.94987100
H	-4.18687600	4.87284700	-3.96142500

H	-5.32251100	5.86036600	-3.00199500
H	-5.12504500	4.12971600	-2.64671900
C	-2.83444600	6.71570500	-2.41379600
H	-2.42667500	6.64685600	-3.43401800
H	-2.02554200	7.03540200	-1.74126400
H	-3.59871500	7.50808600	-2.41438600
C	1.23930000	3.56950400	-2.80577500
C	1.27971000	3.73438800	-4.33296700
H	0.88726500	2.83636500	-4.83490000
H	2.31350800	3.89702800	-4.67993100
H	0.67007700	4.59245400	-4.65585700
C	1.80728600	4.83016600	-2.13840200
H	1.72099100	4.76321900	-1.04351500
H	1.29350500	5.74651600	-2.46446200
H	2.87151200	4.94770800	-2.39854900
C	2.12850800	2.39315500	-2.40750700
H	1.87118700	1.46505700	-2.93528800
H	2.07032200	2.18539700	-1.32903700
H	3.17745800	2.61838700	-2.64875400
C	0.02902300	2.27659300	1.30966900
C	1.31686000	2.82540600	1.42607300
C	1.39936000	4.22467500	1.53140500
H	2.40240200	4.63648500	1.64125000
C	0.29484400	5.08417700	1.58179000
C	-0.97180200	4.49664500	1.57462900
H	-1.87231000	5.10300000	1.68979000
C	-1.09666700	3.11654400	1.45113700
C	-2.25001500	2.25221900	1.52924700
C	-3.58444100	2.55240500	1.77930000
H	-3.88424700	3.59639400	1.89013100
C	-4.50583700	1.51543200	1.92145800
C	-4.01579800	0.20478300	1.85955000
H	-4.70610800	-0.60617300	2.07651600
C	-2.67780000	-0.15105300	1.61463800
C	-1.78920300	0.92220800	1.38764400
C	2.59545300	2.09384200	1.63263500
C	2.75063700	1.24679500	2.74346000
H	1.86802600	1.01850900	3.34259800
C	4.01249700	0.79142100	3.12505500
C	5.11611400	1.15504200	2.33246000
H	6.10513000	0.80674300	2.63116900
C	4.99733400	1.97727600	1.21304600
C	3.71802500	2.44161900	0.88357800
H	3.58706400	3.12260400	0.04108000
C	4.26128700	0.00577200	4.41886900
C	2.96914200	-0.27333800	5.18921800
H	2.46438700	0.65764500	5.48864200
H	3.19641500	-0.83778400	6.10662900
H	2.26019100	-0.87405300	4.60252900
C	4.94260700	-1.33125600	4.09556900
H	4.30143600	-1.94914300	3.45220800
H	5.14489000	-1.89508500	5.02037100
H	5.90117700	-1.18950700	3.57452200
C	5.18127000	0.83750600	5.32878300
H	6.15682100	1.03188000	4.85898900
H	5.36560400	0.30554000	6.27616500

H	4.72275600	1.81022900	5.56473700
C	6.19535500	2.42976300	0.37294200
C	7.52067400	1.88288000	0.90692400
H	7.54467800	0.78395400	0.90176200
H	7.71815700	2.22715100	1.93395500
H	8.34998900	2.23505300	0.27437000
C	6.27240800	3.96511400	0.39064700
H	7.12873800	4.31262600	-0.20959800
H	6.39873100	4.34081800	1.41799500
H	5.36506800	4.42681000	-0.02682900
C	6.00075600	1.94632700	-1.07111800
H	6.86240800	2.22988100	-1.69709300
H	5.10065800	2.38732700	-1.52187000
H	5.88178500	0.85410300	-1.11357700
C	0.44606000	6.60301900	1.71275100
C	1.91040200	7.04518300	1.65911300
H	2.39236300	6.74163700	0.71669000
H	2.49642200	6.63100000	2.49360300
H	1.97050500	8.14236700	1.72627300
C	-0.15171500	7.05950400	3.05214600
H	-0.05971900	8.15195200	3.16582400
H	0.36832900	6.58073200	3.89613000
H	-1.21925900	6.80359700	3.12867500
C	-0.30140600	7.28608900	0.55906500
H	-0.19618200	8.38118900	0.62191200
H	-1.37602600	7.05419700	0.57795700
H	0.09538200	6.95524800	-0.41286300
C	-5.97365000	1.82568500	2.23250900
C	-6.51578200	2.83630100	1.21125700
H	-6.40988400	2.45534900	0.18361000
H	-5.98983300	3.80019700	1.26739400
H	-7.58369100	3.03533300	1.39431600
C	-6.85415600	0.57496400	2.17764100
H	-6.79962600	0.08633400	1.19292600
H	-7.90475400	0.84875700	2.35918500
H	-6.57244900	-0.16381300	2.94329900
C	-6.06678300	2.42891500	3.64276300
H	-5.47295600	3.35191300	3.72468300
H	-5.68898100	1.72075300	4.39618300
H	-7.11182600	2.67419200	3.89348100
C	-2.30819700	-1.58448100	1.75244300
C	-3.28489300	-2.57882100	1.61493500
H	-4.27292600	-2.29658500	1.25059500
C	-3.04378800	-3.91357000	1.94708000
C	-1.74652700	-4.26655700	2.32485700
H	-1.52688800	-5.30400700	2.57057200
C	-0.71685600	-3.32156500	2.40978800
C	-1.02446000	-1.98301100	2.15299200
H	-0.26829800	-1.21562100	2.31824800
C	-4.20830700	-4.91196300	1.94051500
C	-4.79462300	-5.02859600	0.52816000
H	-4.04743200	-5.42511700	-0.17445800
H	-5.12795100	-4.05230300	0.14972900
H	-5.66271500	-5.70711700	0.52344300
C	-5.29908700	-4.40040400	2.89658200
H	-5.70035300	-3.42721000	2.57606100

H	-4.90176200	-4.27988600	3.91618100
H	-6.14007100	-5.11155200	2.93619000
C	-3.78075900	-6.30391500	2.40998200
H	-4.64682700	-6.98346100	2.39825400
H	-3.38885600	-6.28289900	3.43849300
H	-3.00966000	-6.73796200	1.75737400
C	0.68382500	-3.74278700	2.86101900
C	0.70568600	-3.81285500	4.39559600
H	-0.02068900	-4.55161500	4.76828400
H	0.44803000	-2.83752700	4.83677200
H	1.70636700	-4.09987600	4.75876100
C	1.05862300	-5.11267100	2.27913100
H	0.98187200	-5.10196200	1.18133000
H	0.41836500	-5.92202000	2.65981900
H	2.09507900	-5.36658700	2.55262300
C	1.73356900	-2.73910900	2.38680000
H	1.67764400	-2.58878700	1.29941700
H	2.74148500	-3.10824800	2.62568700
H	1.63113700	-1.75384300	2.86100500

5 References

[33] A. Hinz, *Chem. Eur. J.* **2019**, *25*, 3267–3271.

[36] L. E. McDyre, T. Hamilton, D. M. Murphy, K. J. Cavell, W. F. Gabrielli, M. J. Hanton, D. M. Smith, *Dalton Trans.* **2010**, *39*, 7792–7799.

[37] G. R. Fulmer, A. J. M. Miller, N. H. Sherden, H. E. Gottlieb, A. Nudelman, B. M. Stoltz, J. E. Bercaw, K. I. Goldberg, *Organometallics* **2010**, *29*, 2176–2179.

[38] G. M. Sheldrick, **1997**, SHELXS-97.

[39] G. M. Sheldrick, *Acta Crystallogr. A* **2015**, *71*, 3–8.

[40] G. M. Sheldrick, **2018**, SHELXL-2018.

[41] C. B. Hübschle, G. M. Sheldrick, B. Dittrich, *J. Appl. Crystallogr.* **2011**, *44*, 1281–1284.

[42] M. J. Frisch, G. W. Trucks, H. B. Schlegel, G. E. Scuseria, M. A. Robb, J. R. Cheeseman, G. Scalmani, V. Barone, B. Mennucci, G. A. Petersson, et al., *Gaussian 16, Revision B.01*, **2016**.

[43] E. D. Glendening, C. R. Landis, F. Weinhold, *J. Comput. Chem.* **2013**, *34*, 1429–1437.

[44] A. Poater, B. Cosenza, A. Correa, S. Giudice, F. Ragone, V. Scarano, L. Cavallo, *Eur. J. Inorg. Chem.* **2009**, *2009*, 1759–1766.

[45] A. Poater, F. Ragone, S. Giudice, C. Costabile, R. Dorta, S. P. Nolan, L. Cavallo, *Organometallics* **2008**, *27*, 2679–2681.

[46] A. Poater, F. Ragone, R. Mariz, R. Dorta, L. Cavallo, *Chem. Eur. J.* **2010**, *16*, 14348–14353.

[47] I. A. Guzei, M. Wendt, *Dalton Trans.* **2006**, 3991–3999.
