# Supplementary figures and images for: A Mono‐Substituted Silicon(II) Cation: A Crystalline “Supersilylene”
Source: Angew Chem Int Ed Engl. 2020 Aug 26;59(43):19065–9. doi: 10.1002/anie.202009874 (PMC7590127; doi:10.1002/anie.202009874)

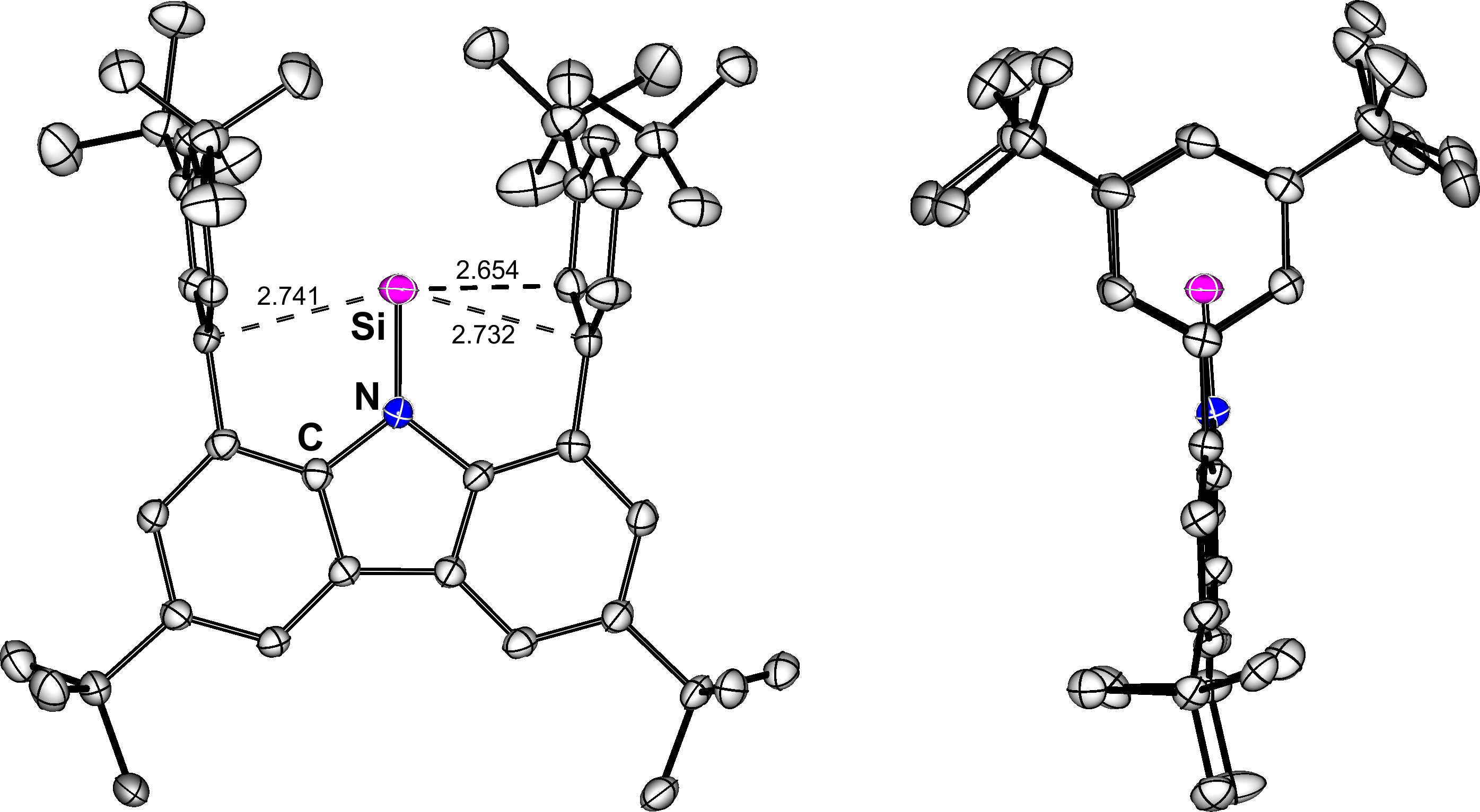

Supplement: Supplementary file 2 — Supplementary [file ANIE-59-19065-s002.tif]

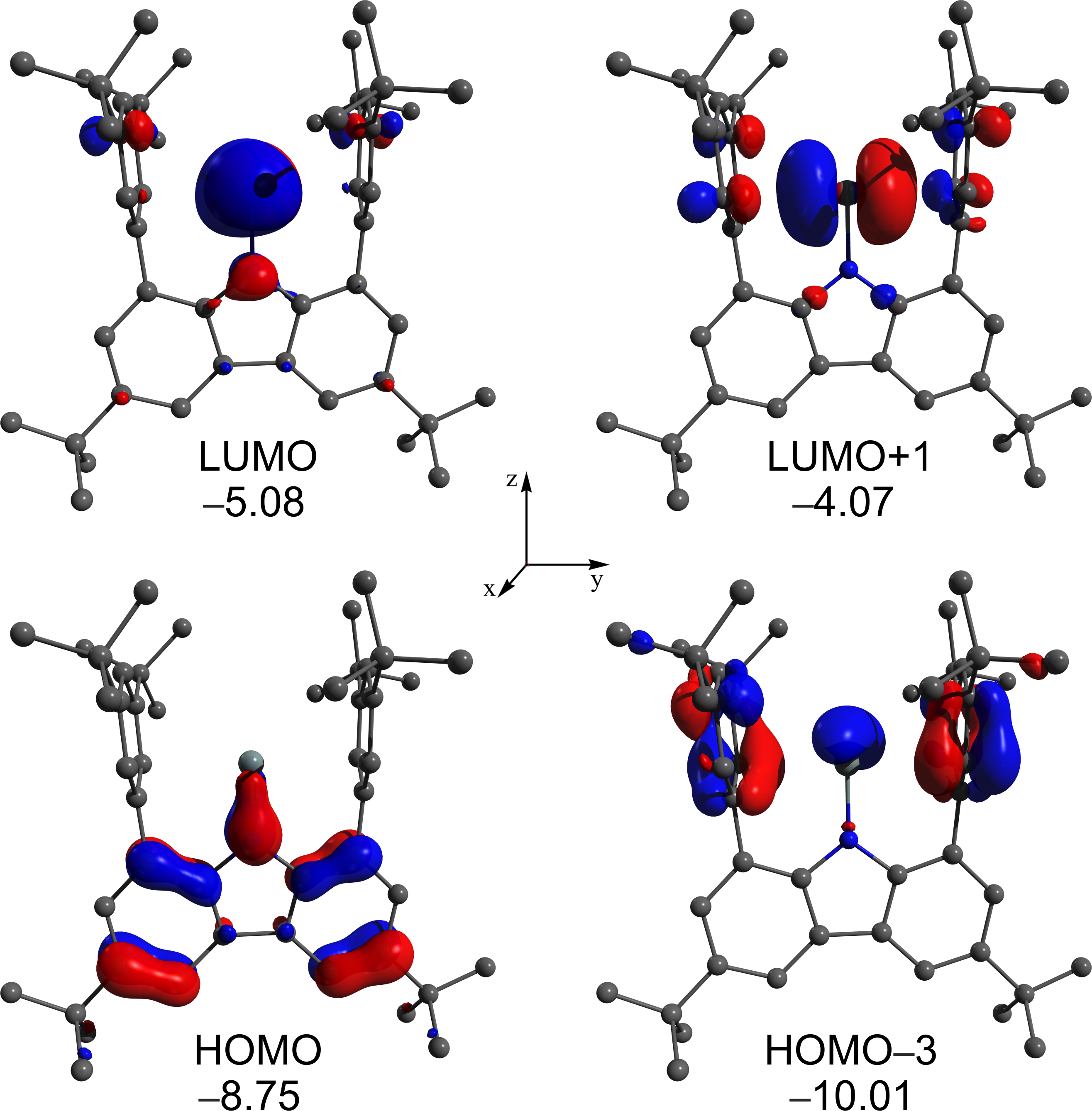

Supplement: Supplementary file 3 — Supplementary [file ANIE-59-19065-s003.tif]

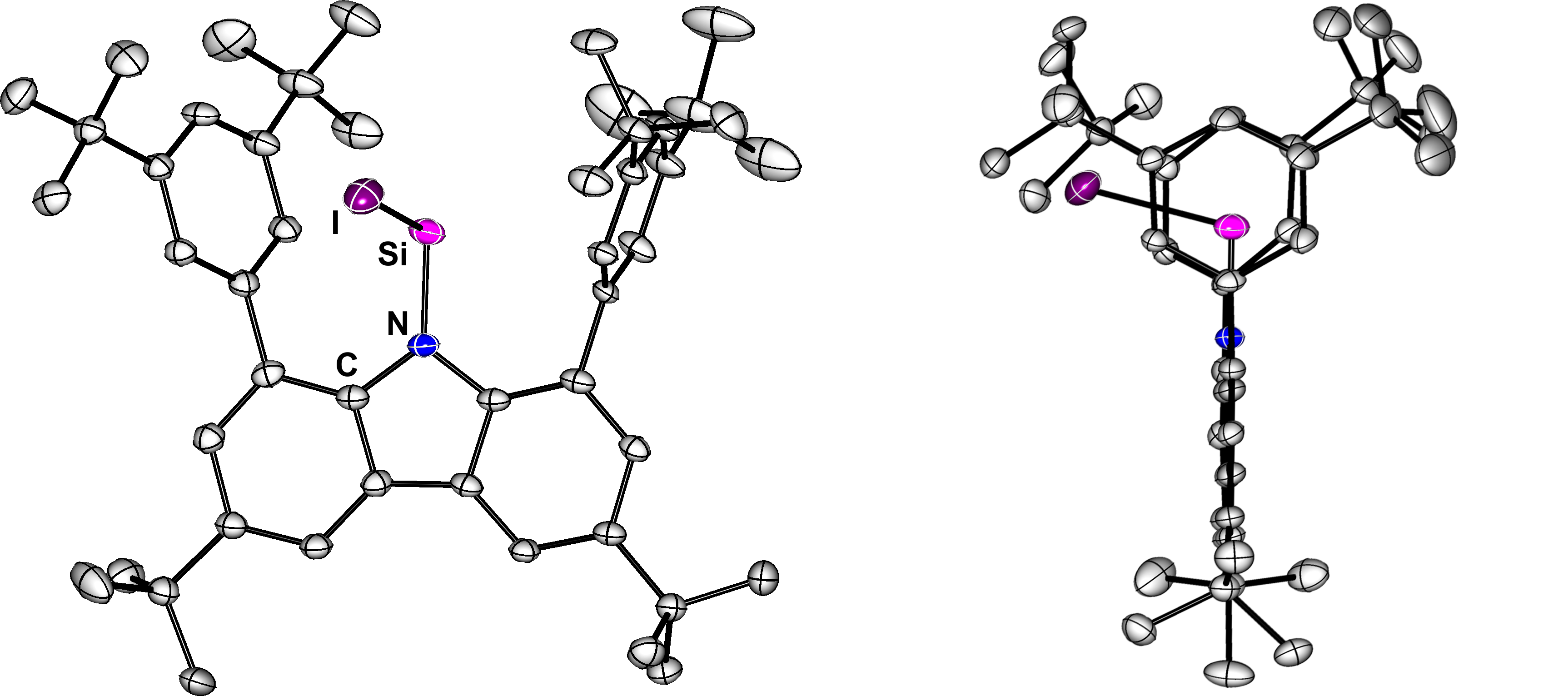

Supplement: Supplementary file 4 — Supplementary [file ANIE-59-19065-s004.tif]

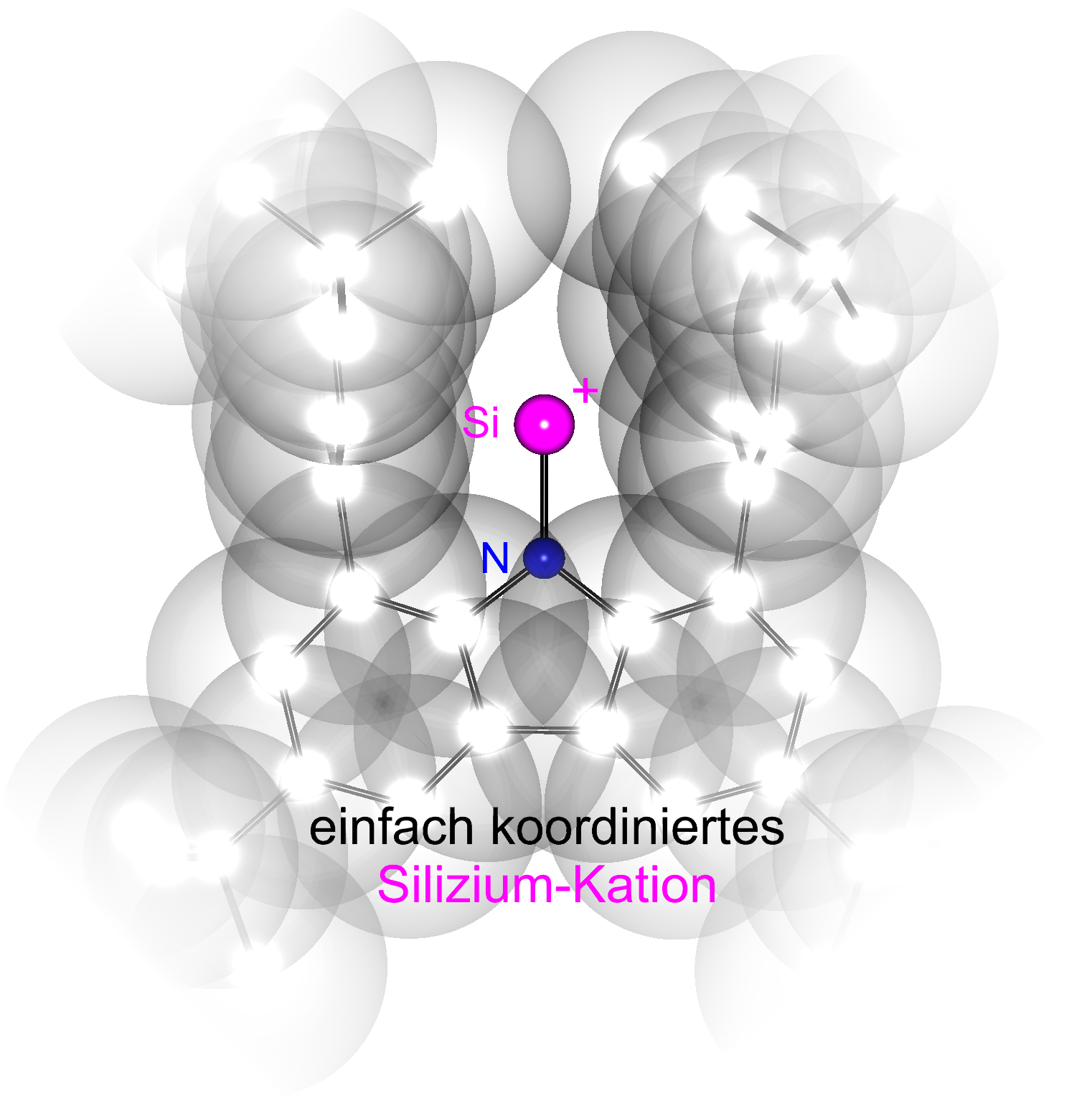

Supplement: Supplementary file 5 — Supplementary [file ANIE-59-19065-s005.tif]

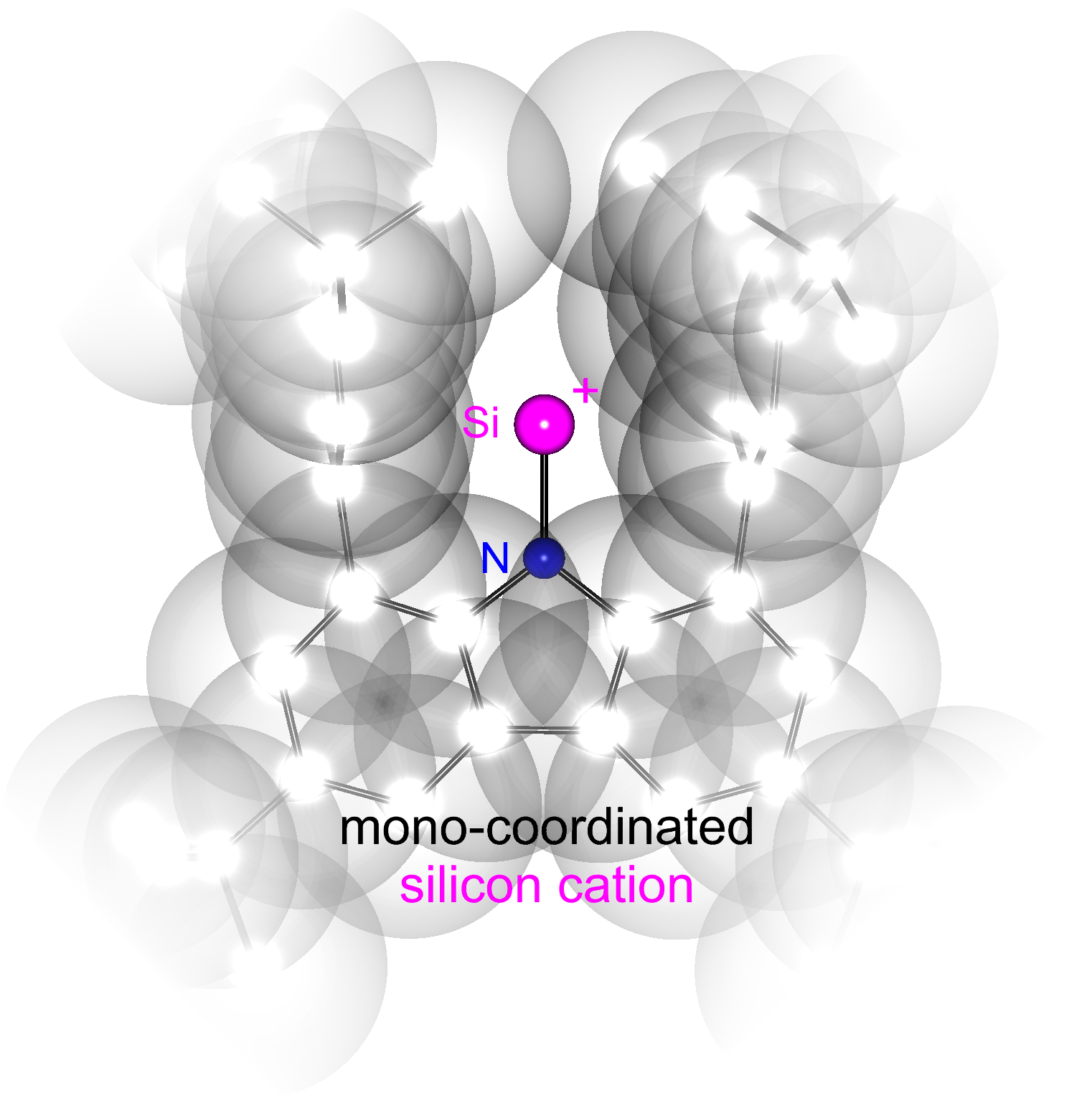

Supplement: Supplementary file 6 — Supplementary [file ANIE-59-19065-s006.tif]
